# Addition of Carboxylic Acids to *gem*-Difluoroalkenes for the Synthesis of *gem*-Difluoromethylenated Compounds

**DOI:** 10.1021/acs.orglett.4c00095

**Published:** 2024-02-01

**Authors:** Yuwei Zong, Gavin Chit Tsui

**Affiliations:** Department of Chemistry, The Chinese University of Hong Kong, Shatin, New Territories 999077, Hong Kong SAR, China

## Abstract

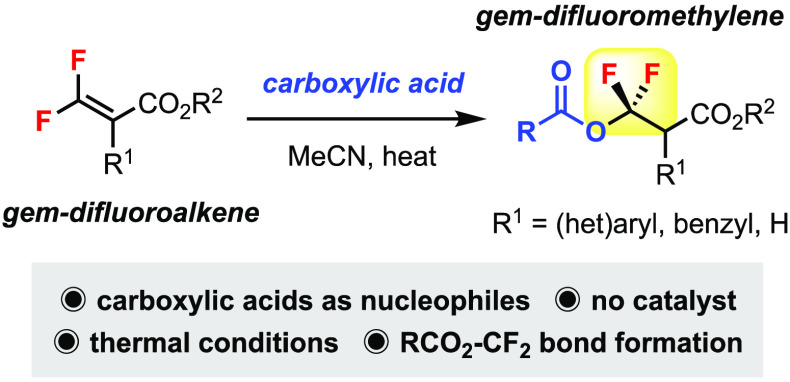

We herein describe a straightforward protocol for the synthesis of carboxylic esters containing a *gem*-difluoromethylene unit. Readily available carboxylic acids can act as nucleophiles to add regioselectively to tetrasubstituted or trisubstituted β,β-difluoroacrylates (formal hydroacetoxylation) for the construction of RCO_2_–CF_2_ bonds. Thermal conditions are sufficient without the use of catalysts or additives.

An increasing level of interest in the synthesis of compounds containing the *gem*-difluoromethylene (-CF_2_-) unit has been seen in recent years.^1^ This is due to the ability of the CF_2_ group to act as a bioisostere for oxygen or carbonyl in pharmaceutical and agrochemical applications.^2^ Despite the existence of various difluoromethylenation methods, the addition of a nucleophile (Nuc-H) to *gem*-difluoroalkenes **1**, a net hydrofunctionalization process, would be one of the simplest approaches for forming difluoromethylenated products **2** (Scheme 1a). It is well-known that *gem*-difluoroalkenes are susceptible to nucleophilic attack at the difluoro position due to the inductive effect of the two strong electron-withdrawing F atoms (Scheme 1b).^3^ However, the β-difluoro anion intermediate thus generated is unstable and rapidly undergoes β-fluoride elimination to form monofluoroalkenes as the major products, resulting in a net C–F bond functionalization.^4^

**Scheme 1:**
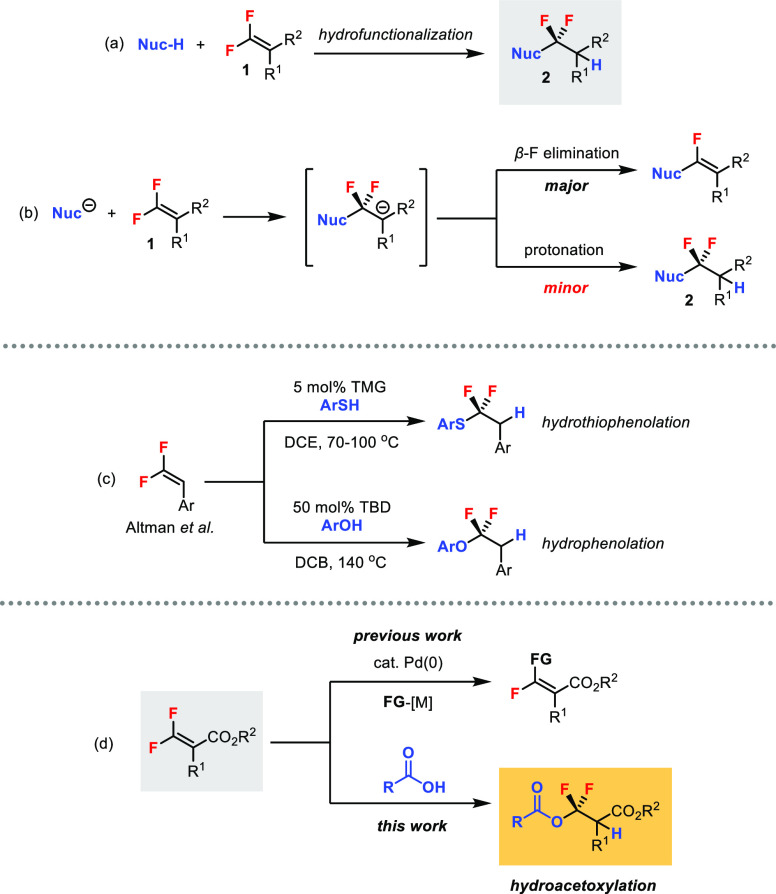
Hydrofunctionalization of *gem*-Difluoroalkenes

Progress has been made in tackling the challenge of shifting the reactivity toward hydrofunctionalization of *gem*-difluoroalkenes via the so-called “fluorine-retentive strategy”.^3a^ Altman and co-workers successfully demonstrated base-catalyzed hydrothiophenolation^5^ and hydrophenolation^5b^ of trisubstituted difluorostyrenes for the construction of S/O–CF_2_ bonds (Scheme 1c). Subsequent development by the same group extended the nucleophile scope to alkyl thiols^5c^ and alcohols^5d^ via acid catalysis and photocatalysis, respectively. Nevertheless, intermolecular hydrofunctionalization of *gem*-difluoroalkenes with heteroatom nucleophiles beyond thiols and alcohols under simple conditions is still very limited.^6^

We have a continuing interest in using *gem*-difluoroalkenes as building blocks for valuable fluorinated molecules. Previously, we have developed a series of palladium-catalyzed stereoselective C–F bond functionalizations of tetrasubstituted β,β-difluoroacrylates for the synthesis of monofluoroalkenes (Scheme 1d).^7^ Herein, we report an unprecedented hydroacetoxylation reaction of the difluoroacrylates, where carboxylic acids can act as effective nucleophiles under catalyst-free conditions. The corresponding carboxylic ester products contain the difluoromethylene unit.

Difluoroalkene **1a** was used as a standard substrate in the initial studies (Scheme 2). Heating **1a** in methanol at 75 °C resulted in a mixture of hydromethoxylation and monofluoroalkene products in low yields (Scheme 2a), and decomposition of the starting material was observed. In stark contrast, heating **1a** in acetic acid gave the desired hydroacetoxylation product **2a** in 92% isolated yield (Scheme 2b). The reaction could be scaled up to 2.0 mmol with a similar yield. Furthermore, substrates containing benzyl (**2b**) and heteroaromatic (**2c**) groups and even a trisubstituted difluoroacrylate (**2d**) afforded the desired products in good yields. Regioselective addition of carboxylic acids to alkenes is not a trivial task,^8^ and no examples of *gem*-difluoroalkenes are known to the best of our knowledge. We also screened other fluorinated and nonfluorinated alkenes under the same conditions for comparison, which were all unreactive (Scheme 2c). These included *gem*-difluoroalkenes **A** and **B** without the ester group, monofluoroalkenes **C** and **D**, and a nonfluorinated alkene as well as *gem*-dichloro/dibromoalkene. The results showed that both the difluoro and the ester functionalities of **1** were important for this reaction.

**Scheme 2:**
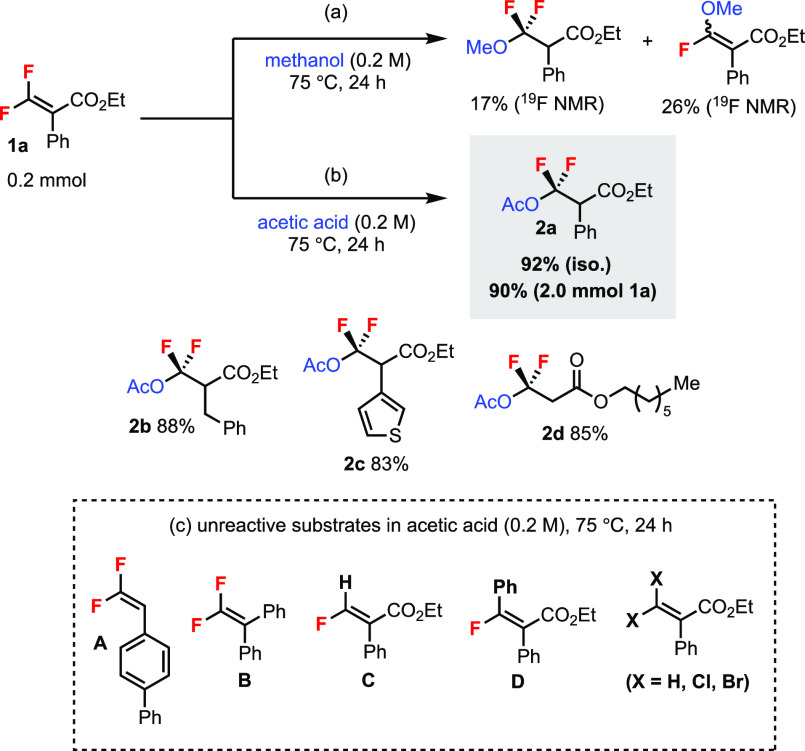
Initial Results

To optimize the reaction conditions further, **1a** was reacted with benzoic acid in different solvents and at different temperatures (Table 1). Several organic solvents were screened at 75 °C using 3.0 equiv of the acid, including DMSO, THF, 1,4-dioxane, and MeCN, and the yields were generally quite low (entries 1–4, respectively). A dramatic increase in the yield was observed when the reaction temperature was increased to 150 °C (entries 5 and 6). Alternatively, increasing the amount of acid to 10 equiv could also maintain a good yield at a lower temperature of 75 °C (entry 7). These two sets of conditions (entries 6 and 7) were used in the subsequent exploration of the reaction scope.

**Table 1:**
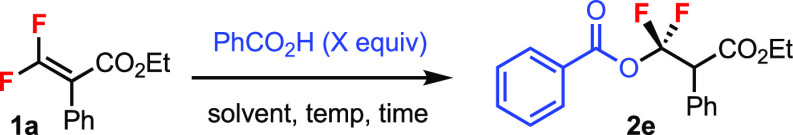
Optimization of the Reaction^a^

entry	X	solvent	temp (°C)/time (h)	yield (%)^b^
1	3.0	DMSO	75/12	27
2	3.0	THF	75/12	37
3	3.0	1,4-dioxane	75/12	9
4	3.0	MeCN	75/12	26
5	3.0	MeCN	120/48	61
6	3.0	MeCN	150/48	93
7	10	MeCN	75/48	90

a Unless specified otherwise, reactions were carried out using 0.1 mmol of **1a** in solvent (0.2 M).

b Determined by ^19^F NMR analysis using benzotrifluoride as an internal standard.

Carboxylic acids are inexpensive, readily available, and structurally diverse commodities. By employing various carboxylic acids as nucleophiles under the optimized conditions, tetrasubstituted *gem*-difluoroalkenes **1** were smoothly transformed into *gem*-difluoromethylenated esters **2e**–**t** (Scheme 3). In most of the examples, a reaction temperature of 75 °C was sufficient to afford good yields (condition A).

**Scheme 3:**
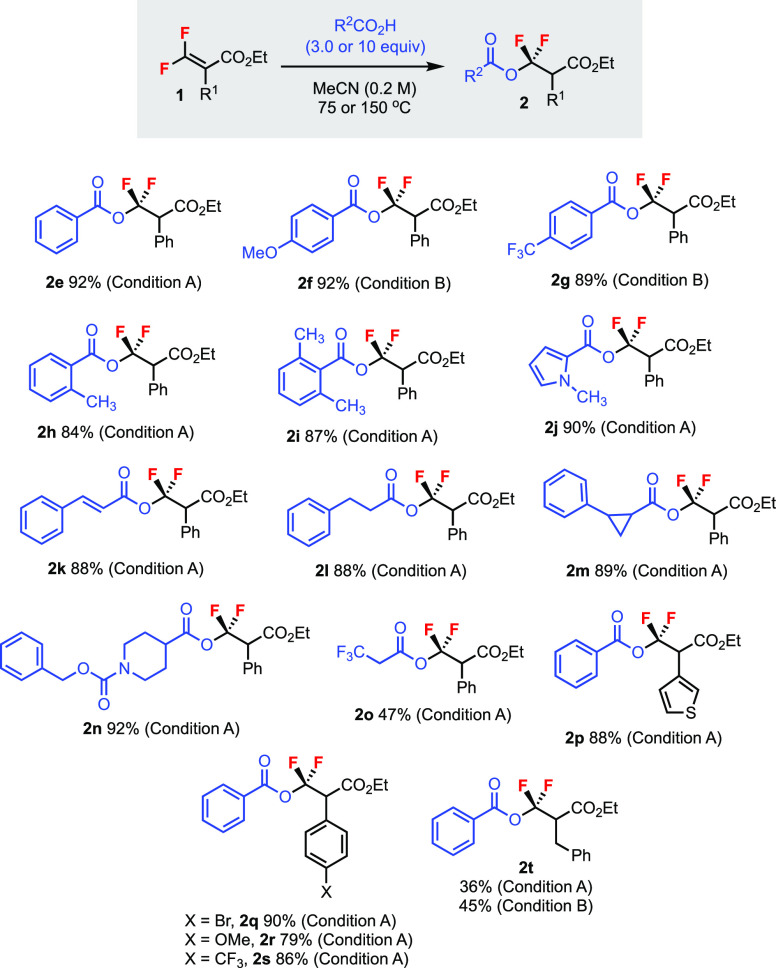
Addition of Carboxylic Acids to Tetrasubstituted *gem*-Difluoroalkenes Unless specified otherwise, reactions were carried out using 0.2 mmol of **1** for 48 h. Isolated yields. Condition A: 10 equiv of carboxylic acid at 75 °C. Condition B: 3.0 equiv of carboxylic acid at 150 °C.

Some carboxylic acids required 150 °C to increase solubility (condition B, **2f** and **2g**). The functional group tolerability of this reaction was excellent. Aromatic carboxylic acids containing electron-rich (**2f**), electron-poor (**2g**), and sterically hindered (**2h** and **2i**) substituents were tolerated. Heteroaryl (**2j**), vinyl (**2k**), and alkyl (**2l**) carboxylic acids were also compatible. Cyclopropane ring (**2m**) and carbamate (**2n**) moieties were preserved in the reaction. However, an electron-deficient carboxylic acid gave a poor yield (**2o**). Substituent R^1^ of difluoroalkene **1** could also be tuned, as shown in products containing heteroaryl (**2p**) and different aryl (**2q**–**s**) groups. On the contrary, the benzyl-substituted substrate (**2t**) gave a yield lower than the aryl ones even at increased temperatures.

Furthermore, the reaction scope could be extended to trisubstituted *gem*-difluoroalkene **3a** in equally good yields (Scheme 4). Various aryl (**4a**), heteroaryl (**4b** and **4c**), and alkyl (**4d**) carboxylic acids were compatible. Drug molecules such as ibuprofen (**4e**), isoxepac (**4f**), and dehydrocholic acid (**4g**) were employed to synthesize the CF_2_-containing ester products. This late-stage functionalization strategy could be attractive in medicinal chemistry for identifying new fluorinated lead compounds.

**Scheme 4:**
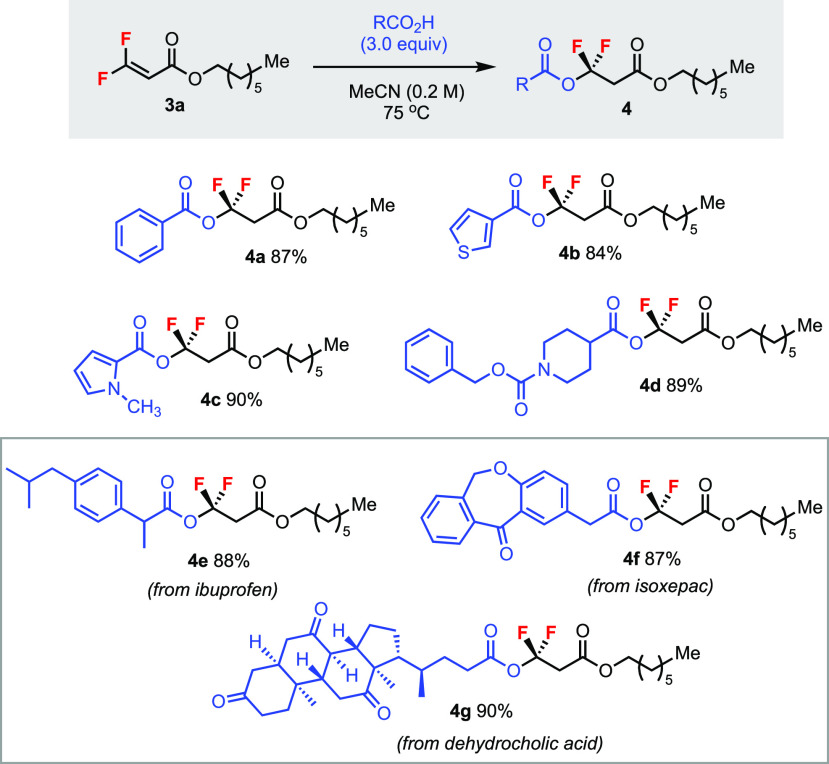
Addition of Carboxylic Acids to a Trisubstituted *gem*-Difluoroalkene Unless specified otherwise, reactions were carried out using 0.2 mmol of **3a** for 24 h. Isolated yields.

Intriguingly, trisubstituted *gem*-difluoroalkene **3a** could react with sulfonic acids to generate products **5** containing the RSO_3_–CF_2_ bond (Scheme 5). Thus, *p*-toluenesulfonic acid and camphor-10-sulfonic acid led to products **5a** (even at 2.0 mmol scale) and **5b**, respectively, in moderate yields. In comparison, a tetrasubstituted substrate such as **1a** did not react with these sulfonic acids. We also tested a BINOL-derived phosphoric acid with **3a** and **1a** but found no reaction.

**Scheme 5:**
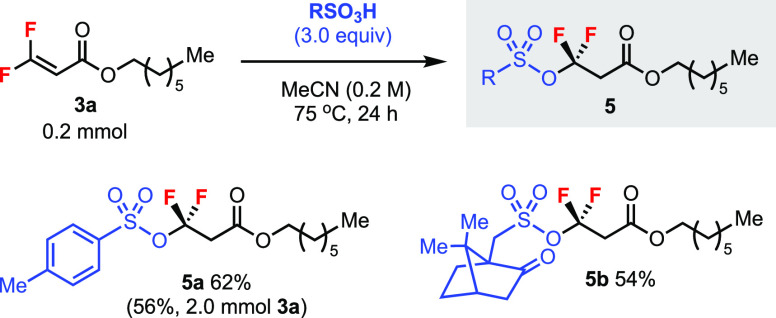
Addition of Sulfonic Acids to a Trisubstituted *gem*-Difluoroalkene

In conclusion, we have discovered a straightforward method for the synthesis of a novel class of *gem*-difluoromethylenated compounds containing RCO_2_–CF_2_ or RSO_3_–CF_2_ bonds. Readily available carboxylic acids can undergo regioselective addition to tetrasubstituted and trisubstituted difluoroacrylates with simple heating. No catalysts or additives were required, and the reaction scope could be extended to sulfonic acids. The reaction mechanism is not completely clear at the moment. Both *gem*-difluoro and ester moieties of **1** were required for the reaction (cf. Scheme 2). We proposed a concerted mechanism (see the Supporting Information) in which the ester group of **1** is protonated by the carboxylic acid while the oxygen of the acid attacks the β-carbon, which is made partially positive due to the two strongly electron-withdrawing F atoms. The resulting enol intermediate tautomerizes to final product **2**. This pathway would also avoid the β-F elimination side product. Further exploration of other types of heteroatom nucleophiles is ongoing in our laboratory.

## Data Availability

The data underlying this study are available in the published article and its Supporting Information.

## Supporting Information Available

Experimental procedures, optimization data, characterization data, and spectral data (PDF)
