# Supplementary material for: Addition of Carboxylic Acids to gem-Difluoroalkenes for the Synthesis of gem-Difluoromethylenated Compounds
Source: Org Lett. 2024 Feb 1;26(6):1261–4. doi: 10.1021/acs.orglett.4c00095 (PMC10877607; doi:10.1021/acs.orglett.4c00095)

Supporting Information

Addition of Carboxylic Acids to *gem*-Difluoroalkenes for the Synthesis of *gem*-Difluoromethylenated Compounds

*Yuwei Zong and Gavin Chit Tsui**

Department of Chemistry, The Chinese University of Hong Kong, Shatin, New Territories, Hong Kong SAR, China
*Email: gctsui@cuhk.edu.hk

Experimental Procedures and Spectral Data

Table of Contents:

General Experimental	**S2**
Materials	**S2**
Instrumentation	**S2**
Experimental Procedures	**S5**
Optimization Studies	**S7**
References	**S8**
Characterization Data	**S9**
Spectra	**S17**

General Experimental.

Unless otherwise noted, reactions were carried out in a 10 mL glass tube with magnetic stirring. Reactions that require heating were carried out in the oil bath. Analytical thin layer chromatography (TLC) was performed with Merck silica gel 60 F_{254} aluminum plates. Visualization was done under a UV lamp (254 nm) and by immersion in potassium permanganate ($KMnO_4$), followed by heating using a heat gun. Organic solutions were concentrated by rotary evaporation at 23-35 °C. Purification of reaction products were generally done by flash column chromatography with Silicycle 60-230 mesh silica gel. Structural assignments were made with additional information from gCOSY, gHSQC, and gHMBC experiments.

Materials.

Gem-difluoroalkenes were prepared according to literature procedure. Acids and other chemicals for substrates preparation were purchased from Acros, J&K Scientific, Aldrich and Dieckmann.

Instrumentation.

Proton nuclear magnetic resonance spectra (^{1}H NMR), carbon nuclear magnetic resonance spectra (^{13}C NMR) and fluorine nuclear magnetic resonance spectra (^{19}F NMR) were recorded at 23 °C on Bruker 400 MHz or 500 MHz spectrometer in $CDCl_3$. Chemical shifts of ^{1}H NMR spectra were reported as parts per million in δ scale using residual solvent signal ($CDCl_3$: 7.26 ppm) or tetramethylsilane (0.00 ppm) as internal standard. Chemical shifts of ^{13}C NMR spectra were reported using residual solvent signal of $CDCl_3$ (77.16 ppm) on the δ scale. Chemical shifts of ^{19}F NMR were reported as parts per million in δ scale using benzotrifluoride (-63.72 ppm) as internal standard. Data are represented as follows: chemical shift (δ ppm), multiplicity (s = singlet, d = doublet, t = triplet, q = quartet, qd = quartet of doublet, p = pentet, m = multiplet), coupling constant (J, Hz) and integration. GC-MS analysis results were obtained on a Shimadzu GCMSQP2010 SE GC-MS Spectrometer. High resolution mass spectra (HRMS) were obtained on a Finnigan MAT 95XL GC Mass Spectrometer or a Thermo Scientific Q Exactive Focus Mass Spectrometer or a Bruker SolariX 9.4T FTMS (mass analyzer type: orbitrap).

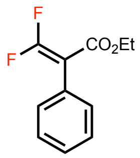

1a

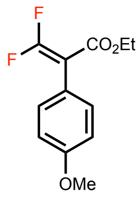

1b

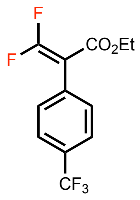

1c

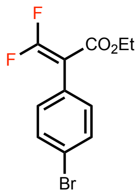

1d

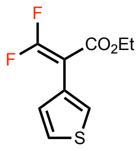

1e

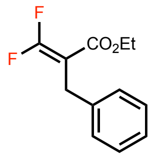

1f

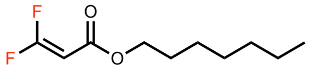

3a

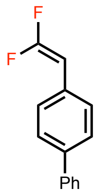

A

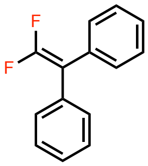

B

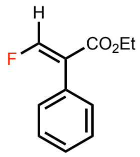

C

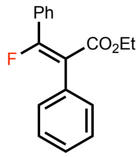

D

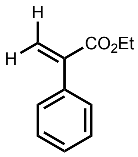

E

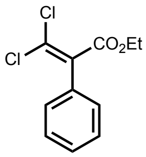

F

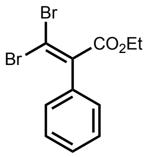

G

Substrates **1**/**3**/**B** are known compounds and prepared according to the literature procedure.[1, 2] Substrate **A** was prepared according to the literature procedure.[3] Substartes **C** and **D** were prepared according to literature procedures.[4,5] Substarte **E** was synthesized according to literature procedures.[6] Substartes **F** and **G** were synthesized according to literature procedures.[7]

Initial results.

Procedure for addition of methanol of *gem*-difuoroalkenes for the synthesis of hydromethoxylatio*n* product:

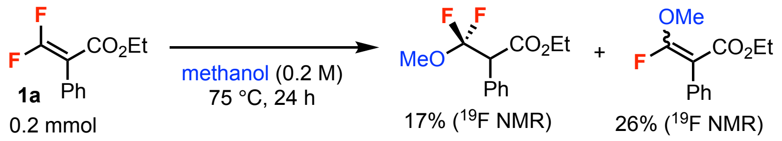

To an oven-dried 10 mL test tube was added **1a** (0.2 mmol, 42.4 mg) and methanol (1.0 mL). The resulting mixture was heated at 75 °C with stirring in an oil bath for 24 h. After cooling to room temperature, the crude product was detected by ^{19}F NMR.

Crude ^{19}F NMR of crude product:

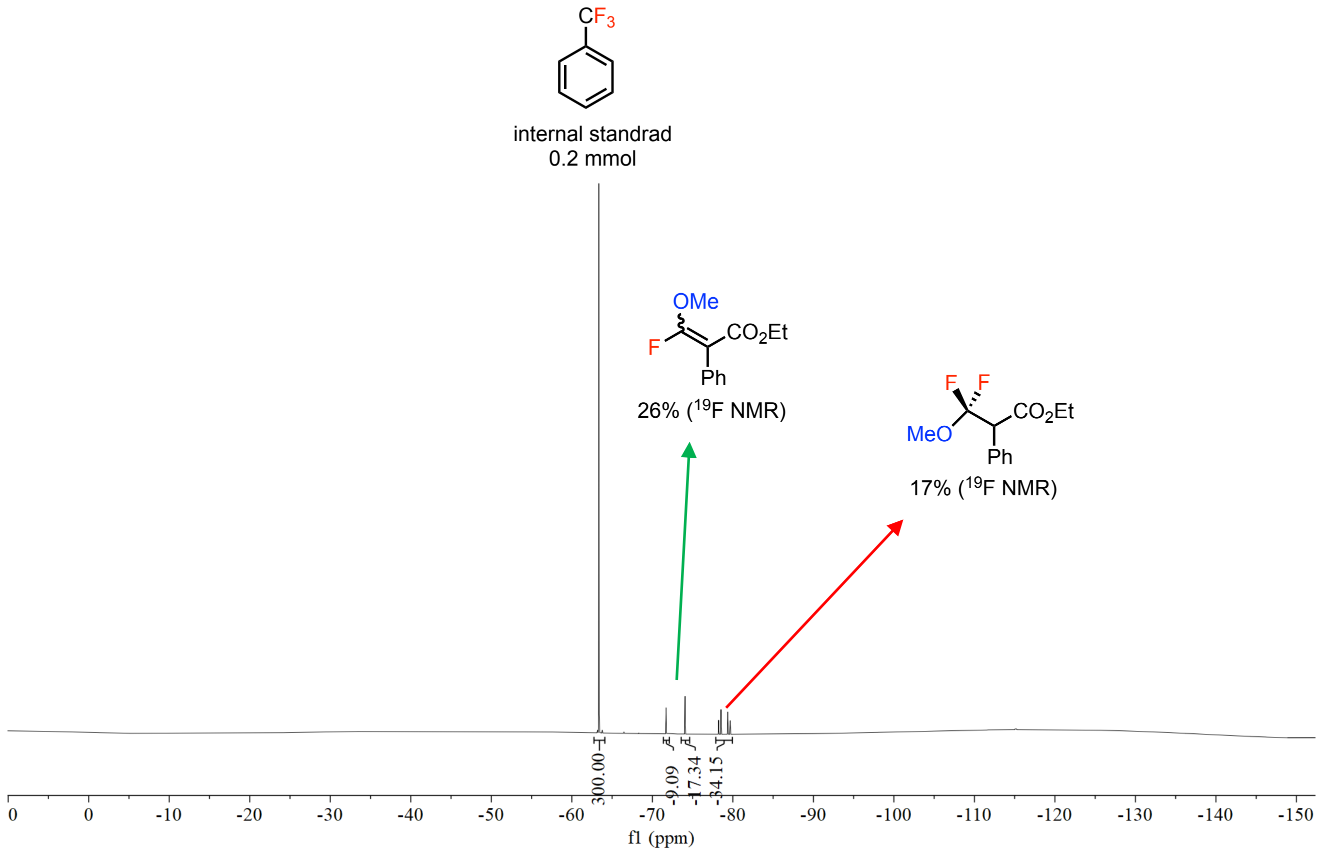

Experimental Procedures.

General procedure (I) for the synthesis of *gem*-difluoroalkenes (using 1a as an example):

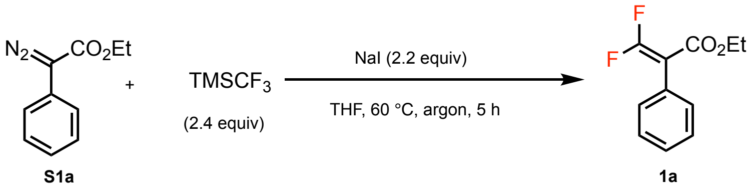

gem-Difluoroalkenes were synthesized according to literature procedure with minor changes.[1,2] For example, to an ovendried 100 mL flask equipped with a stir bar was added NaI (1.6 g, 11 mmol, 2.2 equiv), the solid was heated at 60 °C with an oil bath under vacuo for 30 min (remove H_2O). Then NaI was cooled down to room temperature followed by the addition of **S1a** (951 mg, 5 mmol, 1 equiv) and $TMSCF_3$ (1.7 g, 12 mmol, 2.4 equiv) in 48 mL anhydrous THF under argon. The resulting mixture was heated at 60 °C with an oil bath for 5 h with sharp stirring. After cooling to room temperature, the reaction mixture was extracted with 200 mL Et_2O, washed with H_2O (80 mL) then brine (80 mL), dried over $MgSO_4$ and concentrated in vacuo. The residue was purified by flash column chromatography on silica gel to afford *gem*-difluoroalkene **1a** as a colorless oil (721.5 mg, 68% yield).

General procedure (II) for addition of acetic acid of *gem*-difuoroalkenes for the synthesis of *gem*-difluoromethylenated compounds 2a-d:

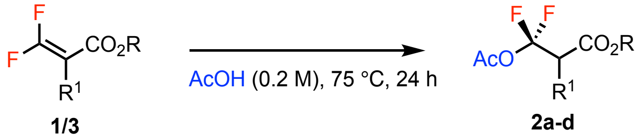

To an oven-dried 10 mL test tube was added **1/3** (0.2 mmol, 1 equiv) and acetic acid (1.0 mL). The resulting mixture was heated at 75 °C with stirring in an oil bath for 24 h. After cooling to room temperature, the reaction mixture was extracted with DCM (3 × 10 mL), combined organic layers were washed with H_2O (2 × 10 mL), then brine (2 × 10 mL), dried over $MgSO_4$ and concentrated in vacuo. The residue was purified by flash column chromatography on silica gel to afford products **2a-d**.

General procedure (III) for addition of carboxylic acid of *gem*-difuoroalkenes for the synthesis of *gem*-difluoromethylenated compounds 2e-t/4/5:

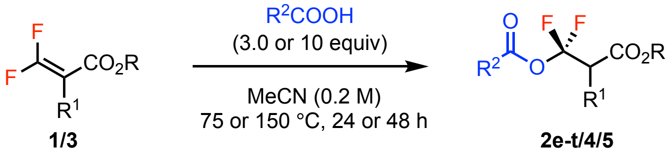

To an oven-dried 10 mL test tube was added **1/3** (0.2 mmol, 1 equiv), carboxylic acid (0.6 mmol or 2 mmol, 3 equiv or 10 equiv) and MeCN (1.0 mL). The resulting mixture was heated at 75 or 150 °C with stirring in an oil bath for 24 or 48 h. After cooling to room temperature, the reaction mixture was extracted with DCM (3 × 10 mL), combined organic layers were washed with H_2O (2 × 10 mL), then brine (2 × 10 mL), dried over $MgSO_4$ and concentrated in vacuo. The residue was purified by flash column chromatography on silica gel to afford products **2e-t/4/5**.

2 mmol scale synthesis of 2a:

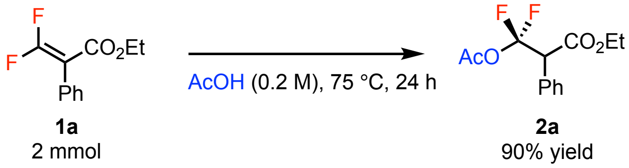

To an oven-dried 50 mL flask was added **1a** (2 mmol, 424.4 mg, 1 equiv) and acetic acid (10 mL). The resulting mixture was heated at 75 °C with stirring in an oil bath for 24 h. After cooling to room temperature, the reaction mixture was extracted with DCM (3 × 30 mL), combined organic layers were washed with H_2O (2 × 30 mL), then brine (2 × 30 mL), dried over $MgSO_4$ and concentrated in vacuo. The residue was purified by flash column chromatography on silica gel (hexane : EA = 75 : 1) to afford product **2a** as a colorless oil (490.1 mg, 90% yield).

2 mmol scale synthesis of 5a:

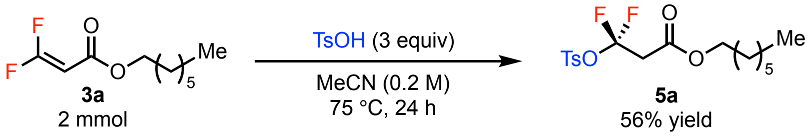

To an oven-dried 50 mL flask was added **3a** (2 mmol, 412.5 mg, 1 equiv), TsOH (6 mmol, 1.03 g, 3 equiv) and MeCN (10 mL). The resulting mixture was heated at 75 °C with stirring in an oil bath for 24 h. After cooling to room temperature, the reaction mixture was extracted with DCM (3 × 30 mL), combined organic layers were washed with H_2O (2 × 30 mL), then brine (2 × 30 mL), dried over $MgSO_4$ and concentrated in vacuo. The residue was purified by flash column chromatography on silica gel (hexane : EA = 15: 1) to afford product **5a** as a white solid (423.6 mg, 56% yield).

Optimization studies.

Table S1. Optimization studies.[a]

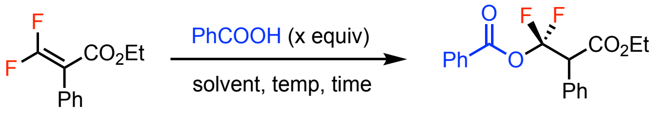

entry	PhCOOH (x equiv)	catalyst (x mol%)	solvent	temp (°C)/time (h)	**2e** (%)[b]
1	3	none	DMSO	75/12	27
2	3	TsOH (50)	DMSO	75/12	0
3	3	none	THF	75/12	37
4	3	none	Dioxane	75/12	9
5	3	none	MeCN	75/12	26
6	3	none	MeCN	120/48	61
**7**	**3**	**none**	**MeCN**	**150/48**	**93**
8	8	none	MeCN	75/48	69
**9**	**10**	**none**	**MeCN**	**75/48**	**90**

[a]Unless specified otherwise, reactions were carried out using 0.1 mmol **1a** in solvent (0.2 M). [b]Determined by ^{19}F NMR analysis using benzotrifluoride as an internal standard.

Mechanistic studies.

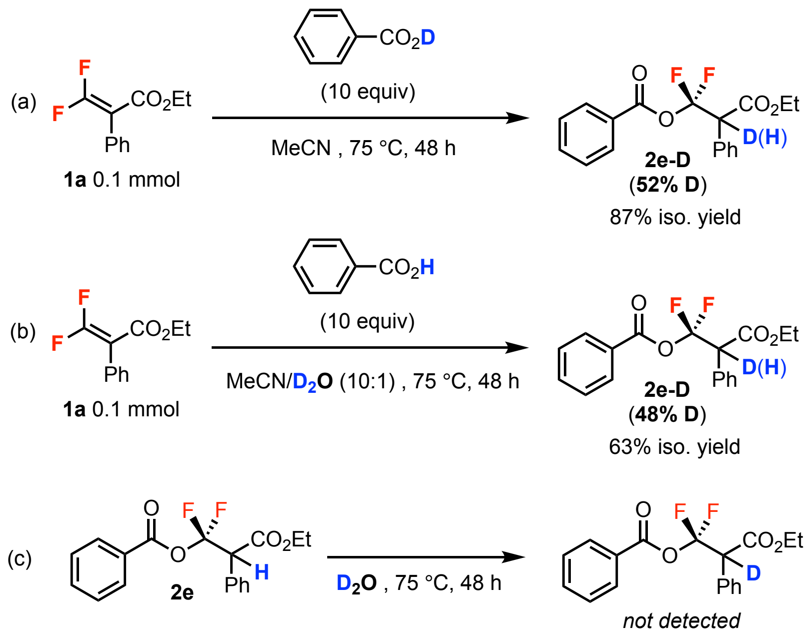

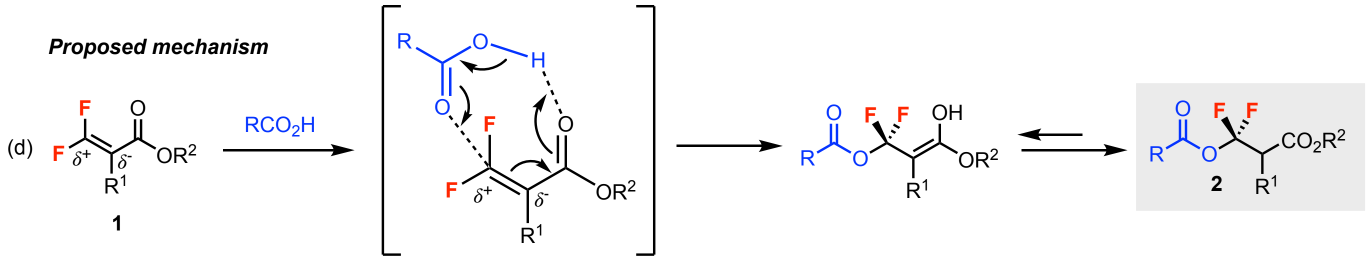

References

1. Hu, M.; Ni, C.; Li, L.; Han, Y.; Hu, J. *gem*-Difluoroolefination of Diazo Compounds with $TMSCF_3$ or $TMSCF_2Br$: Transition-Metal- Free Cross-Coupling of Two Carbene Precursors. *J. Am. Chem. Soc.* **2015**, *137*, 14496-14501.
2. Ma, Q.; Wang, Y.; Tsui, G. C. Stereoselective Palladium-Catalyzed C-F Bond Alkynylation of Tetrasubstituted *gem*-Difluoroalkenes. *Angew. Chem., Int. Ed.* **2020**, *59*, 11293-11297.
3. Liu, J.; Yang, J.; Ferretti, F.; Jackstell, R.; Beller, M. Pd-Catalyzed Selective Carbonylation of *gem*-Difluoroalkenes: A Practical Synthesis of Difluoromethylated Esters. *Angew. Chem., Int. Ed.* **2019**, *58*, 4690-4694.
4. Ma, Q.; Liu, C.; Tsui, G. C. Palladium-Catalyzed Stereoselective Hydrodefluorination of Tetrasubstituted *gem*-Difluoroalkenes. *Org. Lett.* **2020**, *22*, 5193-5197.
5. Wang, Y.; Qi, X.; Ma, Q.; Liu, P.; Tsui, G. C. Stereoselective Palladium-Catalyzed Base-Free Suzuki–Miyaura Cross-Coupling of Tetrasubstituted *gem*-Difluoroalkenes: An Experimental and Computational Study. *ACS Catal.* **2021**, *11*, 4799-4809.
6. Nicely, A. M.; Popov, A. G.; Wendlandt, H. C.; Trammel, G. L.; Kohler, D. G.; Hull, K. L. Cu-Catalyzed Three-Component Carboamination of Electron Deficient Olefins. *Org. Lett.* **2023**, *25*, 5302-5307.
7. Patil, D. V.; Wadia, M. S. A Novel Approach to the Synthesis of 2-Aryl Propionates. *Synth. Commun.* **2002**, *32*, 2821-2827.

Characterization Data.

Characterization data of products:

ethyl 3-acetoxy-3,3-difluoro-2-phenylpropanoate (2a)

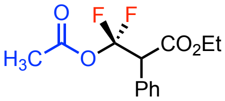

Following the general procedure (II), reaction was run using ethyl 3,3-difluoro-2-phenylacrylate **1a** (42.4 mg, 0.2 mmol) and acetic acid (1.0 mL) for 24 h. The product was purified by flash column chromatography on silica gel (hexane : EA = 75 : 1) and obtained as a colorless oil (50.3 mg, 92% yield), R_f = 0.45 (hexane : EA = 4 : 1). **^{1}H NMR** (500 MHz, $CDCl_3$): δ (ppm) 7.46 - 7.43 (m, 2H), 7.39 - 7.36 (m, 3H), 4.65 (t, *J* = 11.9 Hz, 1H), 4.28 - 4.15 (m, 2H), 2.08 (s, 3H), 1.25 (t, *J* = 7.1 Hz, 3H). **^{13}C NMR** (126 MHz, $CDCl_3$): δ (ppm) 166.9 (d, *J* = 4.1 Hz), 165.5 (t, *J* = 2.3 Hz), 130.5 (d, *J* = 2.7 Hz), 129.9, 129.0, 128.8, 121.2 (dd, *J* = 274.8, 272.9 Hz), 61.9, 56.0 (t, *J* = 27.2 Hz), 21.4, 14.1. **^{19}F NMR** (471 MHz, $CDCl_3$): δ (ppm) -73.45 (dd, *J* = 155.6, 11.7 Hz, 1F), -73.96 (dd, *J* = 155.2, 12.1 Hz, 1F). **HRMS** (ESI) m/z: $[M+Na]^+$ Calcd for $C_{13}H_{14}F_2O_4Na$ 295.0752; Found 295.0753.

ethyl 3-acetoxy-2-benzyl-3,3-difluoropropanoate (2b)

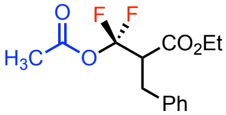

Following the general procedure (II), reaction was run using ethyl 2-benzyl-3,3-difluoroacrylate **1f** (45.2 mg, 0.2 mmol) and acetic acid (1.0 mL) for 24 h. The product was purified by flash column chromatography on silica gel (hexane : EA = 75 : 1) and obtained as a colorless oil (50.6 mg, 88% yield), R_f = 0.48 (hexane : EA = 4 : 1). **^{1}H NMR** (500 MHz, $CDCl_3$): δ (ppm) 7.30 - 7.27 (m, 2H), 7.24 - 7.18 (m, 3H), 4.15 - 4.05 (m, 2H), 3.73 - 3.66 (m, 1H), 3.22 - 3.07 (m, 2H), 2.09 (s, 3H), 1.13 (t, *J* = 7.1 Hz, 3H). **^{13}C NMR** (126 MHz, $CDCl_3$): δ (ppm) 167.7 (d, *J* = 5.4 Hz), 165.3 (t, *J* = 2.3 Hz), 137.0, 129.1, 128.7, 127.1, 121.8 (dd, *J* = 275.7, 273.4 Hz), 61.6, 52.8 (d, *J* = 26.1 Hz), 32.2 (t, *J* = 2.7 Hz), 21.4, 14.1. **^{19}F NMR** (471 MHz, $CDCl_3$): δ (ppm) -74.42 (dd, *J* = 154.3, 9.5 Hz, 1F), -75.41 (dd, *J* = 153.9, 12.2 Hz, 1F). **HRMS** (ESI) m/z: $[M+Na]^+$ Calcd for $C_{14}H_{16}F_2O_4Na$ 309.0909; Found 309.0909.

ethyl 3-acetoxy-3,3-difluoro-2-(thiophen-3-yl)propanoate (2c)

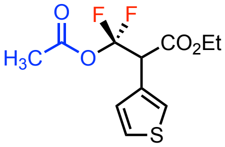

Following the general procedure (II), reaction was run using ethyl 3,3-difluoro-2-(thiophen-3-yl)acrylate **1e** (43.6 mg, 0.2 mmol) and acetic acid (1.0 mL) for 24 h. The product was purified by flash column chromatography on silica gel (hexane : EA = 75 : 1) and obtained as a colorless oil (46.5 mg, 83% yield), R_f = 0.4 (hexane : EA = 4 : 1). **^{1}H NMR** (500 MHz, $CDCl_3$): δ (ppm) 7.40 (d, *J* = 3.4 Hz, 1H), 7.33 (dd, *J* = 5.2, 2.9 Hz, 1H), 7.19 (d, *J* = 5.0 Hz, 1H), 4.82 (t, *J* = 11.8 Hz, 1H), 4.28 - 4.16 (m, 2H), 2.10 (s, 3H), 1.27 (t, *J* = 7.2 Hz, 3H). **^{13}C NMR** (126 MHz, $CDCl_3$): δ (ppm) 166.6 (t, *J* = 2.7 Hz), 165.4 (t, *J* = 2.5 Hz), 129.9 (t, *J* = 2.0 Hz), 128.4, 126.1, 126.0, 121.0 (t, *J* = 274.1 Hz), 62.1, 51.6 (t, *J* = 27.9 Hz), 21.4, 14.1. **^{19}F NMR** (471 MHz, $CDCl_3$): δ (ppm) -74.01 (d, *J* = 11.3 Hz, 2F). **HRMS** (ESI) m/z: $[M+Na]^+$ Calcd for $C_{11}H_{12}F_2O_4SNa$ 301.0317; Found 301.0318.

heptyl 3-acetoxy-3,3-difluoropropanoate (2d)

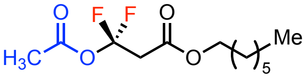

Following the general procedure (II), reaction was run using heptyl 3,3-difluoroacrylate **3a** (41.2 mg, 0.2 mmol) and acetic acid (1.0 mL) for 24 h. The product was purified by flash column chromatography on silica gel (hexane : EA = 100 : 1) and obtained as a colorless oil (45.1 mg, 85% yield), R_f = 0.48 (hexane : EA = 6 : 1). **^{1}H NMR** (500 MHz, $CDCl_3$): δ (ppm) 4.13 (t, *J* = 6.7 Hz, 2H), 3.38 (t, *J* = 11.6 Hz, 2H), 2.18 (s, 3H), 1.64 (p, *J* = 6.7 Hz, 2H), 1.37 - 1.25 (m, 8H), 0.88 (t, *J* = 7.1 Hz, 3H). **^{13}C NMR** (126 MHz, $CDCl_3$): δ (ppm) 166.0 (t, *J* = 2.7 Hz), 165.4 (t, *J* = 5.9 Hz), 120.9 (t, *J* = 266.1 Hz), 65.9, 40.2 (t, *J* = 30.4 Hz), 31.8, 29.0, 28.6, 25.8, 22.7, 21.4, 14.2. **^{19}F NMR** (471 MHz, $CDCl_3$): δ (ppm) -66.99 (t, *J* = 11.7 Hz, 2F). **HRMS** (ESI) m/z: $[M+Na]^+$ Calcd for $C_{12}H_{20}F_2O_4Na$ 289.1222; Found 289.1222.

3-ethoxy-1,1-difluoro-3-oxo-2-phenylpropyl benzoate (2e)

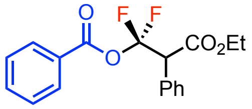

Following the general procedure (III), reaction was run using ethyl 3,3-difluoro-2-phenylacrylate **1a** (42.4 mg, 0.2 mmol) and benzoic acid (2.0 mmol, 244.2 mg) in 1.0 mL MeCN at 75 °C for 48 h. The product was purified by flash column chromatography on silica gel (hexane : EA = 75 : 1) and obtained as a colorless oil (61.2 mg, 92% yield), R_f = 0.52 (hexane : EA = 4 : 1). **^{1}H NMR** (500 MHz, $CDCl_3$): δ (ppm) 7.98 (d, *J* = 8.4 Hz, 2H), 7.62 (t, *J* = 7.5 Hz, 1H), 7.56 - 7.54 (m, 2H), 7.45 (t, *J* = 7.2 Hz, 2H), 7.38 - 7.37 (m, 3H), 4.83 (t, *J* = 11.4 Hz, 1H), 4.30 - 4.16 (m, 2H), 1.23 (t, *J* = 7.2 Hz, 3H). **^{13}C NMR** (126 MHz, $CDCl_3$): δ (ppm) 167.0 (t, *J* = 2.5 Hz), 161.2 (t, *J* = 2.0 Hz), 134.5, 130.4 (t, *J* = 1.8 Hz), 130.3, 130.0, 129.0, 128.8, 128.8, 128.3, 121.8 (t, *J* = 274.8 Hz), 62.0, 56.3 (t, *J* = 27.2 Hz), 14.1. **^{19}F NMR** (471 MHz, $CDCl_3$): δ (ppm) -73.16 (d, *J* = 11.3 Hz, 2F). **HRMS** (ESI) m/z: $[M+Na]^+$ Calcd for $C_{18}H_{16}F_2O_4Na$ 357.0909; Found 357.0910.

3-ethoxy-1,1-difluoro-3-oxo-2-phenylpropyl 4-methoxybenzoate (2f)

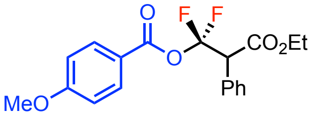

Following the general procedure (III), reaction was run using ethyl 3,3-difluoro-2-phenylacrylate **1a** (42.4 mg, 0.2 mmol) and 4-methoxybenzoic acid (0.6 mmol, 91.3 mg) in 1.0 mL MeCN at 150 °C for 48 h. The product was purified by flash column chromatography on silica gel (hexane : EA = 50 : 1) and obtained as a colorless oil (67.2 mg, 92% yield), R_f = 0.33 (hexane : EA = 4 : 1). **^{1}H NMR** (500 MHz, $CDCl_3$): δ (ppm) 7.93 (d, *J* = 8.9 Hz, 2H), 7.54 (t, *J* = 4.6 Hz, 2H), 7.38 - 7.36 (m, 3H), 6.91 (d, *J* = 8.9 Hz, 2H), 4.82 (t, *J* = 11.5 Hz, 1H), 4.28 - 4.15 (m, 2H), 3.86 (s, 3H), 1.22 (t, *J* = 7.2 Hz, 3H). **^{13}C NMR** (126 MHz, $CDCl_3$): δ (ppm) 167.1 (t, *J* = 2.7 Hz), 164.6, 161.0 (t, *J* = 2.3 Hz), 132.6, 130.6 (t, *J* = 1.8 Hz), 130.0, 129.0, 128.8, 121.8 (t, *J* = 274.1 Hz), 120.5, 114.1, 61.9, 56.3 (t, *J* = 27.5 Hz), 55.7, 14.1. **^{19}F NMR** (471 MHz, $CDCl_3$): δ (ppm) -72.91 (d, *J* = 11.3 Hz, 2F). **HRMS** (ESI) m/z: $[M+Na]^+$ Calcd for $C_{19}H_{18}F_2O_5Na$ 387.1015; Found 387.1015.

3-ethoxy-1,1-difluoro-3-oxo-2-phenylpropyl 4-(trifluoromethyl)benzoate (2g)

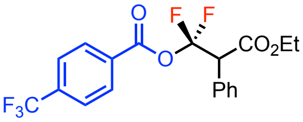

Following the general procedure (III), reaction was run using ethyl 3,3-difluoro-2-phenylacrylate **1a** (42.4 mg, 0.2 mmol) and 4-(trifluoromethyl)benzoic acid (0.6 mmol, 114.1 mg) in 1.0 mL MeCN at 150 °C for 48 h. The product was purified by flash column chromatography on silica gel (hexane : EA = 75 : 1) and obtained as a colorless oil (71.8 mg, 89% yield), R_f = 0.55 (hexane : EA = 4 : 1). **^{1}H NMR** (500 MHz, $CDCl_3$): δ (ppm) 8.09 (d, *J* = 8.2 Hz, 2H), 7.72 (d, *J* = 8.4 Hz, 2H), 7.54 - 7.52 (m, 2H), 7.40 - 7.38 (m, 3H), 4.78 (t, *J* = 11.3 Hz, 1H), 4.30 - 4.17 (m, 2H), 1.24 (t, *J* = 7.2 Hz, 3H). **^{13}C NMR** (126 MHz, $CDCl_3$): δ (ppm) 166.8 (t, *J* = 2.7 Hz), 160.1 (t, *J* = 2.3 Hz), 135.8 (q, *J* = 32.9 Hz), 131.6, 130.7, 130.3 (t, *J* = 2.0 Hz), 129.9, 129.2, 129.0, 125.9 (q, *J* = 3.9 Hz), 123.5 (q, *J* = 272.9 Hz), 121.9 (t, *J* = 275.7 Hz), 62.1, 56.3 (t, *J* = 26.8 Hz), 14.1. **^{19}F NMR** (471 MHz, $CDCl_3$): δ (ppm) -63.34 (s, 3F), -73.01 (dd, *J* = 153.9, 10.8 Hz, 1F), -73.43 (dd, *J* = 153.5, 11.3 Hz, 1F). **HRMS** (ESI) m/z: $[M+Na]^+$ Calcd for $C_{19}H_{15}F_5O_4Na$ 425.0783; Found 425.0782.

3-ethoxy-1,1-difluoro-3-oxo-2-phenylpropyl 2-methylbenzoate (2h)

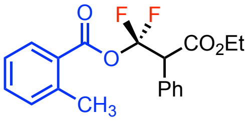

Following the general procedure (III), reaction was run using ethyl 3,3-difluoro-2-phenylacrylate **1a** (42.4 mg, 0.2 mmol) and 2-methylbenzoic acid (2.0 mmol, 272.3 mg) in 1.0 mL MeCN at 75 °C for 48 h. The product was purified by flash column chromatography on silica gel (hexane : EA = 75 : 1) and obtained as a colorless oil (57.9 mg, 84% yield), R_f = 0.52 (hexane : EA = 4 : 1). **^{1}H NMR** (500 MHz, $CDCl_3$): δ (ppm) 7.83 (d, *J* = 7.8 Hz, 1H), 7.57 - 7.55 (m, 2H), 7.48 (t, *J* = 7.5 Hz, 1H), 7.41 - 7.40 (m, 3H), 7.29 - 7.25 (m, 2H), 4.85 (t, *J* = 11.6 Hz, 1H), 4.32 - 4.19 (m, 2H), 2.59 (s, 3H), 1.27 (t, *J* = 7.1 Hz, 3H). **^{13}C NMR** (126 MHz, $CDCl_3$): δ (ppm) 167.1 (d, *J* = 4.5 Hz), 161.5 (t, *J* = 2.0 Hz), 142.1, 133.5, 132.1, 131.3, 130.6 (d, *J* = 3.2 Hz), 130.0, 129.0, 128.8, 127.2, 126.1, 121.9 (t, *J* = 274.1 Hz), 62.0, 56.3 (t, *J* = 27.5 Hz), 21.8, 14.1. **^{19}F NMR** (471 MHz, $CDCl_3$): δ (ppm) -72.85 (dd, *J* = 155.2, 11.3 Hz, 1F), -73.38 (dd, *J* = 154.3, 12.1 Hz, 1F). **HRMS** (ESI) m/z: $[M+Na]^+$ Calcd for $C_{19}H_{18}F_2O_4Na$ 371.1065; Found 371.1066.

3-ethoxy-1,1-difluoro-3-oxo-2-phenylpropyl 2,6-dimethylbenzoate (2i)

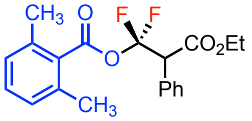

Following the general procedure (III), reaction was run using ethyl 3,3-difluoro-2-phenylacrylate **1a** (42.4 mg, 0.2 mmol) and 2,6-dimethylbenzoic acid (2.0 mmol, 300.4 mg) in 1.0 mL MeCN at 75 °C for 48 h. The product was purified by flash column chromatography on silica gel (hexane : EA = 75 : 1) and obtained as a colorless oil (63.2 mg, 87% yield), R_f = 0.53 (hexane : EA = 4 : 1). **^{1}H NMR** (500 MHz, $CDCl_3$): δ (ppm) 7.51- 7.49 (m, 2H), 7.38 - 7.37 (m, 3H), 7.21 (t, *J* = 7.6 Hz, 1H), 7.01 (d, *J* = 7.6 Hz, 2H), 4.80 (t, *J* = 11.9 Hz, 1H), 4.27 - 4.15 (m, 2H), 2.21 (s, 6H), 1.23 (t, *J* = 7.2 Hz, 3H). **^{13}C NMR** (126 MHz, $CDCl_3$): δ (ppm) 166.9 (d, *J* = 3.6 Hz), 164.3 (t, *J* = 2.5 Hz), 135.8, 131.5, 130.5, 130.4 (d, *J* = 3.2 Hz), 130.1, 129.1, 128.8, 127.9, 121.9 (dd, *J* = 277.0, 273.8 Hz), 62.0, 56.5 (t, *J* = 26.8 Hz), 19.6, 14.1. **^{19}F NMR** (471 MHz, $CDCl_3$): δ (ppm) -72.62 (dd, *J* = 155.6, 10.8 Hz, 1F), -73.74 (dd, *J* = 155.6, 13.4 Hz, 1F). **HRMS** (ESI) m/z: $[M+Na]^+$ Calcd for $C_{20}H_{20}F_2O_4Na$ 385.1222; Found 385.1223.

3-ethoxy-1,1-difluoro-3-oxo-2-phenylpropyl 1-methyl-1H-pyrrole-2-carboxylate (2j)

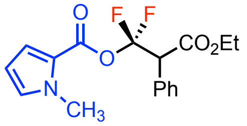

Following the general procedure (III), reaction was run using ethyl 3,3-difluoro-2-phenylacrylate **1a** (42.4 mg, 0.2 mmol) and 1-methyl-*1H*-pyrrole-2-carboxylic acid (2.0 mmol, 250.3 mg) in 1.0 mL MeCN at 75 °C for 48 h. The product was purified by flash column chromatography on silica gel (hexane : EA = 20 : 1) and obtained as a colorless oil (60.3 mg, 90% yield), R_f = 0.58 (hexane : EA = 2 : 1). **^{1}H NMR** (500 MHz, $CDCl_3$): δ (ppm) 7.55 - 7.54 (m, 2H), 7.41 - 7.36 (m, 3H), 6.93 (d, *J* = 4.1 Hz, 1H), 6.86 (s, 1H), 6.12 (t, *J* = 3.6 Hz, 1H), 4.70 (t, *J* = 11.1 Hz, 1H), 4.30 - 4.16 (m, 2H), 3.88 (s, 3H), 1.24 (t, *J* = 7.2 Hz, 3H). **^{13}C NMR** (126 MHz, $CDCl_3$): δ (ppm) 167.1 (t, *J* = 2.5 Hz), 155.0 (t, *J* = 2.3 Hz), 132.0, 130.6 (t, *J* = 1.8 Hz), 129.9, 128.9, 128.7, 121.6 (t, *J* = 274.1 Hz), 120.7, 120.2, 108.8, 61.9, 56.7 (t, *J* = 27.9 Hz), 36. 9, 14.1. **^{19}F NMR** (471 MHz, $CDCl_3$): δ (ppm) -72.37 (dd, *J* = 152.9, 10.6 Hz, 1F), -72.40 (dd, *J* = 153.1, 11.3 Hz, 1F). **HRMS** (ESI) m/z: $[M+Na]^+$ Calcd for $C_{17}H_{17}F_2NO_4Na$ 360.1018; Found 360.1020.

3-ethoxy-1,1-difluoro-3-oxo-2-phenylpropyl cinnamate (2k)

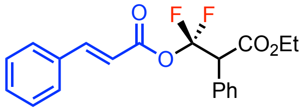

Following the general procedure (III), reaction was run using ethyl 3,3-difluoro-2-phenylacrylate **1a** (42.4 mg, 0.2 mmol) and cinnamic acid (2.0 mmol, 296.3 mg) in 1.0 mL MeCN at 75 °C for 48 h. The product was purified by flash column chromatography on silica gel (hexane : EA = 75 : 1) and obtained as a colorless oil (63.4 mg, 88% yield), R_f = 0.45 (hexane : EA = 4 : 1). **^{1}H NMR** (400 MHz, $CDCl_3$): δ (ppm) 7.72 (d, *J* = 16.0 Hz, 1H), 7.54 - 7.50 (m, 4H), 7.45 - 7.38 (m, 6H), 6.33 (d, *J* = 16.0 Hz, 1H), 4.81 (t, *J* = 11.9 Hz, 1H), 4.31 - 4.16 (m, 2H), 1.26 (t, *J* = 7.2 Hz, 3H). **^{13}C NMR** (101 MHz, $CDCl_3$): δ (ppm) 167.0 (d, *J* = 3.7 Hz), 161.3 (t, *J* = 2.2 Hz), 148.5, 133.6, 131.4, 130.6 (d, *J* = 2.9 Hz), 129.9, 129.1, 129.0, 128.8, 128.6, 121.6 (t, *J* = 273.7 Hz), 115.9, 61.9, 56.1 (t, *J* = 27.3 Hz), 14.1. **^{19}F NMR** (471 MHz, $CDCl_3$): δ (ppm) -72.75 (dd, *J* = 155.2, 11.3 Hz, 1F), -73.28 (dd, *J* = 155.6, 12.6 Hz, 1F). **HRMS** (ESI) m/z: $[M+Na]^+$ Calcd for $C_{20}H_{18}F_2O_4Na$ 383.1065; Found 383.1067.

ethyl 3,3-difluoro-2-phenyl-3-((3-phenylpropanoyl)oxy)propanoate (2l)

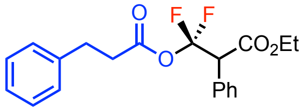

Following the general procedure (III), reaction was run using ethyl 3,3-difluoro-2-phenylacrylate **1a** (42.4 mg, 0.2 mmol) and 3-phenylpropanoic acid (2.0 mmol, 300.4 mg) in 1.0 mL MeCN at 75 °C for 48 h. The product was purified by flash column chromatography on silica gel (hexane : EA = 75 : 1) and obtained as a colorless oil (64.0 mg, 88% yield), R_f = 0.52 (hexane : EA = 4 : 1). **^{1}H NMR** (500 MHz, $CDCl_3$): δ (ppm) 7.44 - 7.42 (m, 2H), 7.39 - 7.34 (m, 3H), 7.30 (t, *J* = 7.6 Hz, 2H), 7.23 (t, *J* = 6.8 Hz, 1H), 7.16 (d, *J* = 7.6 Hz, 2H), 4.65 (t, *J* = 11.8 Hz, 1H), 4.27 - 4.14 (m, 2H), 2.89 (t, *J* = 7.8 Hz, 2H), 2.71 - 2.58 (m, 2H), 1.25 (t, *J* = 7.1 Hz, 3H). **^{13}C NMR** (126 MHz, $CDCl_3$): δ (ppm) 167.4 (t, *J* = 2.3 Hz), 166.9 (d, *J* = 3.6 Hz), 139.6, 130.4 (d, *J* = 3.2 Hz), 129.9, 129.0, 128.8, 128.7, 128.4, 126.7, 121.3 (dd, *J* = 276.1, 272.9 Hz), 61.9, 56.0(t, *J* = 27.0 Hz), 36.1, 30.2, 14.1. **^{19}F NMR** (471 MHz, $CDCl_3$): δ (ppm) -72.97 (dd, *J* = 154.8, 10.8 Hz, 1F), -74.01 (dd, *J* = 154.8, 12.6 Hz, 1F). **HRMS** (ESI) m/z: $[M+Na]^+$ Calcd for $C_{20}H_{20}F_2O_4Na$ 385.1222; Found 385.1223.

3-ethoxy-1,1-difluoro-3-oxo-2-phenylpropyl 2-phenylcyclopropane-1-carboxylate (2m)

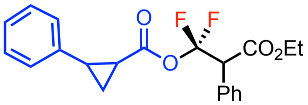

Following the general procedure (III), reaction was run using ethyl 3,3-difluoro-2-phenylacrylate **1a** (42.4 mg, 0.2 mmol) and 2-phenylcyclopropane-1-carboxylic acid (2.0 mmol, 324.4 mg) in 1.0 mL MeCN at 75 °C for 48 h. The product was purified by flash column chromatography on silica gel (hexane : EA = 75 : 1) and obtained as a colorless oil (66.2 mg, 89% yield), R_f = 0.48 (hexane : EA = 4 : 1). **^{1}H NMR** (500 MHz, $CDCl_3$): δ (ppm) 7.40 - 7.31 (m, 6H), 7.28 -7.26 (m, 2H), 7.24 (d, *J* = 7.3 Hz, 2H), 4.46 (dd, *J* = 12.3, 9.2 Hz, 1H), 4.24 - 4.13 (m, 2H), 1.74 - 1.69 (m, 1H), 1.60 - 1.55 (m, 1H), 1.32 - 1.23 (m, 5H). **^{13}C NMR** (126 MHz, $CDCl_3$): δ (ppm) 169.0, 166.7 (t, *J* = 2.3 Hz), 137.8, 130.7, 130.4 (d, *J* = 1.4 Hz), 129.9, 128.8, 128.7, 128.4, 127.7, 121.2 (dd, *J* = 276.6, 274.3 Hz), 61.8, 56.2 (t, *J* = 27.5 Hz), 29.4, 17.9, 17.7, 14.1. **^{19}F NMR** (471 MHz, $CDCl_3$): δ (ppm) -72.72 (dd, *J* = 152.2, 9.1 Hz, 1F), -74.91 (dd, *J* = 152.2, 12.6 Hz, 1F). **HRMS** (ESI) m/z: $[M+Na]^+$ Calcd for $C_{21}H_{20}F_2O_4Na$ 397.1222; Found 397.1226.

1-benzyl 4-(3-ethoxy-1,1-difluoro-3-oxo-2-phenylpropyl) piperidine-1,4-dicarboxylate (2n)

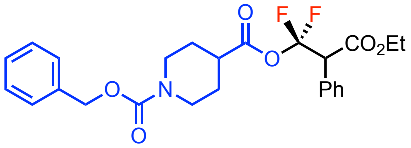

Following the general procedure (III), reaction was run using ethyl 3,3-difluoro-2-phenylacrylate **1a** (42.4 mg, 0.2 mmol) and 1-((benzyloxy)carbonyl)piperidine-4-carboxylic acid (2.0 mmol, 526.6 mg) in 1.0 mL MeCN at 75 °C for 48 h. The product was purified by flash column chromatography on silica gel (hexane : EA = 20 : 1) and obtained as a white solid (87.8 mg, 92% yield), R_f = 0.45 (hexane : EA = 2 : 1). **^{1}H NMR** (500 MHz, $CDCl_3$): δ (ppm) 7.44 - 7.30 (m, 10H), 5.12 (s, 2H), 4.67 (dd, *J* = 13.2, 10.5 Hz, 1H), 4.26 - 4.14 (m, 2H), 4.06 - 4.0 (m, 2H), 2.90 (q, *J* = 12.6 Hz, 2H), 2.47 (tt, *J* = 10.7, 4.0 Hz, 1H), 1.85 - 1.72 (m, 2H), 1.63 - 1.47 (m, 2H), 1.24 (t, *J* = 7.1 Hz, 3H). **^{13}C NMR** (126 MHz, $CDCl_3$): δ (ppm) 168.9 (t, *J* = 1.8 Hz), 166.8 (d, *J* = 3.6 Hz), 136.7, 130.4 (d, *J* = 3.2 Hz), 129.8, 129.1, 128.8, 128.6, 128.2, 128.0, 121.5 (dd, *J* = 276.6, 272.5 Hz), 67.3, 62.0, 55.9 (t, *J* = 27.0 Hz), 42.9, 41.1, 27.2, 14.1. **^{19}F NMR** (471 MHz, $CDCl_3$): δ (ppm) -72.50 (dd, *J* = 155.2, 84.1 Hz, 1F), -73.95 (d, *J* = 156.1 Hz, 1F). **HRMS** (ESI) m/z: $[M+Na]^+$ Calcd for $C_{25}H_{27}F_2NO_6Na$ 498.1699; Found 498.1704.

ethyl 3,3-difluoro-2-phenyl-3-((3,3,3-trifluoropropanoyl)oxy)propanoate (2o)

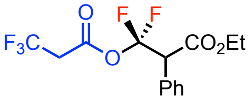

Following the general procedure (III), reaction was run using ethyl 3,3-difluoro-2-phenylacrylate **1a** (42.4 mg, 0.2 mmol) and 3,3,3-trifluoropropanoic acid (2.0 mmol, 256.1 mg) in 1.0 mL MeCN at 75 °C for 48 h. The product was purified by flash column chromatography on silica gel (hexane : EA = 75 : 1) and obtained as a colorless oil (32.3 mg, 47% yield), R_f = 0.48 (hexane : EA = 4 : 1). **^{1}H NMR** (500 MHz, $CDCl_3$): δ (ppm) 7.45 - 7.36 (m, 5H), 4.61 (t, *J* = 11.8 Hz, 1H), 4.28 - 4.15 (m, 2H), 3.28 - 3.13 (m, 2H), 1.25 (t, *J* = 7.2 Hz, 3H). **^{13}C NMR** (126 MHz, $CDCl_3$): δ (ppm) 166.55 - 166.51 (m), 158.8 - 158.7 (m), 129.9, 129.3, 129.0, 122.7 (q, *J* = 276.6 Hz), 121.5 (t, *J* = 277.3 Hz), 62.2, 55.9 (t, *J* = 26.3 Hz), 39.9 (q, *J* = 32.2 Hz), 14.0. **^{19}F NMR** (471 MHz, $CDCl_3$): δ (ppm) -63.45 (t, *J* = 9.5 Hz, 3F). -73.51 (dd, *J* = 154.2, 12.1 Hz, 1F), -73.91 (dd, *J* = 153.5, 12.1 Hz, 1F). **HRMS** (ESI) m/z: $[M+Na]^+$ Calcd for $C_{14}H_{13}F_5O_4Na$ 363.0626; Found 363.0627.

3-ethoxy-1,1-difluoro-3-oxo-2-(thiophen-3-yl)propyl benzoate (2p)

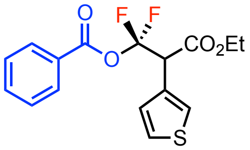

Following the general procedure (III), reaction was run using ethyl 3,3-difluoro-2-(thiophen-3-yl)acrylate **1e** (43.6 mg, 0.2 mmol) and benzoic acid (2.0 mmol, 244.2 mg) in 1.0 mL MeCN at 75 °C for 48 h. The product was purified by flash column chromatography on silica gel (hexane : EA = 75 : 1) and obtained as a colorless oil (59.6 mg, 88% yield), R_f = 0.43 (hexane : EA = 4 : 1). **^{1}H NMR** (500 MHz, $CDCl_3$): δ (ppm) 8.01 (d, *J* = 6.9 Hz, 2H), 7.65 (t, *J* = 7.5 Hz, 1H), 7.50 - 7.47 (m, 3H), 7.35 (dd, *J* = 5.1, 3.0 Hz, 1H), 7.29 (d, *J* = 4.1 Hz, 1H), 5.00 (t, *J* = 11.4 Hz, 1H), 4.31 - 4.19 (m, 2H), 1.25 (t, *J* = 7.1 Hz, 3H). **^{13}C NMR** (126 MHz, $CDCl_3$): δ (ppm) 166.7 (d, *J* = 3.7 Hz), 161.2 (t, *J* = 2.3 Hz), 134.5, 130.4, 129.9, 128.8, 128.4, 128.3, 126.1, 121.6 (t, *J* = 274.8 Hz), 62.1, 52.0 (t, *J* = 27.9 Hz), 14.1. **^{19}F NMR** (471 MHz, $CDCl_3$): δ (ppm) -73.24 (dd, *J* = 152.6, 11.3 Hz, 1F), -73.70 (dd, *J* = 153.0, 11.7 Hz, 1F). **HRMS** (ESI) m/z: $[M+Na]^+$ Calcd for $C_{16}H_{14}F_2O_4SNa$ 363.0473; Found 363.0476.

2-(4-bromophenyl)-3-ethoxy-1,1-difluoro-3-oxopropyl benzoate (2q)

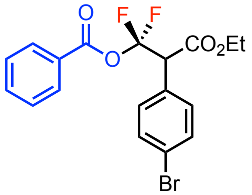

Following the general procedure (III), reaction was run using ethyl 2-(4-bromophenyl)-3,3-difluoroacrylate **1d** (58.2 mg, 0.2 mmol) and benzoic acid (2.0 mmol, 244.2 mg) in 1.0 mL MeCN at 75 °C for 48 h. The product was purified by flash column chromatography on silica gel (hexane : EA = 75 : 1) and obtained as a colorless oil (74.1 mg, 90% yield), R_f = 0.5 (hexane : EA = 4 : 1). **^{1}H NMR** (500 MHz, $CDCl_3$): δ (ppm) 7.98 (d, *J* = 7.5 Hz, 2H), 7.63 (t, *J* = 7.5 Hz, 1H), 7.52 - 7.41 (m, 6H), 4.80 (t, *J* = 11.5 Hz, 1H), 4.28 - 4.15 (m, 2H), 1.22 (t, *J* = 7.2 Hz, 3H). **^{13}C NMR** (126 MHz, $CDCl_3$): δ (ppm) 166.6 (d, *J* = 5.0 Hz), 161.2 (t, *J* = 2.3 Hz), 134.6, 132.1, 131.7, 130.4, 129.5, 128.9, 128.2, 123.5, 121.6 (t, *J* = 274.4 Hz), 62.2, 55.7 (t, *J* = 27.5 Hz), 14.1. **^{19}F NMR** (471 MHz, $CDCl_3$): δ (ppm) -72.82 (dd, *J* = 155.2, 11.3 Hz, 1F), -73.28 (dd, *J* = 154.3, 12.1 Hz, 1F). **HRMS** (ESI) m/z: $[M+Na]^+$ Calcd for $C_{18}H_{15}BrF_2O_4Na$ 435.0014; Found 435.0018.

3-ethoxy-1,1-difluoro-2-(4-methoxyphenyl)-3-oxopropyl benzoate (2r)

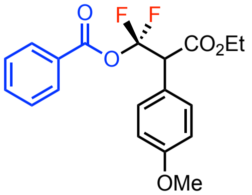

Following the general procedure (III), reaction was run using ethyl 3,3-difluoro-2-(4-methoxyphenyl)acrylate **1b** (48.4 mg, 0.2 mmol) and benzoic acid (2.0 mmol, 244.2 mg) in 1.0 mL MeCN at 75 °C for 48 h. The product was purified by flash column chromatography on silica gel (hexane : EA = 50 : 1) and obtained as a colorless oil (57.6 mg, 79% yield), R_f = 0.38 (hexane : EA = 4 : 1). **^{1}H NMR** (500 MHz, $CDCl_3$): δ (ppm) 7.98 (d, *J* = 8.1 Hz, 2H), 7.62 (t, *J* = 7.5 Hz, 1H), 7.46 (t, *J* = 7.0 Hz, 4H), 6.90 (d, *J* = 8.9 Hz, 2H), 4.75 (t, *J* = 11.5 Hz, 1H), 4.28 - 3.80 (m, 2H), 3.80 (s, 3H), 1.22 (t, *J* = 7.2 Hz, 3H). **^{13}C NMR** (126 MHz, $CDCl_3$): δ (ppm) 167.3 (t, *J* = 2.5 Hz), 161.3 (t, *J* = 2.3 Hz), 160.1, 134.4, 131.1, 130.4, 128.8, 128.4, 122.4 (t, *J* = 1.8 Hz), 121.9 (t, *J* = 274.5 Hz), 114.3, 61.9, 55.5 (t, *J* = 27.2 Hz), 55.4, 14.1. **^{19}F NMR** (471 MHz, $CDCl_3$): δ (ppm) -73.57 (d, *J* = 12.1 Hz, 2F). **HRMS** (ESI) m/z: $[M+Na]^+$ Calcd for $C_{19}H_{18}F_2O_5Na$ 387.1015; Found 387.1016.

3-ethoxy-1,1-difluoro-3-oxo-2-(4-(trifluoromethyl)phenyl)propyl benzoate (2s)

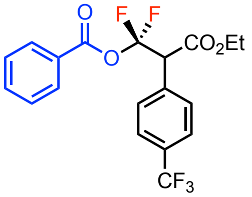

Following the general procedure (III), reaction was run using ethyl 3,3-difluoro-2-(4-(trifluoromethyl)phenyl)acrylate **1c** (56.0 mg, 0.2 mmol) and benzoic acid (2.0 mmol, 244.2 mg) in 1.0 mL MeCN at 75 °C for 48 h. The product was purified by flash column chromatography on silica gel (hexane : EA = 75 : 1) and obtained as a colorless oil (68.9 mg, 86% yield), R_f = 0.47 (hexane : EA = 4 : 1). **^{1}H NMR** (500 MHz, $CDCl_3$): δ (ppm) 7.98 (d, *J* = 8.2 Hz, 2H), 7.70 - 7.62 (m, 5H), 7.47 (t, *J* = 7.8 Hz, 2H), 4.93 (t, *J* = 11.5 Hz, 1H), 4.30 - 4.17 (m, 2H), 1.22 (t, *J* = 7.1 Hz, 3H). **^{13}C NMR** (126 MHz, $CDCl_3$): δ (ppm) 166.4 (d, *J* = 4.5 Hz), 161.2 (t, *J* = 2.3 Hz), 134.7, 134.4, 131.3 (q, *J* = 32.7 Hz), 130.5, 130.4, 128.9, 128.1, 125.8 (q, *J* = 3.9 Hz), 124.0 (q, *J* = 272.3 Hz), 121.6 (t, *J* = 274.5 Hz), 62.4, 56.0 (t, *J* = 27.7 Hz), 14.0. **^{19}F NMR** (471 MHz, $CDCl_3$): δ (ppm) -62.84 (s, 3F), -72.27 (dd, *J* = 154.8, 10.8 Hz, 1F), -73.02 (dd, *J* = 154.8, 11.7 Hz, 1F). **HRMS** (ESI) m/z: $[M+Na]^+$ Calcd for $C_{19}H_{15}F_5O_4Na$ 425.0783; Found 425.0785.

2-benzyl-3-ethoxy-1,1-difluoro-3-oxopropyl benzoate (2t)

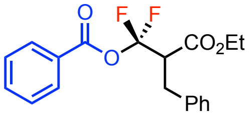

Following the general procedure (III), reaction was run using ethyl 2-benzyl-3,3-difluoroacrylate **1f** (45.2 mg, 0.2 mmol) and benzoic acid (2.0 mmol, 244.2 mg) in 1.0 mL MeCN at 75 °C for 48 h. The product was purified by flash column chromatography on silica gel (hexane : EA = 75 : 1) and obtained as a colorless oil (25.3 mg, 36% yield), R_f = 0.55 (hexane : EA = 4 : 1). **^{1}H NMR** (500 MHz, $CDCl_3$): δ (ppm) 8.02 (dd, *J* = 8.4, 1.4 Hz, 2H), 7.66 - 7.62 (m, 1H), 7.48 (t, *J* = 7.9 Hz, 2H), 7.30 - 7.20 (m, 5H), 4.07 (qd, *J* = 7.2, 2.2

Hz, 2H), 3.89 - 3.82 (m, 1H), 3.32 - 3.27 (m, 2H), 1.05 (t, *J* = 7.2 Hz, 3H). **^{13}C NMR** (126 MHz, $CDCl_3$): δ (ppm) 167.8 (d, *J* = 5.9 Hz), 161.0, 137.0, 134.5, 130.4, 129.0, 128.8, 128.8, 128.3, 127.1, 122.4 (dd, *J* = 276.6, 273.8 Hz), 61.7, 53.2 (t, *J* = 26.3 Hz), 32.2 (t, *J* = 2.7 Hz), 14.0. **^{19}F NMR** (471 MHz, $CDCl_3$): δ (ppm) -73.38 (dd, *J* = 152.6, 7.8 Hz, 1F), -75.46 (dd, *J* = 152.6, 13.0 Hz, 1F). **HRMS** (ESI) m/z: $[M+Na]^+$ Calcd for $C_{19}H_{18}F_2O_4Na$ 371.1065; Found 371.1068.

1,1-difluoro-3-(heptyloxy)-3-oxopropyl benzoate (4a)

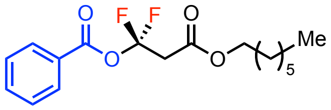

Following the general procedure (III), reaction was run using heptyl 3,3-difluoroacrylate **3a** (41.2 mg, 0.2 mmol) and benzoic acid (0.6 mmol, 73.3 mg) in 1.0 mL MeCN at 75 °C for 24 h. The product was purified by flash column chromatography on silica gel (hexane : EA = 100 : 1) and obtained as a colorless oil (56.8 mg, 87% yield), R_f = 0.52 (hexane : EA = 6 : 1). **^{1}H NMR** (500 MHz, $CDCl_3$): δ (ppm) 8.07 (dd, *J* = 8.4, 1.5 Hz, 2H), 7.64 (tt, *J* = 7.3, 1.4 Hz, 1H), 7.48 (t, *J* = 7.9 Hz, 2H), 4.11 (t, *J* = 6.6 Hz, 2H), 3.54 (t, *J* = 11.5 Hz, 2H), 1.59 - 1.53 (m, 2H), 1.30 - 1.17 (m, 8H), 0.86 (t, *J* = 7.1 Hz, 3H). **^{13}C NMR** (126 MHz, $CDCl_3$): δ (ppm) 165.4 (t, *J* = 5.9 Hz), 161.7 (t, *J* = 2.7 Hz), 134.5, 130.4, 128.8, 128.3, 121.6 (t, *J* = 266.6 Hz), 65.9, 40.7 (t, *J* = 30.2 Hz), 31.8, 28.9, 28.5, 25.8, 22.7, 14.2. **^{19}F NMR** (471 MHz, $CDCl_3$): δ (ppm) -66.56 (t, *J* = 11.3 Hz, 2F). **HRMS** (ESI) m/z: $[M+Na]^+$ Calcd for $C_{17}H_{22}F_2O_4Na$ 351.1378; Found 351.1380.

1,1-difluoro-3-(heptyloxy)-3-oxopropyl thiophene-3-carboxylate (4b)

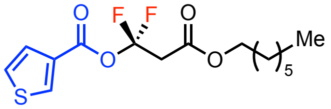

Following the general procedure (III), reaction was run using heptyl 3,3-difluoroacrylate **3a** (41.2 mg, 0.2 mmol) and thiophene-3-carboxylic acid (0.6 mmol, 76.9 mg) in 1.0 mL MeCN at 75 °C for 24 h. The product was purified by flash column chromatography on silica gel (hexane : EA = 100 : 1) and obtained as a colorless oil (56.2 mg, 84% yield), R_f = 0.48 (hexane : DCM = 6 : 1). **^{1}H NMR** (500 MHz, $CDCl_3$): δ (ppm) 8.25 (d, *J* = 3.5 Hz, 1H), 7.56 (d, *J* = 5.2 Hz, 1H), 7.36 (t, *J* = 4.6 Hz, 1H), 4.12 (t, *J* = 6.6 Hz, 2H), 3.51 (t, *J* = 11.5 Hz, 2H), 1.59 - 1.55 (m, 2H), 1.30 - 1.21 (m, 8H), 0.87 (t, *J* = 6.9 Hz, 3H). **^{13}C NMR** (126 MHz, $CDCl_3$): δ (ppm) 165.4 (t, *J* = 5.9 Hz), 157.2 (t, *J* = 3.0 Hz), 135.5, 131.5, 128.1, 126.9, 121.4 (t, *J* = 266.6 Hz), 65.9, 40.7 (t, *J* = 30.2 Hz), 31.8, 28.9, 28.5, 25.8, 22.7, 14.2. **^{19}F NMR** (471 MHz, $CDCl_3$): δ (ppm) -66.35 (t, *J* = 11.3 Hz, 2F). **HRMS** (ESI) m/z: $[M+Na]^+$ Calcd for $C_{15}H_{20}F_2O_4SNa$ 357.0943; Found 357.0944.

1,1-difluoro-3-(heptyloxy)-3-oxopropyl 1-methyl-*1H*-pyrrole-2-carboxylate (4c)

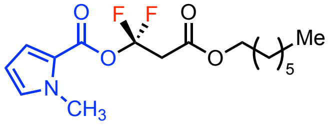

Following the general procedure (III), reaction was run using heptyl 3,3-difluoroacrylate **3a** (41.2 mg, 0.2 mmol) and 1-methyl-*1H*-pyrrole-2-carboxylic acid (0.6 mmol, 75.1 mg) in 1.0 mL MeCN at 75 °C for 24 h. The product was purified by flash column chromatography on silica gel (hexane : EA = 50 : 1) and obtained as a colorless oil (60.1 mg, 90% yield), R_f = 0.47 (hexane : EA = 4 : 1). **^{1}H NMR** (500 MHz, $CDCl_3$): δ (ppm) 7.06 - 7.05 (m, 1H), 6.88 (s, 1H), 6.15 (dt, *J* = 4.0, 1.7 Hz, 1H), 4.13 (t, *J* = 6.7 Hz, 2H), 3.91 (s, 3H), 3.46 (t, *J* = 11.4 Hz, 2H), 1.62 - 1.58 (m, 2H), 1.33 - 1.20 (m, 8H), 0.87 (t, *J* = 7.0 Hz, 3H). **^{13}C NMR** (126 MHz, $CDCl_3$): δ (ppm) 165.5 (t, *J* = 5.4 Hz), 155.5 (t, *J* = 2.7 Hz), 132.0, 121.3 (t, *J* = 266.3 Hz), 121.0, 120.3, 108.9, 65.8, 41.1 (t, *J* = 30.7 Hz), 37.0, 31.8, 29.0, 28.5, 25.8, 22.7, 14.2. **^{19}F NMR** (471 MHz, $CDCl_3$): δ (ppm) -66.01 (t, *J* = 11.3 Hz, 2F). **HRMS** (ESI) m/z: $[M+Na]^+$ Calcd for $C_{16}H_{23}F_2NO_4Na$ 354.1487; Found 354.1491.

1-benzyl 4-(1,1-difluoro-3-(heptyloxy)-3-oxopropyl) piperidine-1,4-dicarboxylate (4d)

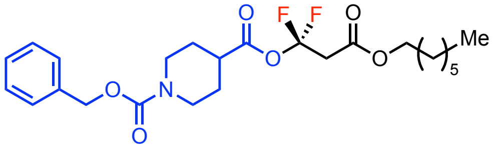

Following the general procedure (III), reaction was run using heptyl 3,3-difluoroacrylate **3a** (41.2 mg, 0.2 mmol) and 1-((benzyloxy)carbonyl)piperidine-4-carboxylic acid (0.6 mmol, 158.0 mg) in 1.0 mL MeCN at 75 °C for 24 h. The product was purified by flash column chromatography on silica gel (hexane : EA = 50 : 1) and obtained as a white solid (83.3 mg, 89% yield), R_f = 0.31 (hexane : EA = 4 : 1). **^{1}H NMR** (500 MHz, $CDCl_3$): δ (ppm) 7.38 - 7.30 (m, 5H), 5.13 (s, 2H), 4.12 (t, *J* = 6.7 Hz, 4H), 3.39 (t, *J*

= 11.4 Hz, 2H), 2.95 (t, *J* = 13.0 Hz, 2H), 2.59 (tt, *J* = 11.0, 4.0 Hz, 1H), 1.96 (d, *J* = 13.0 Hz, 2H), 1.74 - 1.60 (m, 4H), 1.34 - 1.25 (m, 8H), 0.88 (t, *J* = 6.9 Hz, 3H). **^{13}C NMR** (126 MHz, $CDCl_3$): δ (ppm) 169.5 (t, *J* = 2.5 Hz), 165.3 (t, *J* = 6.4 Hz), 155.2, 136.8, 128.6, 128.2, 128.1, 121.1 (t, *J* = 266.4 Hz), 67.4, 65.9, 43.0, 41.2, 40.0 (t, *J* = 30.4 Hz), 31.8, 29.0, 28.5, 27.4, 25.8, 22.7, 14.2. **^{19}F NMR** (471 MHz, $CDCl_3$): δ (ppm) -66.59 (t, *J* = 11.3 Hz, 2F). **HRMS** (ESI) m/z: $[M+Na]^+$ Calcd for $C_{24}H_{33}F_2NO_6Na$ 492.2168; Found 492.2172.

heptyl 3,3-difluoro-3-((2-(4-isobutylphenyl)propanoyl)oxy)propanoate (4e)

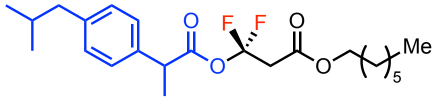

Following the general procedure (III), reaction was run using heptyl 3,3-difluoroacrylate **3a** (41.2 mg, 0.2 mmol) and ibuprofen (0.6 mmol, 123.8 mg) in 1.0 mL MeCN at 75 °C for 24 h. The product was purified by flash column chromatography on silica gel (hexane : EA = 100 : 1) and obtained as a white solid (72.6 mg, 88% yield), R_f = 0.55 (hexane : EA = 6 : 1). **^{1}H NMR** (500 MHz, $CDCl_3$): δ (ppm) 7.19 (d, *J* = 7.9 Hz, 2H), 7.11 (d, *J* = 7.8 Hz, 2H), 4.05 (t, *J* = 6.8 Hz, 2H), 3.76 (q, *J* = 7.1 Hz, 1H), 3.41 - 3.24 (m, 2H), 2.45 (d, *J* = 7.2 Hz, 2H), 1.85 (hept, *J* = 6.8 Hz, 1H), 1.59 - 1.52 (m, 5H), 1.32 - 1.25 (m, 8H), 0.90 - 0.87 (m, 9H). **^{13}C NMR** (126 MHz, $CDCl_3$): δ (ppm) 169.8 (t, *J* = 2.7 Hz), 165.2 (t, *J* = 5.4 Hz), 141.2, 136.0, 129.7, 127.4, 121.1 (t, *J* = 267.3 Hz), 65.8, 45.5, 45.2, 40.4 (t, *J* = 30.0 Hz), 31.8, 30.3, 29.0, 28.5, 25.8, 22.7, 22.5, 18.3, 14.2. **^{19}F NMR** (471 MHz, $CDCl_3$): δ (ppm) -67.20 (dt, *J* = 160.4, 11.3 Hz, 1F), -67.66 (dt, *J* = 159.5, 12.1 Hz, 1F). **HRMS** (ESI) m/z: $[M+Na]^+$ Calcd for $C_{23}H_{34}F_2O_4Na$ 435.2317; Found 435.2322.

heptyl 3,3-difluoro-3-(2-(11-oxo-6,11-dihydrodibenzo[*b*,*e*]oxepin-2-yl)acetoxy)propanoate (4f)

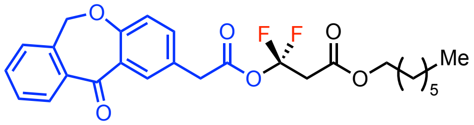

Following the general procedure (III), reaction was run using heptyl 3,3-difluoroacrylate **3a** (41.2 mg, 0.2 mmol) and isoxepac (0.6 mmol, 161.0 mg) in 1.0 mL MeCN at 75 °C for 24 h. The product was purified by flash column chromatography on silica gel (hexane : EA = 50 : 1) and obtained as a white solid (82.8 mg, 87% yield), R_f = 0.34 (hexane : EA = 4 : 1). **^{1}H NMR** (500 MHz, $CDCl_3$): δ (ppm) 8.12 (s, 1H), 7.89 (d, *J* = 7.8 Hz, 1H), 7.58 - 7.36 (m, 4H), 7.05 (d, *J* = 8.4 Hz, 1H), 5.19 (s, 2H), 4.12 (t, *J* = 6.7 Hz, 2H), 3.77 (s, 2H), 3.39 (t, *J* = 11.6 Hz, 2H), 1.64 - 1.60 (m, 2H), 1.34 - 1.27 (m, 8H), 0.87 (t, *J* = 6.6 Hz, 3H). **^{13}C NMR** (126 MHz, $CDCl_3$): δ (ppm) 190.8, 166.7, 165.3 (t, *J* = 5.7 Hz), 160.9, 140.5, 136.4, 135.6, 133.0, 133.0, 129.6, 129.4, 128.0, 125.9, 125.3, 121.5, 121.1 (t, *J* = 267.0 Hz), 73.8, 65.9, 40.1, 40.1 (t, *J* = 30.2 Hz), 31.8, 28.9, 28.5, 25.8, 22.7, 14.2. **^{19}F NMR** (471 MHz, $CDCl_3$): δ (ppm) -66.81 (t, *J* = 11.7 Hz, 2F). **HRMS** (ESI) m/z: $[M+Na]^+$ Calcd for $C_{26}H_{28}F_2O_6Na$ 497.1746; Found 497.1753.

1,1-difluoro-3-(heptyloxy)-3-oxopropyl (*R*)-4-((5*S*,8*R*,9*S*,10*S*,13*R*,14*S*,17*R*)-10,13-dimethyl-3,7,12-trioxohexadecahydro-1*H*-cyclopenta[*a*]phenanthren-17-yl)pentanoate (4g)

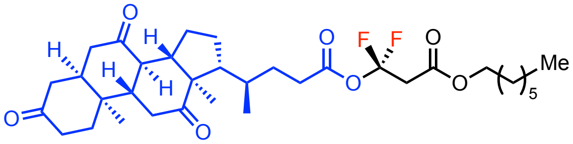

Following the general procedure (III), reaction was run using heptyl 3,3-difluoroacrylate **3a** (41.2 mg, 0.2 mmol) and dehydrocholic acid (0.6 mmol, 241.5 mg) in 1.0 mL MeCN at 75 °C for 24 h. The product was purified by flash column chromatography on silica gel (hexane : EA = 8 : 1) and obtained as a white solid (108.7 mg, 90% yield), R_f = 0.5 (hexane : EA = 1 : 2). **^{1}H NMR** (500 MHz, $CDCl_3$): δ (ppm) 4.11 (t, *J* = 6.7 Hz, 2H), 3.36 (t, *J* = 11.6 Hz, 2H), 2.92 - 2.80 (m, 3H), 2.53 - 1.80 (m, 17H), 1.64 - 1.60 (m, 2H), 1.44 - 1.22 (m, 15H), 1.06 (s, 3H), 0.88 - 0.83 (m, 6H). **^{13}C NMR** (126 MHz, $CDCl_3$): δ (ppm) 212.0, 209.2, 208.8, 169.1 (t, *J* = 2.3 Hz), 165.3 (t, *J* = 5.7 Hz), 120.9 (t, *J* = 266.1 Hz), 65.8, 57.0, 51.8, 49.1, 46.9, 45.6, 45.6, 45.1, 42.9, 40.3 (t, *J* = 30.4 Hz), 38.7, 36.6, 36.1, 35.4, 35.3, 31.8, 31.7, 29.7, 28.9, 28.5, 27.7, 25.8, 25.2, 22.6, 22.0, 18.6, 14.2, 11.9. **^{19}F NMR** (471 MHz, $CDCl_3$): δ (ppm) -66.87 (td, *J* = 12.1, 11.7, 6.1 Hz, 2F). **HRMS** (ESI) m/z: $[M+Na]^+$ Calcd for $C_{34}H_{50}F_2O_7Na$ 631.3417; Found 631.3427.

heptyl 3,3-difluoro-3-(tosyloxy)propanoate (5a)

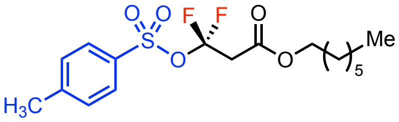

Following the general procedure (III), reaction was run using heptyl 3,3-difluoroacrylate **3a** (41.2 mg, 0.2 mmol) and 4-methylbenzenesulfonic acid (0.6 mmol, 103.3 mg) in 1.0 mL MeCN at 75 °C for 24 h. The product was purified by flash column

chromatography on silica gel (hexane : EA = 15 : 1) and obtained as a white solid (46.5 mg, 62% yield), R_f = 0.6 (hexane : EA = 3 : 1). **^{1}H NMR** (500 MHz, $CDCl_3$): δ (ppm) 7.87 (d, J = 8.1 Hz, 2H), 7.37 (d, J = 8.2 Hz, 2H), 4.08 (t, J = 6.8 Hz, 2H), 3.19 (t, J = 11.5 Hz, 2H), 2.47 (s, 3H), 1.60 - 1.55 (m, 2H), 1.31 - 1.25 (m, 8H), 0.88 (t, J = 6.9 Hz, 3H). **^{13}C NMR** (126 MHz, $CDCl_3$): δ (ppm) 164.4 (t, J = 4.1 Hz), 146.2, 134.0, 130.1, 128.4, 121.5 (t, J = 276.1 Hz), 66.0, 41.8 (t, J = 28.8 Hz), 31.8, 29.0, 28.4, 25.8, 22.7, 21.9, 14.2. **^{19}F NMR** (471 MHz, $CDCl_3$): δ (ppm) -64.94 (t, J = 11.7 Hz, 2F). **HRMS** (ESI) m/z: $[M+Na]^+$ Calcd for $C_{17}H_{24}F_2O_5SNa$ 401.1205; Found 401.1207.

heptyl 3-(((((1*R*,4*S*)-7,7-dimethyl-2-oxobicyclo[2.2.1]heptan-1-yl)methyl)sulfonyl)oxy)-3,3-difluoropropanoate (5b)

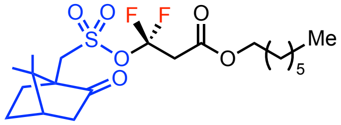

Following the general procedure (III), reaction was run using heptyl 3,3-difluoroacrylate **3a** (41.2 mg, 0.2 mmol) and Camphor-10-sulfonic acid (0.6 mmol, 139.4 mg) in 1.0 mL MeCN at 75 °C for 24 h. The product was purified by flash column chromatography on silica gel (hexane : EA = 15 : 1) and obtained as a white solid (47.4 mg, 54% yield), R_f = 0.5 (hexane : EA = 3 : 1). **^{1}H NMR** (500 MHz, $CDCl_3$): δ (ppm) 4.16 (t, J = 6.8 Hz, 2H), 3.87 (d, J = 15.0 Hz, 1H), 3.33 - 3.25 (m, 3H), 2.45 - 2.38 (m, 2H), 2.15 - 2.05 (m, 2H), 1.97 (d, J = 18.5 Hz, 1H), 1.75 - 1.61 (m, 3H), 1.49 - 1.44 (m, 1H), 1.33 - 1.27 (m, 8H), 1.13 (s, 3H), 0.91 - 0.86 (m, 6H). **^{13}C NMR** (126 MHz, $CDCl_3$): δ (ppm) 213.6, 164.5 (t, J = 4.5 Hz), 121.6 (t, J = 275.9 Hz), 66.1, 58.4, 51.8, 48.1, 43.0, 42.5, 41.5 (t, J = 29.1 Hz), 31.8, 29.0, 28.5, 27.0, 25.8, 25.3, 22.7, 19.9, 19.8, 14.2. **^{19}F NMR** (471 MHz, $CDCl_3$): δ (ppm) -63.98 (dt, J = 149.1, 11.3 Hz, 1F), -64.89 (dt, J = 148.3, 11.3 Hz, 1F). **HRMS** (ESI) m/z: $[M+Na]^+$ Calcd for $C_{20}H_{32}F_2O_6SNa$ 461.1780; Found 461.1785.

Spectra.

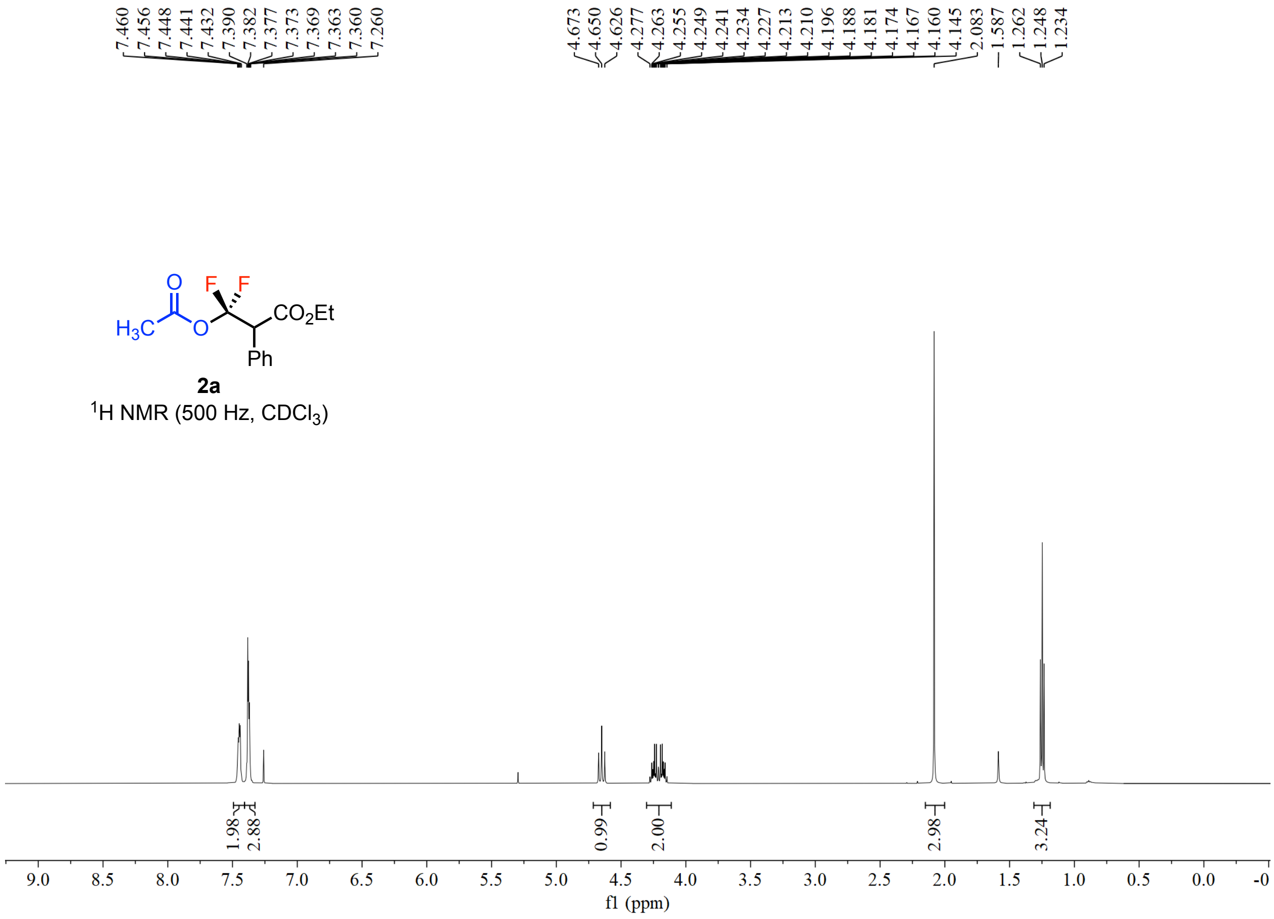

7.460
7.456
7.448
7.441
7.432
7.390
7.382
7.377
7.373
7.369
7.363
7.360
7.260
4.673
4.650
4.626
4.277
4.263
4.255
4.249
4.241
4.234
4.227
4.213
4.210
4.196
4.188
4.181
4.174
4.167
4.160
4.145
2.083
1.587
1.262
1.248
1.234
O
F
F
H3C
O
CO2Et
Ph
2a
1H NMR (500 Hz, CDCl3)
1.98
2.88
0.99
2.00
2.98
3.24
9.0
8.5
8.0
7.5
7.0
6.5
6.0
5.5
5.0
4.5
4.0
3.5
3.0
2.5
2.0
1.5
1.0
0.5
0.0
-0
f1 (ppm)

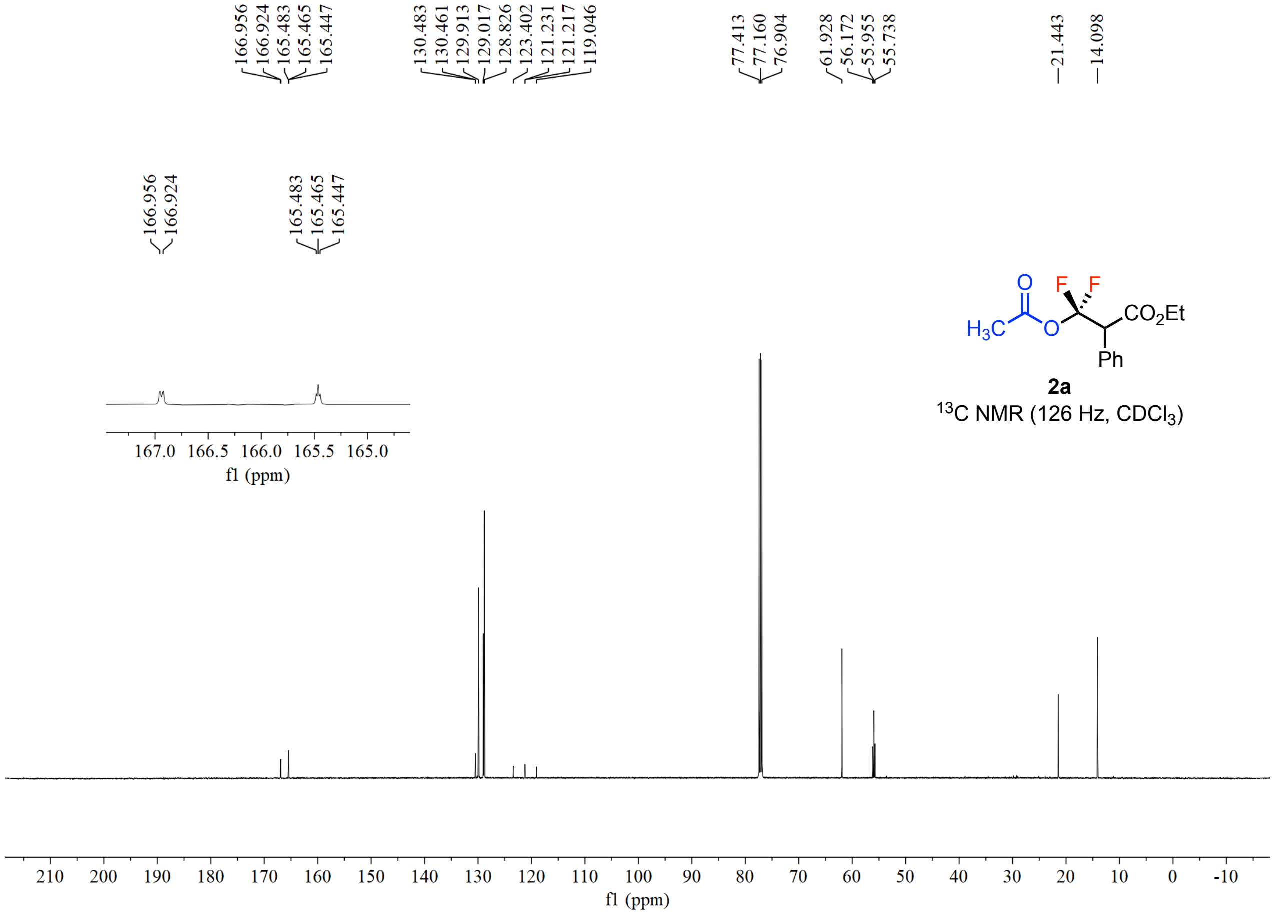

166.956
166.924
165.483
165.465
165.447
130.483
130.461
129.913
129.017
128.826
123.402
121.231
121.217
119.046
77.413
77.160
76.904
61.928
56.172
55.955
55.738
21.443
14.098
166.956
166.924
165.483
165.465
165.447
167.0
166.5
166.0
165.5
165.0
f1 (ppm)
O
F
F
H3C
O
CO2Et
Ph
2a
13C NMR (126 Hz, CDCl3)
210
200
190
180
170
160
150
140
130
120
110
100
90
80
70
60
50
40
30
20
10
0
-10
f1 (ppm)

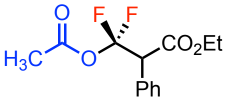

2a

^{19}F NMR (471 Hz, $CDCl_3$)

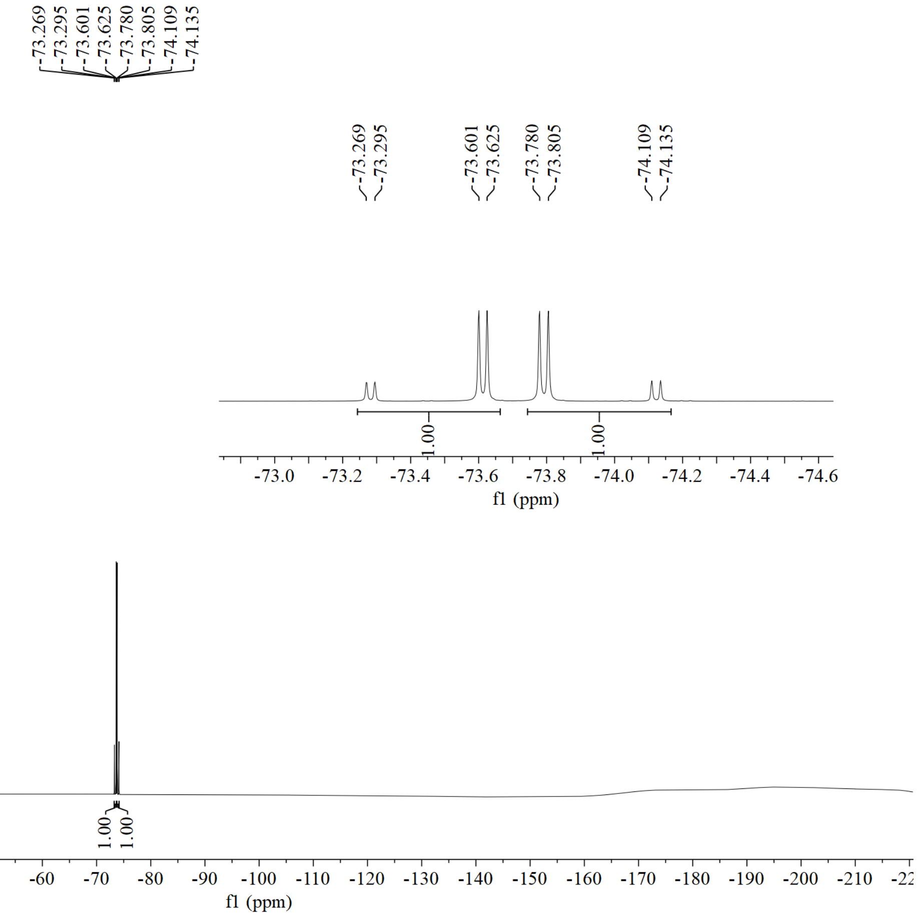

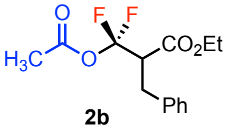

2b

^{1}H NMR (500 Hz, $CDCl_3$)

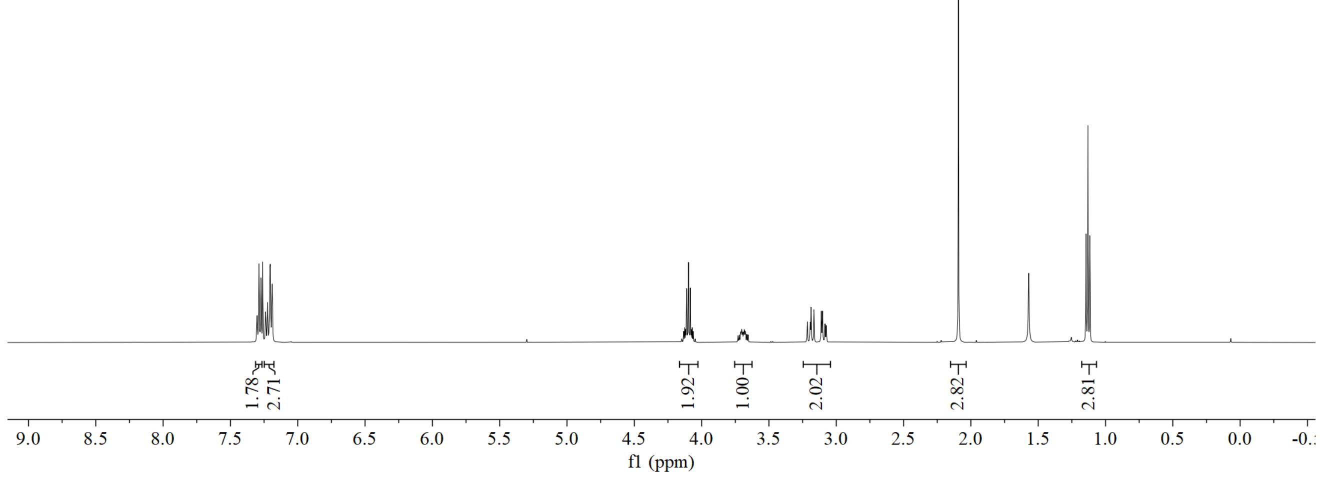

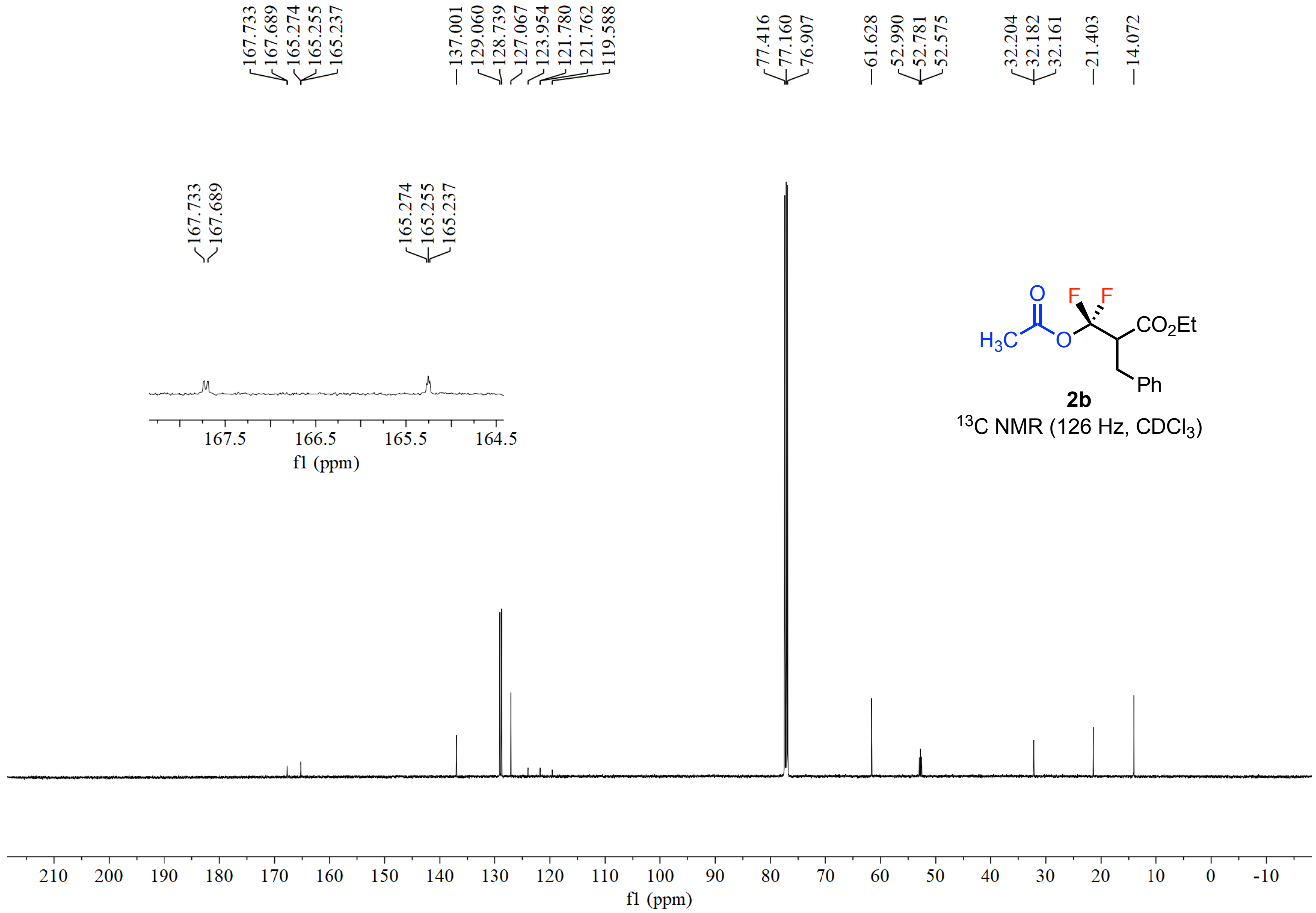

167.733
167.689
165.274
165.255
165.237
137.001
129.060
128.739
127.067
123.954
121.780
121.762
119.588
77.416
77.160
76.907
61.628
52.990
52.781
52.575
32.204
32.182
32.161
21.403
14.072
167.5
166.5
165.5
164.5
f1 (ppm)
H3C
O
O
F
F
CO2Et
Ph
2b
13C NMR (126 Hz, CDCl3)
210 200 190 180 170 160 150 140 130 120 110 100 90 80 70 60 50 40 30 20 10 0 -10
f1 (ppm)

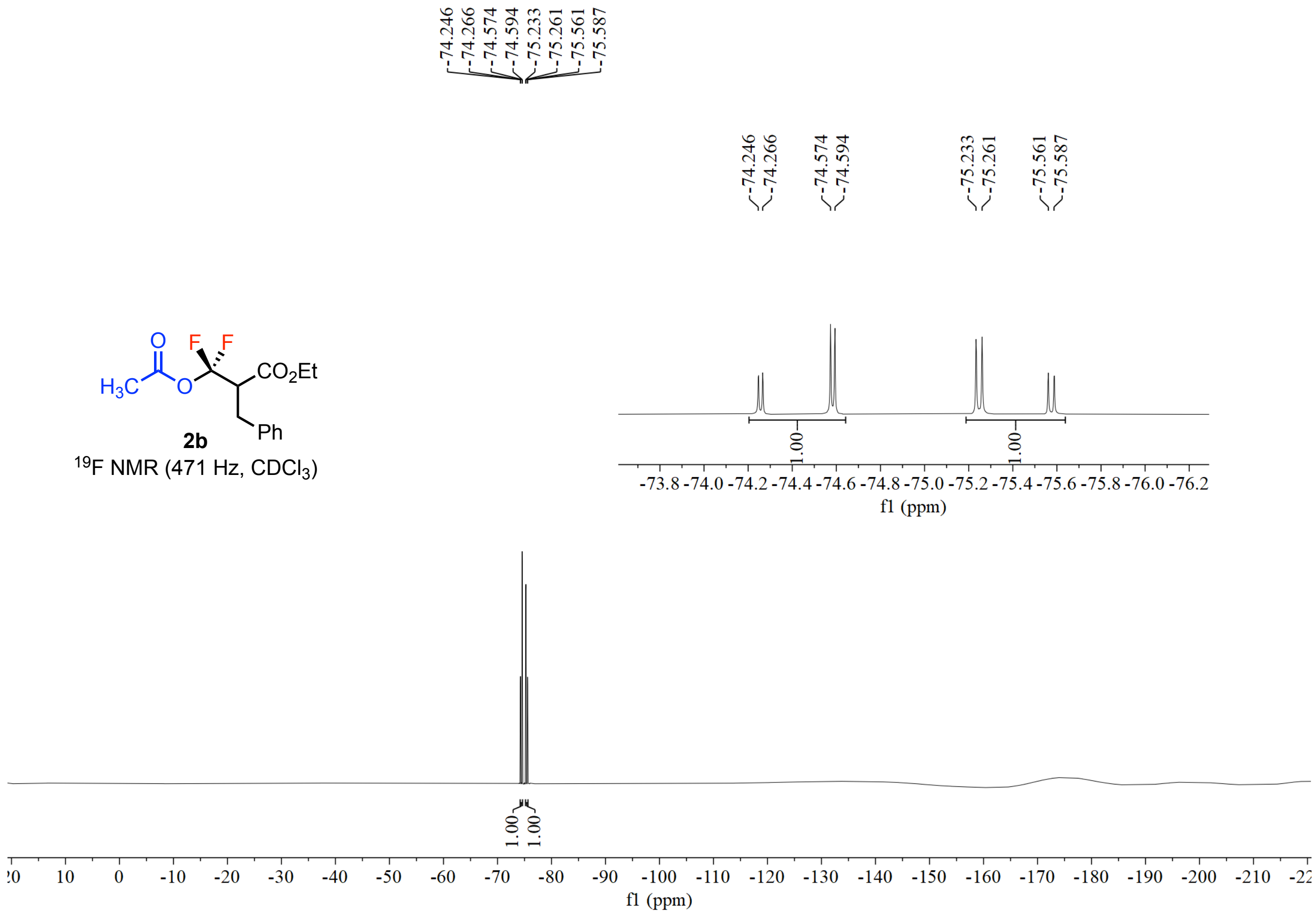

-74.246
-74.266
-74.574
-74.594
-75.233
-75.261
-75.561
-75.587
H3C
O
O
F
F
CO2Et
Ph
2b
19F NMR (471 Hz, CDCl3)
1.00
1.00
-73.8 -74.0 -74.2 -74.4 -74.6 -74.8 -75.0 -75.2 -75.4 -75.6 -75.8 -76.0 -76.2
f1 (ppm)
10 0 -10 -20 -30 -40 -50 -60 -70 -80 -90 -100 -110 -120 -130 -140 -150 -160 -170 -180 -190 -200 -210
f1 (ppm)

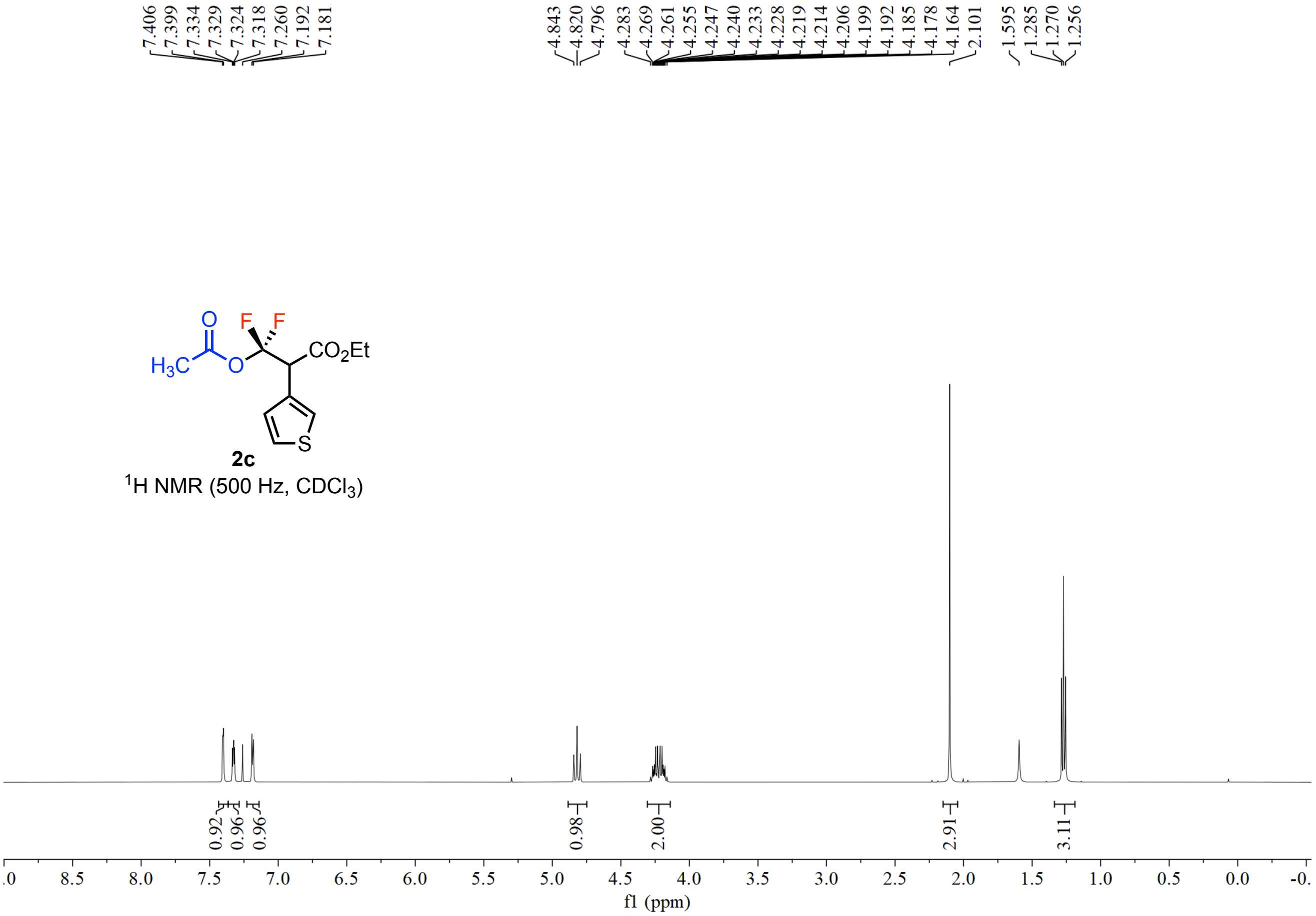

7.406
7.399
7.334
7.329
7.324
7.318
7.260
7.192
7.181
4.843
4.820
4.796
4.283
4.269
4.261
4.255
4.247
4.240
4.233
4.228
4.219
4.214
4.206
4.199
4.192
4.185
4.178
4.164
2.101
1.595
1.285
1.270
1.256
2c
^{1}H NMR (500 Hz, CDCl$_3$)
0.92
0.96
0.96
0.98
2.00
2.91
3.11
f1 (ppm)

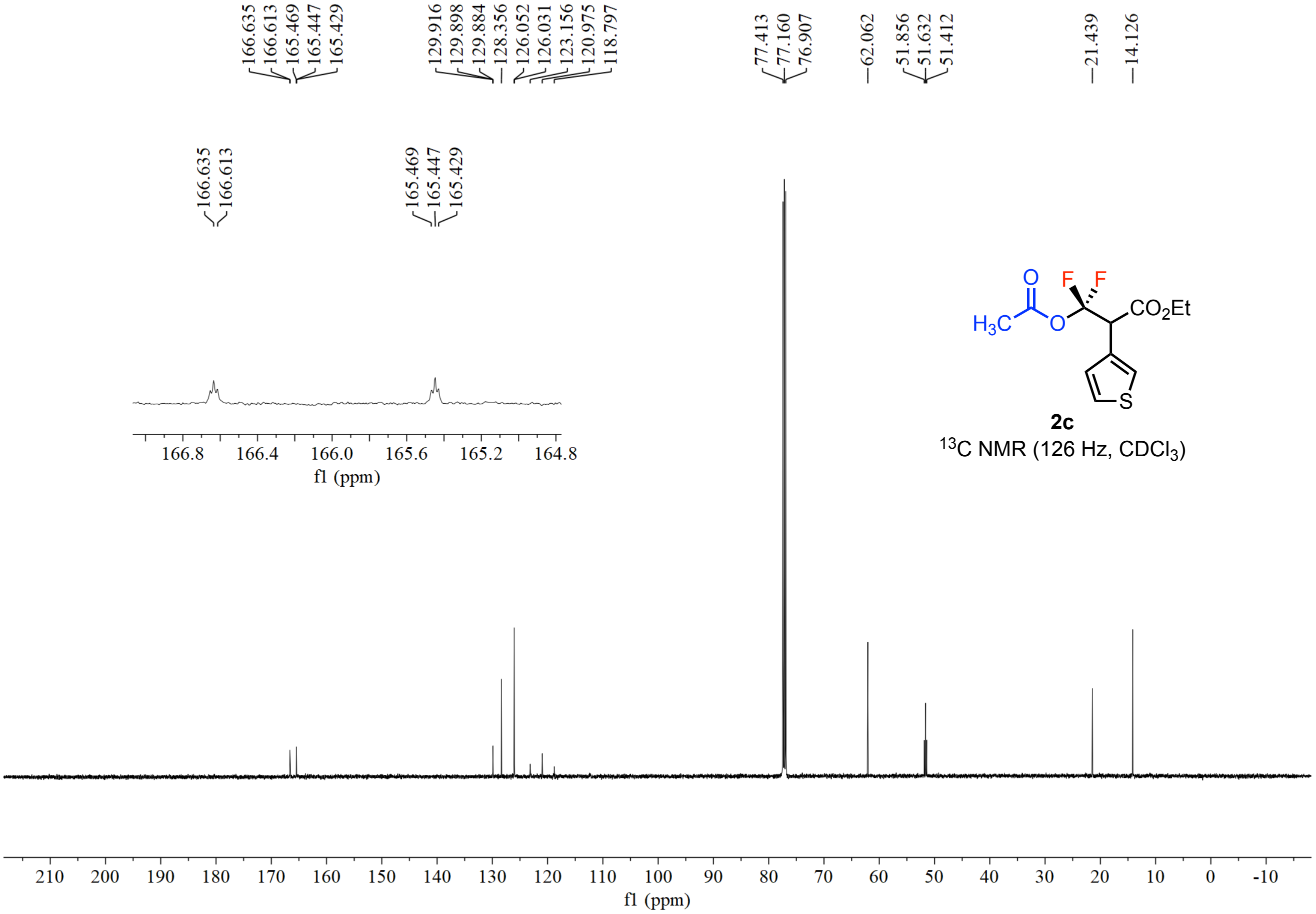

166.635
166.613
165.469
165.447
165.429
129.916
129.898
129.884
128.356
126.052
126.031
123.156
120.975
118.797
77.413
77.160
76.907
62.062
51.856
51.632
51.412
21.439
14.126
2c
^{13}C NMR (126 Hz, CDCl$_3$)
f1 (ppm)

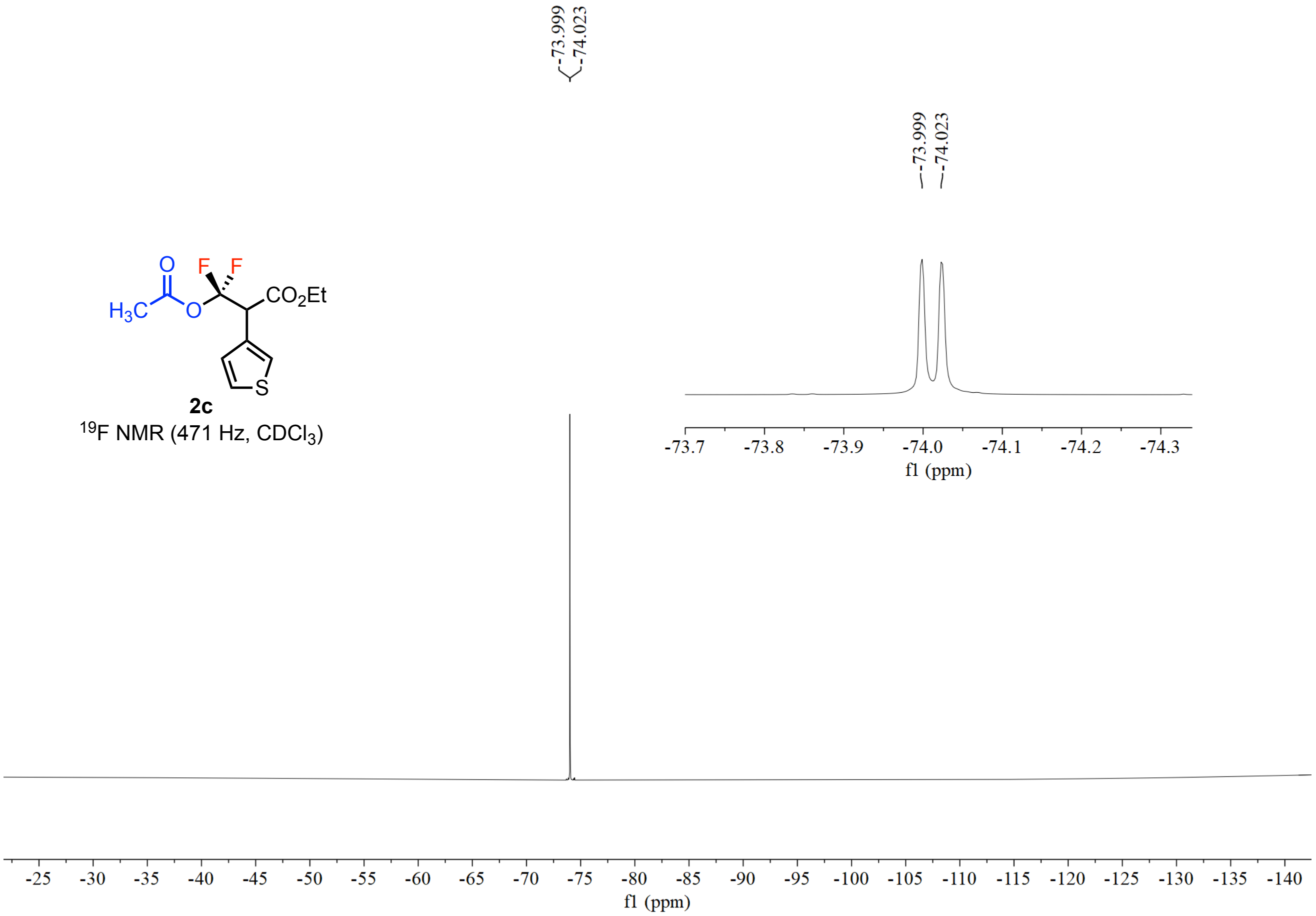
-73.999
-74.023
CO2Et
2c
19F NMR (471 Hz, CDCl3)
f1 (ppm)

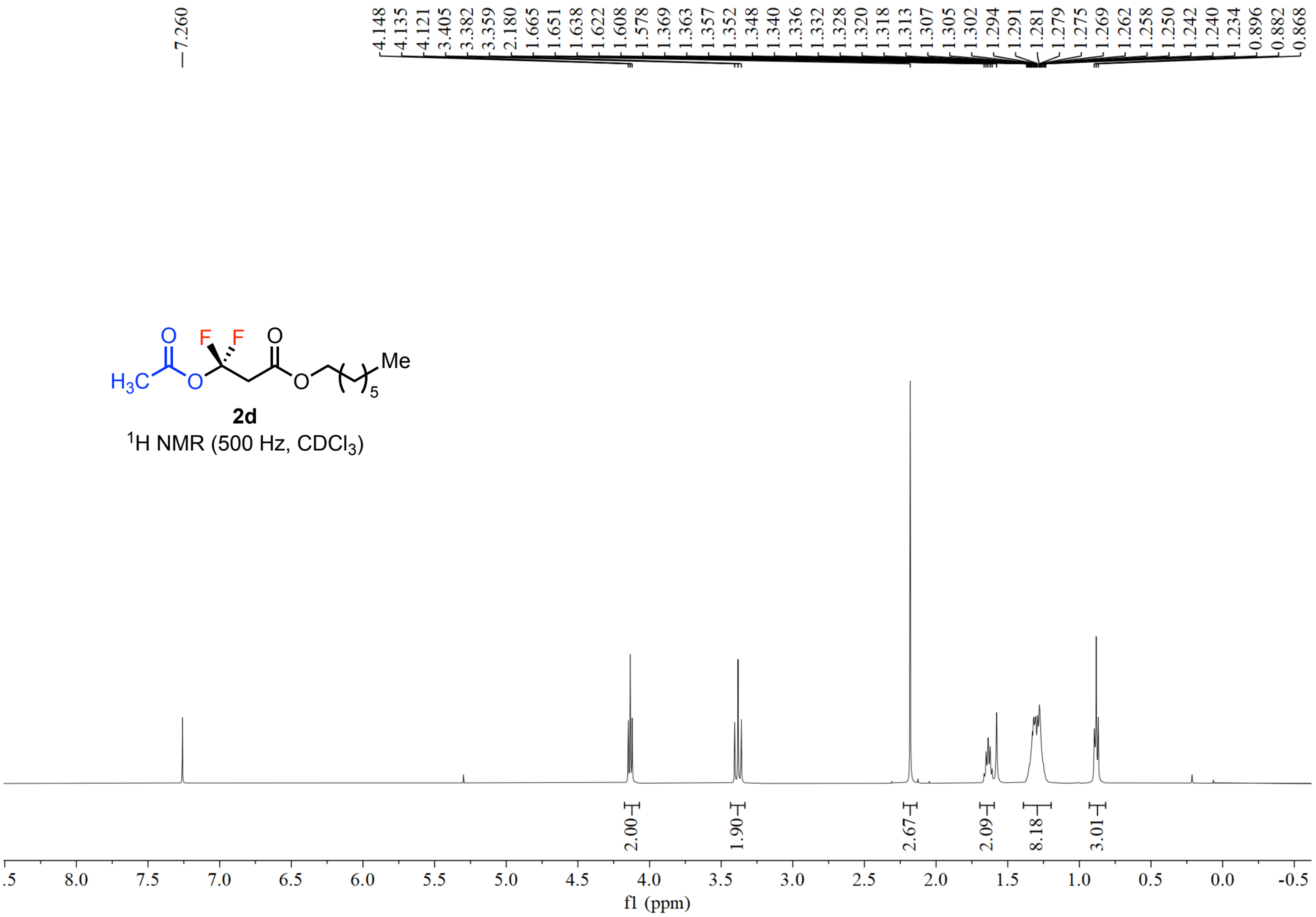
7.260
4.148 4.135 4.121 3.405 3.382 3.359 2.180 1.665 1.651 1.638 1.622 1.608 1.578 1.369 1.363 1.357 1.352 1.348 1.340 1.336 1.332 1.328 1.320 1.318 1.313 1.307 1.305 1.302 1.294 1.291 1.281 1.279 1.275 1.269 1.262 1.258 1.250 1.242 1.240 1.234 0.896 0.882 0.868
Me
2d
1H NMR (500 Hz, CDCl3)
2.00 1.90 2.67 2.09 8.18 3.01
f1 (ppm)

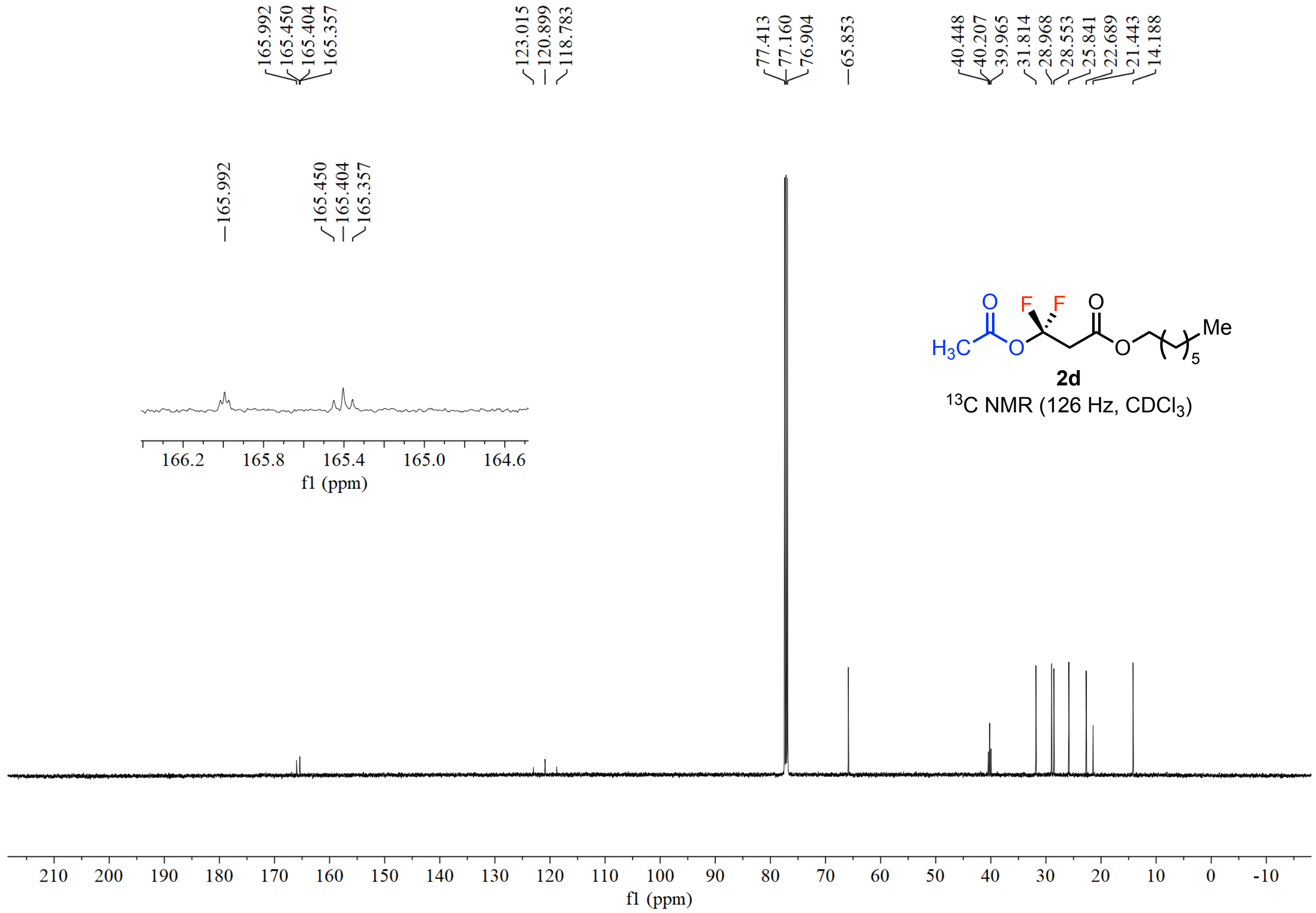

165.992
165.450
165.404
165.357
123.015
120.899
118.783
77.413
77.160
76.904
65.853
40.448
40.207
39.965
31.814
28.968
28.553
25.841
22.689
21.443
14.188
166.2 165.8 165.4 165.0 164.6
f1 (ppm)
O F F O
H3C O O Me
5
2d
13C NMR (126 Hz, CDCl3)
210 200 190 180 170 160 150 140 130 120 110 100 90 80 70 60 50 40 30 20 10 0 -10
f1 (ppm)

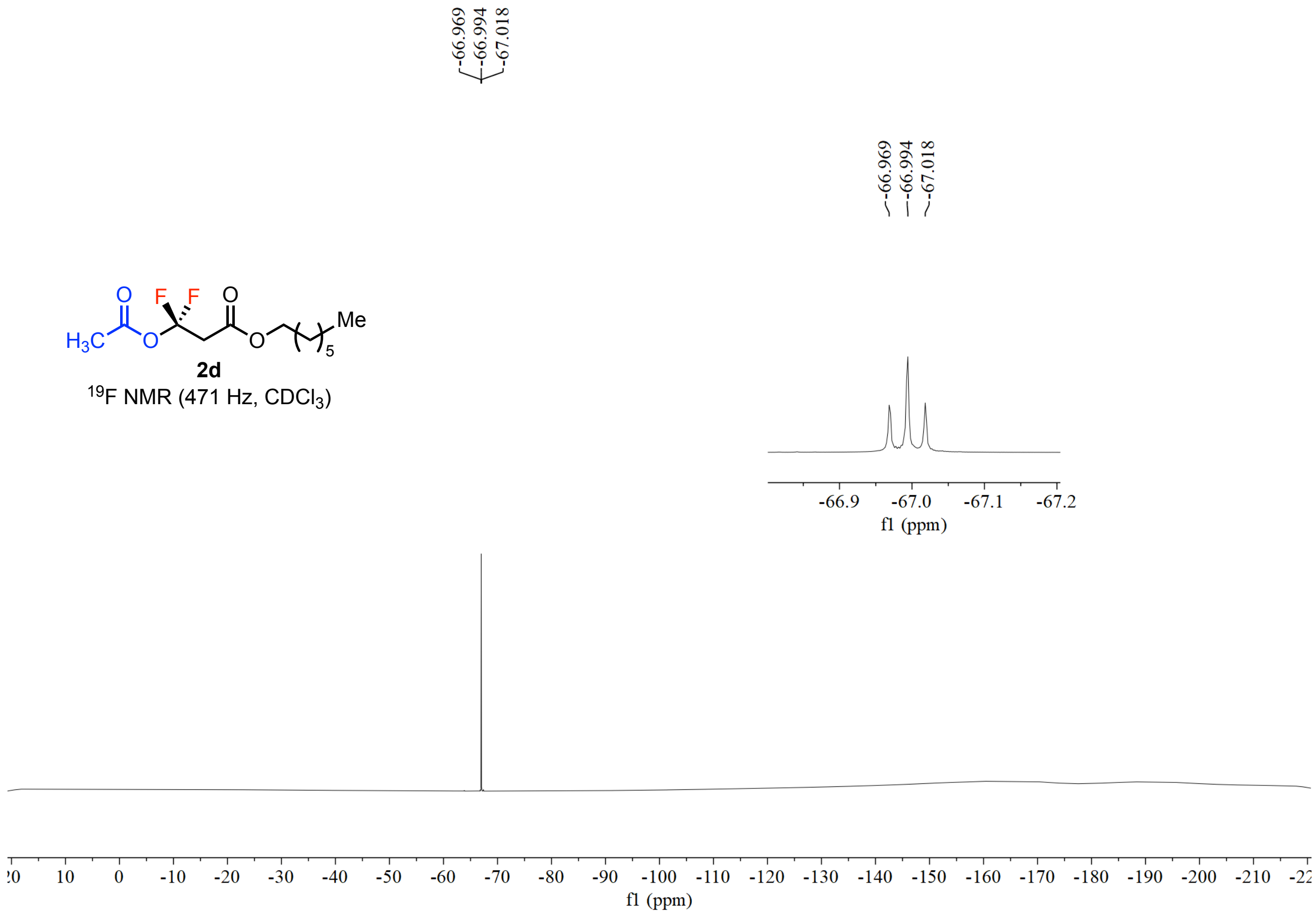

-66.969
-66.994
-67.018
O F F O
H3C O O Me
5
2d
19F NMR (471 Hz, CDCl3)
-66.9 -67.0 -67.1 -67.2
f1 (ppm)
10 0 -10 -20 -30 -40 -50 -60 -70 -80 -90 -100 -110 -120 -130 -140 -150 -160 -170 -180 -190 -200 -210
f1 (ppm)

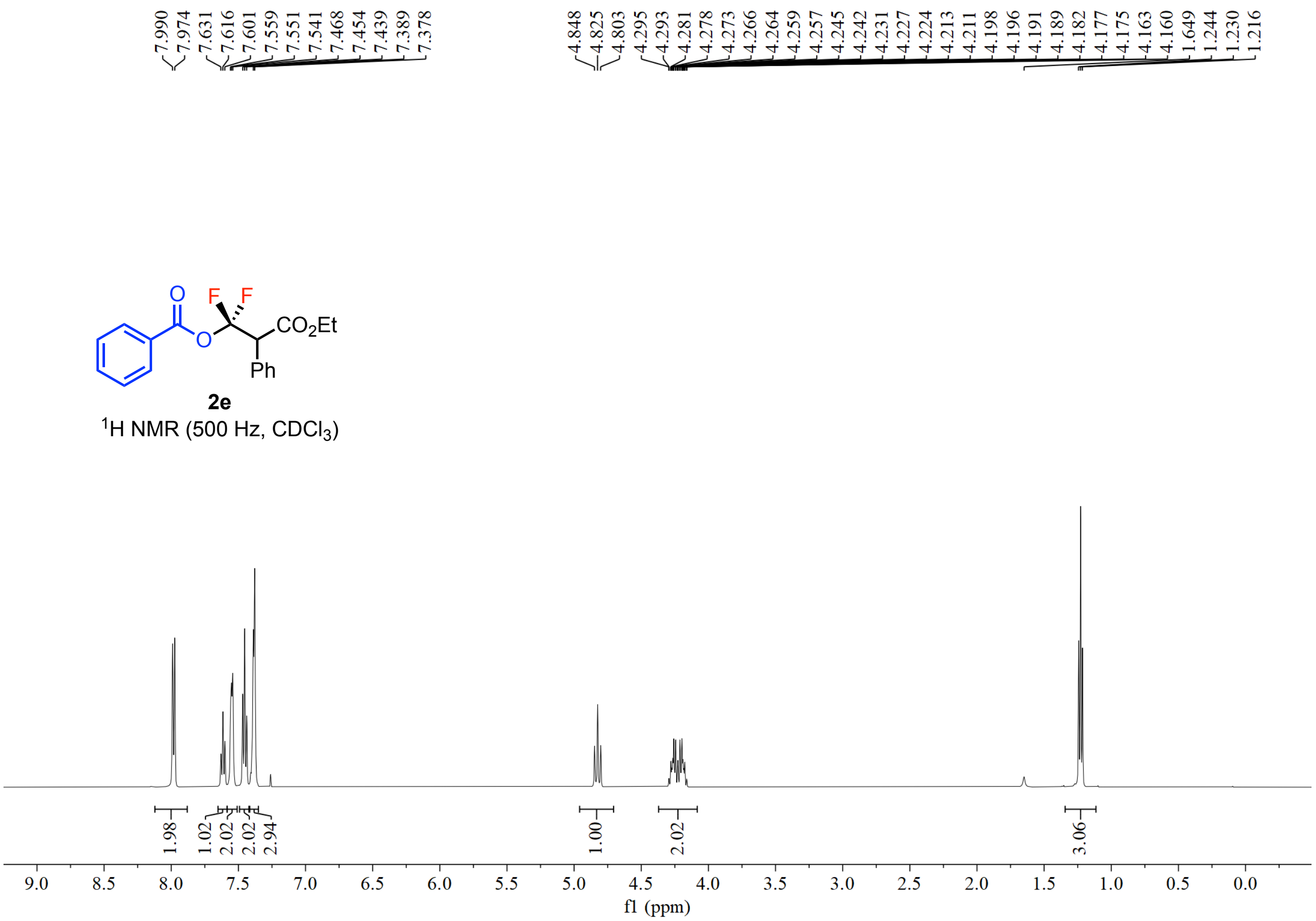
7.990
7.974
7.631
7.616
7.601
7.559
7.551
7.541
7.468
7.454
7.439
7.389
7.378
4.848
4.825
4.803
4.295
4.293
4.281
4.278
4.273
4.266
4.264
4.259
4.257
4.245
4.242
4.231
4.227
4.224
4.213
4.211
4.198
4.196
4.191
4.189
4.182
4.177
4.175
4.163
4.160
1.649
1.244
1.230
1.216
O
F
F
CO_2Et
O
Ph
2e
^1H NMR (500 Hz, $CDCl_3$)
1.98
1.02
2.02
2.02
2.94
1.00
2.02
3.06
9.0
8.5
8.0
7.5
7.0
6.5
6.0
5.5
5.0
4.5
4.0
3.5
3.0
2.5
2.0
1.5
1.0
0.5
0.0
f1 (ppm)

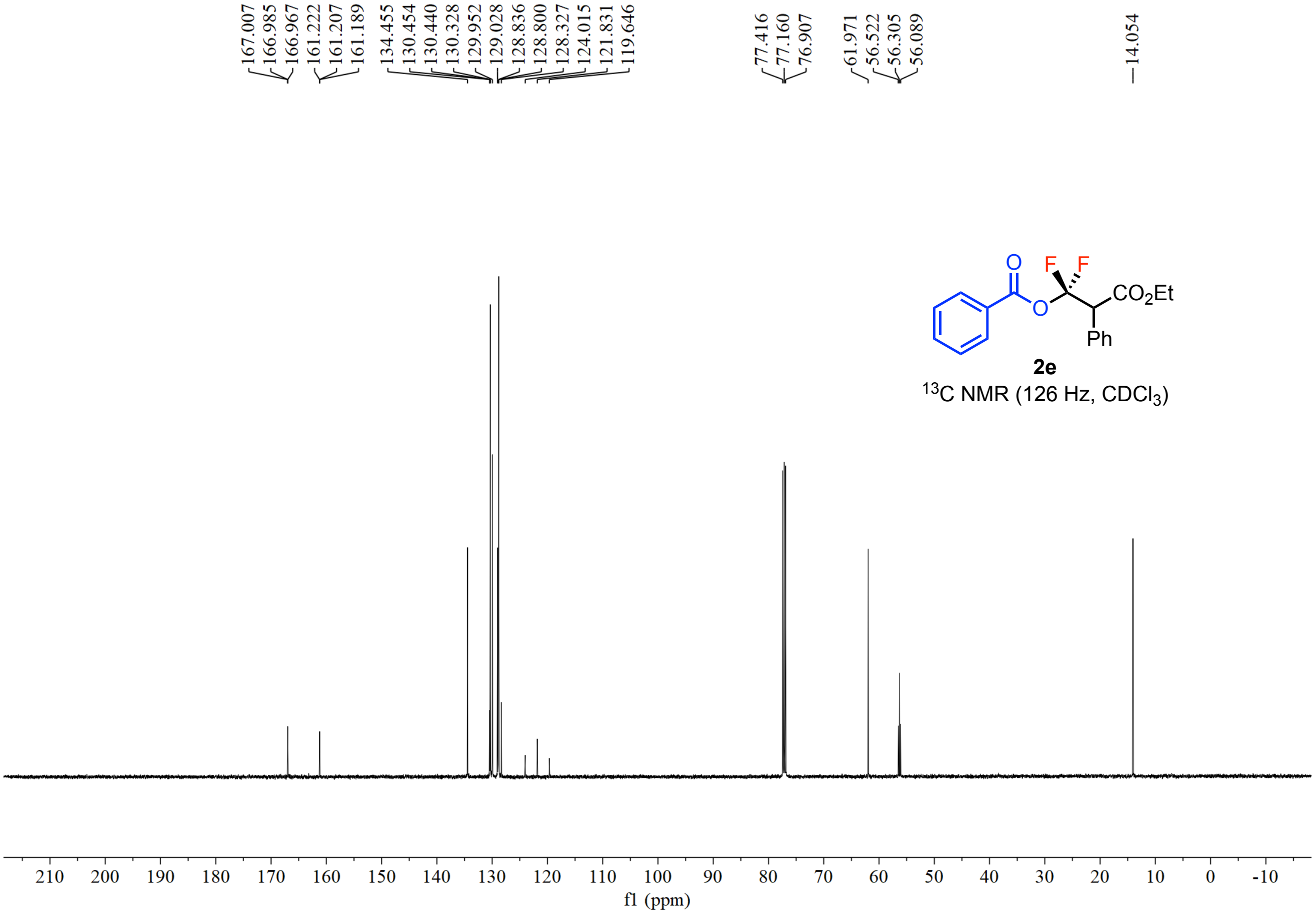
167.007
166.985
166.967
161.222
161.207
161.189
134.455
130.454
130.440
130.328
129.952
129.028
128.836
128.800
128.327
124.015
121.831
119.646
77.416
77.160
76.907
61.971
56.522
56.305
56.089
14.054
O
F
F
CO_2Et
O
Ph
2e
^{13}C NMR (126 Hz, $CDCl_3$)
210
200
190
180
170
160
150
140
130
120
110
100
90
80
70
60
50
40
30
20
10
0
-10
f1 (ppm)

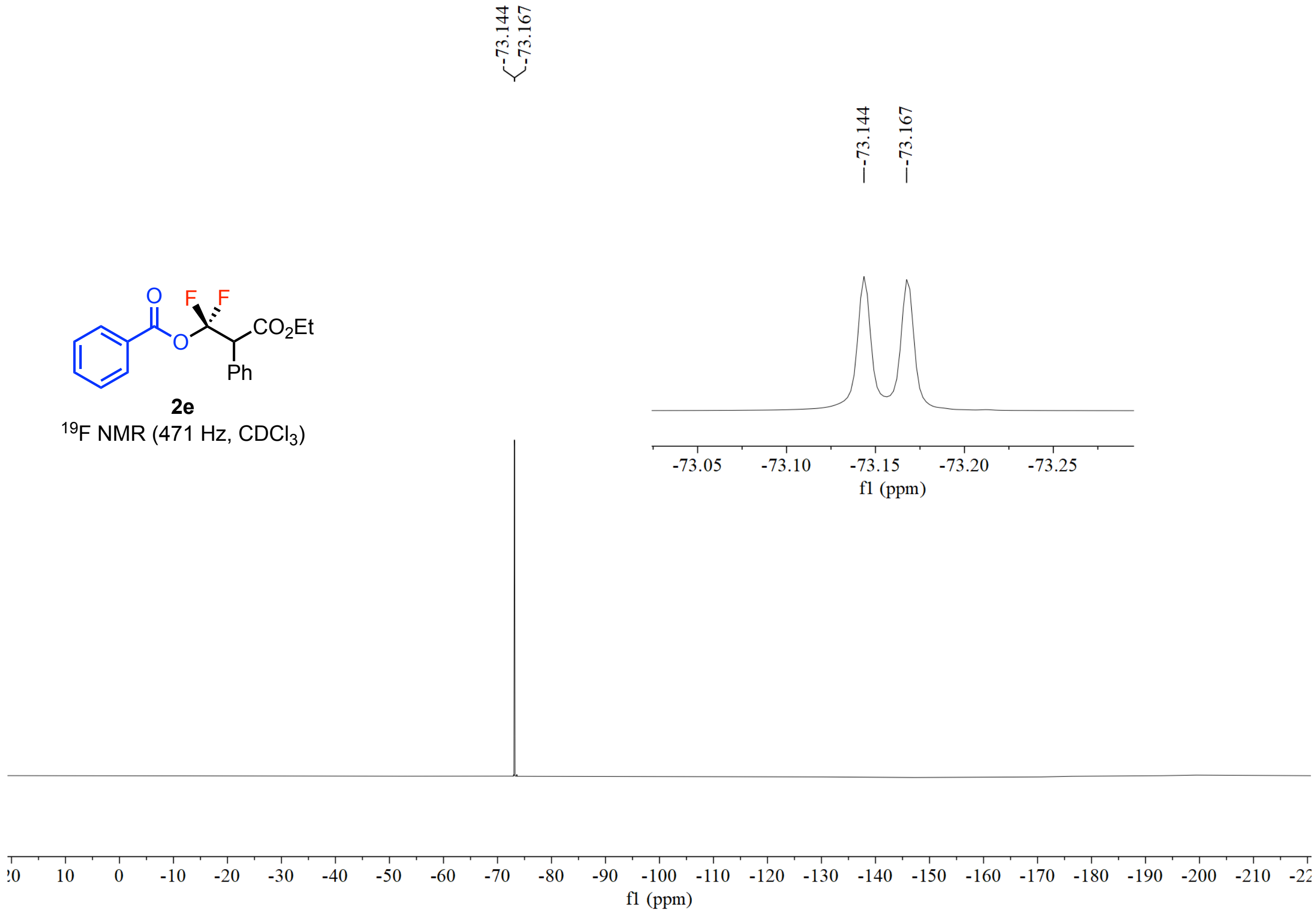
-73.144
-73.167
2e
^{19}F NMR (471 Hz, CDCl$_3$)
-73.05 -73.10 -73.15 -73.20 -73.25
f1 (ppm)
20 10 0 -10 -20 -30 -40 -50 -60 -70 -80 -90 -100 -110 -120 -130 -140 -150 -160 -170 -180 -190 -200 -210 -220
f1 (ppm)

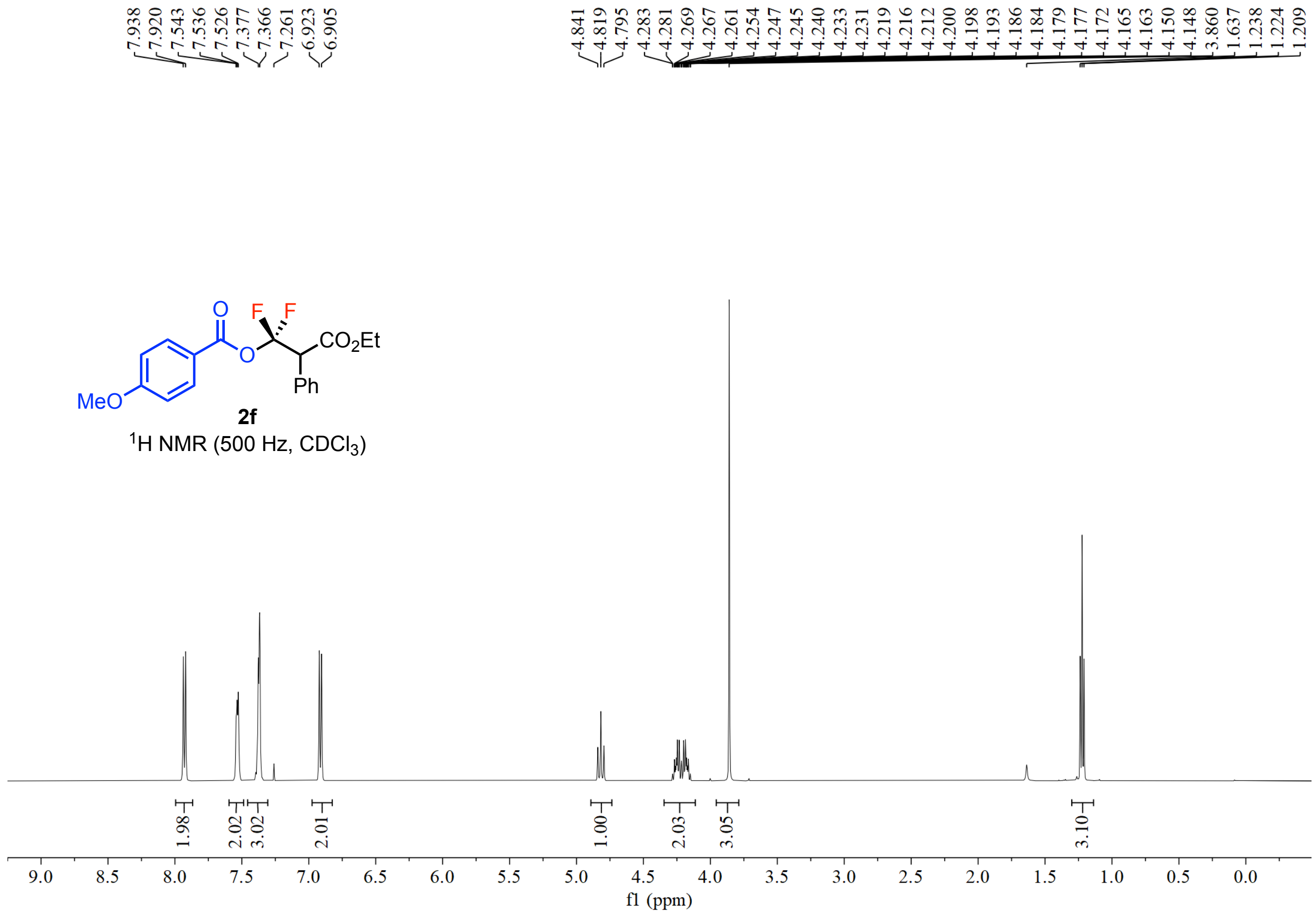
7.938 7.920 7.543 7.536 7.526 7.377 7.366 7.261 6.923 6.905
4.841 4.819 4.795 4.283 4.281 4.269 4.267 4.261 4.254 4.247 4.245 4.240 4.233 4.231 4.219 4.216 4.212 4.200 4.198 4.193 4.186 4.184 4.179 4.177 4.172 4.165 4.163 4.150 4.148 3.860 1.637 1.238 1.224 1.209
MeO
2f
^{1}H NMR (500 Hz, CDCl$_3$)
1.98 2.02 3.02 2.01 1.00 2.03 3.05 3.10
9.0 8.5 8.0 7.5 7.0 6.5 6.0 5.5 5.0 4.5 4.0 3.5 3.0 2.5 2.0 1.5 1.0 0.5 0.0
f1 (ppm)

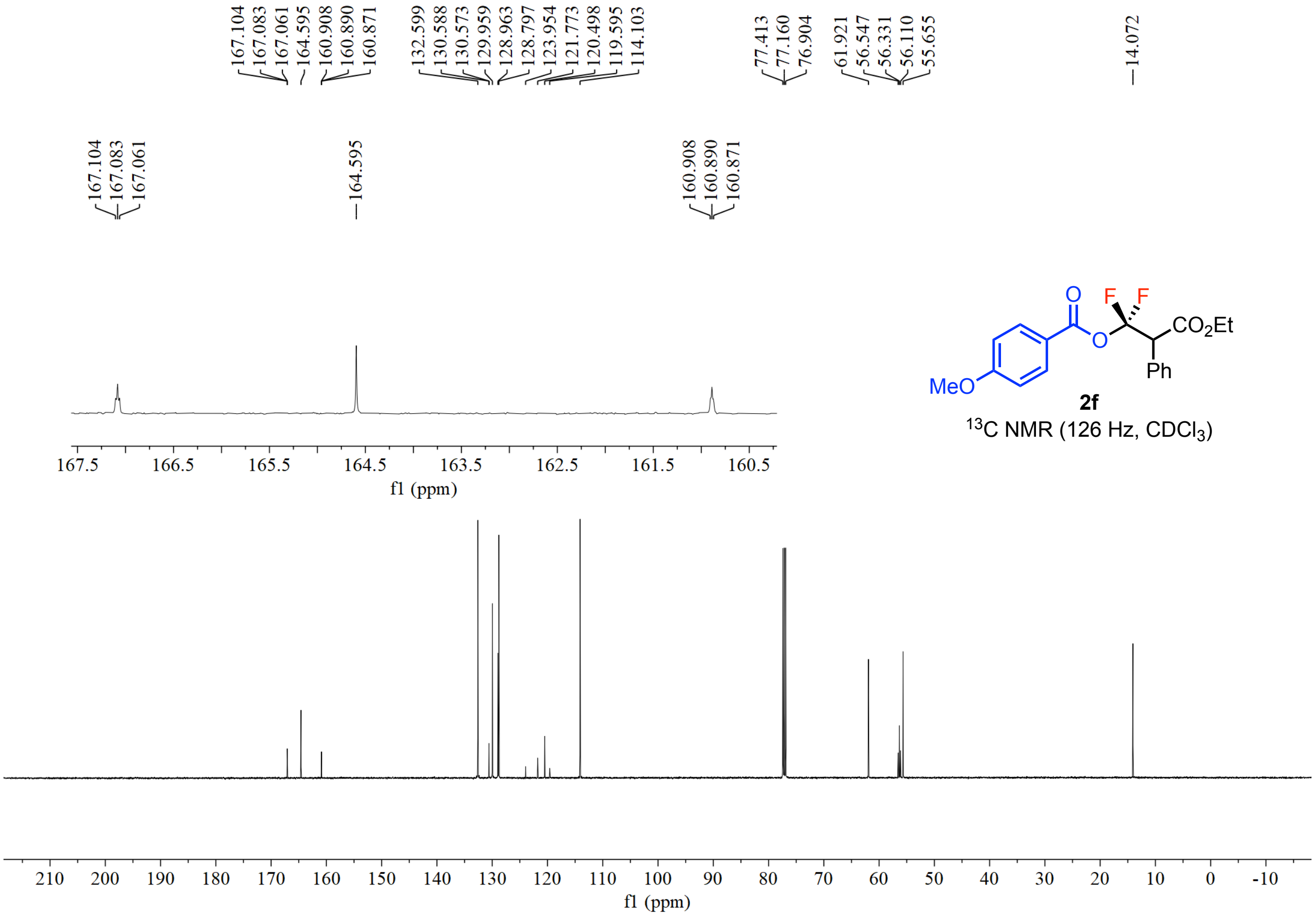
167.104
167.083
167.061
164.595
160.908
160.890
160.871
132.599
130.588
130.573
129.959
128.963
128.797
123.954
121.773
120.498
119.595
114.103
77.413
77.160
76.904
61.921
56.547
56.331
56.110
55.655
14.072
MeO
O
O
F
F
CO2Et
Ph
2f
13C NMR (126 Hz, CDCl3)
f1 (ppm)

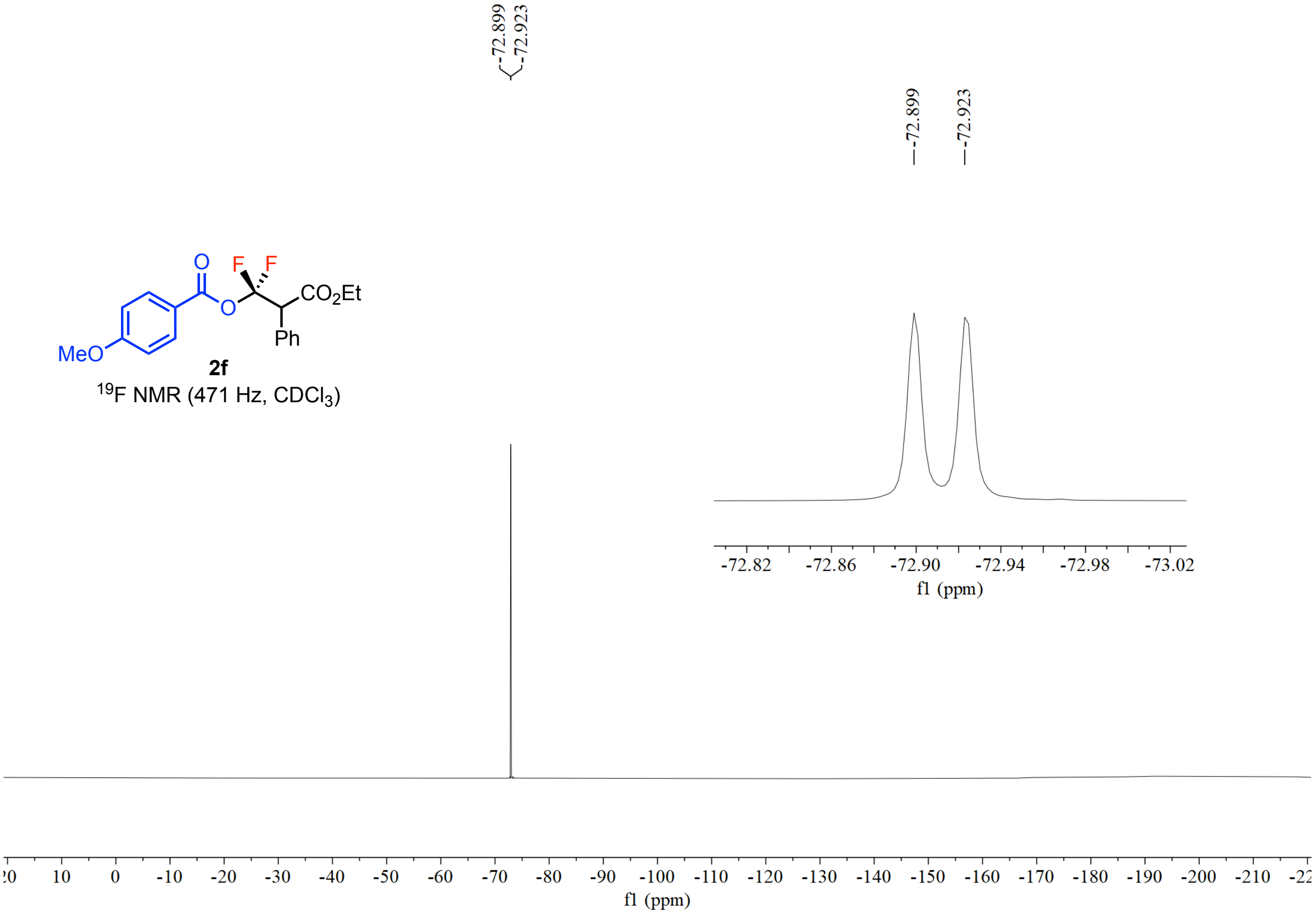
-72.899
-72.923
MeO
O
O
F
F
CO2Et
Ph
2f
19F NMR (471 Hz, CDCl3)
f1 (ppm)

2g

^{1}H NMR (500 Hz, CDCl$_3$)

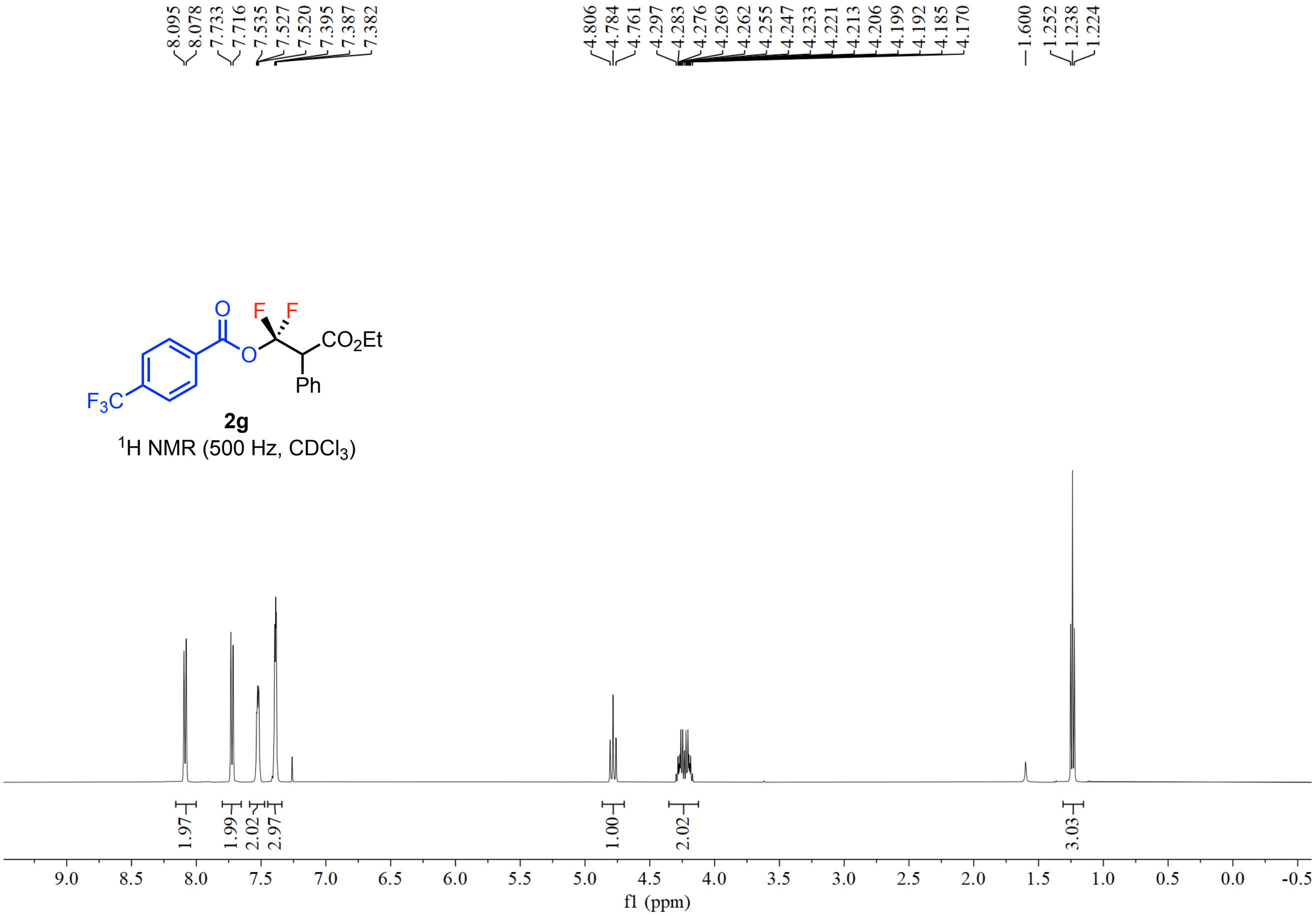

2g

^{13}C NMR (126 Hz, CDCl$_3$)

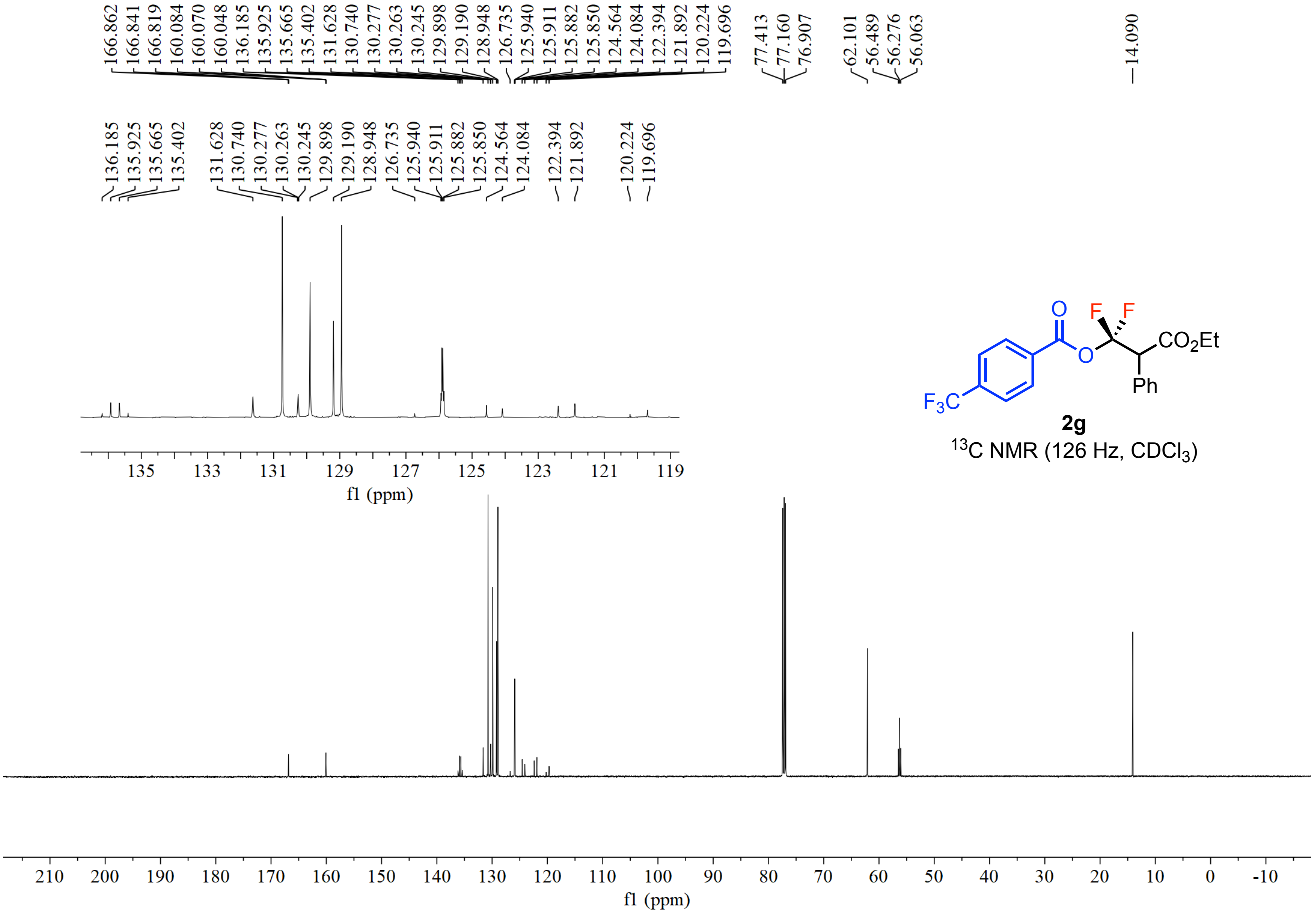

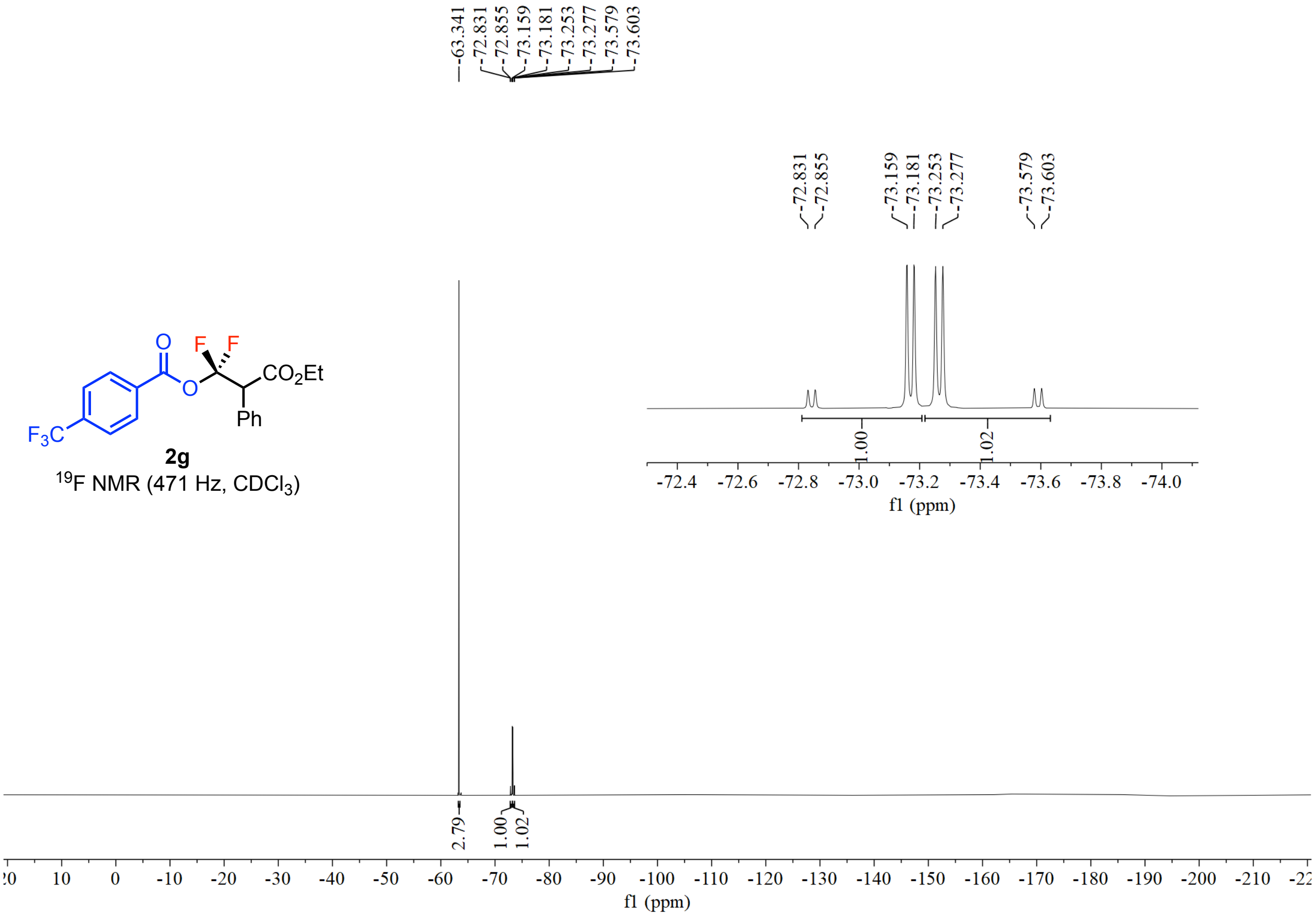
-63.341
-72.831
-72.855
-73.159
-73.181
-73.253
-73.277
-73.579
-73.603
2g
^{19}F NMR (471 Hz, CDCl$_3$)
f1 (ppm)

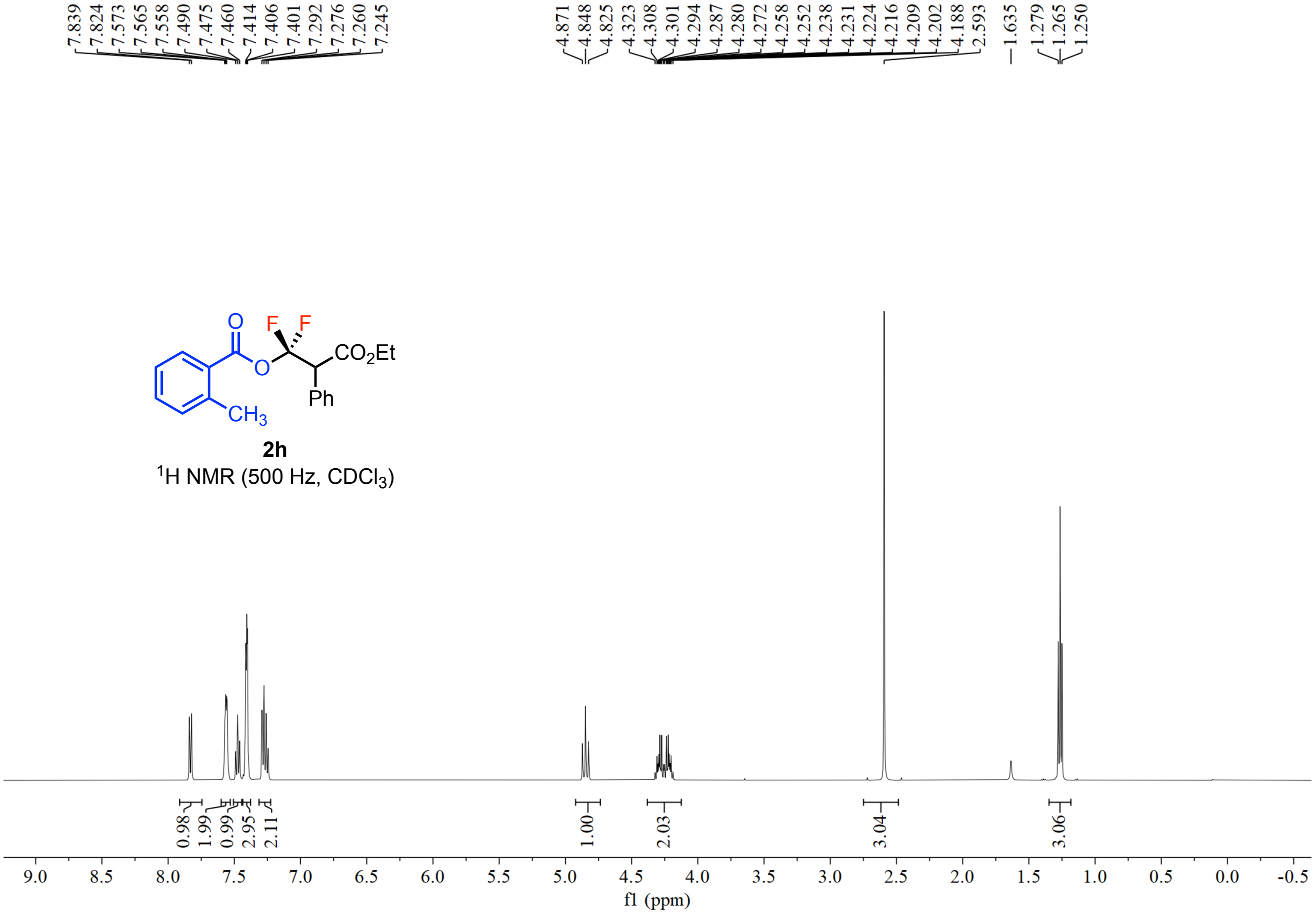
7.839
7.824
7.573
7.565
7.558
7.490
7.475
7.460
7.414
7.406
7.401
7.292
7.276
7.260
7.245
4.871
4.848
4.825
4.323
4.308
4.301
4.294
4.287
4.280
4.272
4.258
4.252
4.238
4.231
4.224
4.216
4.209
4.202
4.188
2.593
1.635
1.279
1.265
1.250
2h
^{1}H NMR (500 Hz, CDCl$_3$)
f1 (ppm)

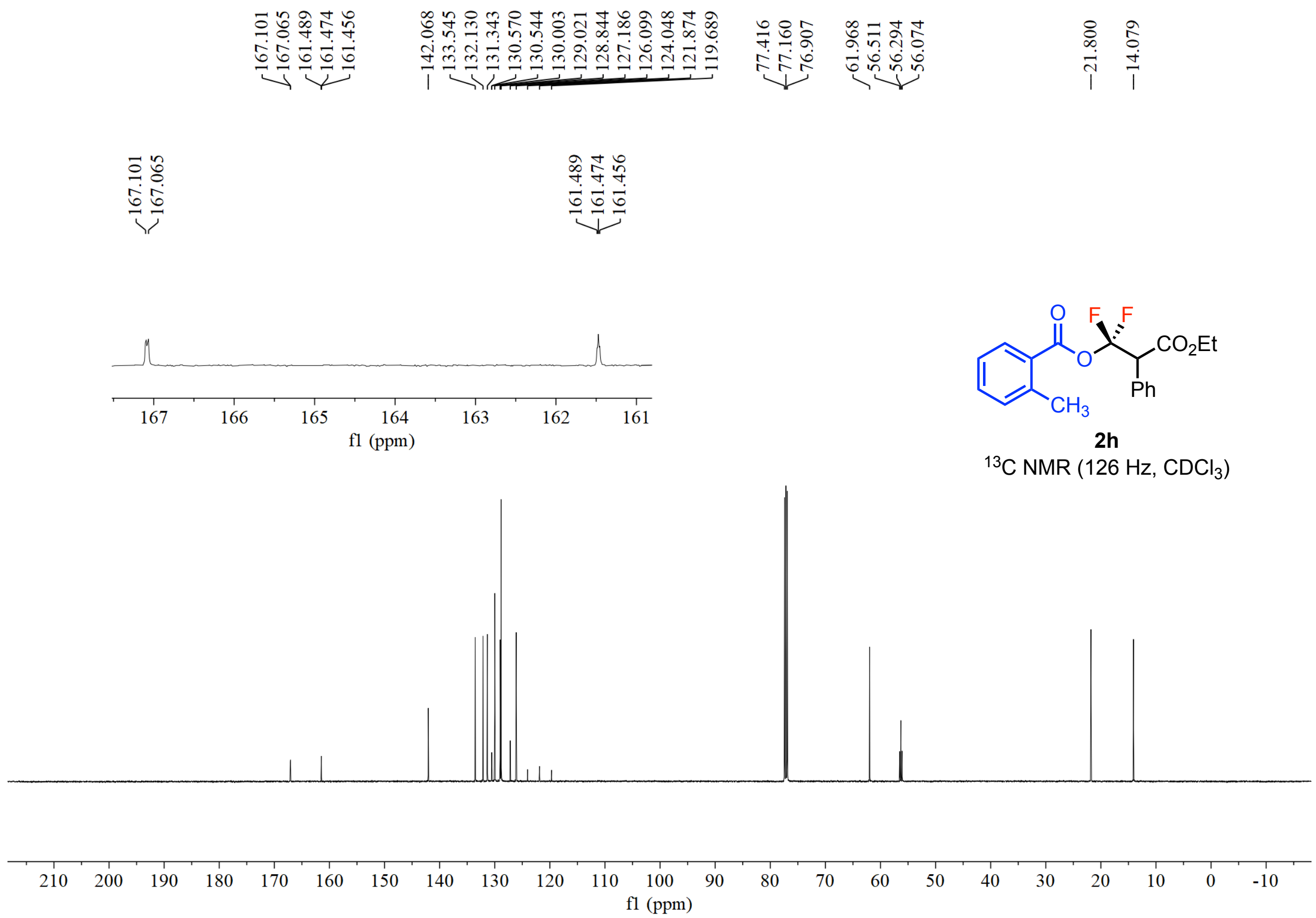
167.101
167.065
161.489
161.474
161.456
142.068
133.545
132.130
131.343
130.570
130.544
130.003
129.021
128.844
127.186
126.099
124.048
121.874
119.689
77.416
77.160
76.907
61.968
56.511
56.294
56.074
21.800
14.079
f1 (ppm)
O
F
F
O
CO2Et
Ph
CH3
2h
13C NMR (126 Hz, CDCl3)

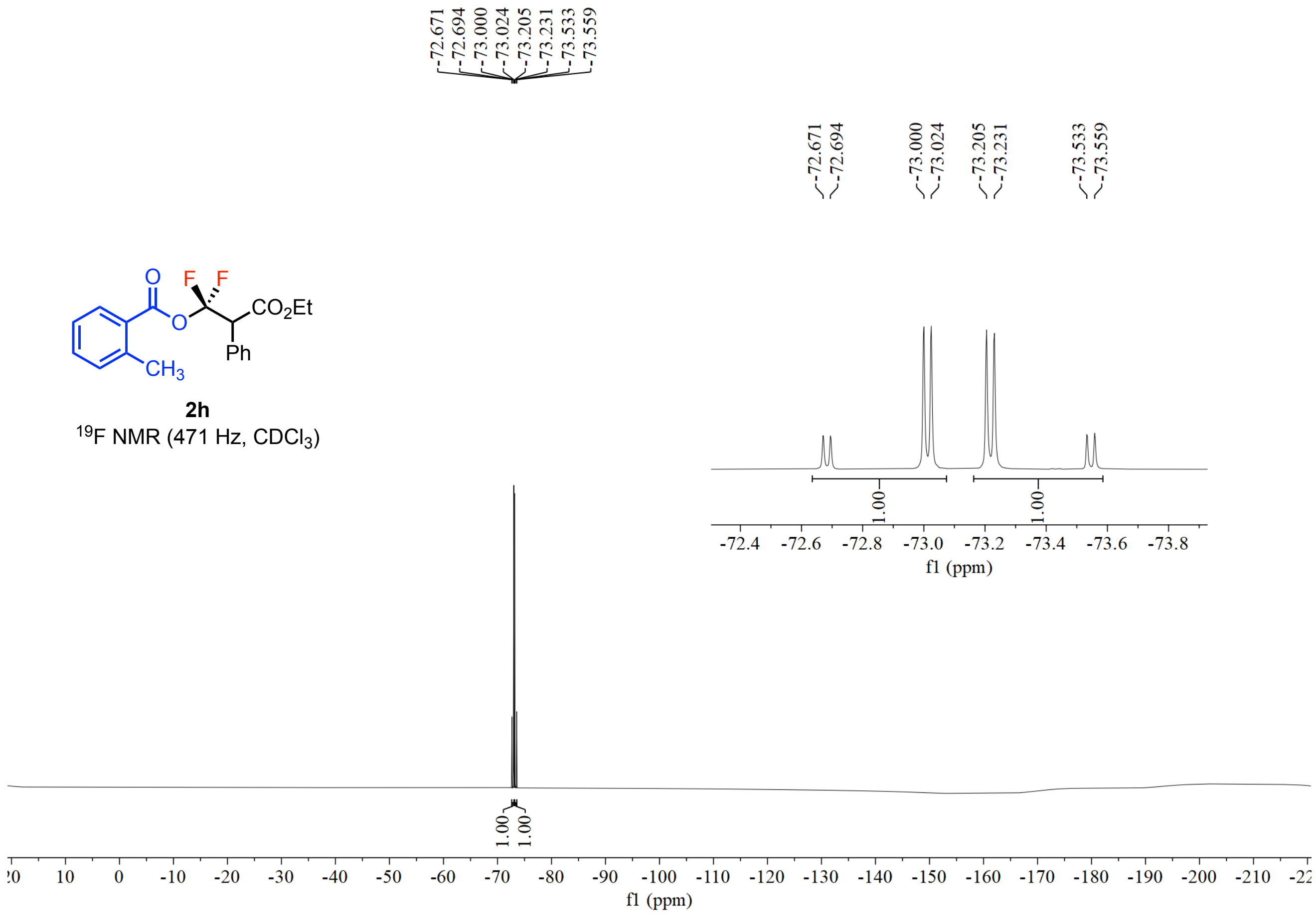
-72.671
-72.694
-73.000
-73.024
-73.205
-73.231
-73.533
-73.559
O
F
F
O
CO2Et
Ph
CH3
2h
19F NMR (471 Hz, CDCl3)
1.00
1.00
f1 (ppm)

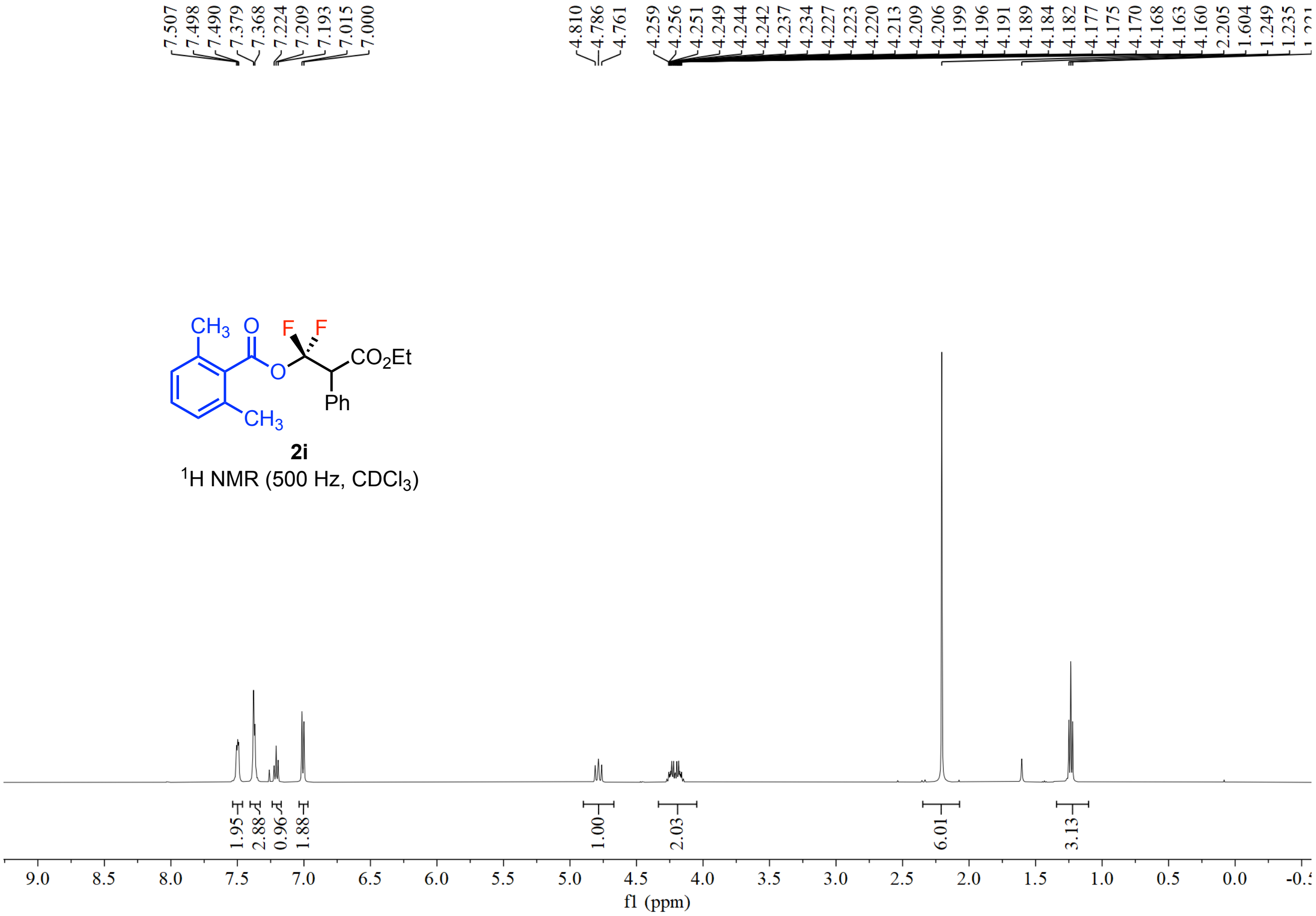
7.507
7.498
7.490
7.379
7.368
7.224
7.209
7.193
7.015
7.000
4.810
4.786
4.761
4.259
4.256
4.251
4.249
4.244
4.242
4.237
4.234
4.227
4.223
4.220
4.213
4.209
4.206
4.199
4.196
4.191
4.189
4.184
4.182
4.177
4.175
4.170
4.168
4.163
4.160
2.205
1.604
1.249
1.235
CH3
O
F
F
CO2Et
O
Ph
CH3
2i
1H NMR (500 Hz, CDCl3)
1.95
2.88
0.96
1.88
1.00
2.03
6.01
3.13
f1 (ppm)

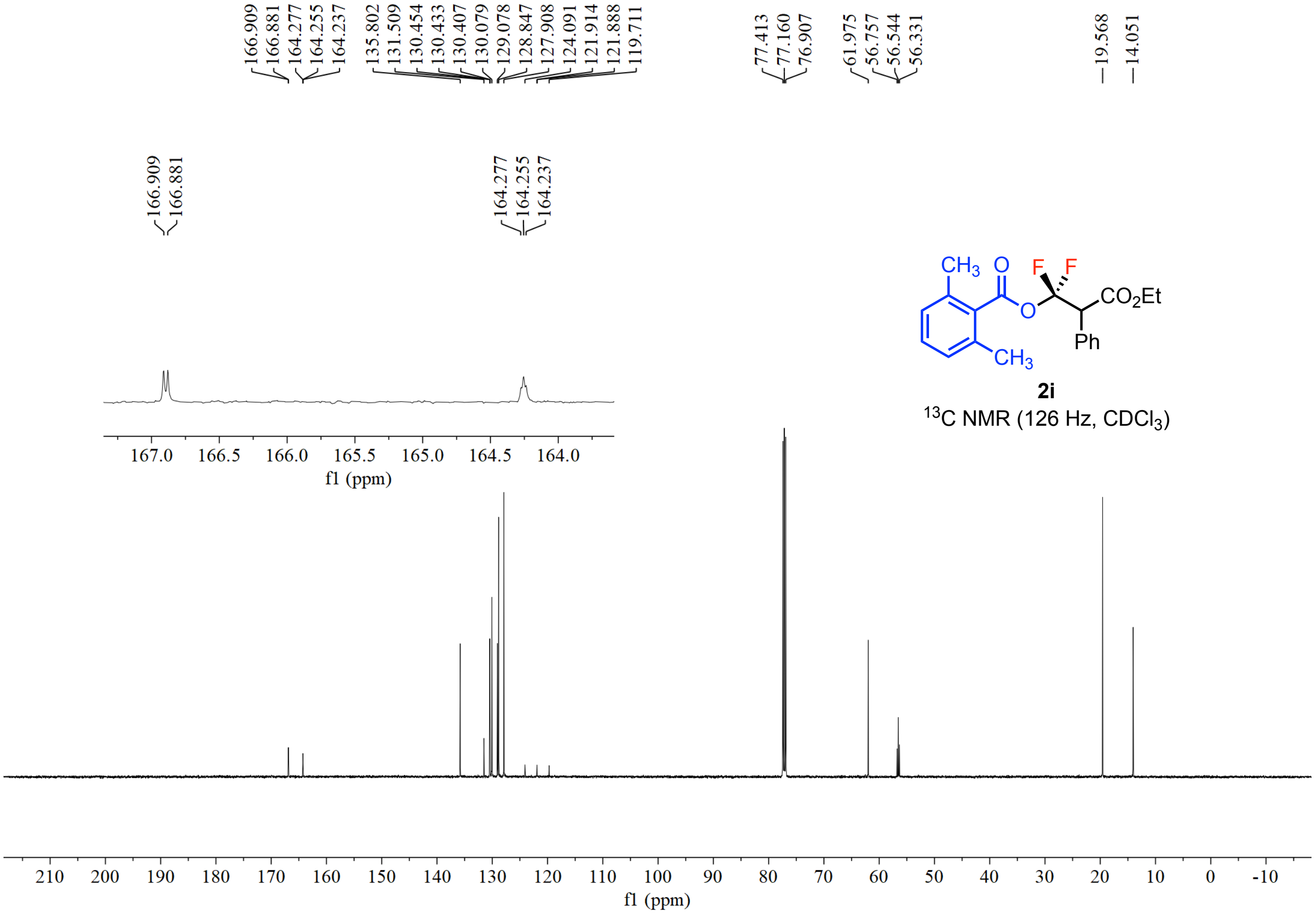
166.909
166.881
164.277
164.255
164.237
135.802
131.509
130.454
130.433
130.407
130.079
129.078
128.847
127.908
124.091
121.914
121.888
119.711
77.413
77.160
76.907
61.975
56.757
56.544
56.331
19.568
14.051
CH3
O
F
F
CO2Et
O
Ph
CH3
2i
13C NMR (126 Hz, CDCl3)
f1 (ppm)

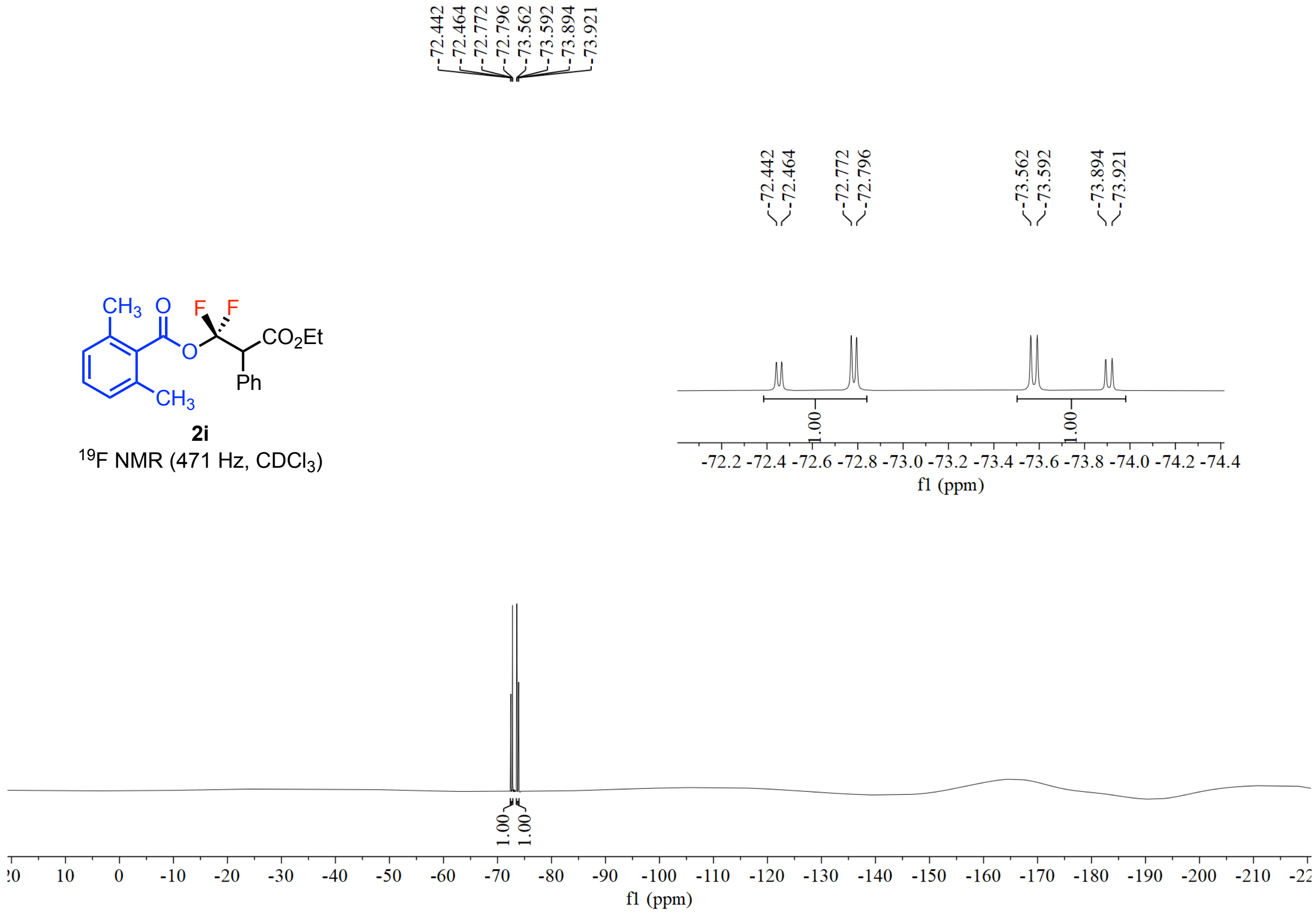
2i
19F NMR (471 Hz, CDCl3)
f1 (ppm)

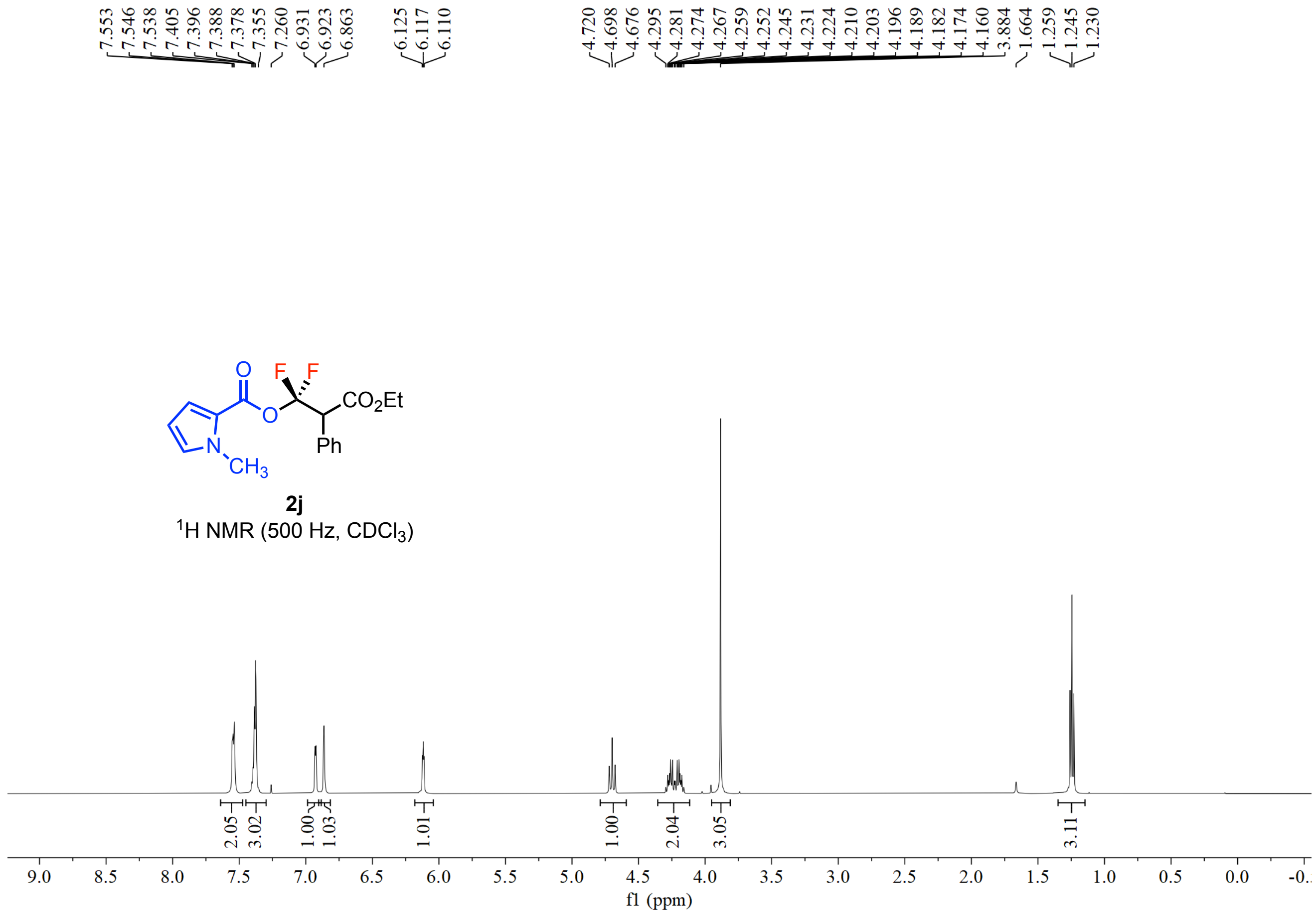
2j
1H NMR (500 Hz, CDCl3)
f1 (ppm)

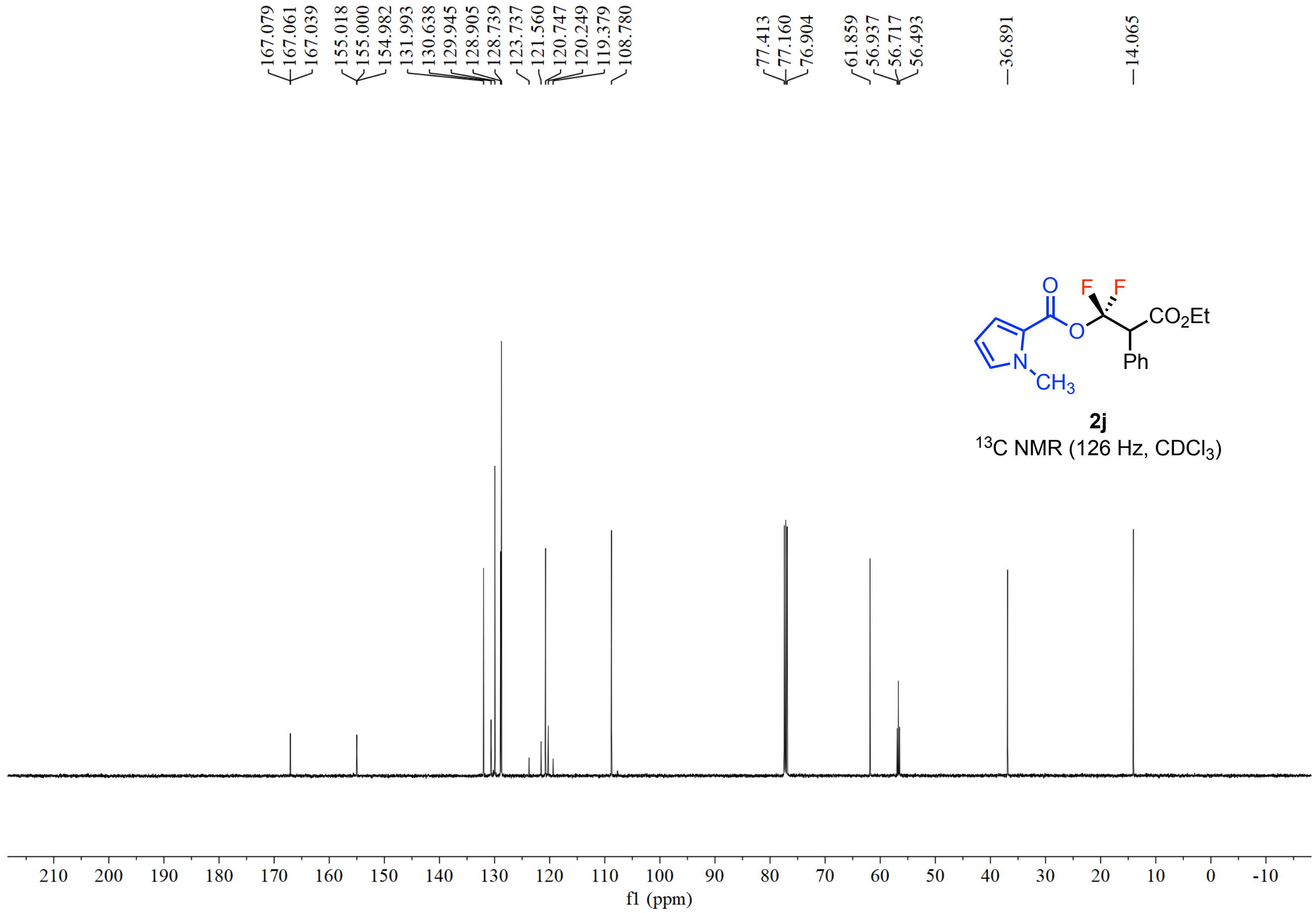
167.079
167.061
167.039
155.018
155.000
154.982
131.993
130.638
129.945
128.905
128.739
123.737
121.560
120.747
120.249
119.379
108.780
77.413
77.160
76.904
61.859
56.937
56.717
56.493
36.891
14.065
CO2Et
Ph
CH3
2j
13C NMR (126 Hz, CDCl3)
f1 (ppm)

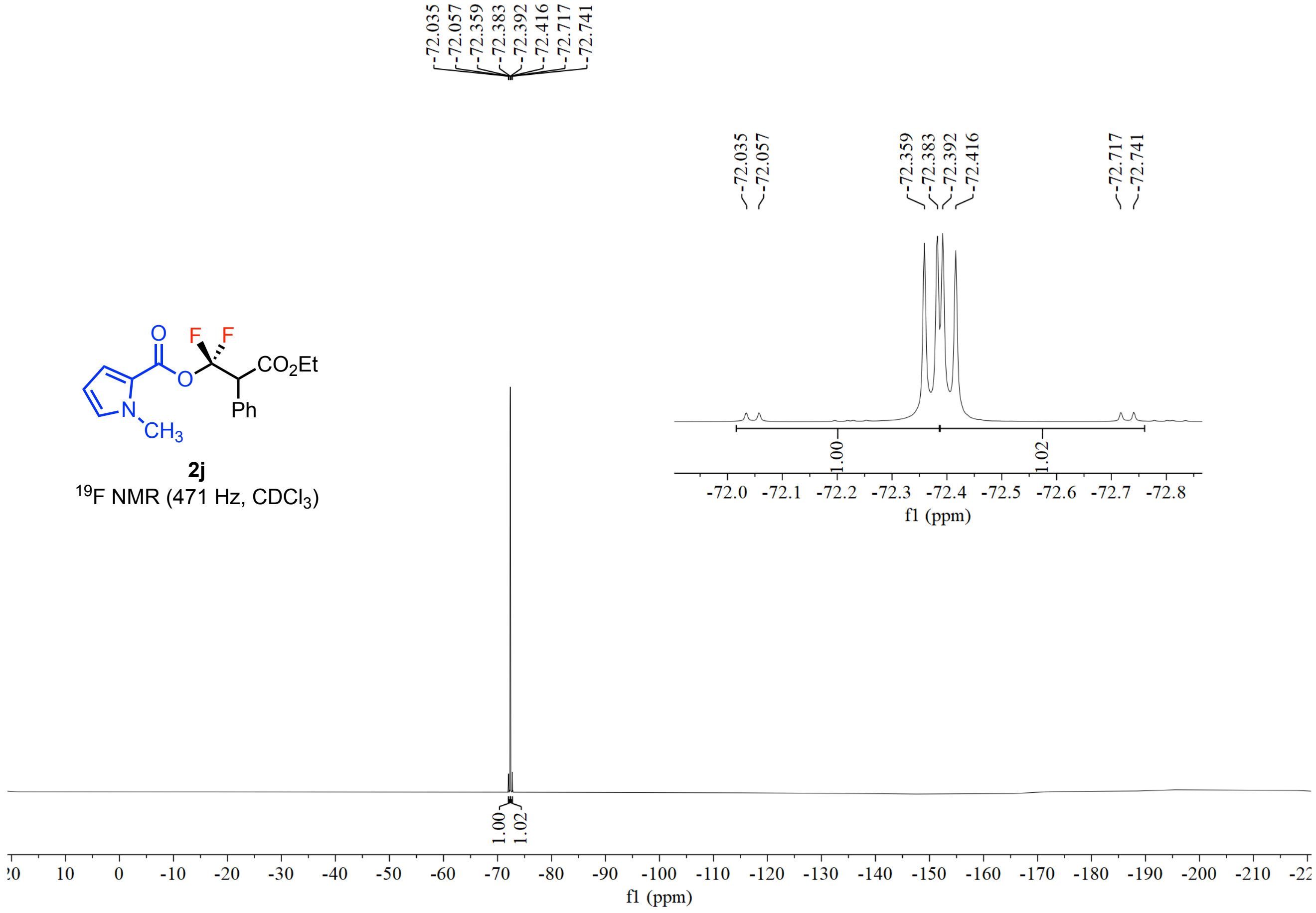
-72.035
-72.057
-72.359
-72.383
-72.392
-72.416
-72.717
-72.741
CO2Et
Ph
CH3
2j
19F NMR (471 Hz, CDCl3)
1.00
1.02
f1 (ppm)

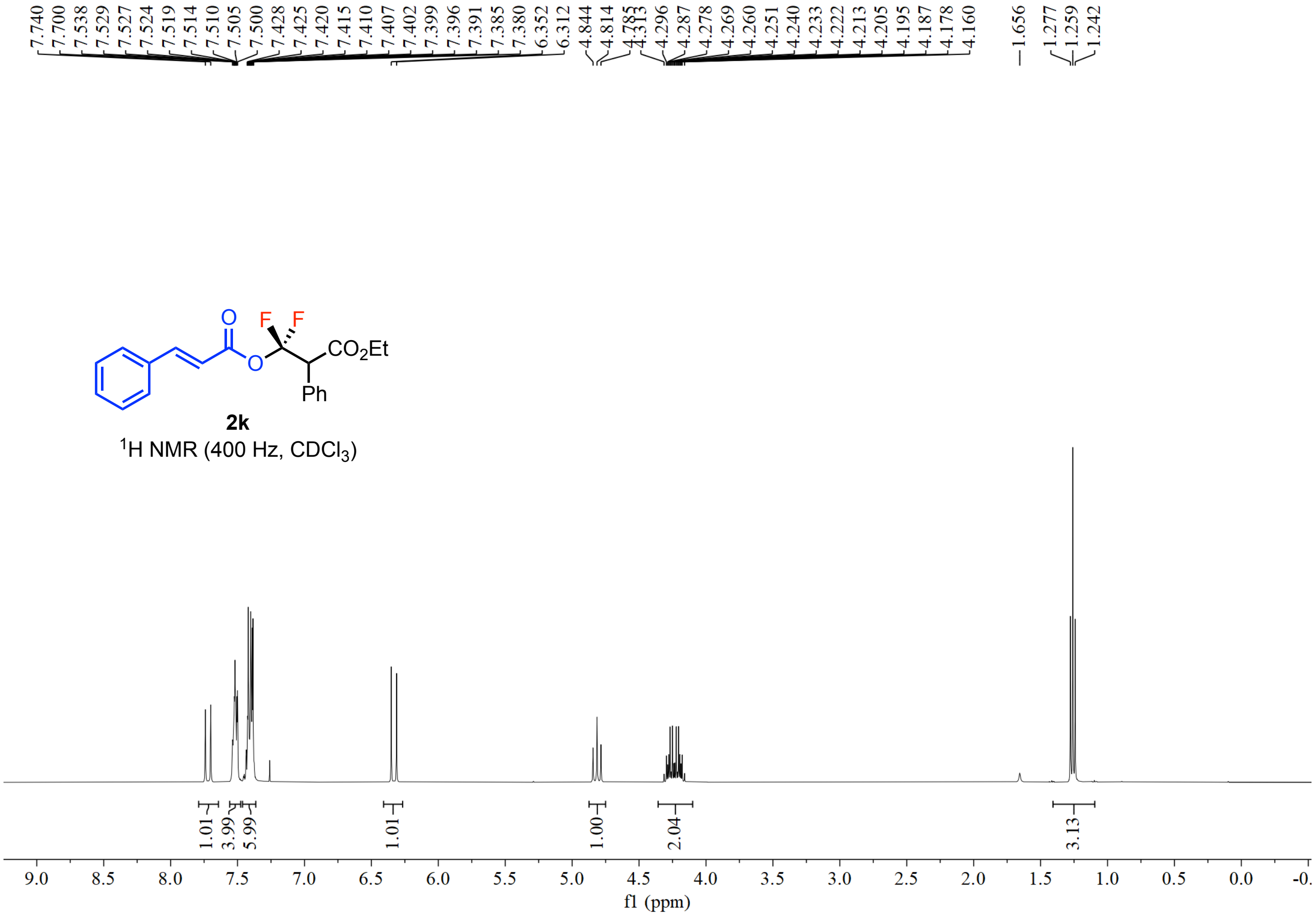
7.740 7.700 7.538 7.529 7.527 7.524 7.519 7.514 7.510 7.505 7.500 7.428 7.425 7.420 7.415 7.410 7.407 7.402 7.399 7.396 7.391 7.385 7.380 6.352 6.312 4.844 4.814 4.785 4.313 4.296 4.287 4.278 4.269 4.260 4.251 4.240 4.233 4.222 4.213 4.205 4.195 4.187 4.178 4.160
1.656
1.277 1.259 1.242
O
F F
CO_2Et
O
Ph
2k
^{1}H NMR (400 Hz, $CDCl_3$)
1.01 3.99 5.99 1.01 1.00 2.04 3.13
9.0 8.5 8.0 7.5 7.0 6.5 6.0 5.5 5.0 4.5 4.0 3.5 3.0 2.5 2.0 1.5 1.0 0.5 0.0 -0.
f1 (ppm)

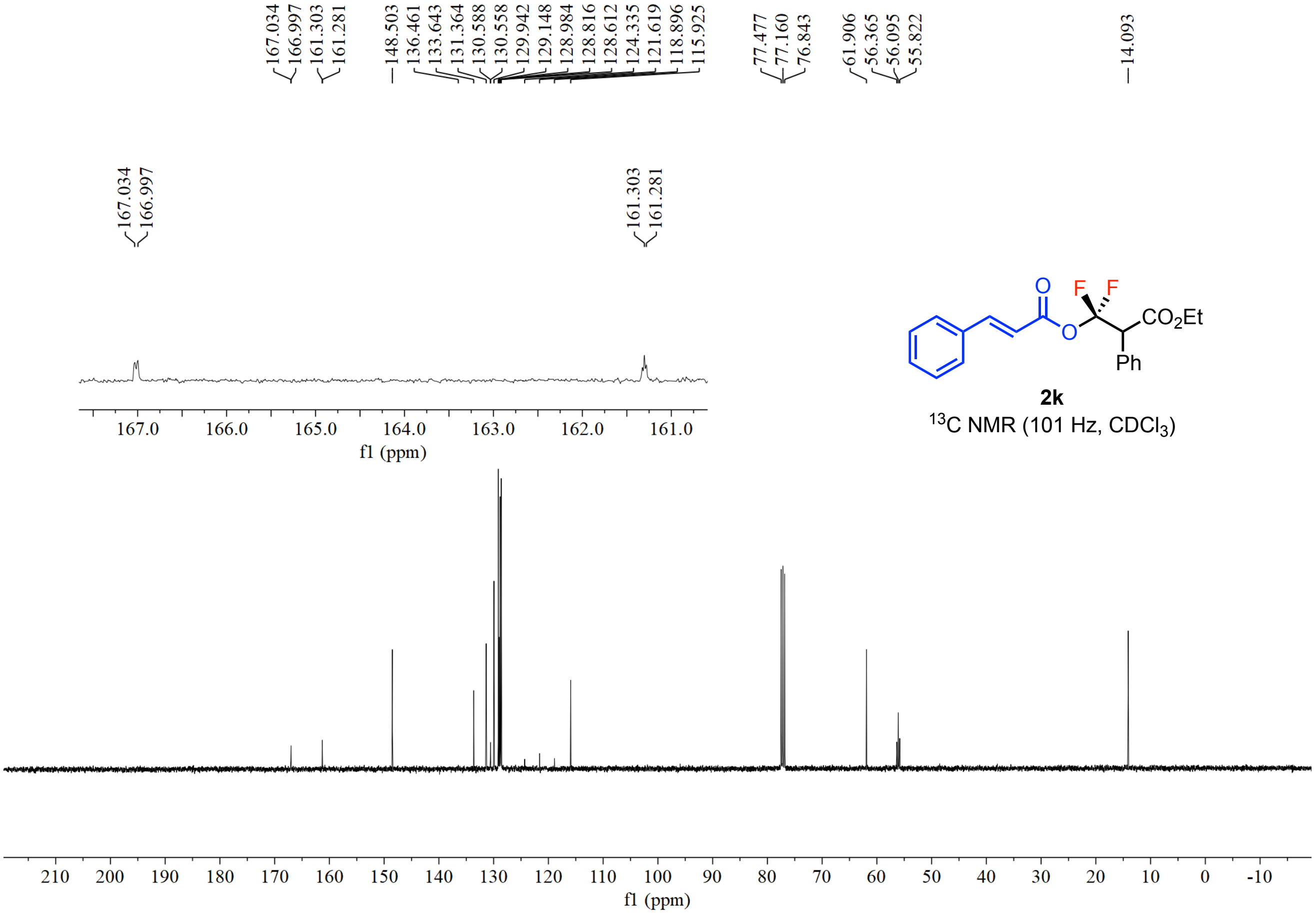
167.034 166.997 161.303 161.281
148.503 136.461 133.643 131.364 130.588 130.558 129.942 129.148 128.984 128.816 128.612 124.335 121.619 118.896 115.925
77.477 77.160 76.843
61.906 56.365 56.095 55.822
14.093
167.034 166.997
161.303 161.281
167.0 166.0 165.0 164.0 163.0 162.0 161.0
f1 (ppm)
O
F F
CO_2Et
O
Ph
2k
^{13}C NMR (101 Hz, $CDCl_3$)
210 200 190 180 170 160 150 140 130 120 110 100 90 80 70 60 50 40 30 20 10 0 -10
f1 (ppm)

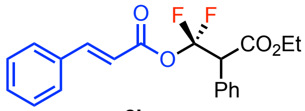

2k

^{19}F NMR (471 Hz, $CDCl_3$)

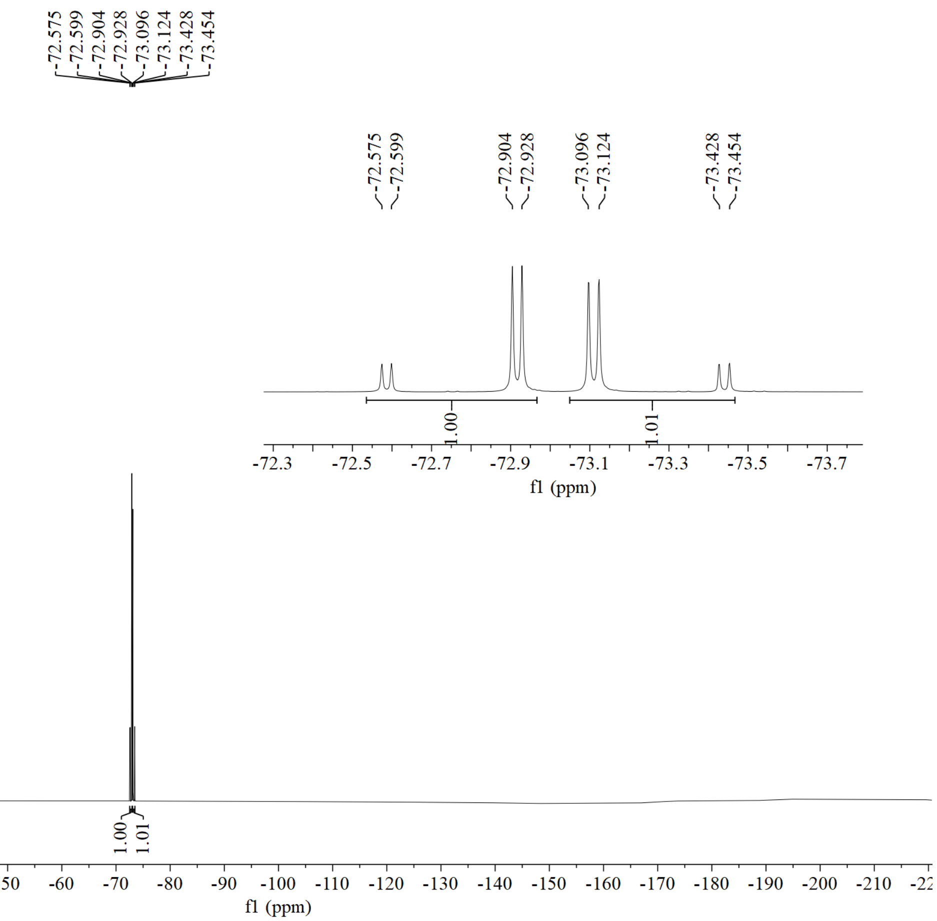

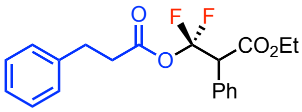

2l

^{1}H NMR (500 Hz, $CDCl_3$)

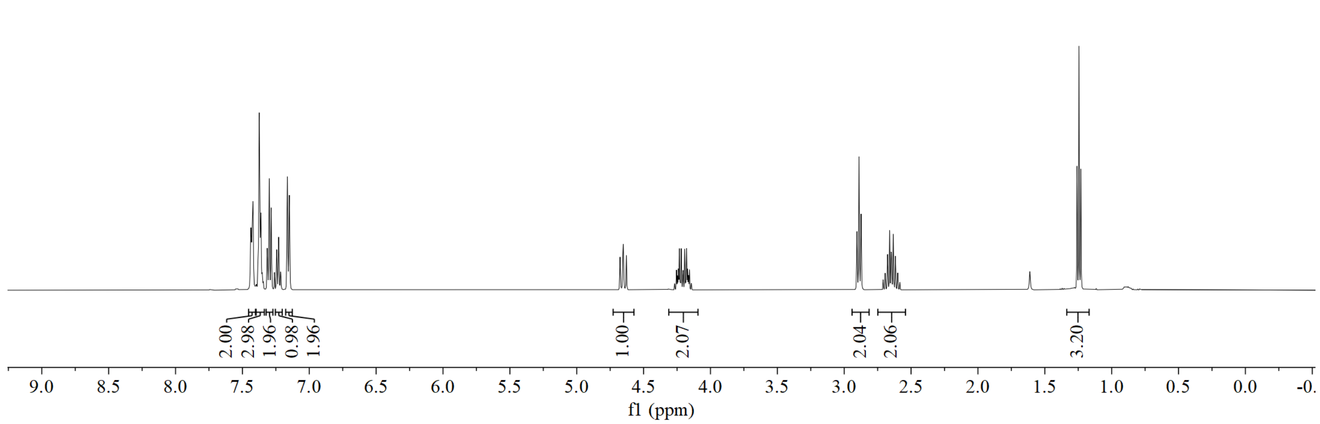

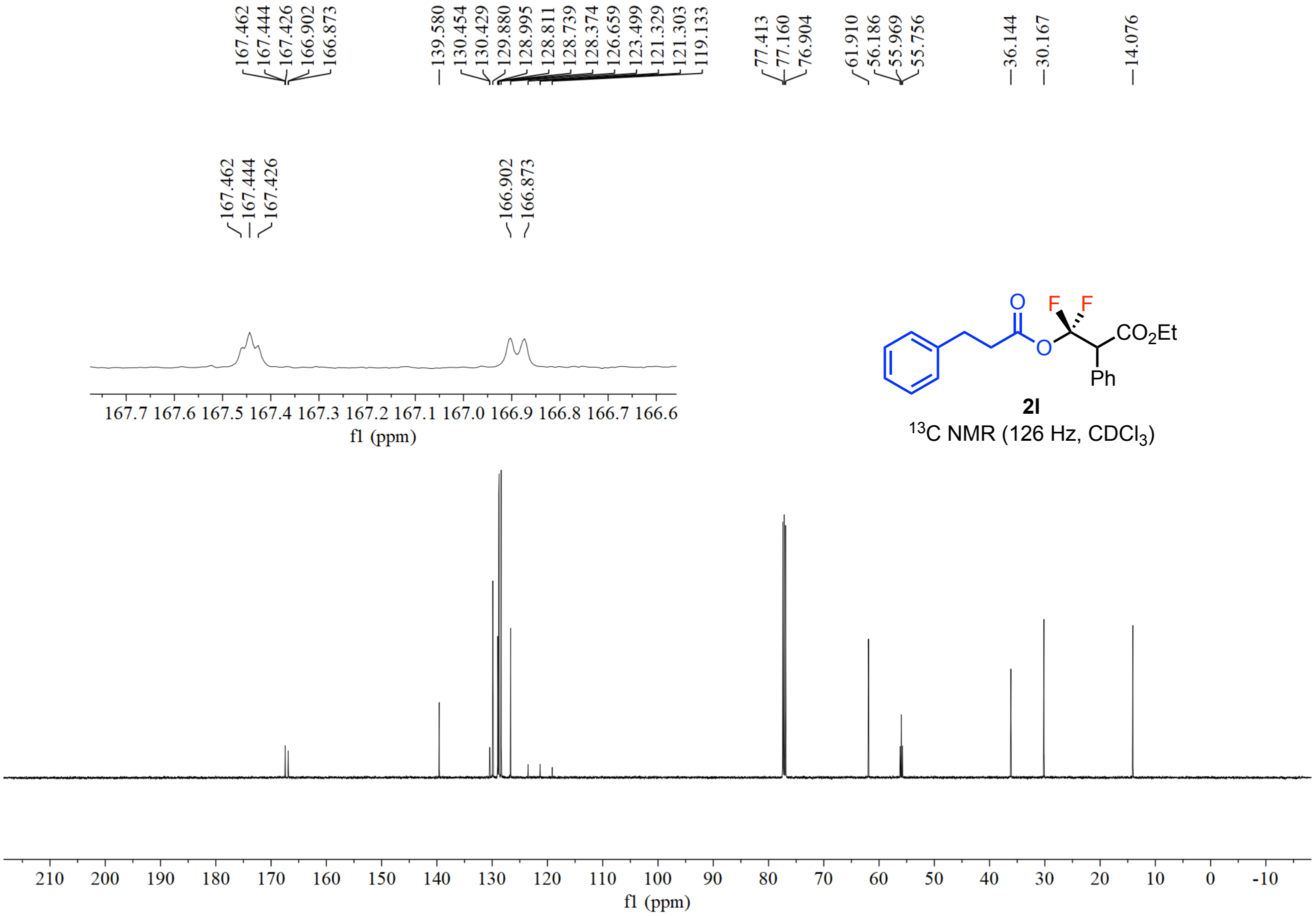

2l

^{13}C NMR (126 Hz, $CDCl_3$)

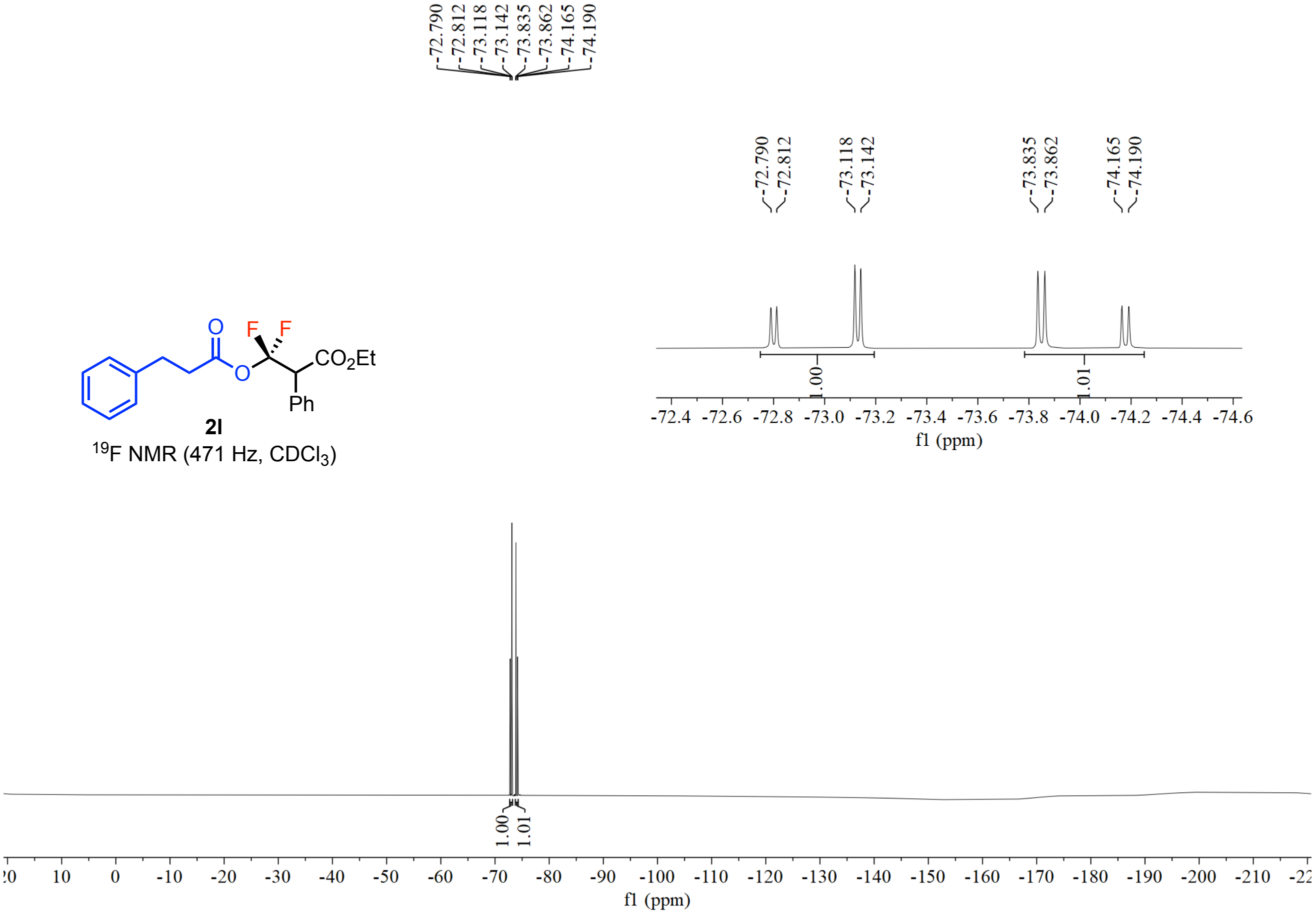

2l

^{19}F NMR (471 Hz, $CDCl_3$)

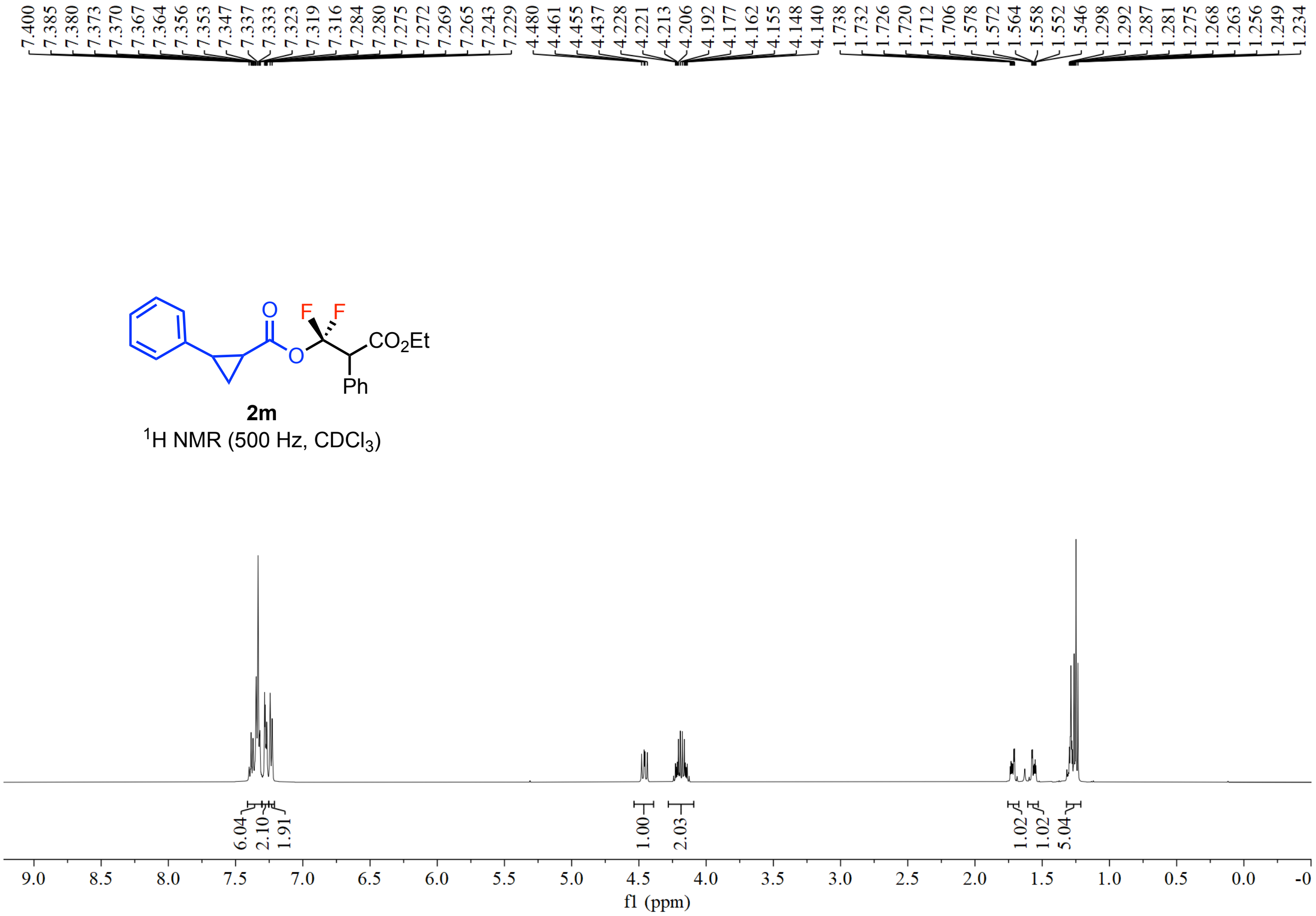

2m

^{1}H NMR (500 Hz, $CDCl_3$)

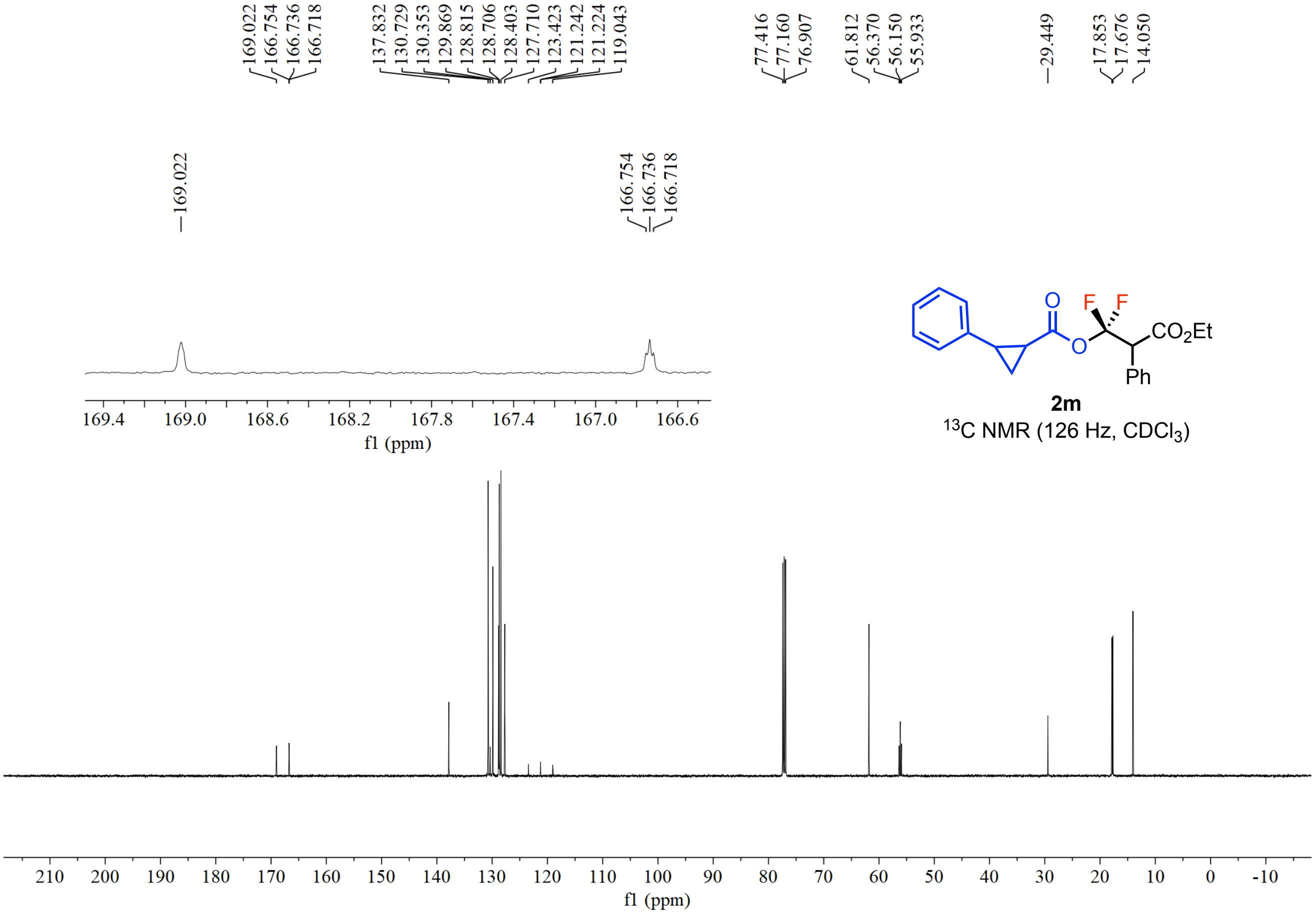

2m

^{13}C NMR (126 Hz, $CDCl_3$)

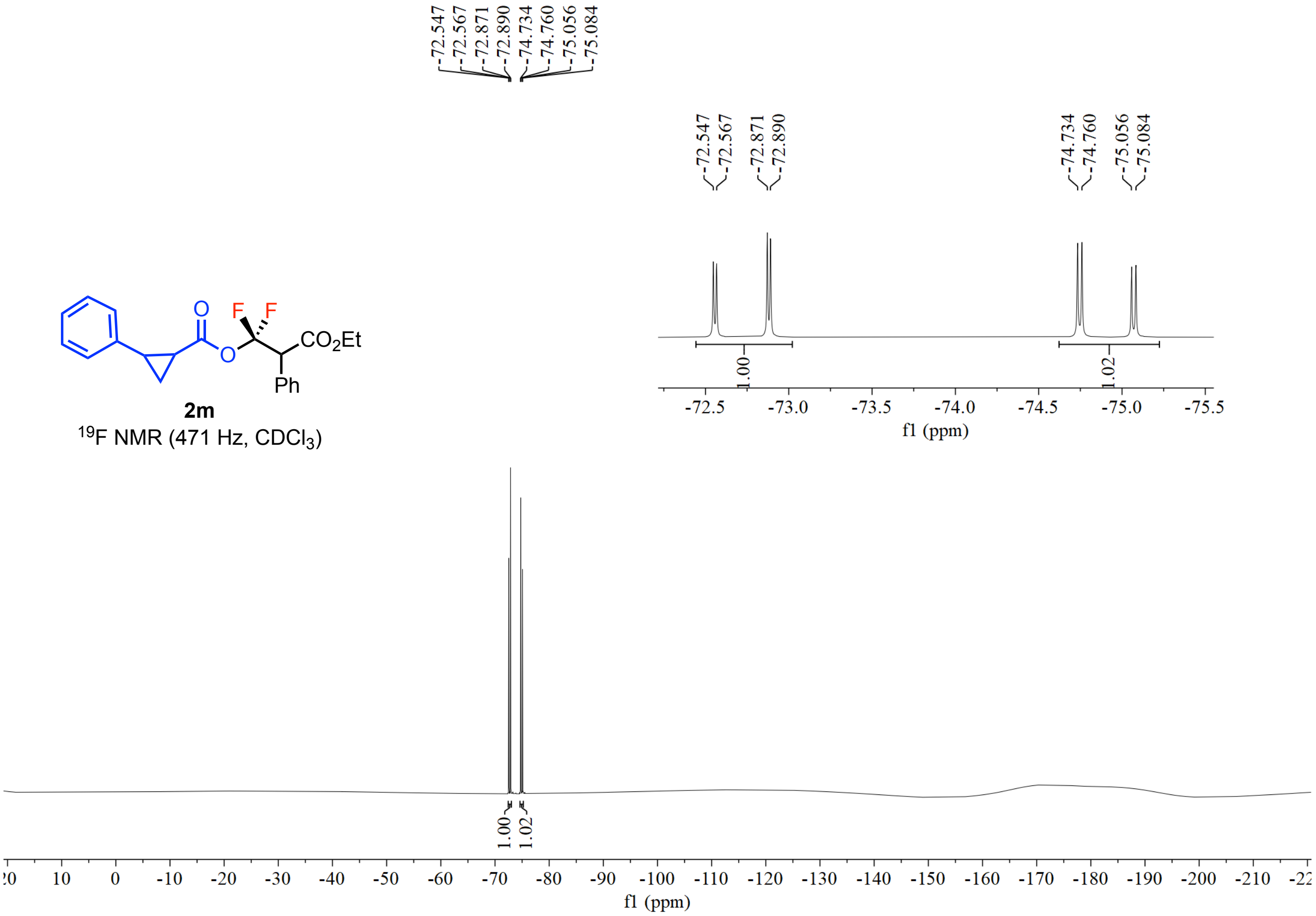
-72.547
-72.567
-72.871
-72.890
-74.734
-74.760
-75.056
-75.084
O
F
F
CO2Et
O
Ph
2m
19F NMR (471 Hz, CDCl3)
1.00
1.02
-72.5
-73.0
-73.5
-74.0
-74.5
-75.0
-75.5
f1 (ppm)
10
0
-10
-20
-30
-40
-50
-60
-70
-80
-90
-100
-110
-120
-130
-140
-150
-160
-170
-180
-190
-200
-210

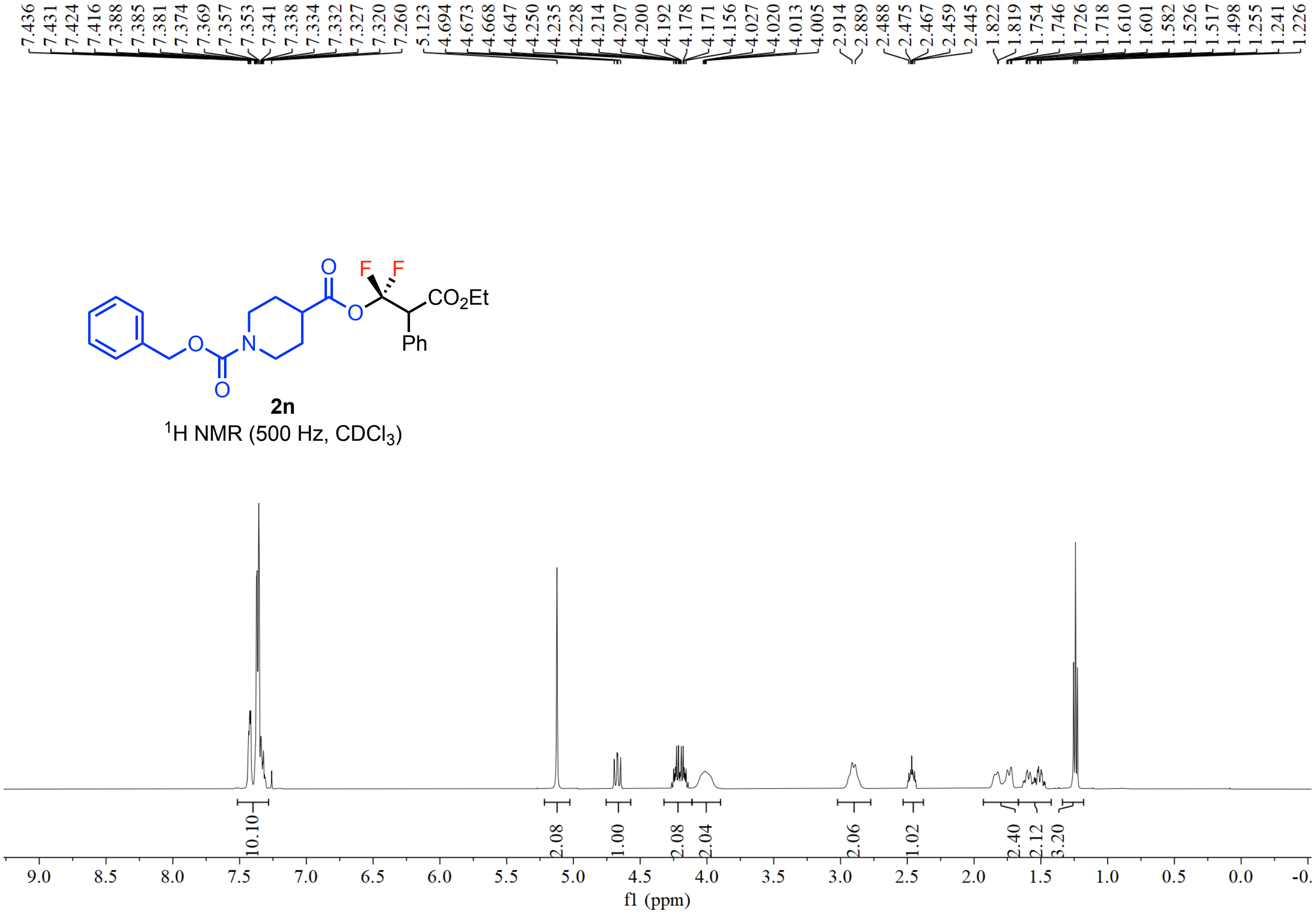
7.436
7.431
7.424
7.416
7.388
7.385
7.381
7.374
7.369
7.357
7.353
7.341
7.338
7.334
7.332
7.327
7.320
7.260
5.123
4.694
4.673
4.668
4.647
4.250
4.235
4.228
4.214
4.207
4.200
4.192
4.178
4.171
4.156
4.027
4.020
4.013
4.005
2.914
2.889
2.488
2.475
2.467
2.459
2.445
1.822
1.819
1.754
1.746
1.726
1.718
1.610
1.601
1.582
1.526
1.517
1.498
1.255
1.241
1.226
O
F
F
CO2Et
O
O
N
O
Ph
2n
1H NMR (500 Hz, CDCl3)
10.10
2.08
1.00
2.08
2.04
2.06
1.02
2.40
2.12
3.20
9.0
8.5
8.0
7.5
7.0
6.5
6.0
5.5
5.0
4.5
4.0
3.5
3.0
2.5
2.0
1.5
1.0
0.5
0.0
f1 (ppm)

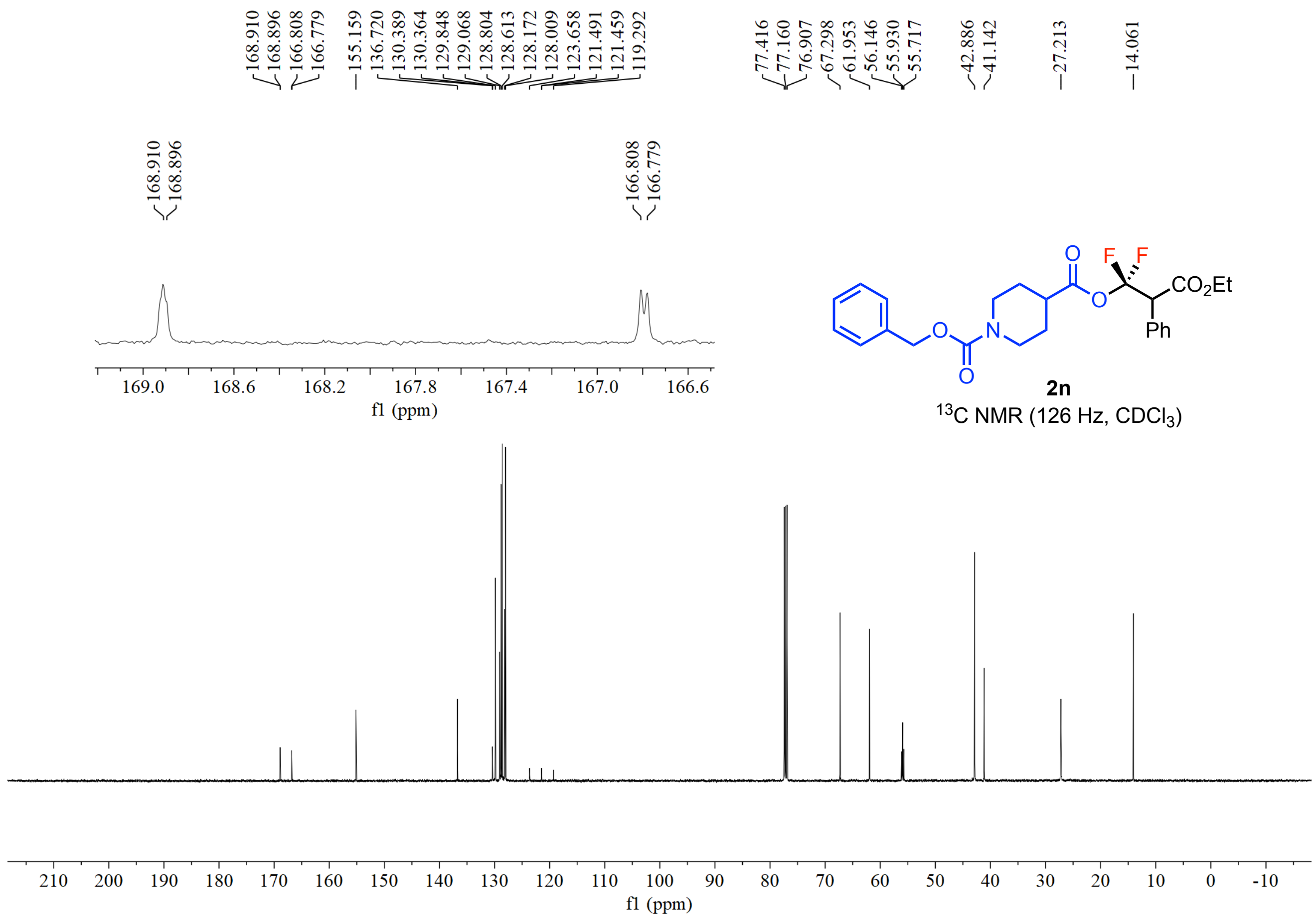

2n

^{13}C NMR (126 Hz, $CDCl_3$)

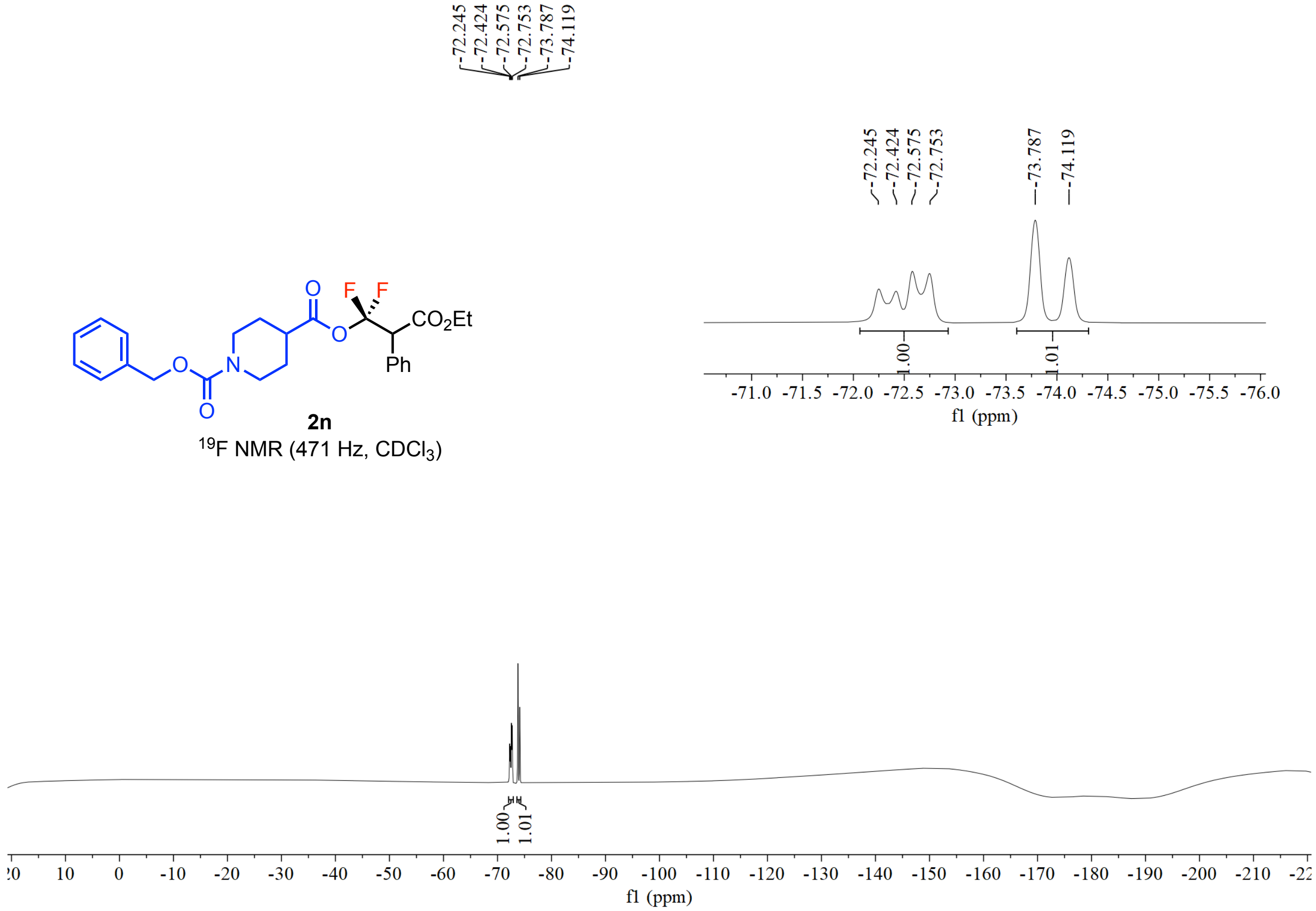

2n

^{19}F NMR (471 Hz, $CDCl_3$)

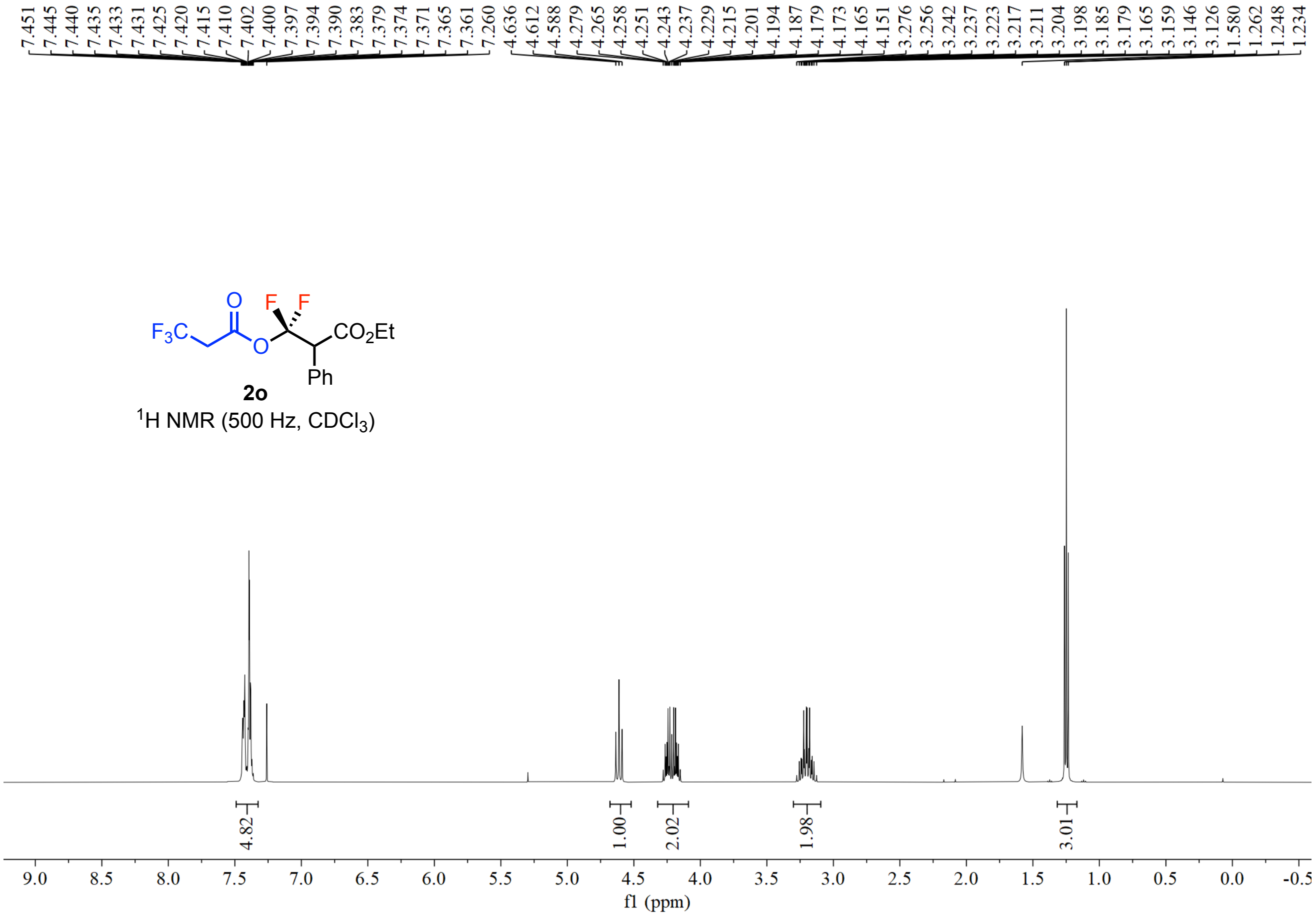
7.451 7.445 7.440 7.435 7.433 7.431 7.425 7.420 7.415 7.410 7.402 7.400 7.397 7.394 7.390 7.383 7.379 7.374 7.371 7.365 7.361 7.260 4.636 4.612 4.588 4.279 4.265 4.258 4.251 4.243 4.237 4.229 4.215 4.201 4.194 4.187 4.179 4.173 4.165 4.151 3.276 3.256 3.242 3.237 3.223 3.217 3.211 3.204 3.198 3.185 3.179 3.165 3.159 3.146 3.126 1.580 1.262 1.248 1.234
F3C O F F CO2Et O Ph
2o
1H NMR (500 Hz, CDCl3)
4.82 1.00 2.02 1.98 3.01
f1 (ppm)

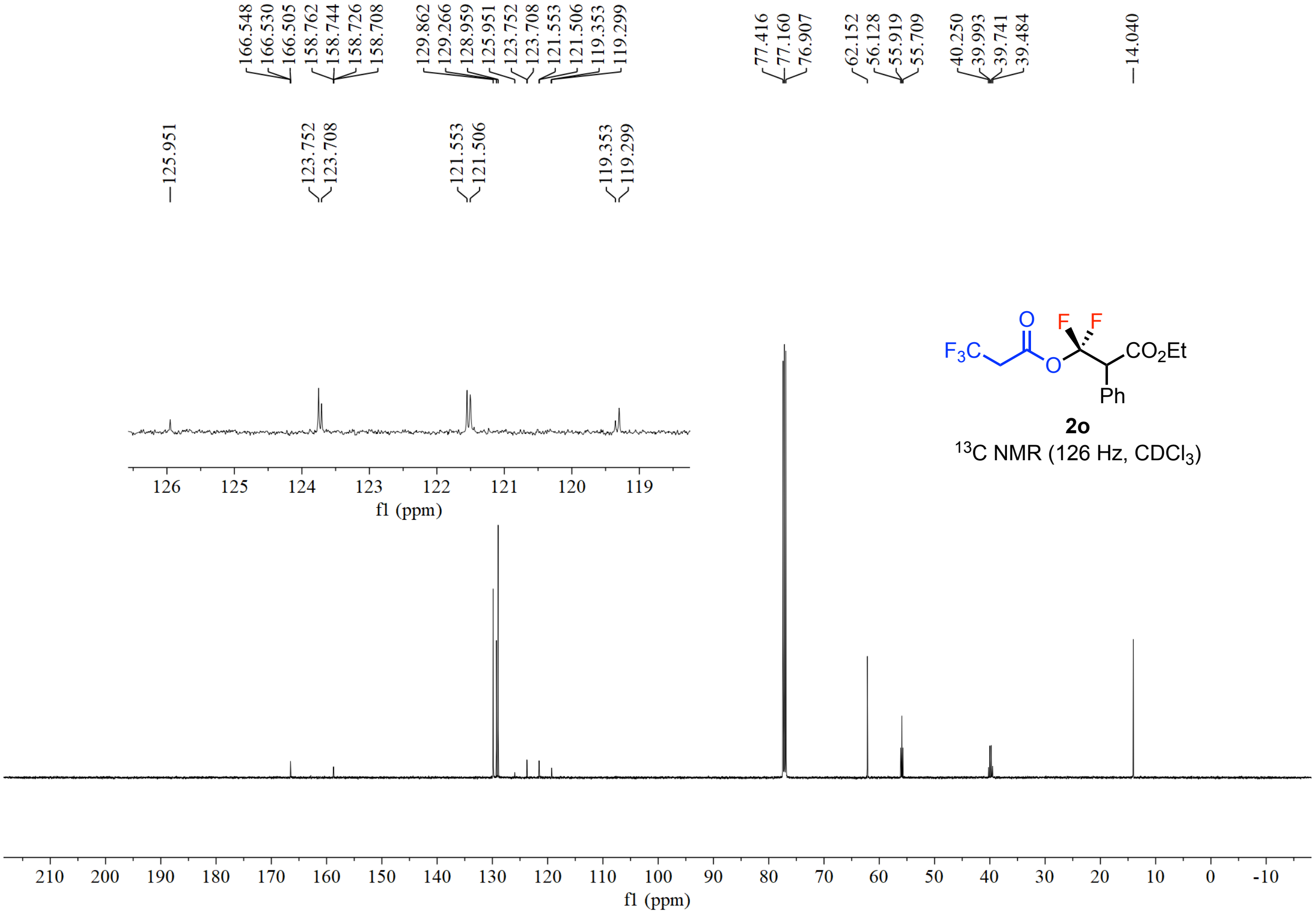
166.548 166.530 166.505 158.762 158.744 158.726 158.708 129.862 129.266 128.959 125.951 123.752 123.708 121.553 121.506 119.353 119.299 77.416 77.160 76.907 62.152 56.128 55.919 55.709 40.250 39.993 39.741 39.484 14.040
125.951 123.752 123.708 121.553 121.506 119.353 119.299
F3C O F F CO2Et O Ph
2o
13C NMR (126 Hz, CDCl3)
f1 (ppm)

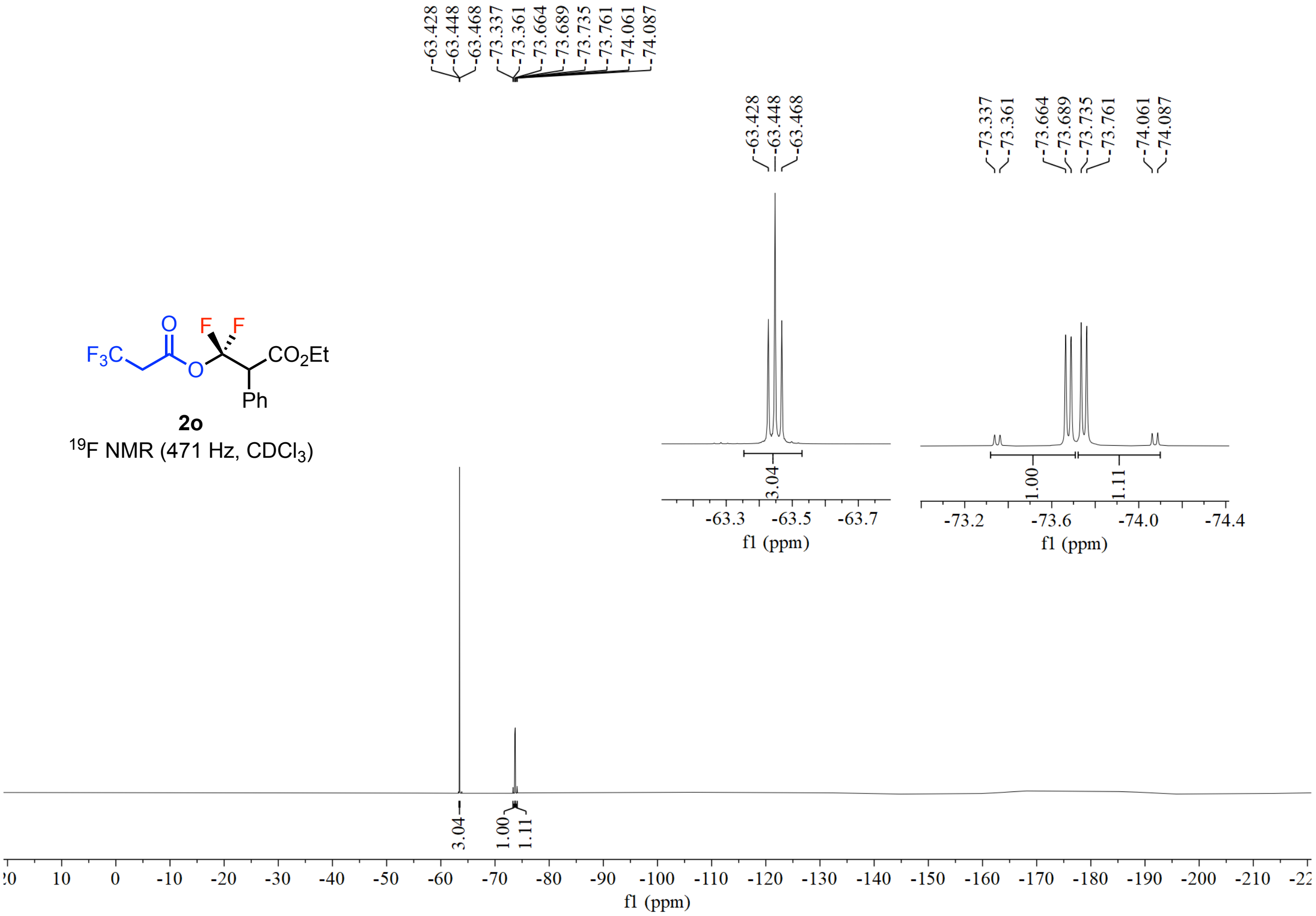
-63.428
-63.448
-63.468
-73.337
-73.361
-73.664
-73.689
-73.735
-73.761
-74.061
-74.087
2o
19F NMR (471 Hz, CDCl3)
3.04
1.00
1.11
f1 (ppm)

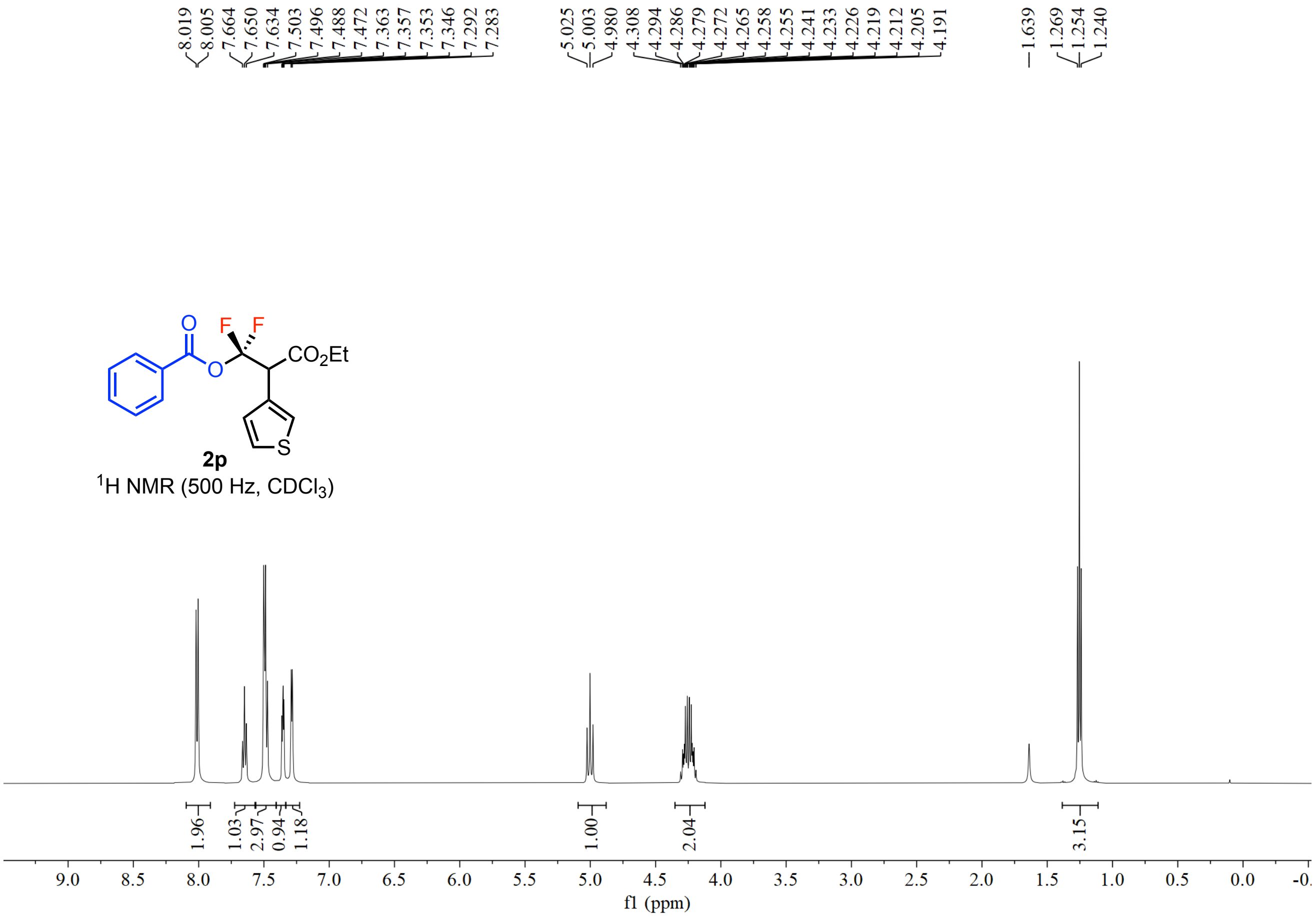
8.019
8.005
7.664
7.650
7.634
7.503
7.496
7.488
7.472
7.363
7.357
7.353
7.346
7.292
7.283
5.025
5.003
4.980
4.308
4.294
4.286
4.279
4.272
4.265
4.258
4.255
4.241
4.233
4.226
4.219
4.212
4.205
4.191
1.639
1.269
1.254
1.240
2p
1H NMR (500 Hz, CDCl3)
1.96
1.03
2.97
0.94
1.18
1.00
2.04
3.15
f1 (ppm)

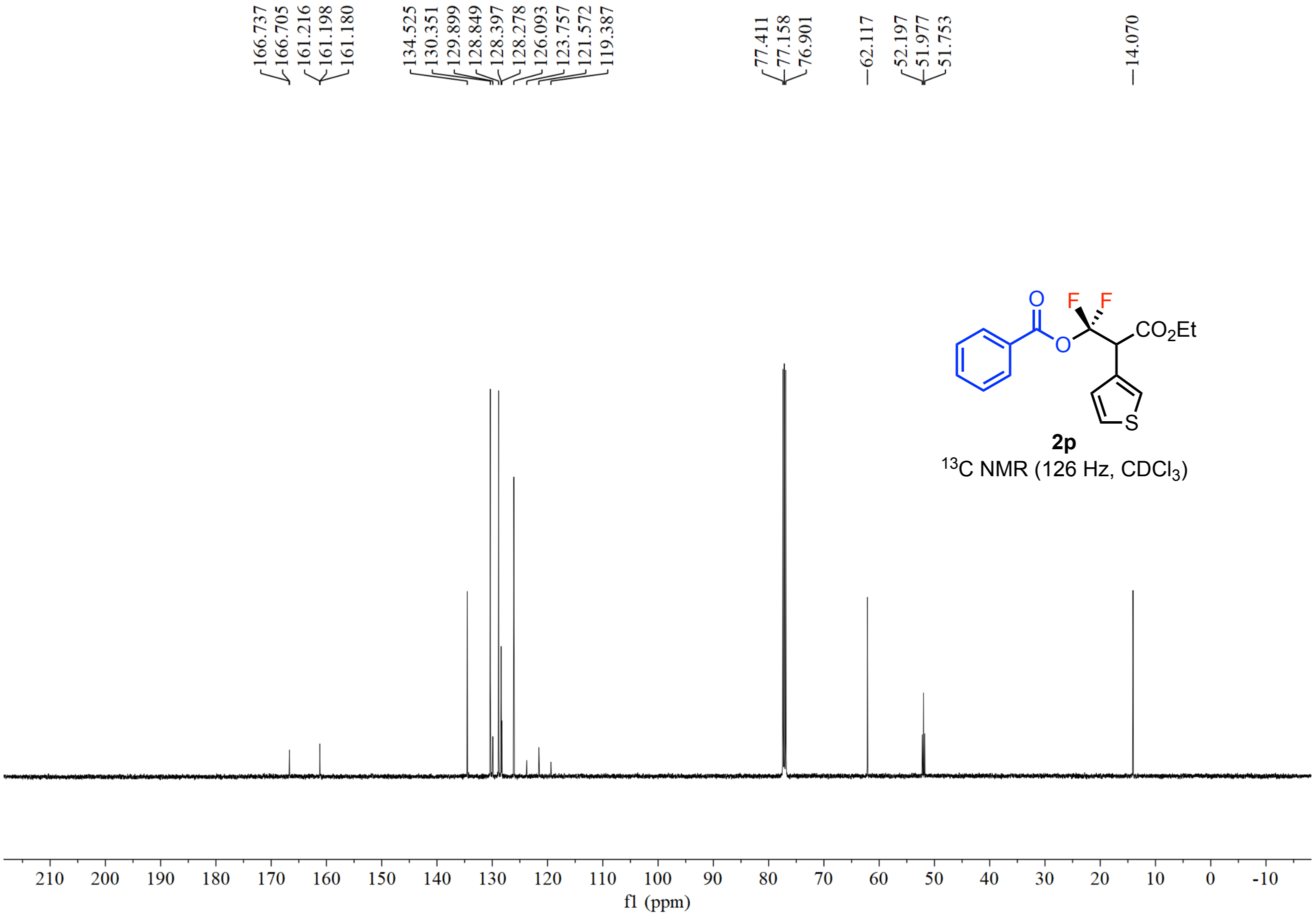

166.737
166.705
161.216
161.198
161.180
134.525
130.351
129.899
128.849
128.397
128.278
126.093
123.757
121.572
119.387
77.411
77.158
76.901
62.117
52.197
51.977
51.753
14.070
O
F
F
O
CO2Et
S
2p
13C NMR (126 Hz, CDCl3)
210 200 190 180 170 160 150 140 130 120 110 100 90 80 70 60 50 40 30 20 10 0 -10
f1 (ppm)

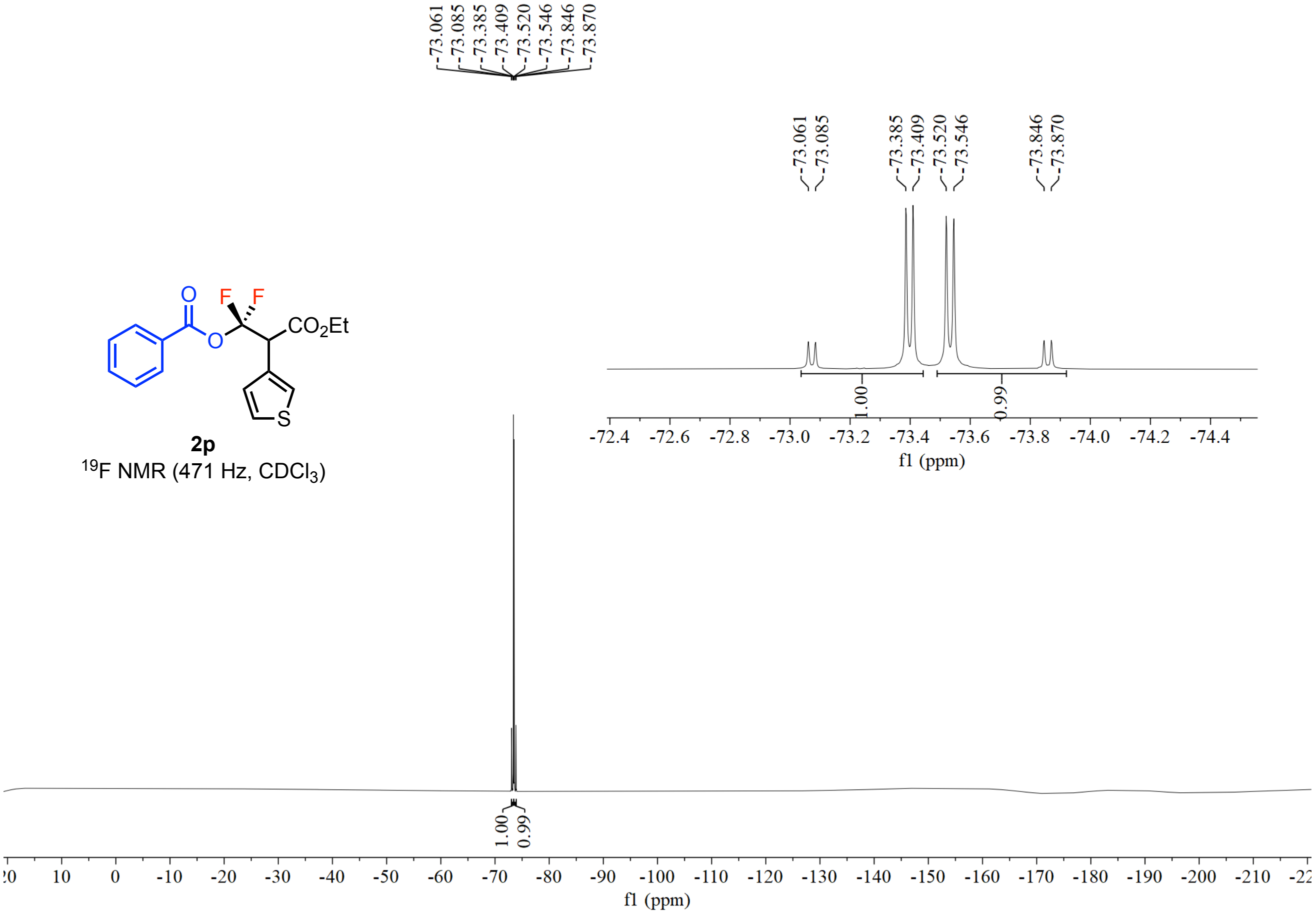

-73.061
-73.085
-73.385
-73.409
-73.520
-73.546
-73.846
-73.870
1.00
0.99
-72.4 -72.6 -72.8 -73.0 -73.2 -73.4 -73.6 -73.8 -74.0 -74.2 -74.4
f1 (ppm)
O
F
F
O
CO2Et
S
2p
19F NMR (471 Hz, CDCl3)
10 0 -10 -20 -30 -40 -50 -60 -70 -80 -90 -100 -110 -120 -130 -140 -150 -160 -170 -180 -190 -200 -210
f1 (ppm)

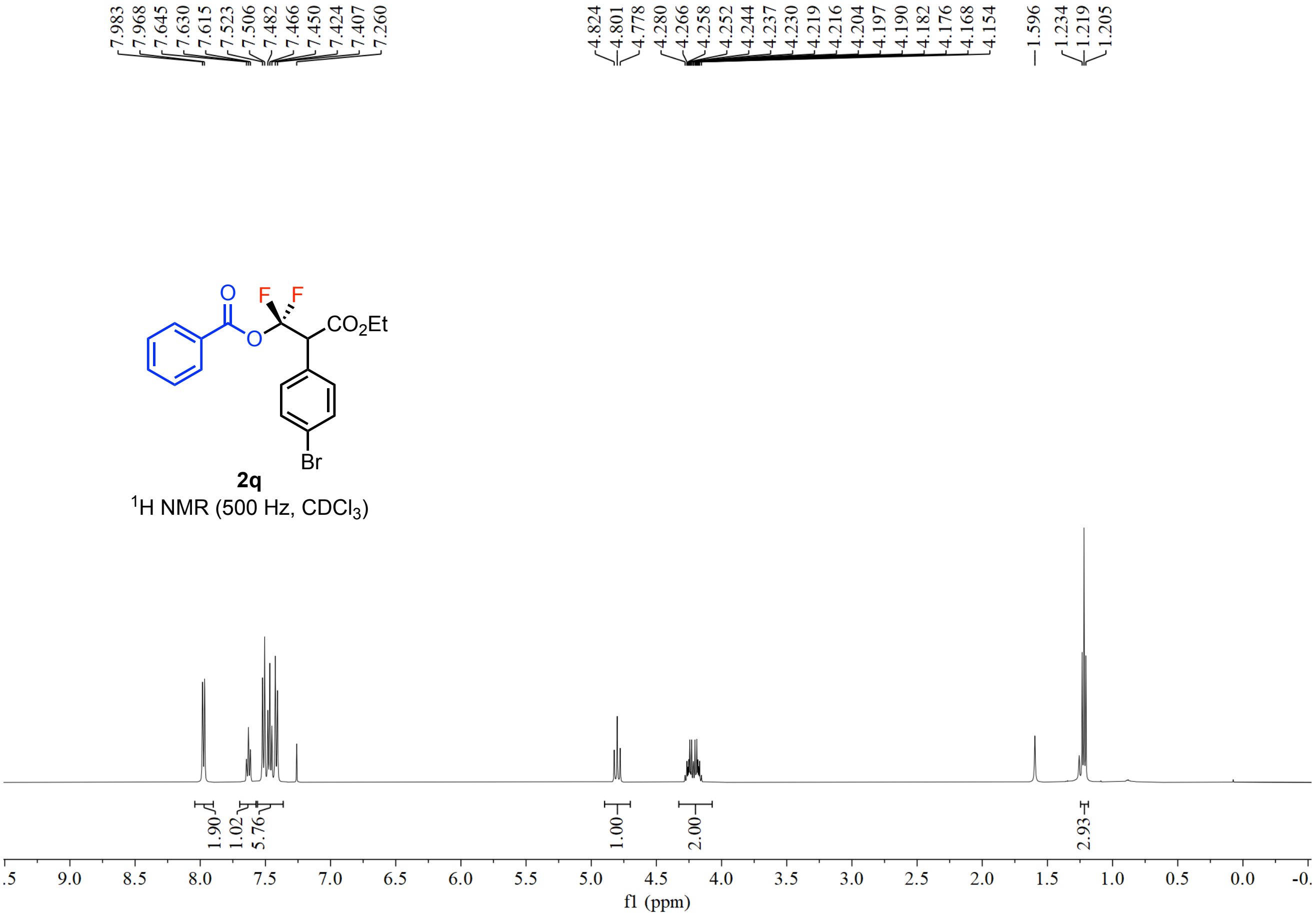
7.983
7.968
7.645
7.630
7.615
7.523
7.506
7.482
7.466
7.450
7.424
7.407
7.260
4.824
4.801
4.778
4.280
4.266
4.258
4.252
4.244
4.237
4.230
4.219
4.216
4.204
4.197
4.190
4.182
4.176
4.168
4.154
1.596
1.234
1.219
1.205
CO2Et
Br
2q
1H NMR (500 Hz, CDCl3)
1.90
1.02
5.76
1.00
2.00
2.93
f1 (ppm)

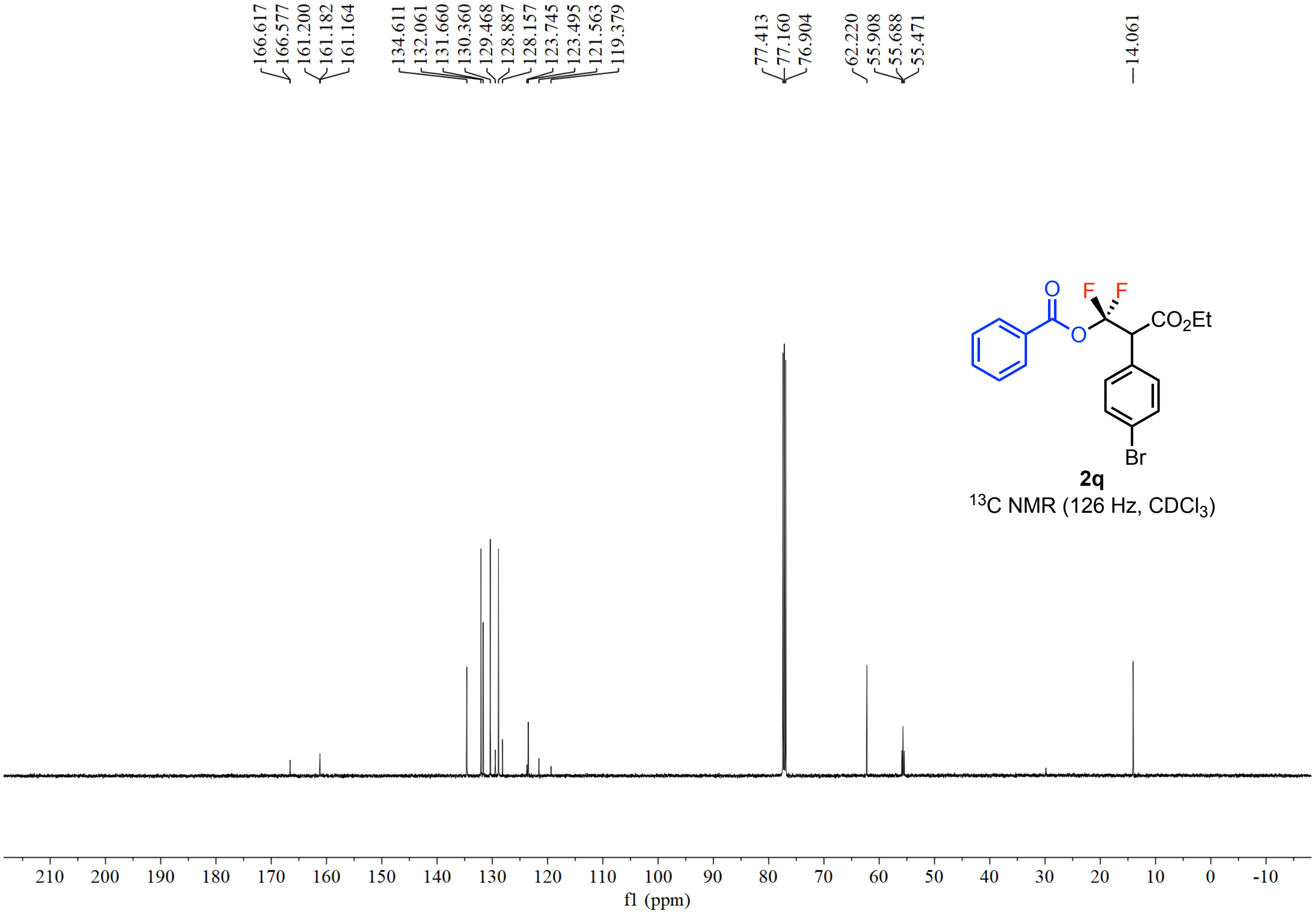
166.617
166.577
161.200
161.182
161.164
134.611
132.061
131.660
130.360
129.468
128.887
128.157
123.745
123.495
121.563
119.379
77.413
77.160
76.904
62.220
55.908
55.688
55.471
14.061
CO2Et
Br
2q
13C NMR (126 Hz, CDCl3)
f1 (ppm)

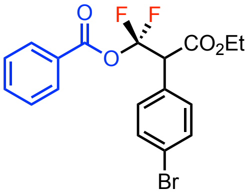

2q

^{19}F NMR (471 Hz, $CDCl_3$)

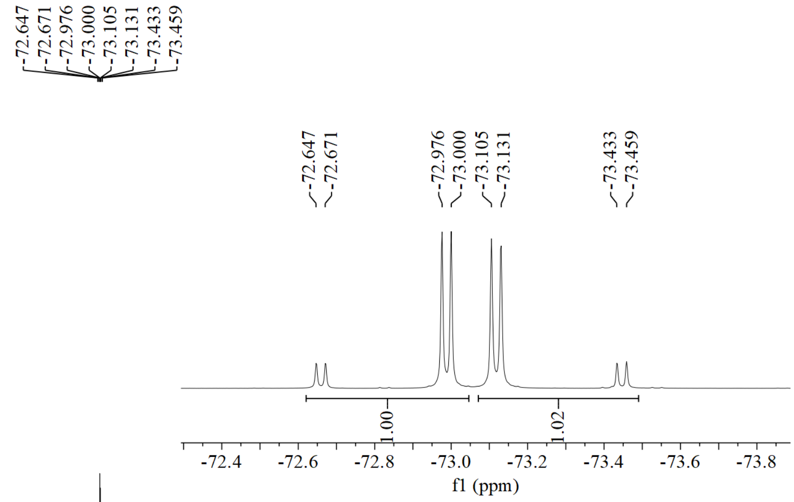

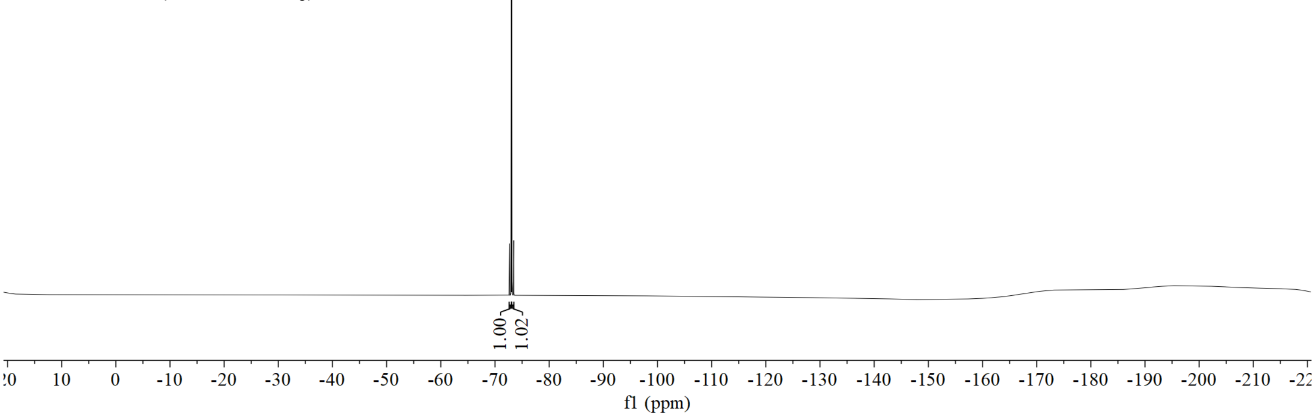

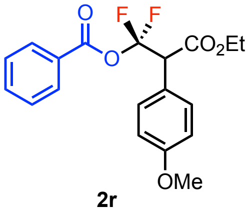

2r

^{1}H NMR (500 Hz, $CDCl_3$)

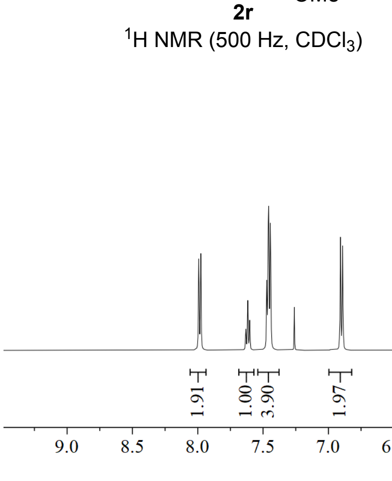
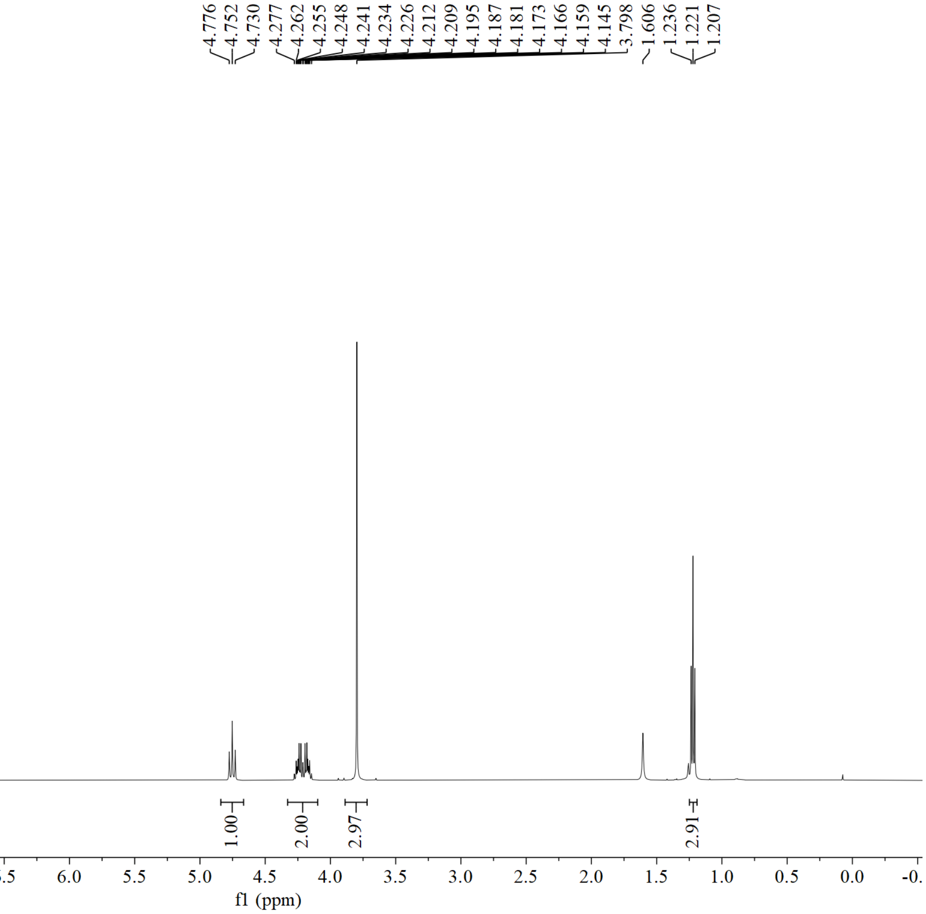

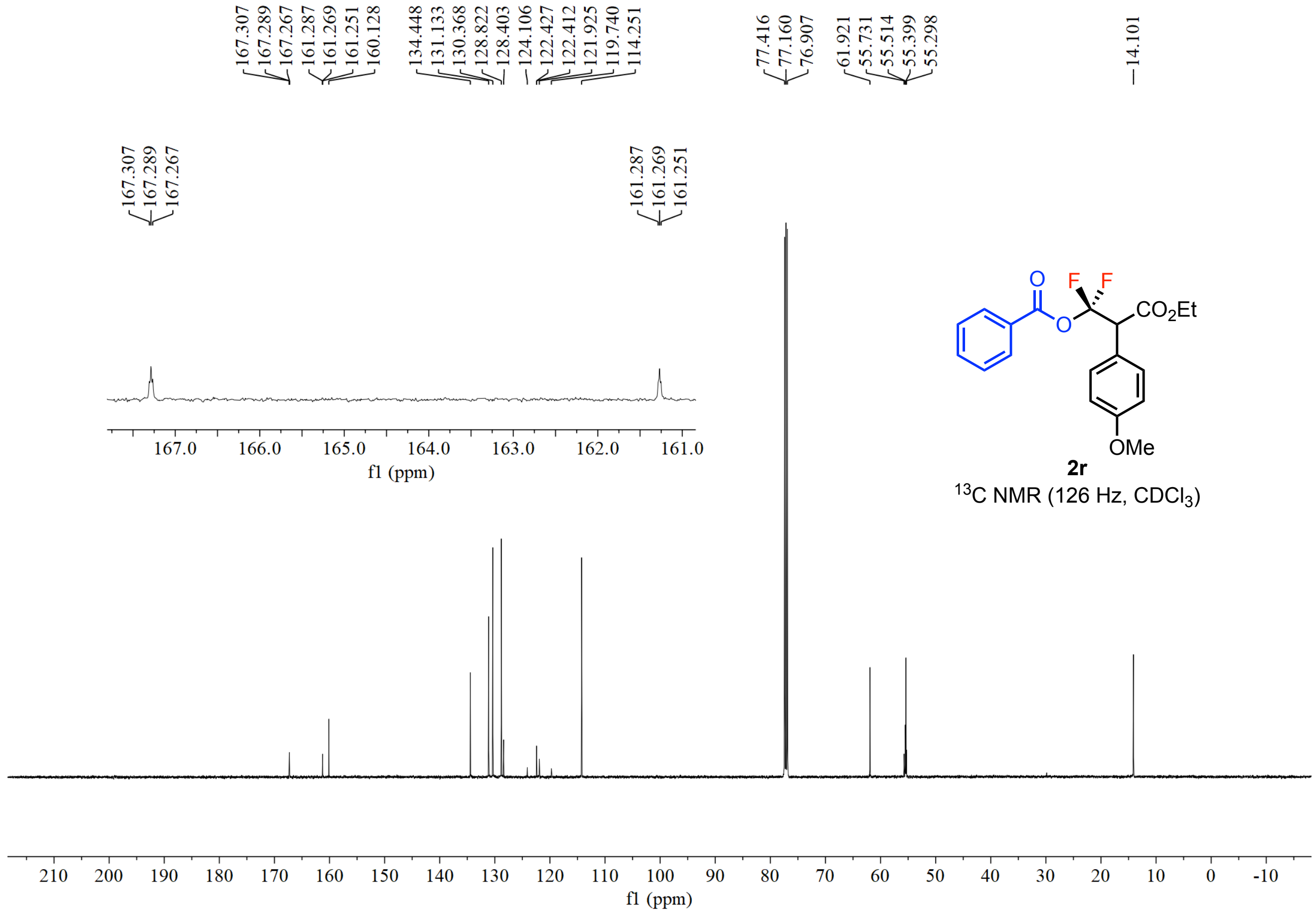
167.307
167.289
167.267
161.287
161.269
161.251
160.128
134.448
131.133
130.368
128.822
128.403
124.106
122.427
122.412
121.925
119.740
114.251
77.416
77.160
76.907
61.921
55.731
55.514
55.399
55.298
14.101
167.307
167.289
167.267
161.287
161.269
161.251
167.0
166.0
165.0
164.0
163.0
162.0
161.0
f1 (ppm)
O
F
F
O
CO_2Et
OMe
2r
^{13}C NMR (126 Hz, $CDCl_3$)
210
200
190
180
170
160
150
140
130
120
110
100
90
80
70
60
50
40
30
20
10
0
-10
f1 (ppm)

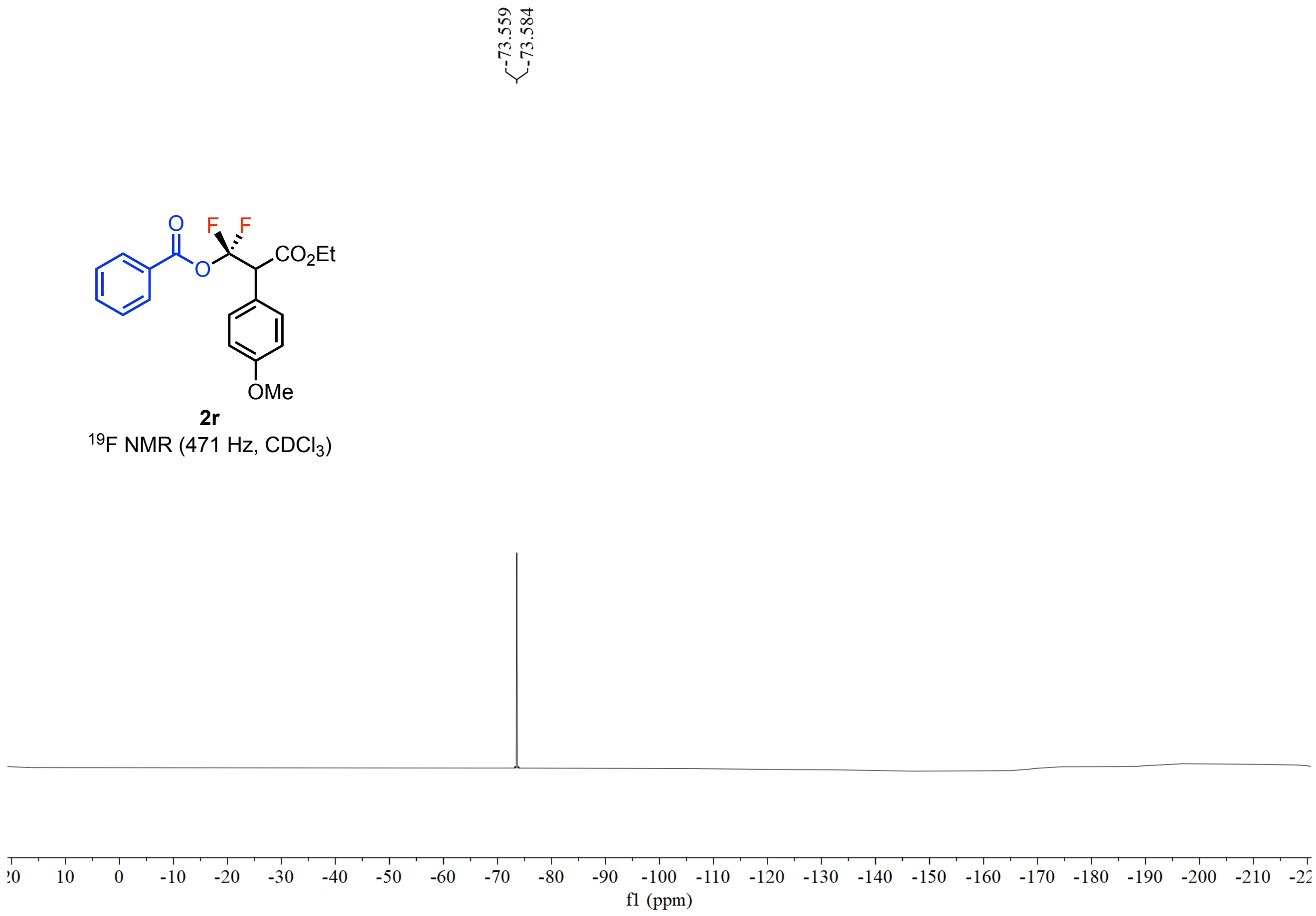
-73.559
-73.584
O
F
F
O
CO_2Et
OMe
2r
^{19}F NMR (471 Hz, $CDCl_3$)
10
0
-10
-20
-30
-40
-50
-60
-70
-80
-90
-100
-110
-120
-130
-140
-150
-160
-170
-180
-190
-200
-210
f1 (ppm)

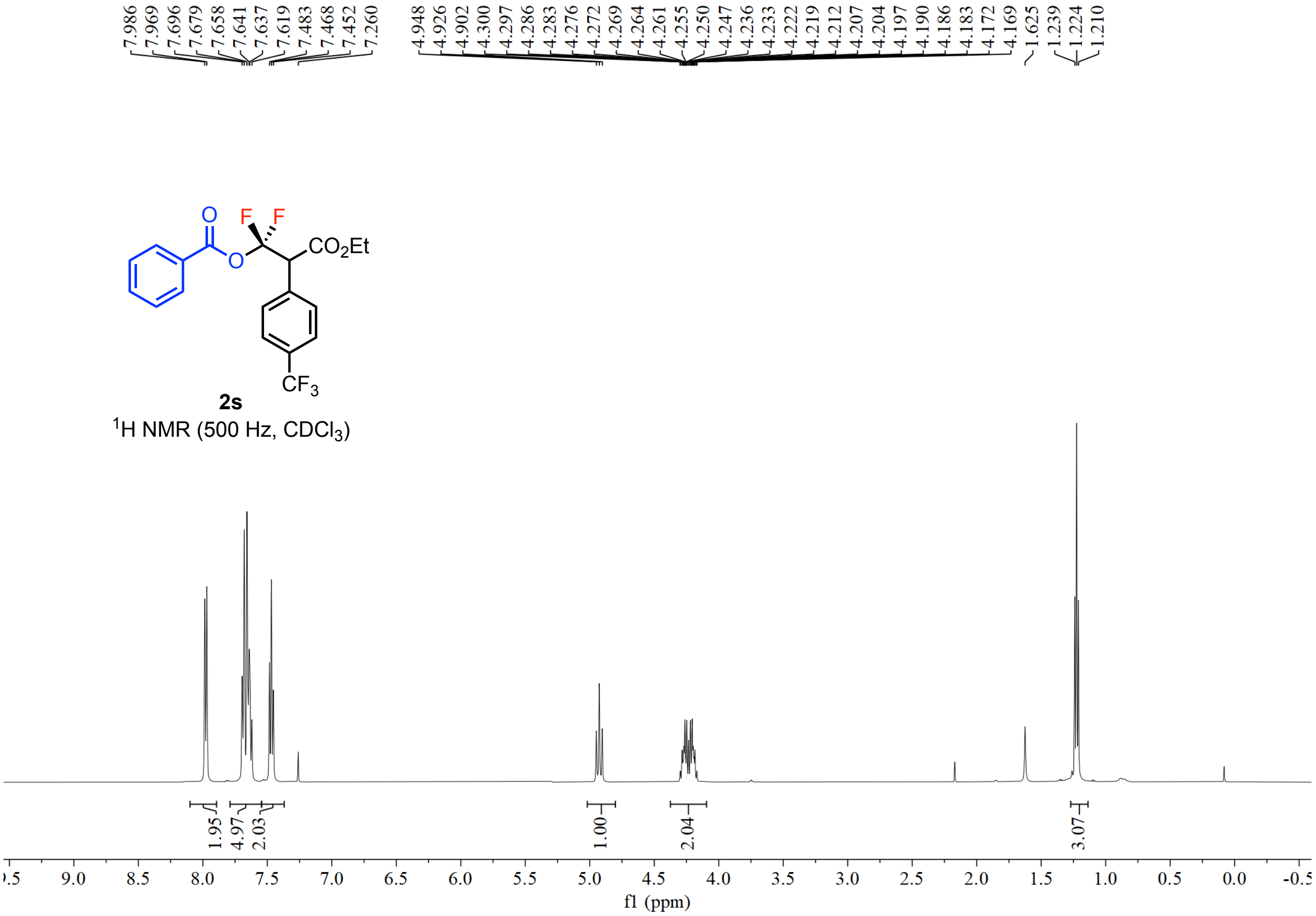
7.986
7.969
7.696
7.679
7.658
7.641
7.637
7.619
7.483
7.468
7.452
7.260
4.948
4.926
4.902
4.300
4.297
4.286
4.283
4.276
4.272
4.269
4.264
4.261
4.255
4.250
4.247
4.236
4.233
4.222
4.219
4.212
4.207
4.204
4.197
4.190
4.186
4.183
4.172
4.169
1.625
1.239
1.224
1.210
F
F
O
O
CO_2Et
CF_3
2s
^{1}H NMR (500 Hz, $CDCl_3$)
1.95
4.97
2.03
1.00
2.04
3.07
f1 (ppm)

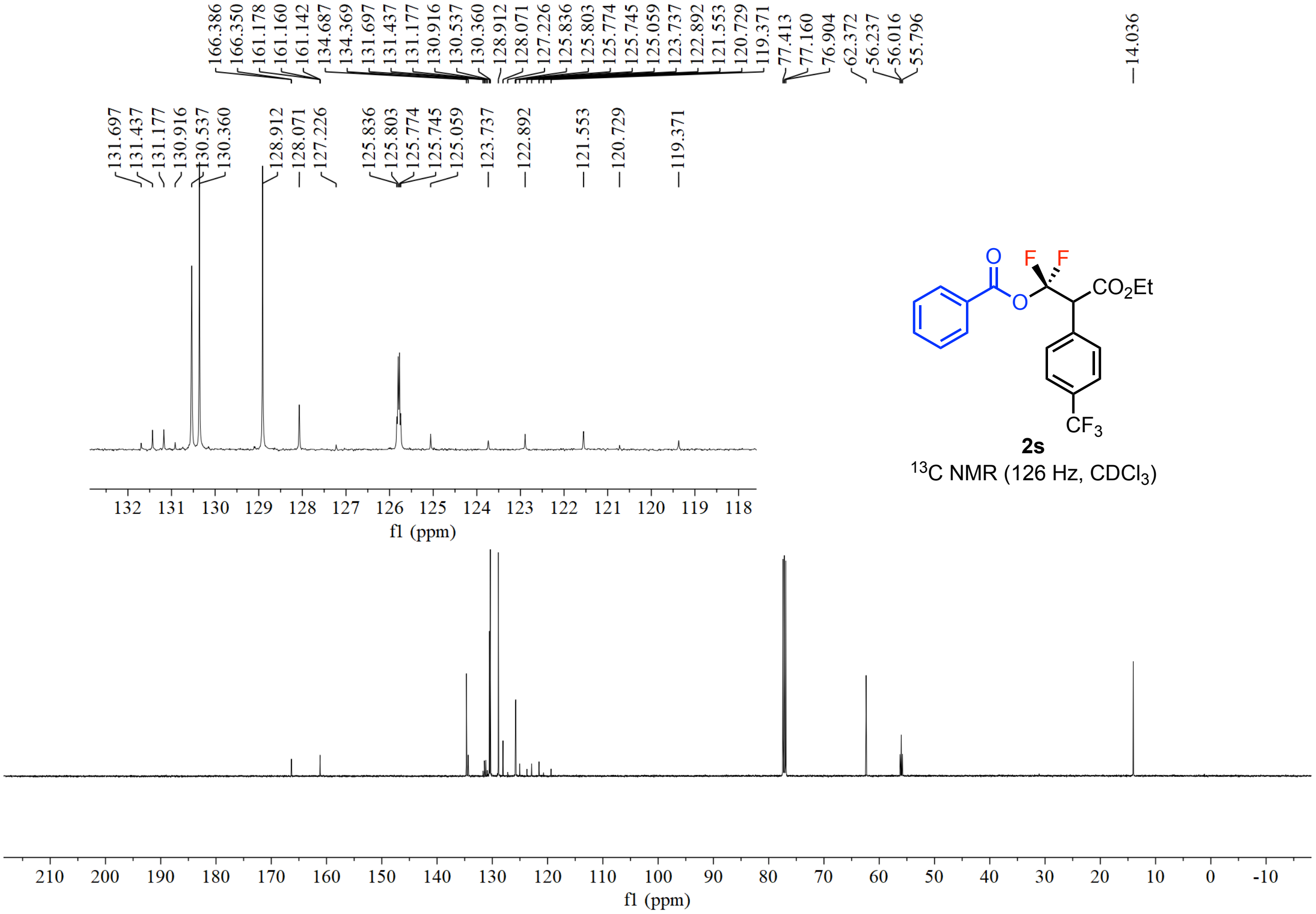
166.386
166.350
161.178
161.160
161.142
134.687
134.369
131.697
131.437
131.177
130.916
130.537
130.360
128.912
128.071
127.226
125.836
125.803
125.774
125.745
125.059
123.737
122.892
121.553
120.729
119.371
77.413
77.160
76.904
62.372
56.237
56.016
55.796
14.036
F
F
O
O
CO_2Et
CF_3
2s
^{13}C NMR (126 Hz, $CDCl_3$)
f1 (ppm)

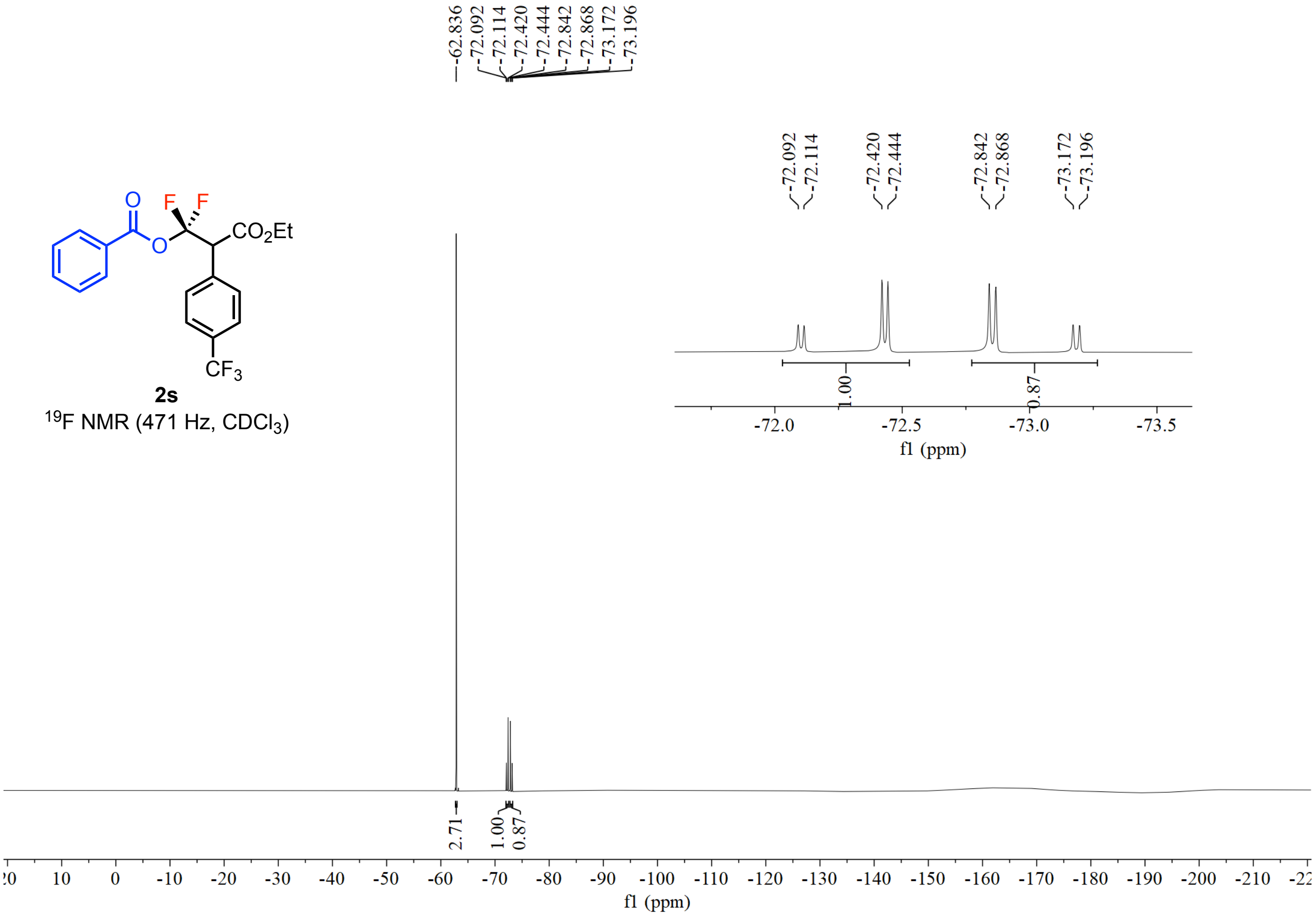
-62.836
-72.092
-72.114
-72.420
-72.444
-72.842
-72.868
-73.172
-73.196
CO$_2$Et
CF$_3$
2s
^{19}F NMR (471 Hz, CDCl$_3$)
1.00
0.87
-72.0
-72.5
-73.0
-73.5
f1 (ppm)
2.71
1.00
0.87
f1 (ppm)

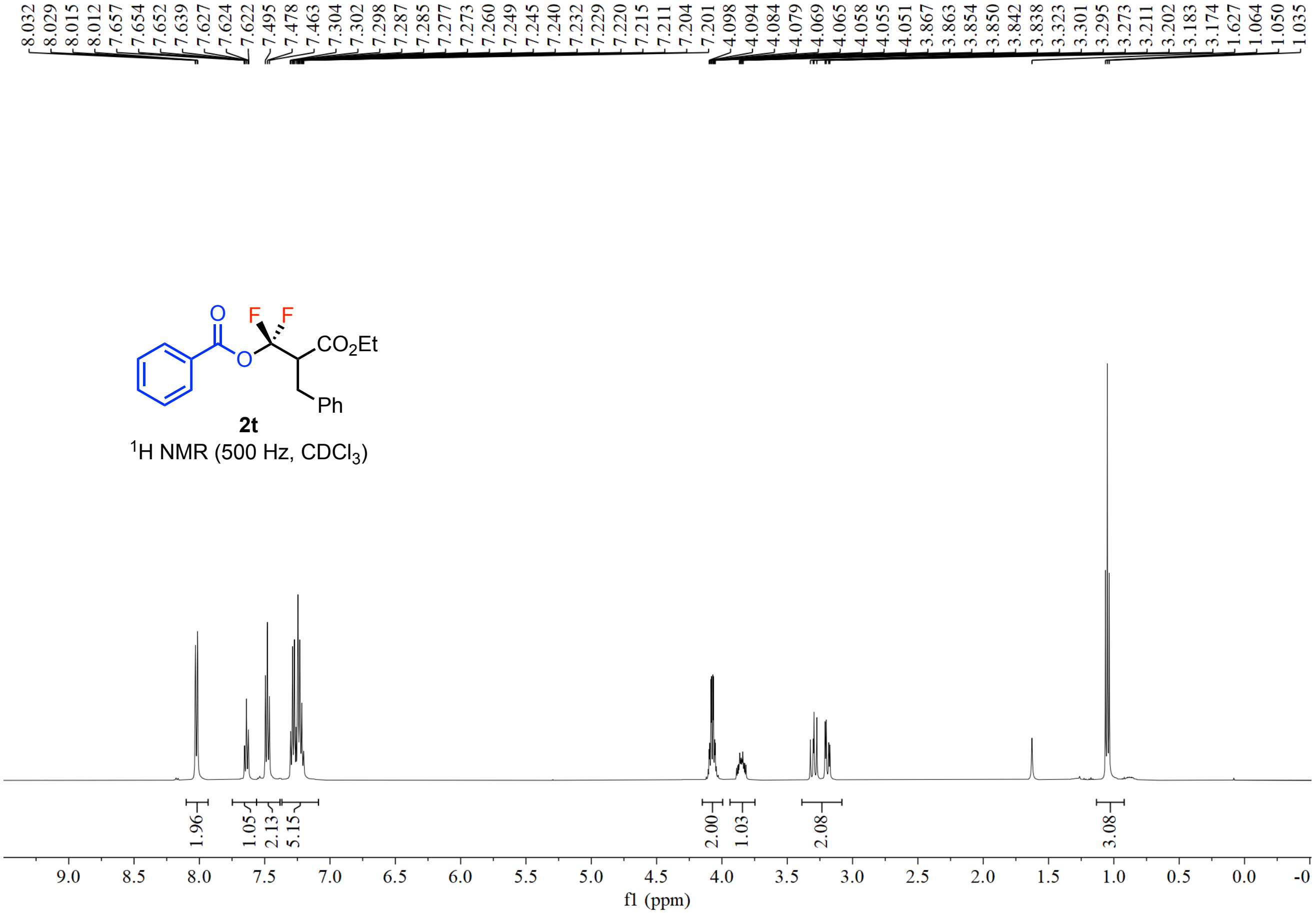
8.032
8.029
8.015
8.012
7.657
7.654
7.652
7.639
7.627
7.624
7.622
7.495
7.478
7.463
7.304
7.302
7.298
7.287
7.285
7.277
7.273
7.260
7.249
7.245
7.240
7.232
7.229
7.220
7.215
7.211
7.204
7.201
4.098
4.094
4.084
4.079
4.069
4.065
4.058
4.055
4.051
3.867
3.863
3.854
3.850
3.842
3.838
3.323
3.301
3.295
3.273
3.211
3.202
3.183
3.174
1.627
1.064
1.050
1.035
CO$_2$Et
Ph
2t
^{1}H NMR (500 Hz, CDCl$_3$)
1.96
1.05
2.13
5.15
2.00
1.03
2.08
3.08
f1 (ppm)

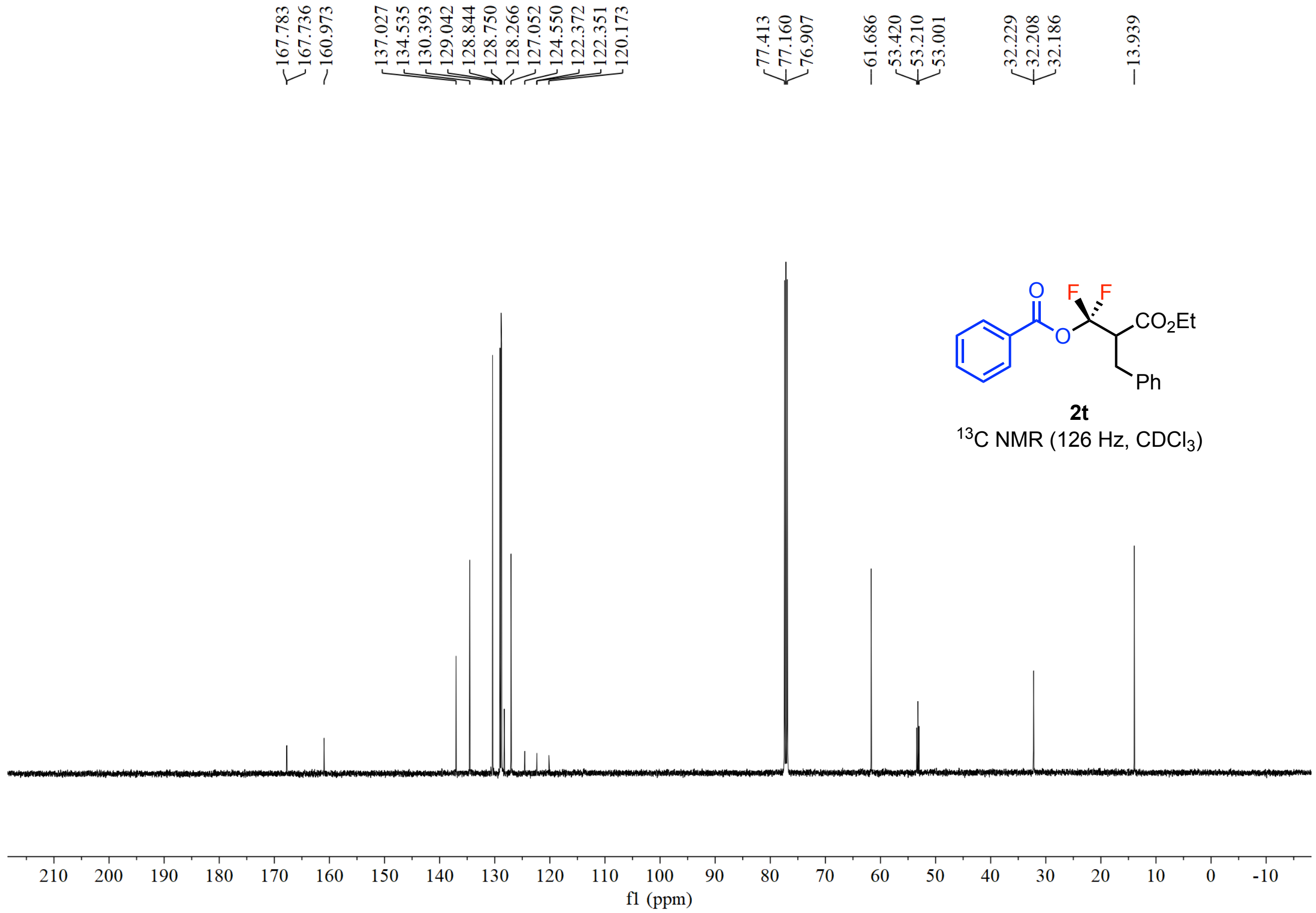
167.783
167.736
160.973
137.027
134.535
130.393
129.042
128.844
128.750
128.266
127.052
124.550
122.372
122.351
120.173
77.413
77.160
76.907
61.686
53.420
53.210
53.001
32.229
32.208
32.186
13.939
O
O
F
F
CO_2Et
Ph
2t
^{13}C NMR (126 Hz, $CDCl_3$)
210 200 190 180 170 160 150 140 130 120 110 100 90 80 70 60 50 40 30 20 10 0 -10
f1 (ppm)

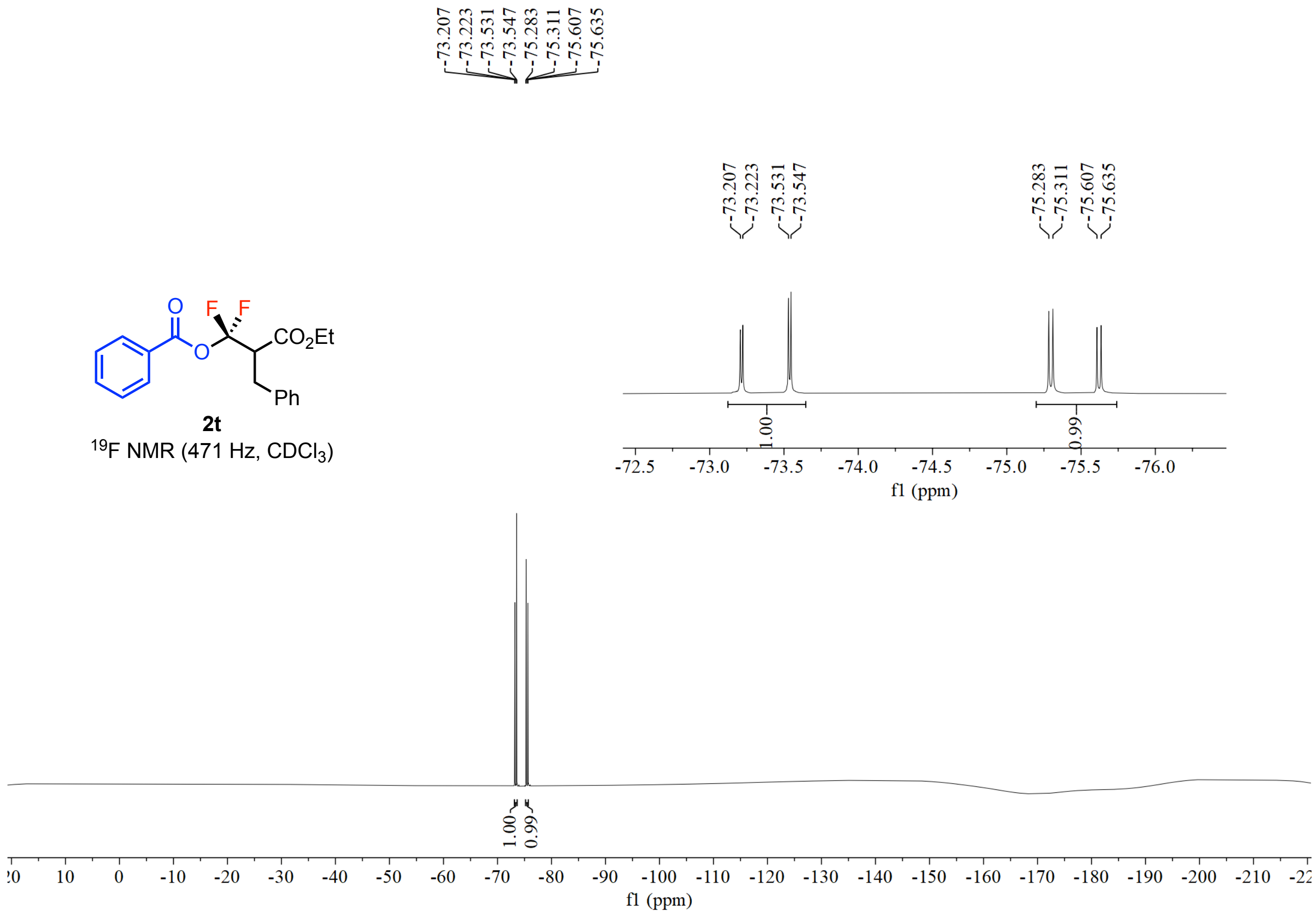
-73.207
-73.223
-73.531
-73.547
-75.283
-75.311
-75.607
-75.635
O
O
F
F
CO_2Et
Ph
2t
^{19}F NMR (471 Hz, $CDCl_3$)
1.00
0.99
-72.5 -73.0 -73.5 -74.0 -74.5 -75.0 -75.5 -76.0
f1 (ppm)
20 10 0 -10 -20 -30 -40 -50 -60 -70 -80 -90 -100 -110 -120 -130 -140 -150 -160 -170 -180 -190 -200 -210 -22
f1 (ppm)

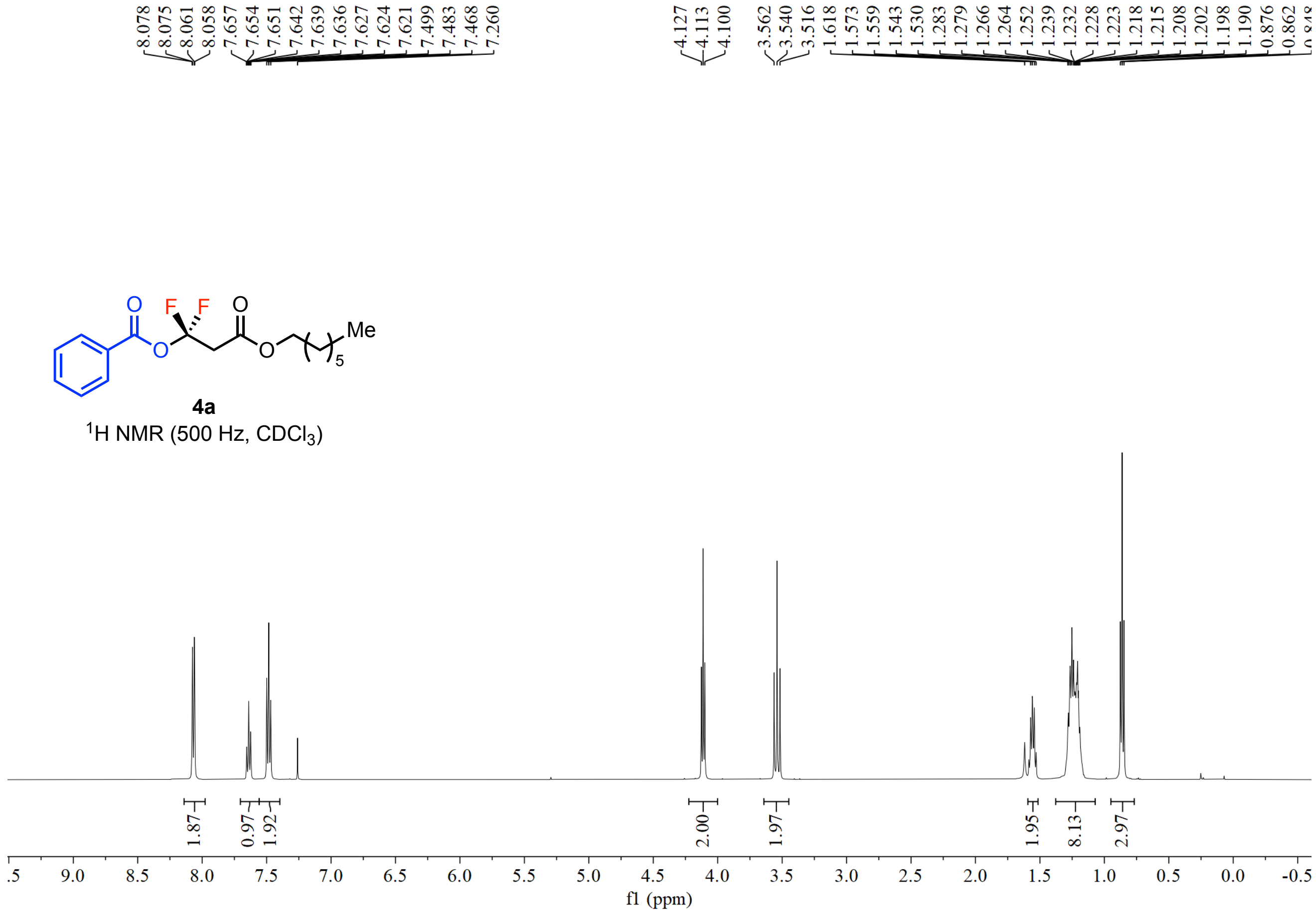
8.078
8.075
8.061
8.058
7.657
7.654
7.651
7.642
7.639
7.636
7.627
7.624
7.621
7.499
7.483
7.468
7.260
4.127
4.113
4.100
3.562
3.540
3.516
1.618
1.573
1.559
1.543
1.530
1.283
1.279
1.266
1.264
1.252
1.239
1.232
1.228
1.223
1.218
1.215
1.208
1.202
1.198
1.190
0.876
0.862
O
F
F
O
O
Me
5
4a
1H NMR (500 Hz, CDCl3)
1.87
0.97
1.92
2.00
1.97
1.95
8.13
2.97
f1 (ppm)

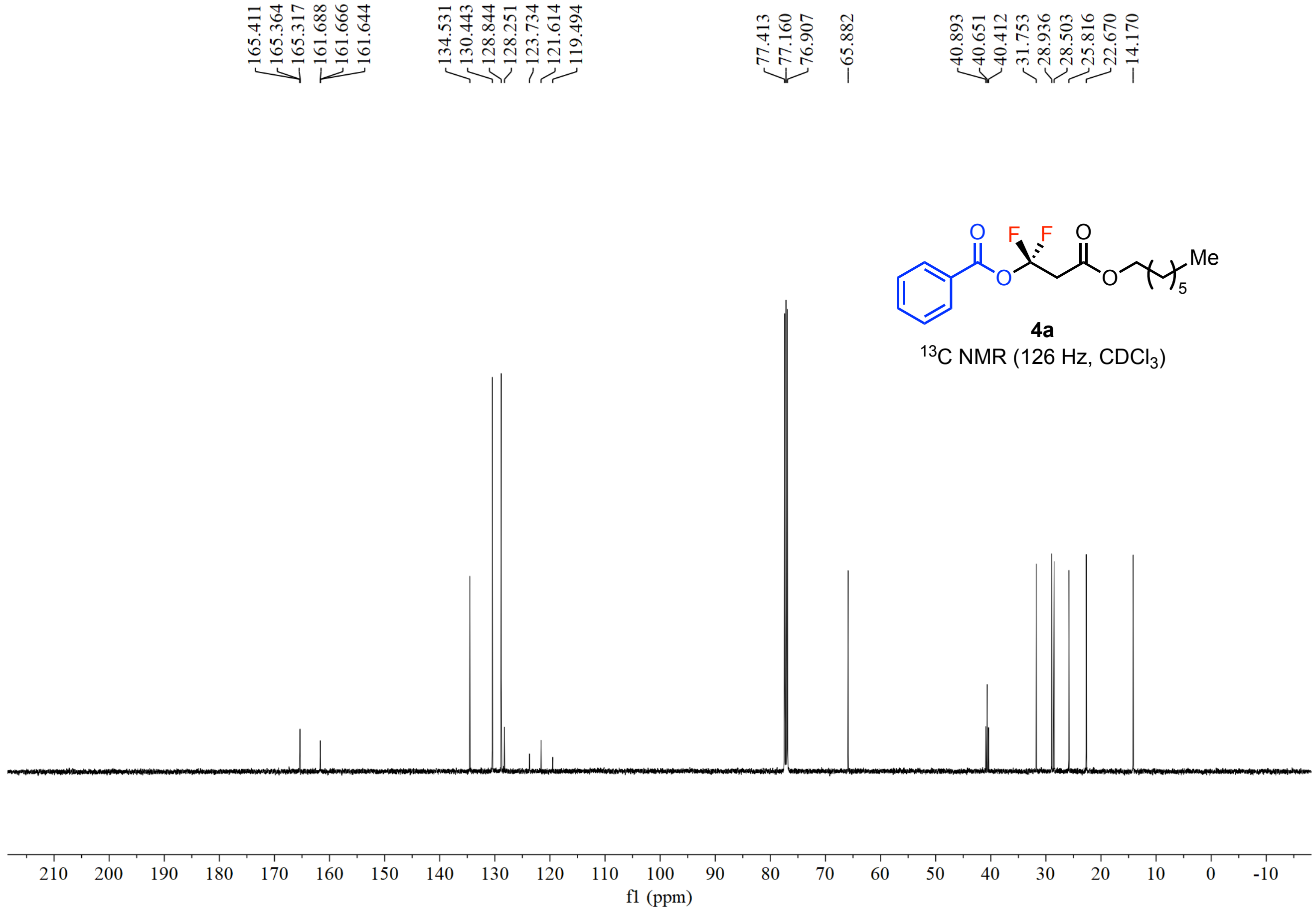
165.411
165.364
165.317
161.688
161.666
161.644
134.531
130.443
128.844
128.251
123.734
121.614
119.494
77.413
77.160
76.907
65.882
40.893
40.651
40.412
31.753
28.936
28.503
25.816
22.670
14.170
O
F
F
O
O
Me
5
4a
13C NMR (126 Hz, CDCl3)
f1 (ppm)

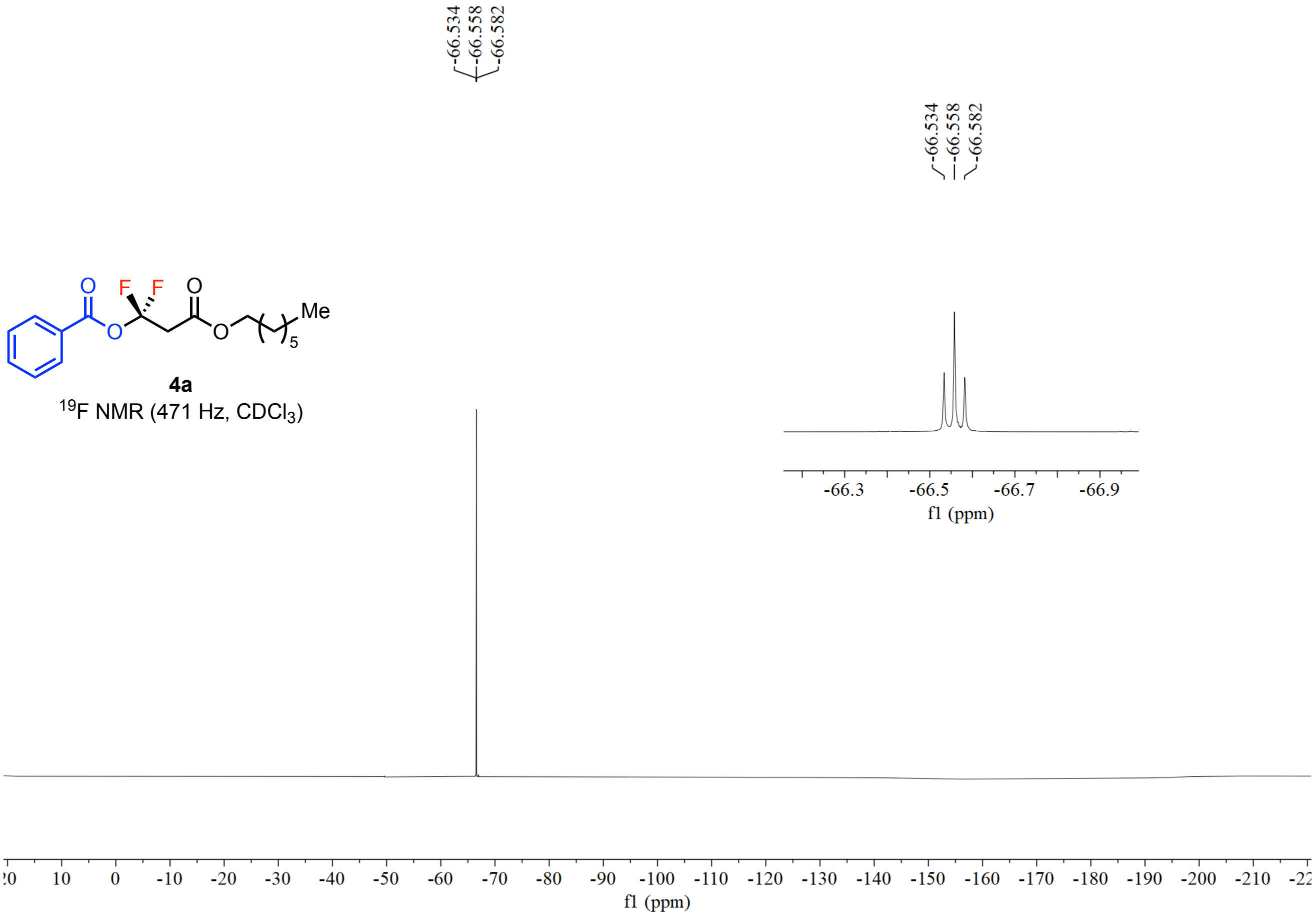

4a

^{19}F NMR (471 Hz, $CDCl_3$)

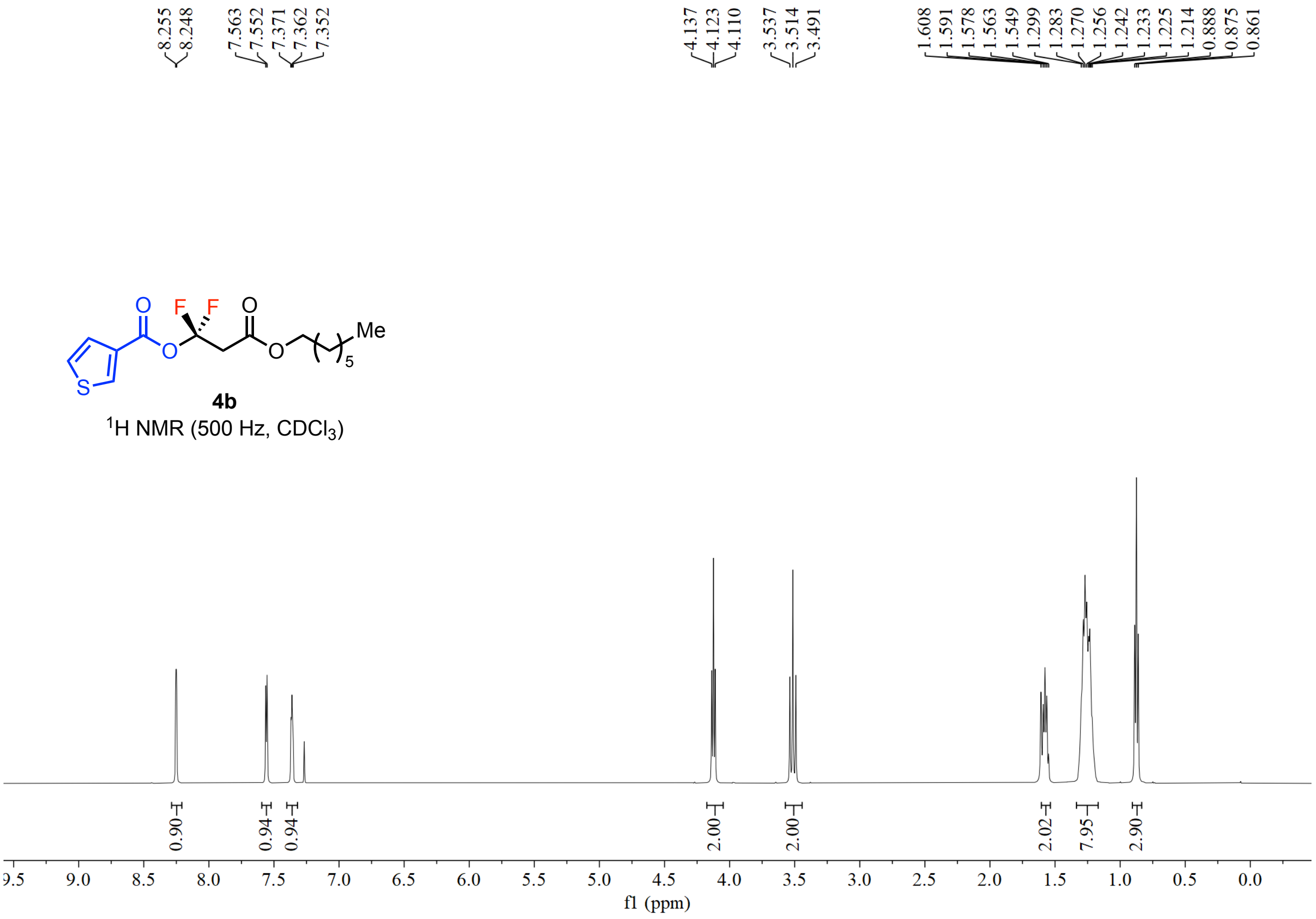

4b

^{1}H NMR (500 Hz, $CDCl_3$)

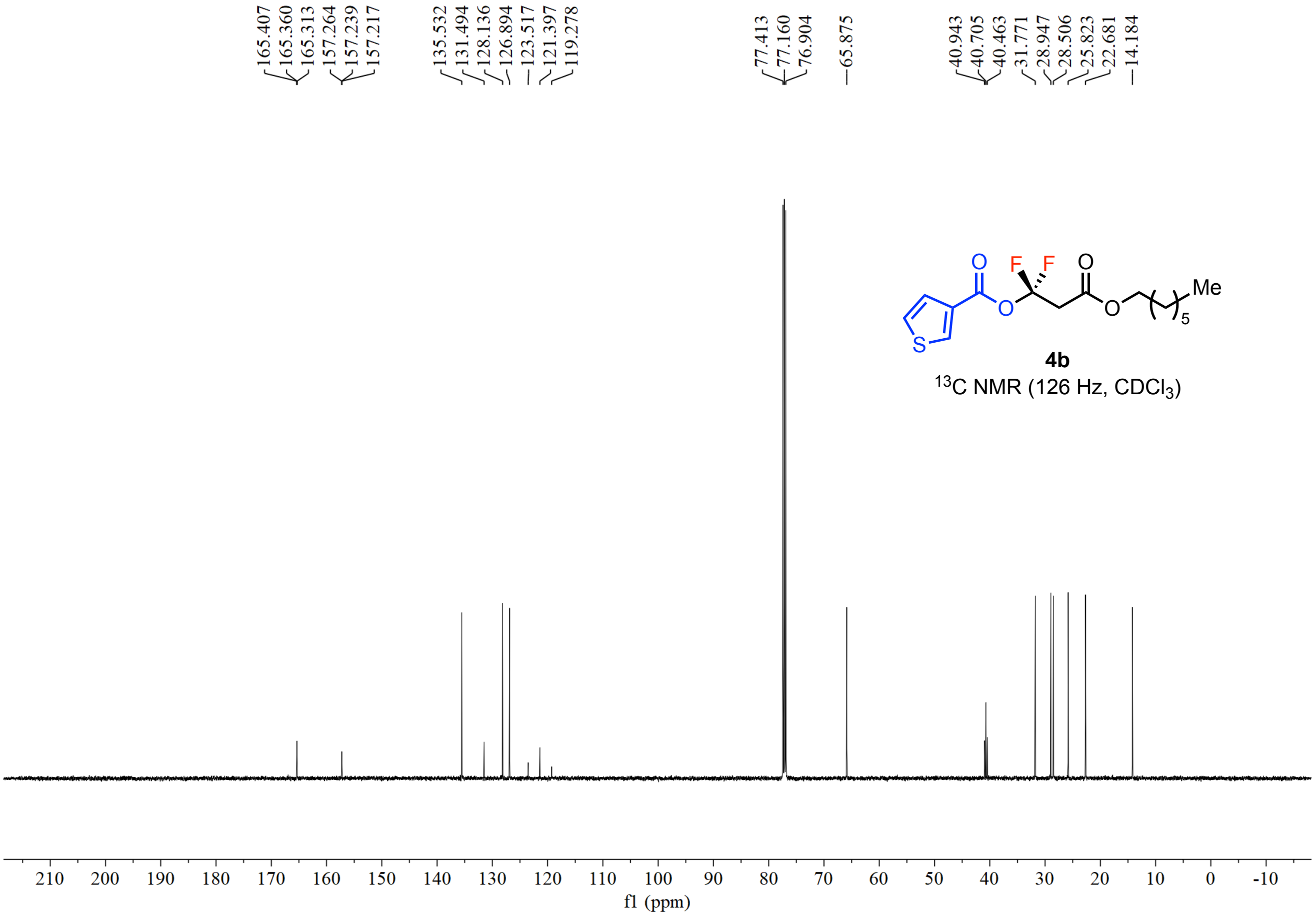
165.407
165.360
165.313
157.264
157.239
157.217
135.532
131.494
128.136
126.894
123.517
121.397
119.278
77.413
77.160
76.904
65.875
40.943
40.705
40.463
31.771
28.947
28.506
25.823
22.681
14.184
O
F F
O
Me
S
O
O
5
4b
13C NMR (126 Hz, CDCl3)
210 200 190 180 170 160 150 140 130 120 110 100 90 80 70 60 50 40 30 20 10 0 -10
f1 (ppm)

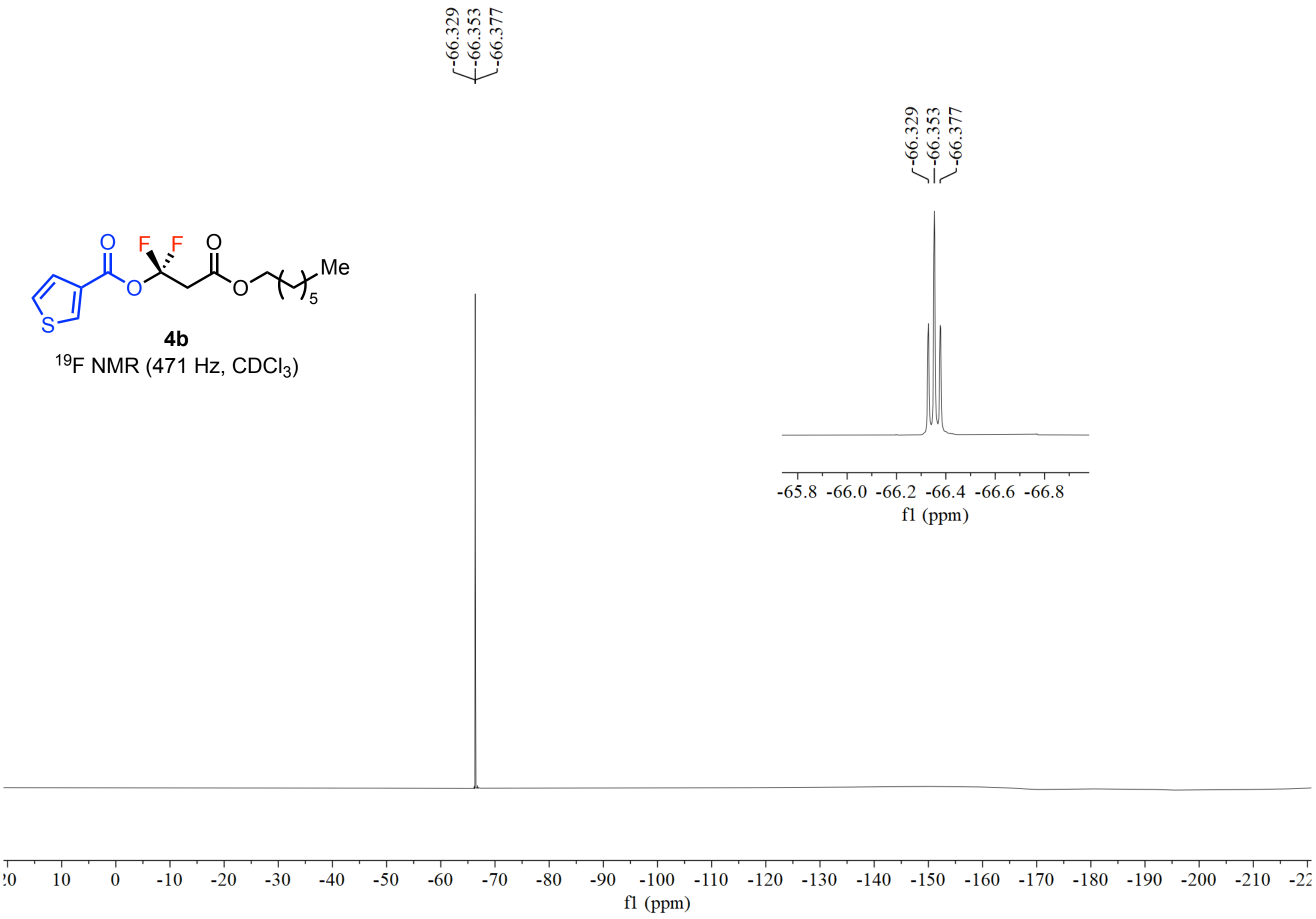
-66.329
-66.353
-66.377
O
F F
O
Me
S
O
O
5
4b
19F NMR (471 Hz, CDCl3)
-65.8 -66.0 -66.2 -66.4 -66.6 -66.8
f1 (ppm)
10 0 -10 -20 -30 -40 -50 -60 -70 -80 -90 -100 -110 -120 -130 -140 -150 -160 -170 -180 -190 -200 -210
f1 (ppm)

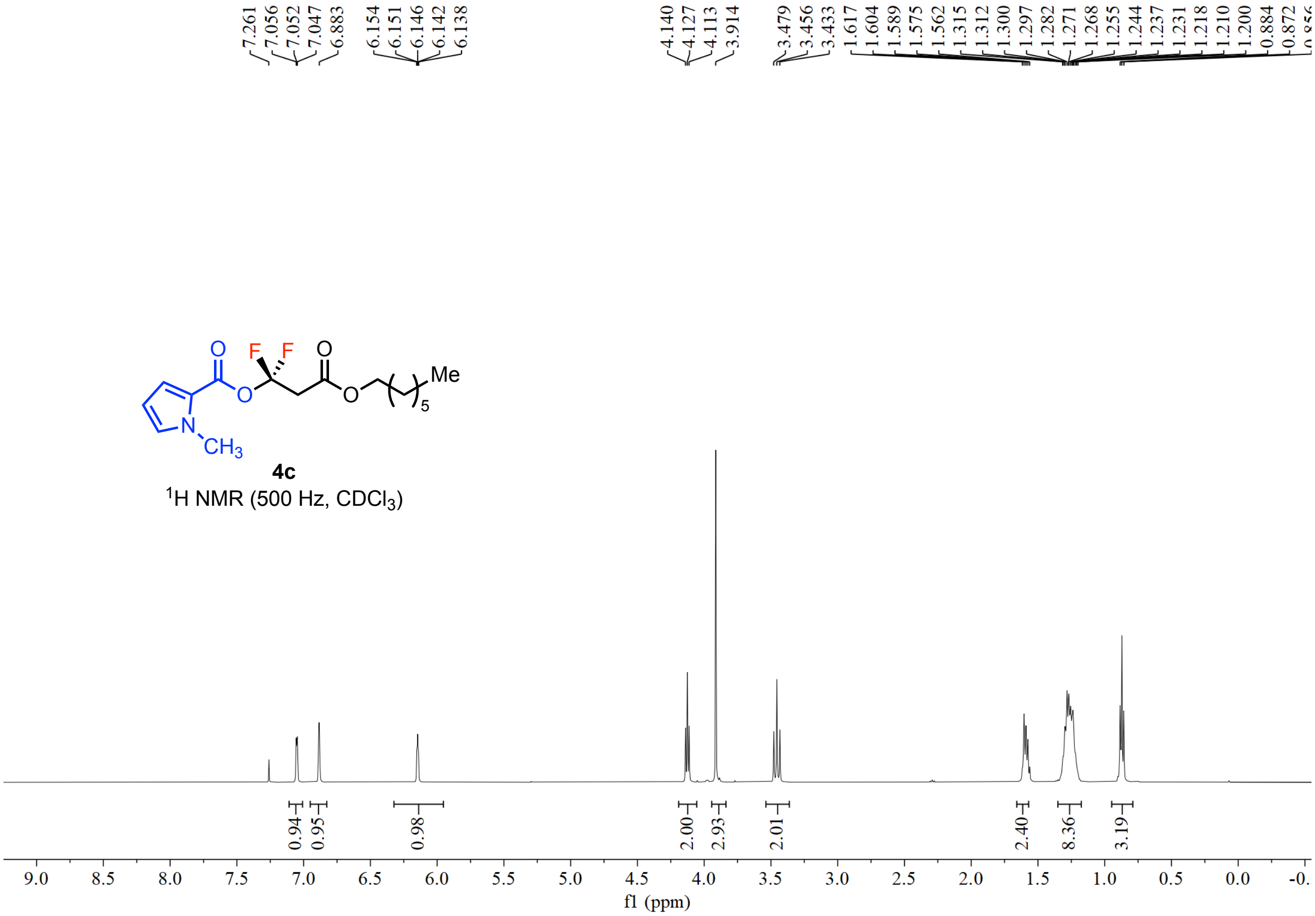
7.261
7.056
7.052
7.047
6.883
6.154
6.151
6.146
6.142
6.138
4.140
4.127
4.113
3.914
3.479
3.456
3.433
1.617
1.604
1.589
1.575
1.562
1.315
1.312
1.300
1.297
1.282
1.271
1.268
1.255
1.244
1.237
1.231
1.218
1.210
1.200
0.884
0.872
O
F
F
O
O
O
N
CH3
Me
5
4c
1H NMR (500 Hz, CDCl3)
0.94
0.95
0.98
2.00
2.93
2.01
2.40
8.36
3.19
f1 (ppm)

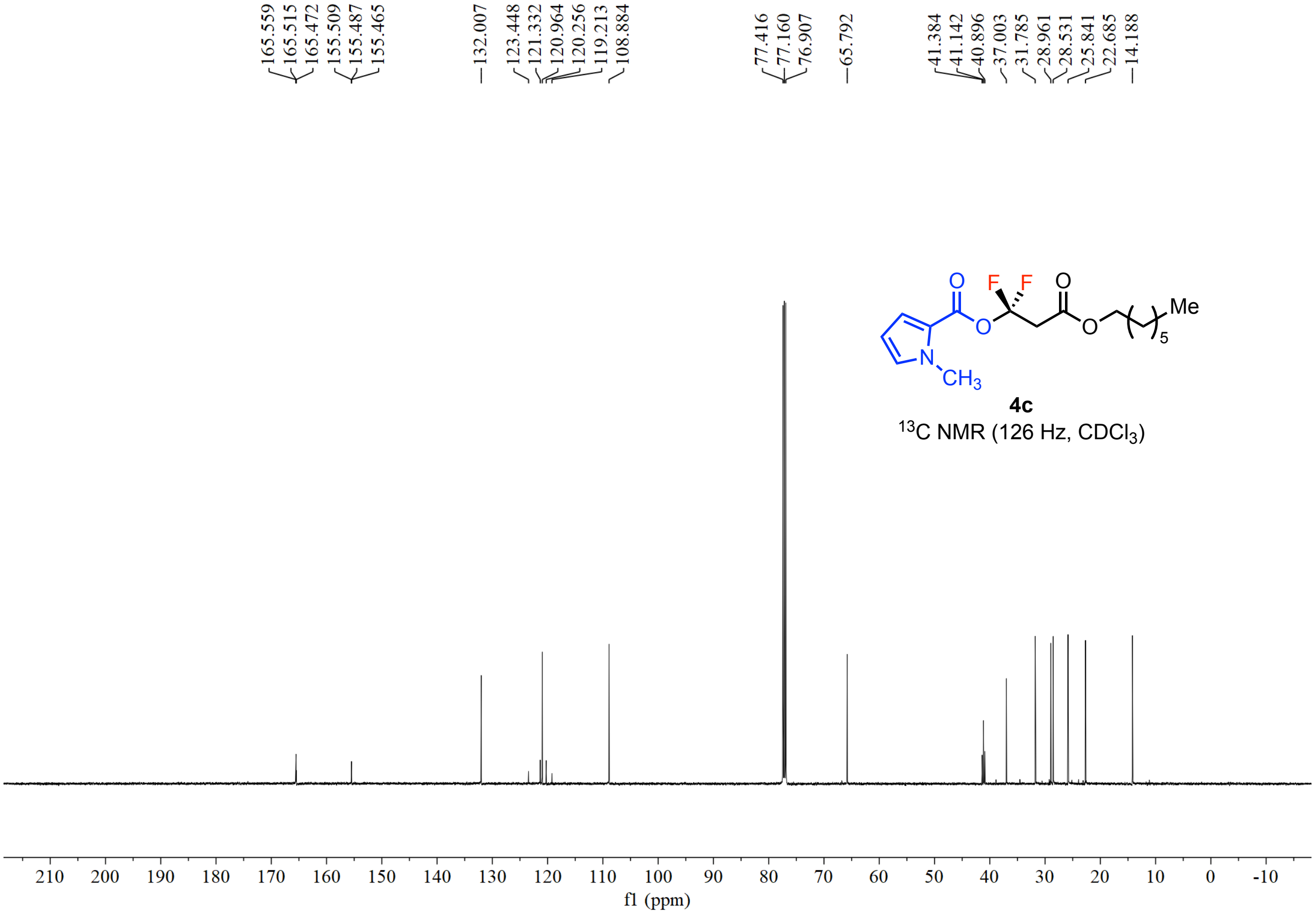
165.559
165.515
165.472
155.509
155.487
155.465
132.007
123.448
121.332
120.964
120.256
119.213
108.884
77.416
77.160
76.907
65.792
41.384
41.142
40.896
37.003
31.785
28.961
28.531
25.841
22.685
14.188
O
F
F
O
O
O
N
CH3
Me
5
4c
13C NMR (126 Hz, CDCl3)
f1 (ppm)

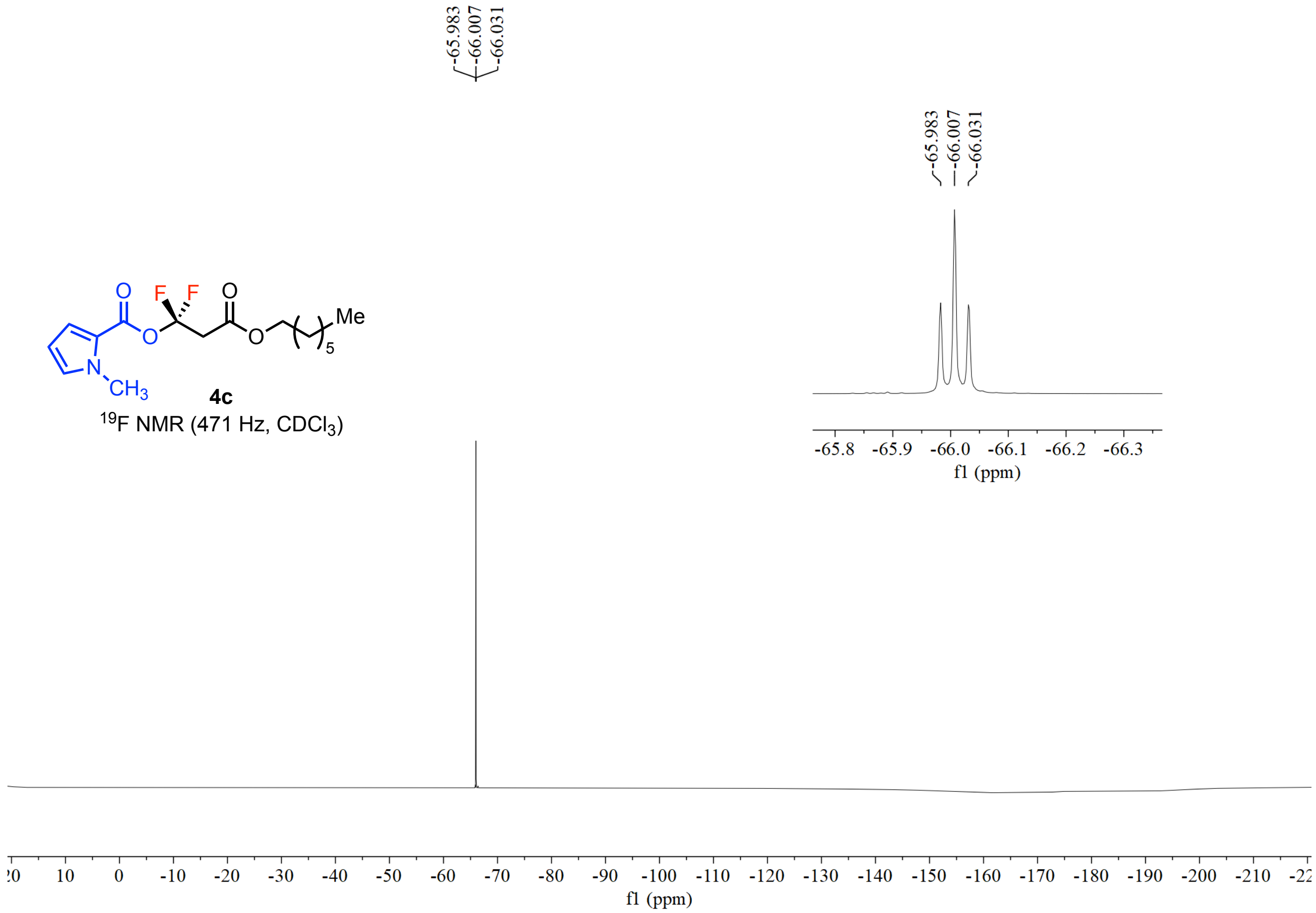
-65.983
-66.007
-66.031
4c
19F NMR (471 Hz, CDCl3)
-65.8 -65.9 -66.0 -66.1 -66.2 -66.3
f1 (ppm)
20 10 0 -10 -20 -30 -40 -50 -60 -70 -80 -90 -100 -110 -120 -130 -140 -150 -160 -170 -180 -190 -200 -210 -22
f1 (ppm)

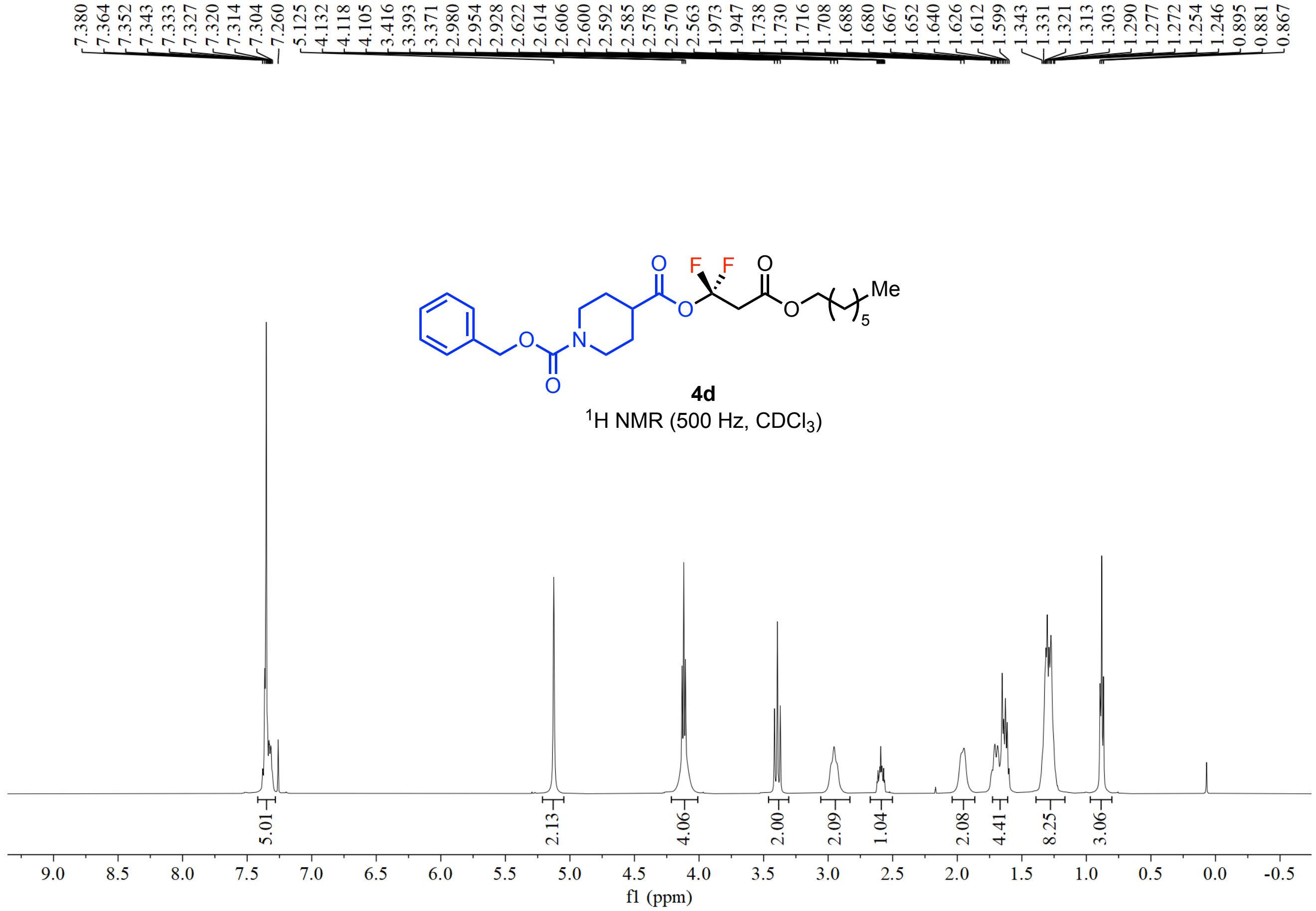
7.380 7.364 7.352 7.343 7.333 7.327 7.320 7.314 7.304 7.260 5.125 4.132 4.118 4.105 3.416 3.393 3.371 2.980 2.954 2.928 2.622 2.614 2.606 2.600 2.592 2.585 2.578 2.570 2.563 1.973 1.947 1.738 1.730 1.716 1.708 1.688 1.680 1.667 1.652 1.640 1.626 1.612 1.599 1.343 1.331 1.321 1.313 1.303 1.290 1.277 1.272 1.254 1.246 0.895 0.881 0.867
4d
1H NMR (500 Hz, CDCl3)
5.01 2.13 4.06 2.00 2.09 1.04 2.08 4.41 8.25 3.06
9.0 8.5 8.0 7.5 7.0 6.5 6.0 5.5 5.0 4.5 4.0 3.5 3.0 2.5 2.0 1.5 1.0 0.5 0.0 -0.5
f1 (ppm)

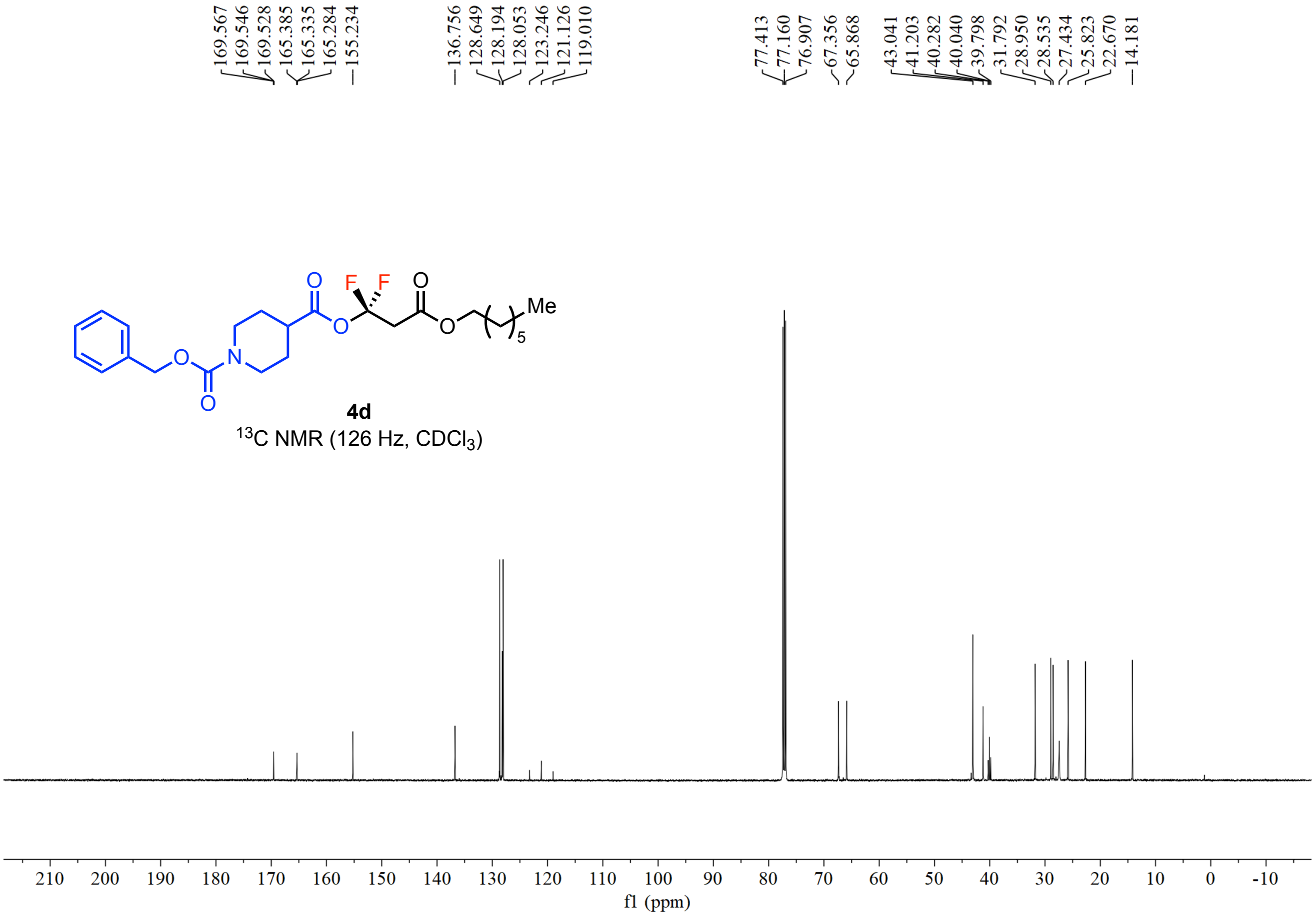
169.567
169.546
169.528
165.385
165.335
165.284
155.234
136.756
128.649
128.194
128.053
123.246
121.126
119.010
77.413
77.160
76.907
67.356
65.868
43.041
41.203
40.282
40.040
39.798
31.792
28.950
28.535
27.434
25.823
22.670
14.181
Me
5
4d
13C NMR (126 Hz, CDCl3)
f1 (ppm)

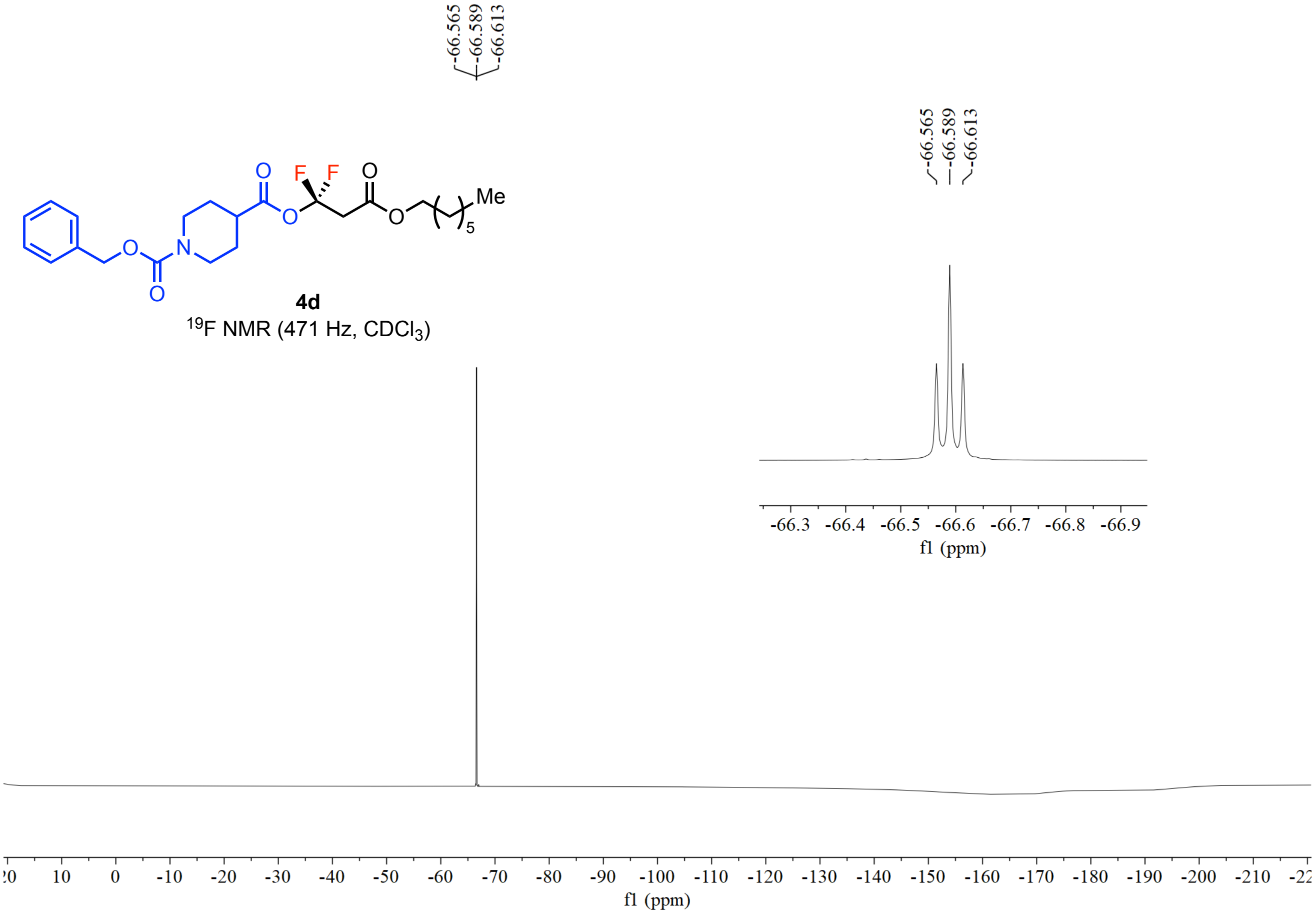
-66.565
-66.589
-66.613
Me
5
4d
19F NMR (471 Hz, CDCl3)
f1 (ppm)

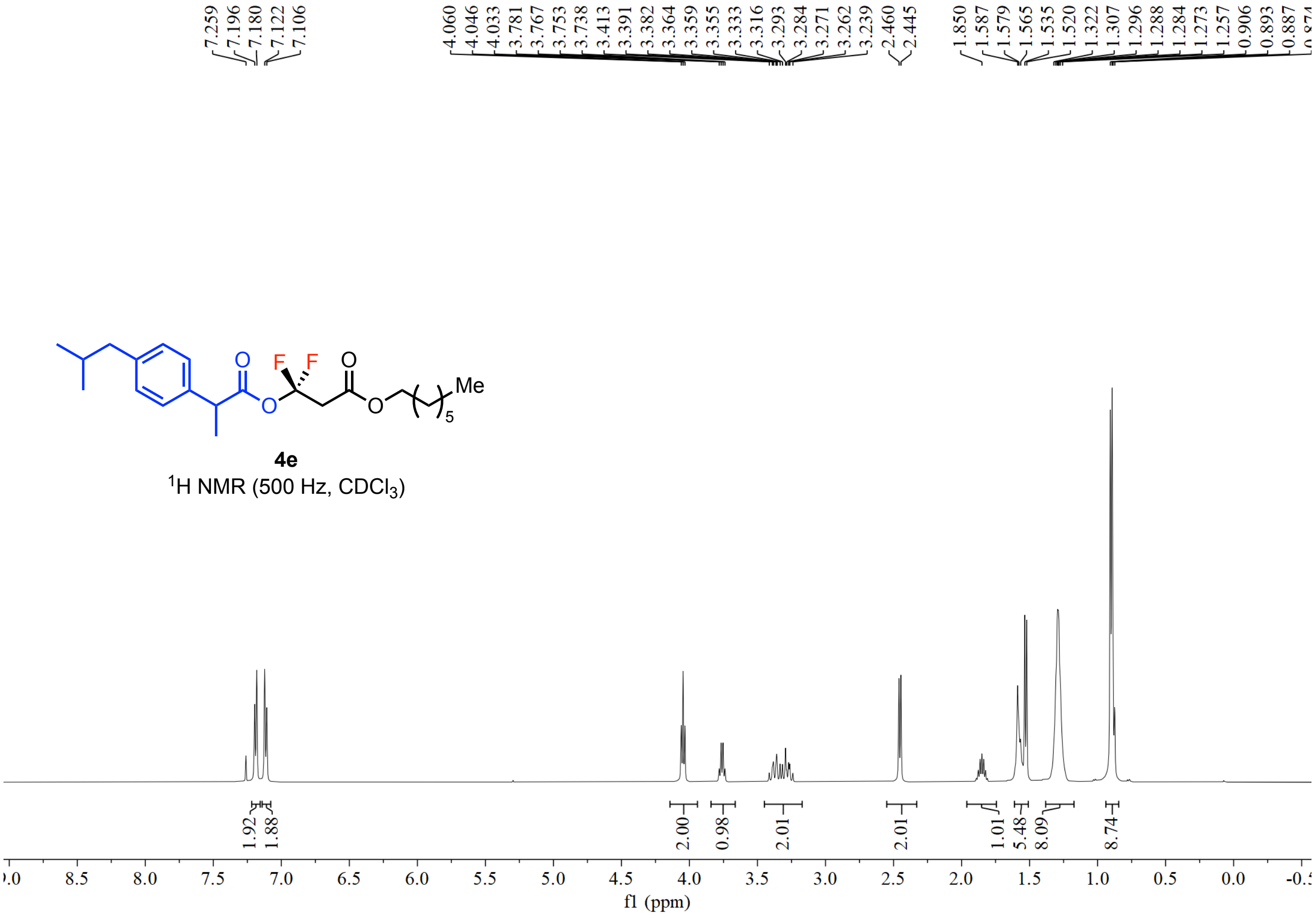
7.259
7.196
7.180
7.122
7.106
4.060
4.046
4.033
3.781
3.767
3.753
3.738
3.413
3.391
3.382
3.364
3.359
3.355
3.333
3.316
3.293
3.284
3.271
3.262
3.239
2.460
2.445
1.850
1.587
1.579
1.565
1.535
1.520
1.322
1.307
1.296
1.288
1.284
1.273
1.257
0.906
0.893
0.887
F
F
O
O
O
O
Me
5
4e
^{1}H NMR (500 Hz, CDCl$_3$)
1.92
1.88
2.00
0.98
2.01
2.01
1.01
5.48
8.09
8.74
8.5
8.0
7.5
7.0
6.5
6.0
5.5
5.0
4.5
4.0
3.5
3.0
2.5
2.0
1.5
1.0
0.5
0.0
f1 (ppm)

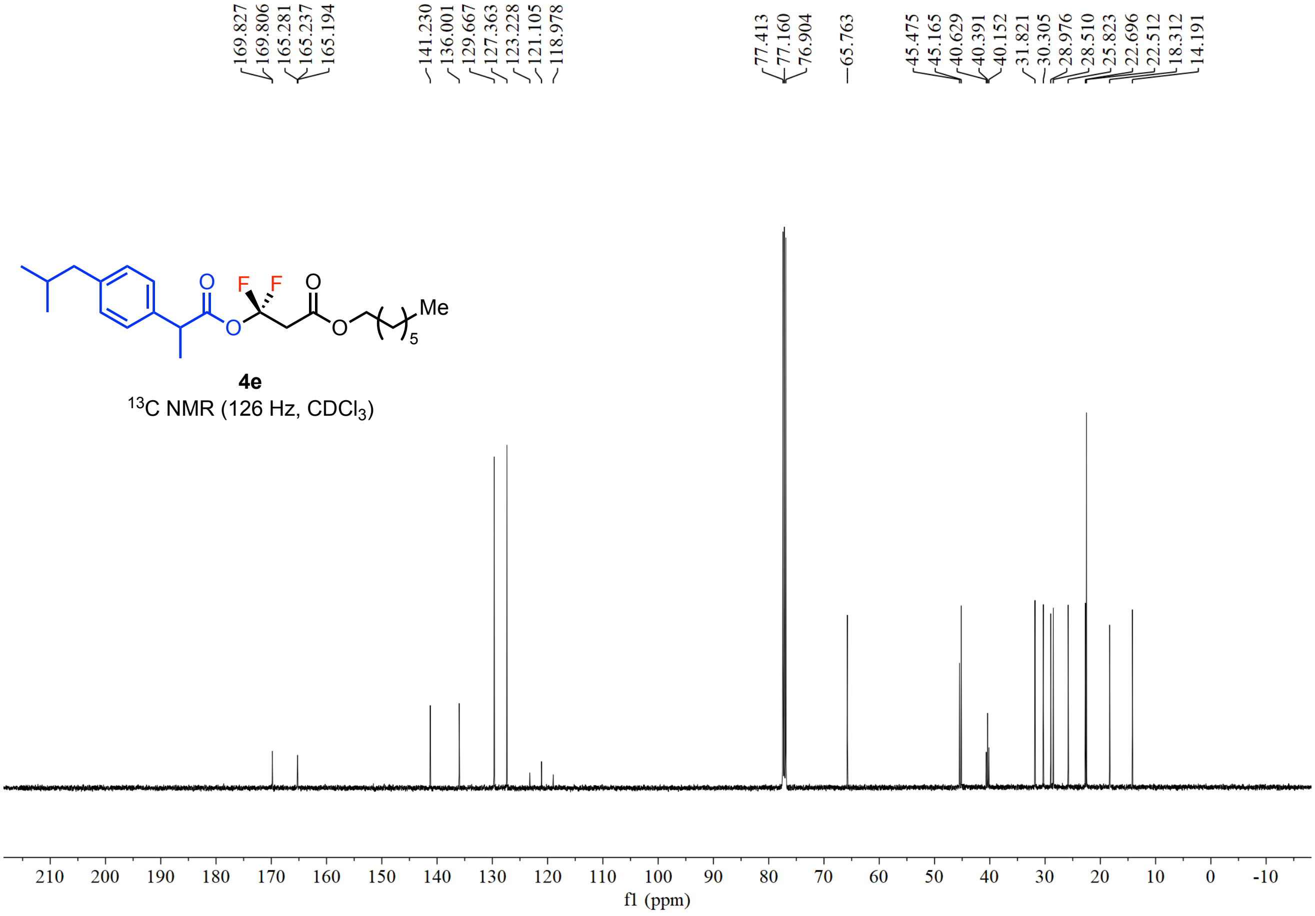
169.827
169.806
165.281
165.237
165.194
141.230
136.001
129.667
127.363
123.228
121.105
118.978
77.413
77.160
76.904
65.763
45.475
45.165
40.629
40.391
40.152
31.821
30.305
28.976
28.510
25.823
22.696
22.512
18.312
14.191
F
F
O
O
O
O
Me
5
4e
^{13}C NMR (126 Hz, CDCl$_3$)
210
200
190
180
170
160
150
140
130
120
110
100
90
80
70
60
50
40
30
20
10
0
-10
f1 (ppm)

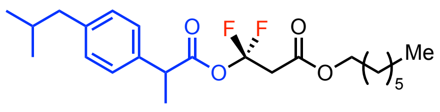

4e

^{19}F NMR (471 Hz, $CDCl_3$)

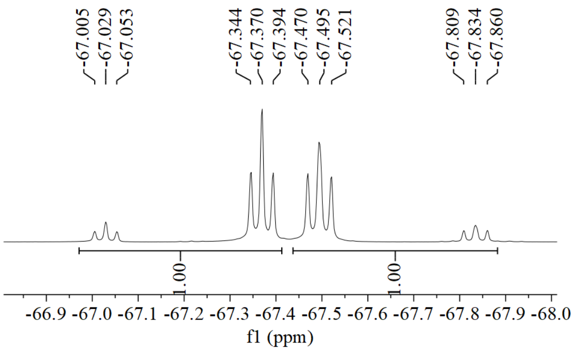

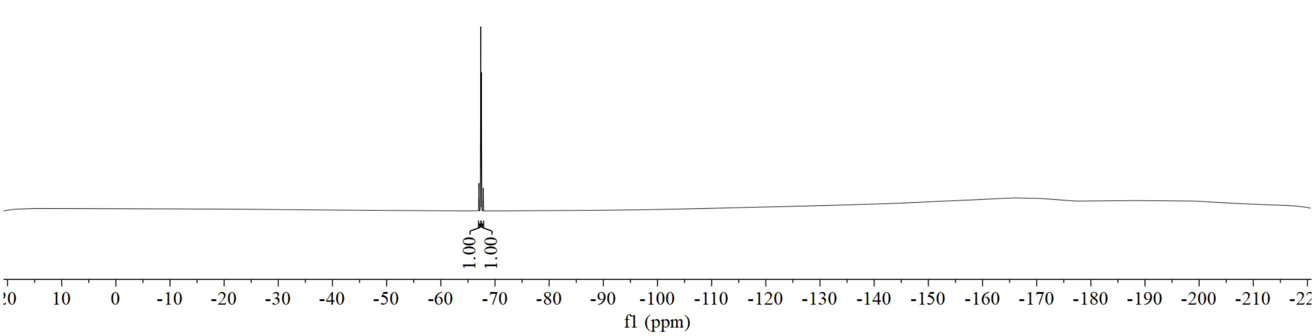

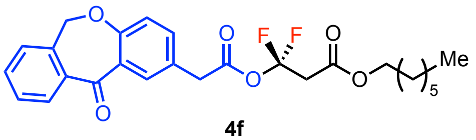

4f

^{1}H NMR (500 Hz, $CDCl_3$)

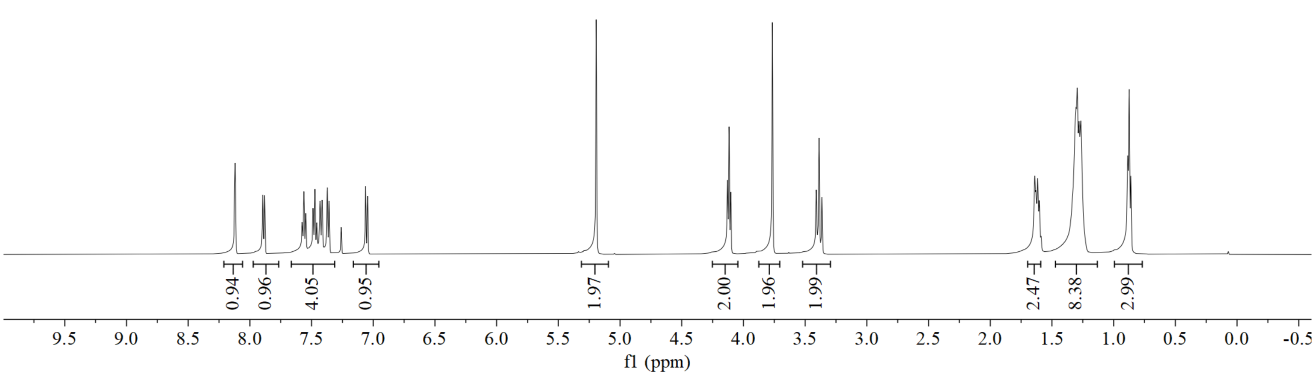

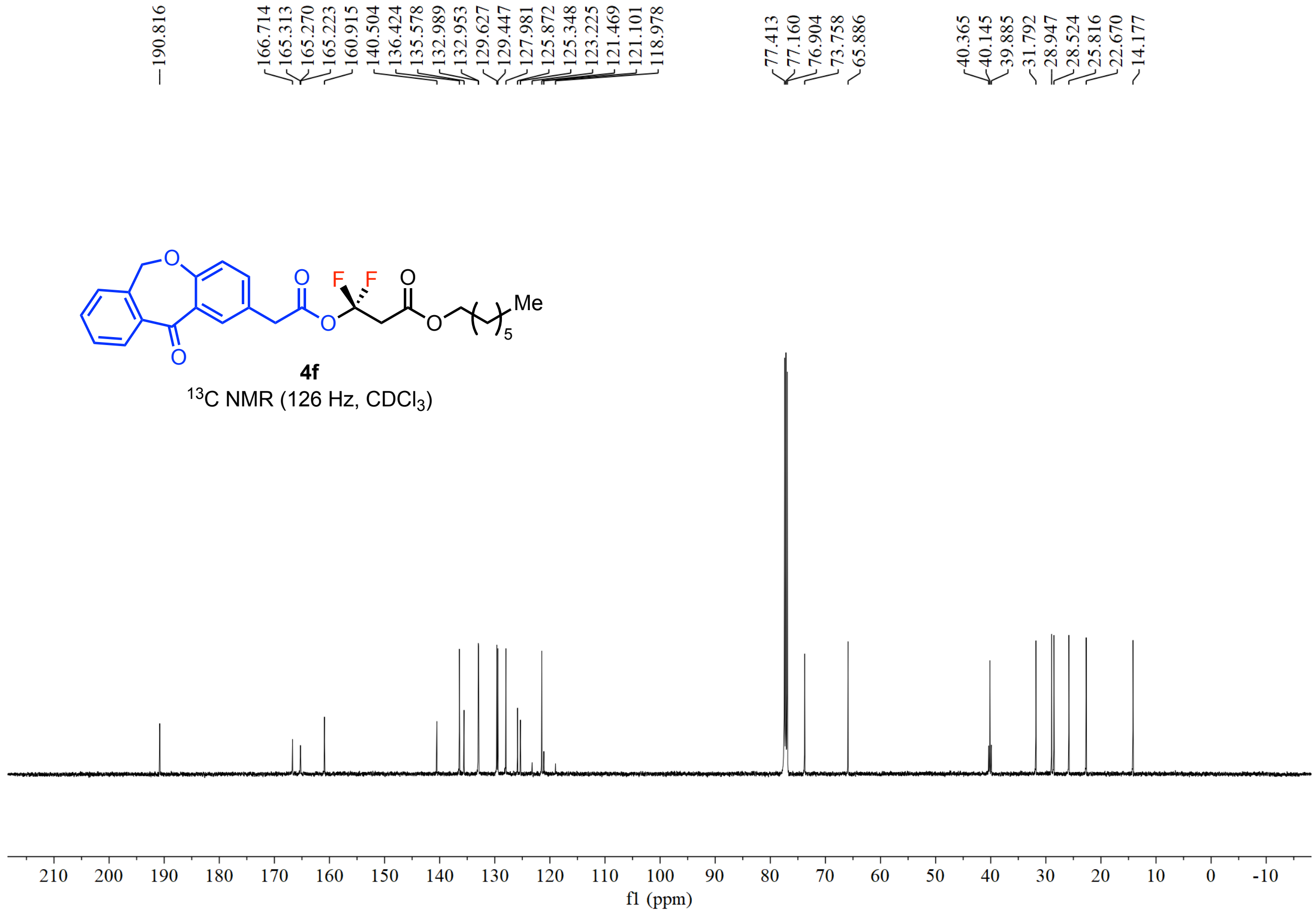
190.816
166.714
165.313
165.270
165.223
160.915
140.504
136.424
135.578
132.989
132.953
129.627
129.447
127.981
125.872
125.348
123.225
121.469
121.101
118.978
77.413
77.160
76.904
73.758
65.886
40.365
40.145
39.885
31.792
28.947
28.524
25.816
22.670
14.177
O
O
F
F
O
O
O
O
Me
5
4f
^{13}C NMR (126 Hz, CDCl$_3$)
f1 (ppm)

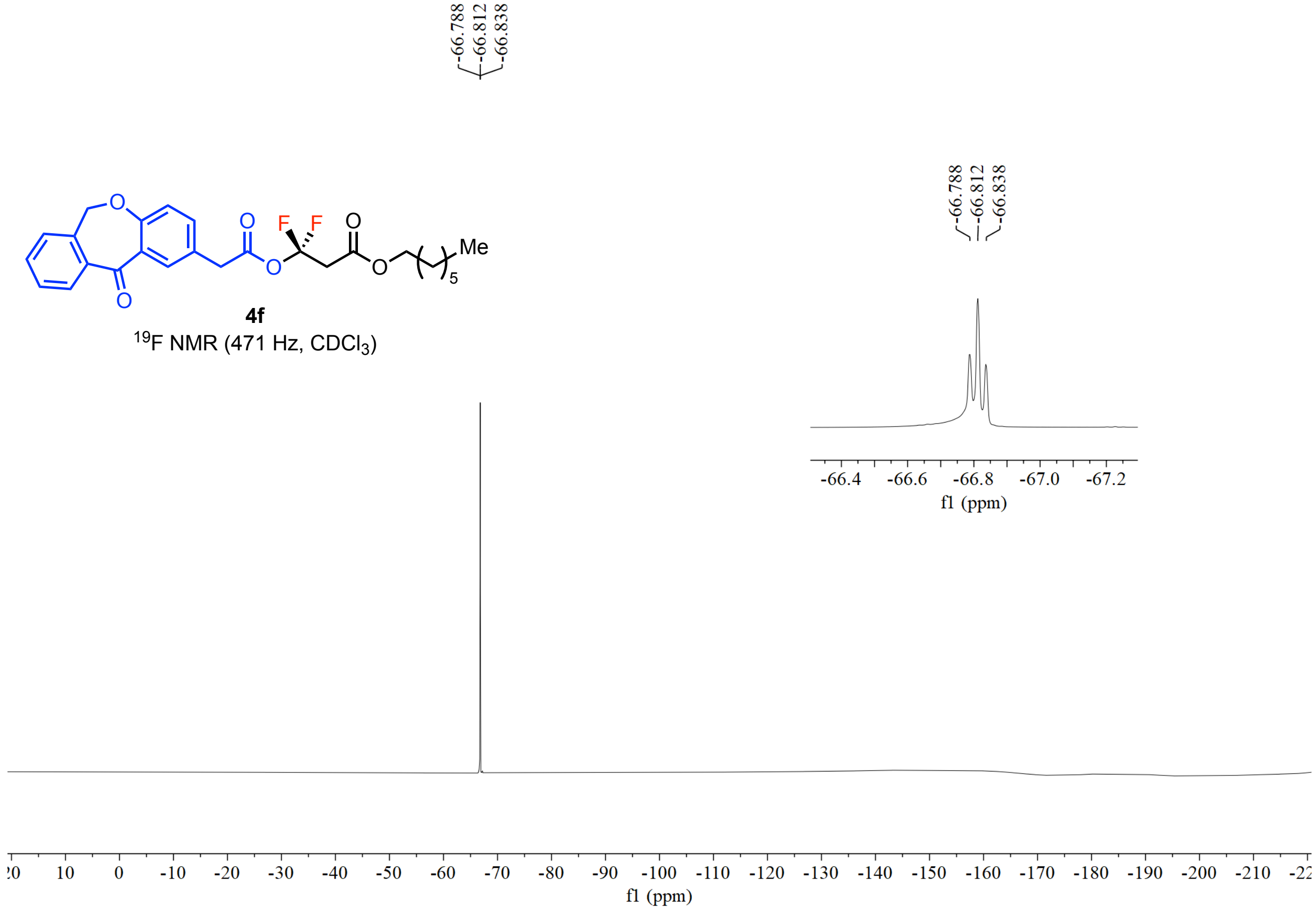
-66.788
-66.812
-66.838
O
O
F
F
O
O
O
O
Me
5
4f
^{19}F NMR (471 Hz, CDCl$_3$)
f1 (ppm)

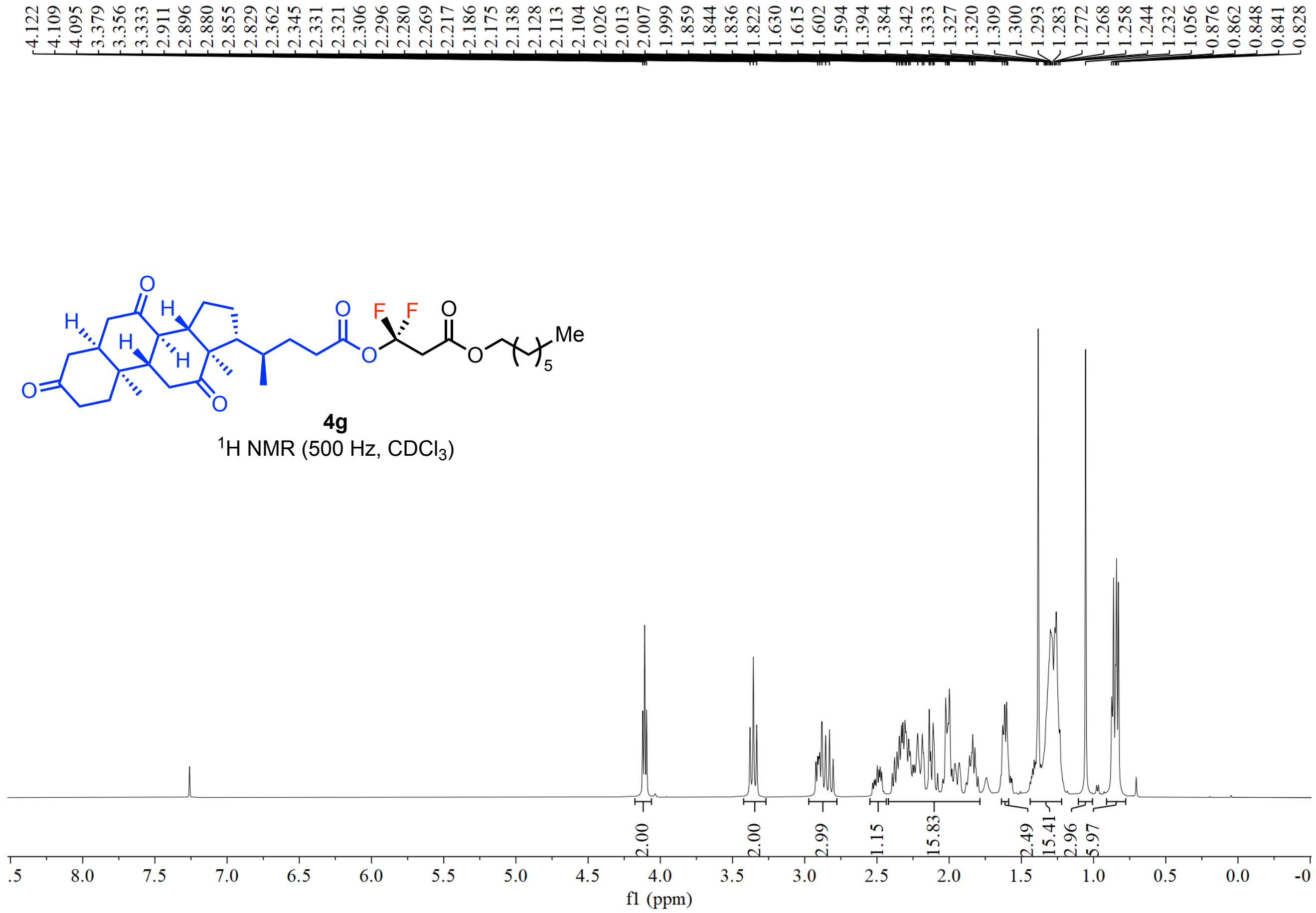
4.122 4.109 4.095 3.379 3.356 3.333 2.911 2.896 2.880 2.855 2.829 2.362 2.345 2.331 2.321 2.306 2.296 2.280 2.269 2.217 2.186 2.175 2.138 2.128 2.113 2.104 2.026 2.013 2.007 1.999 1.859 1.844 1.836 1.822 1.630 1.615 1.602 1.594 1.394 1.384 1.342 1.333 1.327 1.320 1.309 1.300 1.293 1.283 1.272 1.268 1.258 1.244 1.232 1.056 0.876 0.862 0.848 0.841 0.828
2.00 2.00 2.99 1.15 15.83 2.49 15.41 2.96 5.97
f1 (ppm)
4g
^{1}H NMR (500 Hz, $CDCl_3$)

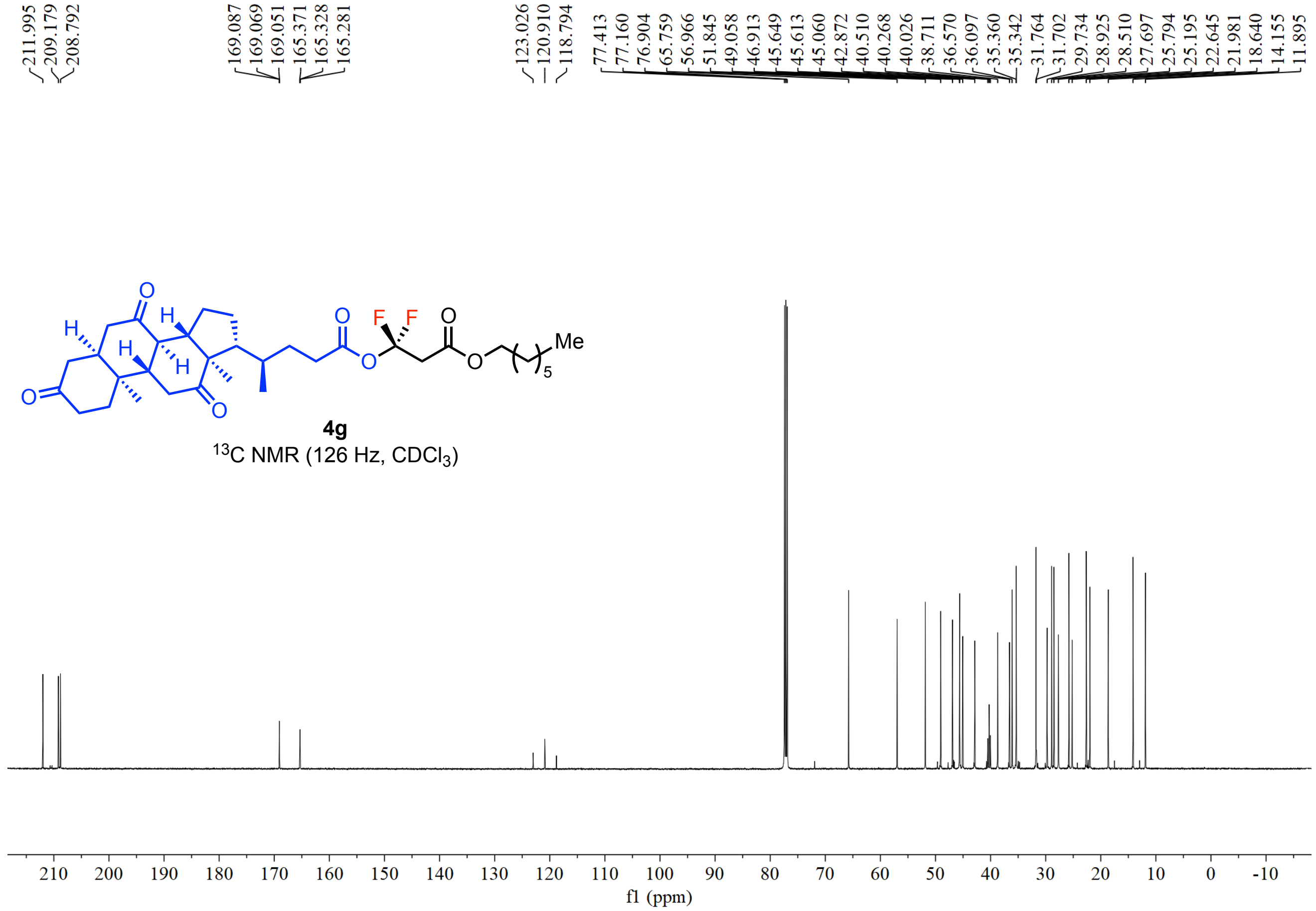
211.995 209.179 208.792 169.087 169.069 169.051 165.371 165.328 165.281 123.026 120.910 118.794 77.413 77.160 76.904 65.759 56.966 51.845 49.058 46.913 45.649 45.613 45.060 42.872 40.510 40.268 40.026 38.711 36.570 36.097 35.360 35.342 31.764 31.702 29.734 28.925 28.510 27.697 25.794 25.195 22.645 21.981 18.640 14.155 11.895
f1 (ppm)
4g
^{13}C NMR (126 Hz, $CDCl_3$)

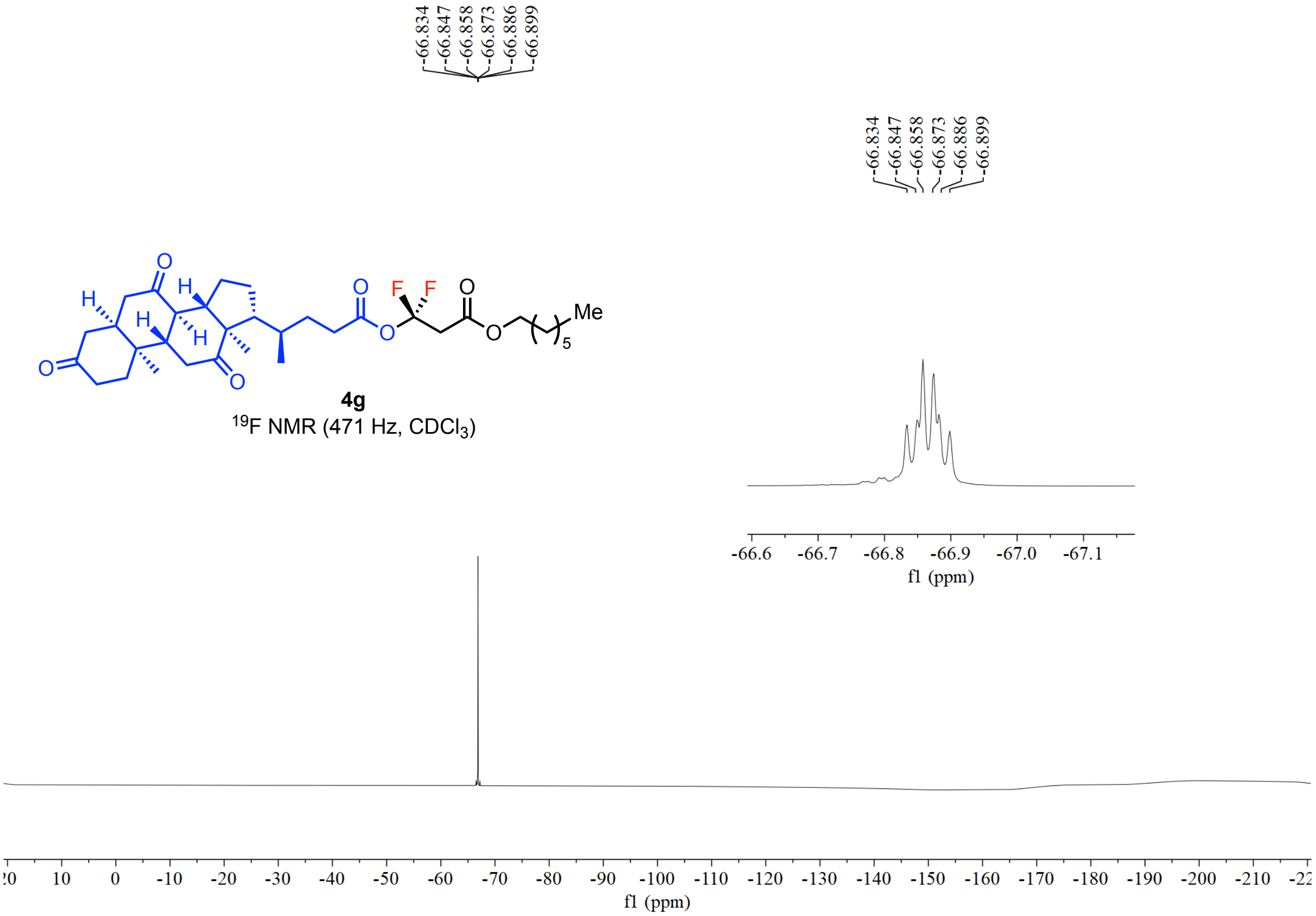
-66.834
-66.847
-66.858
-66.873
-66.886
-66.899
O
H
H
H
H
O
O
O
O
F
F
O
O
Me
5
4g
19F NMR (471 Hz, CDCl3)
f1 (ppm)

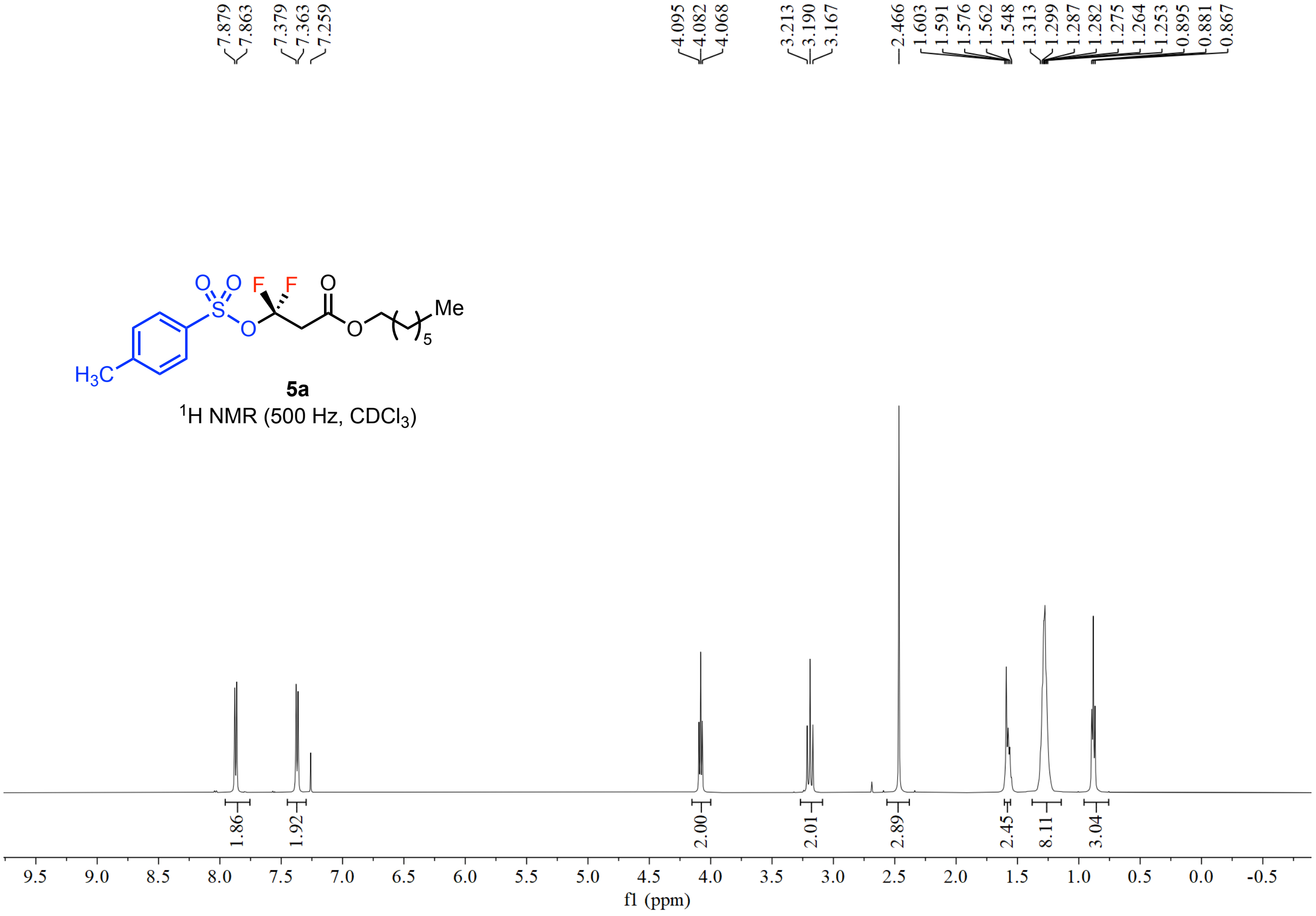
7.879
7.863
7.379
7.363
7.259
4.095
4.082
4.068
3.213
3.190
3.167
2.466
1.603
1.591
1.576
1.562
1.548
1.313
1.299
1.287
1.282
1.275
1.264
1.253
0.895
0.881
0.867
O
O
S
O
F
F
O
O
Me
5
H3C
5a
1H NMR (500 Hz, CDCl3)
1.86
1.92
2.00
2.01
2.89
2.45
8.11
3.04
f1 (ppm)

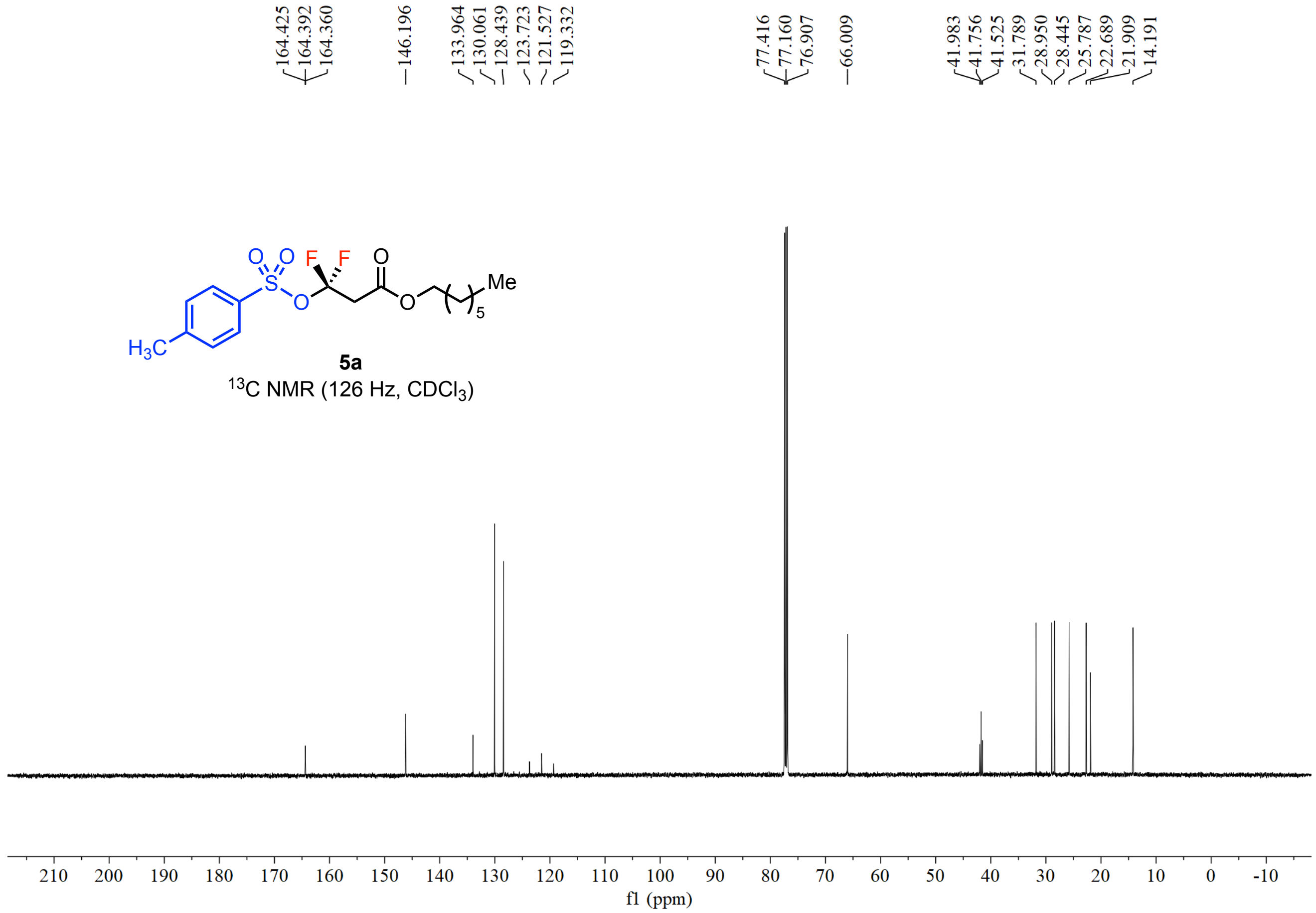
164.425
164.392
164.360
146.196
133.964
130.061
128.439
123.723
121.527
119.332
77.416
77.160
76.907
66.009
41.983
41.756
41.525
31.789
28.950
28.445
25.787
22.689
21.909
14.191
5a
13C NMR (126 Hz, CDCl3)
f1 (ppm)

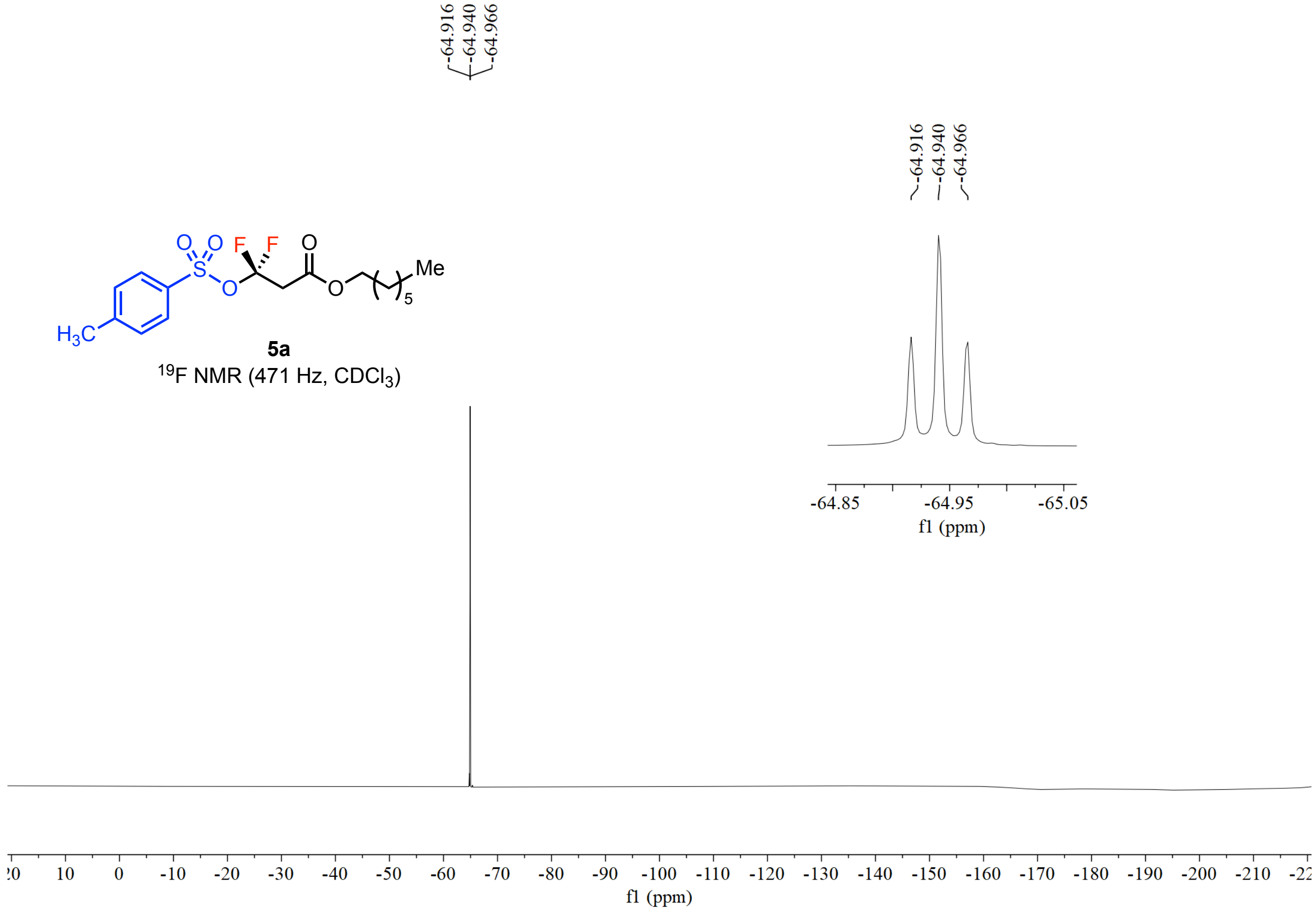
-64.916
-64.940
-64.966
5a
19F NMR (471 Hz, CDCl3)
-64.85
-64.95
-65.05
f1 (ppm)

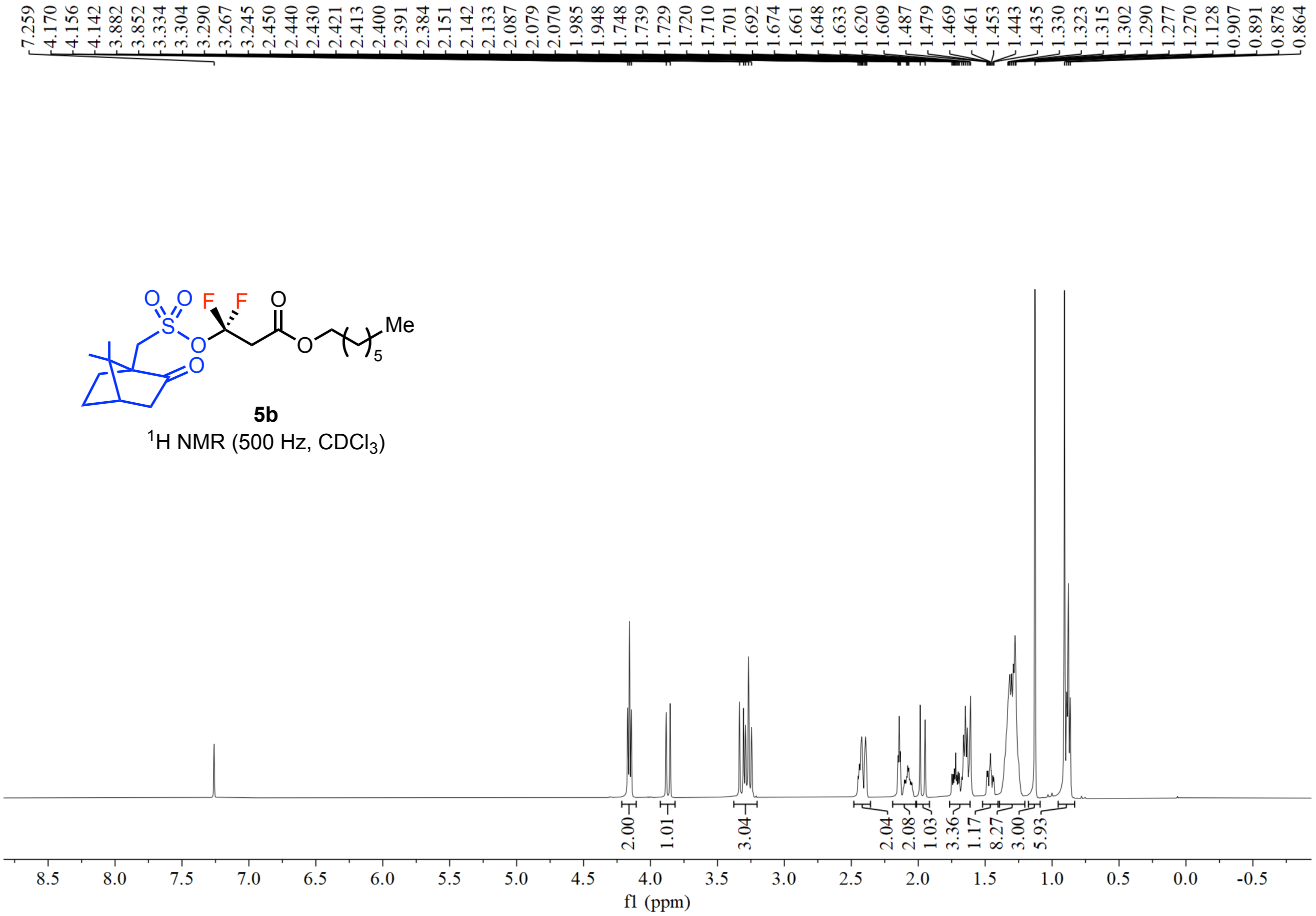
7.259
4.170
4.156
4.142
3.882
3.852
3.334
3.304
3.290
3.267
3.245
2.450
2.440
2.430
2.421
2.413
2.400
2.391
2.384
2.151
2.142
2.133
2.087
2.079
2.070
1.985
1.948
1.748
1.739
1.729
1.720
1.710
1.701
1.692
1.674
1.661
1.648
1.633
1.620
1.609
1.487
1.479
1.469
1.461
1.453
1.443
1.435
1.330
1.323
1.315
1.302
1.290
1.277
1.270
1.128
0.907
0.891
0.878
0.864
O O F F O
S
O
O
Me
O
5
5b
^{1}H NMR (500 Hz, CDCl$_3$)
2.00
1.01
3.04
2.04
2.08
1.03
3.36
1.17
8.27
3.00
5.93
8.5 8.0 7.5 7.0 6.5 6.0 5.5 5.0 4.5 4.0 3.5 3.0 2.5 2.0 1.5 1.0 0.5 0.0 -0.5
f1 (ppm)

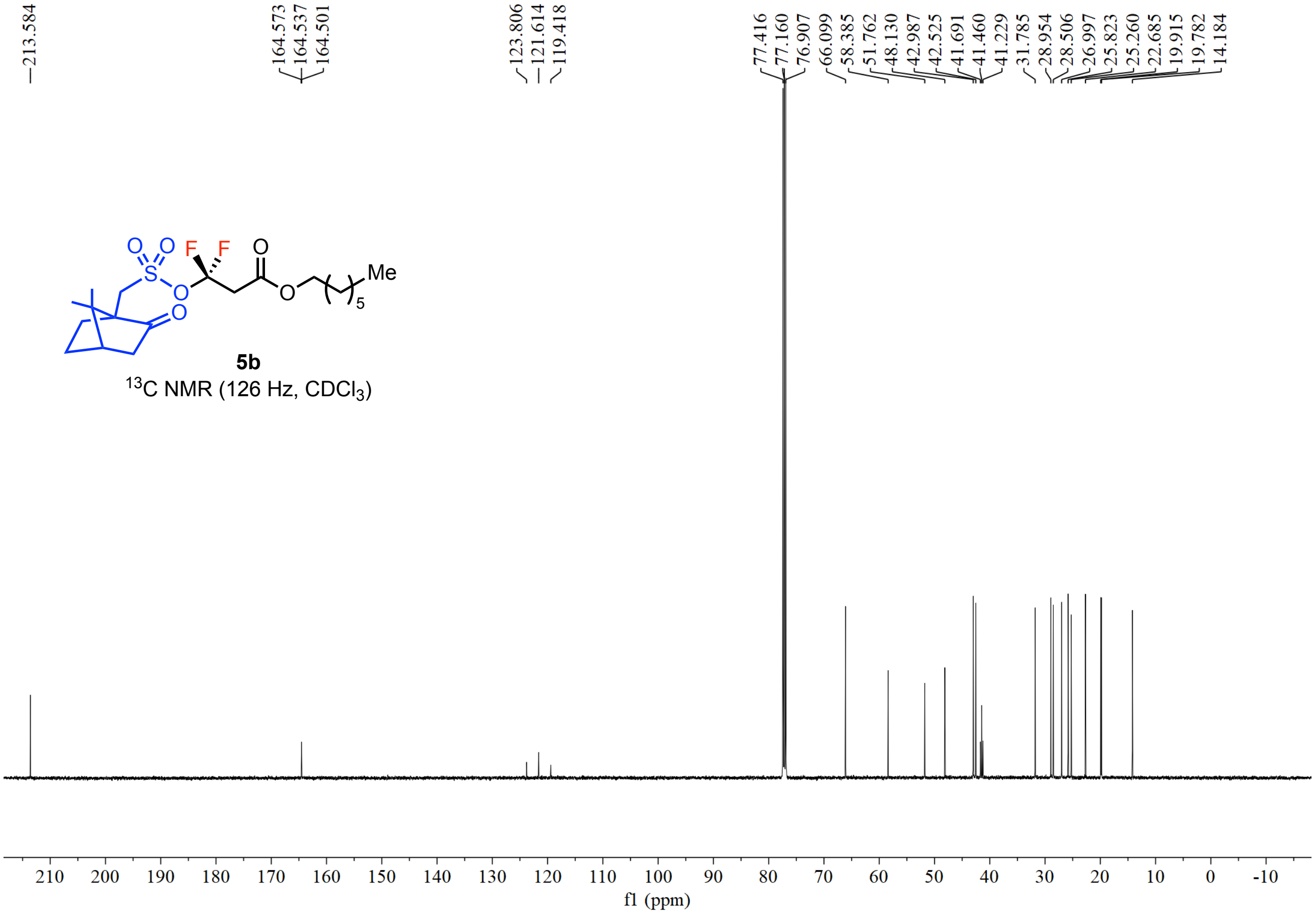
213.584
164.573
164.537
164.501
123.806
121.614
119.418
77.416
77.160
76.907
66.099
58.385
51.762
48.130
42.987
42.525
41.691
41.460
41.229
31.785
28.954
28.506
26.997
25.823
25.260
22.685
19.915
19.782
14.184
O O F F O
S
O
O
Me
O
5
5b
^{13}C NMR (126 Hz, CDCl$_3$)
210 200 190 180 170 160 150 140 130 120 110 100 90 80 70 60 50 40 30 20 10 0 -10
f1 (ppm)

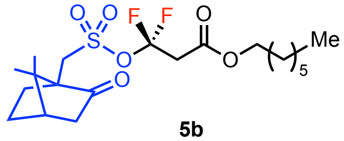

5b

^{19}F NMR (471 Hz, $CDCl_3$)

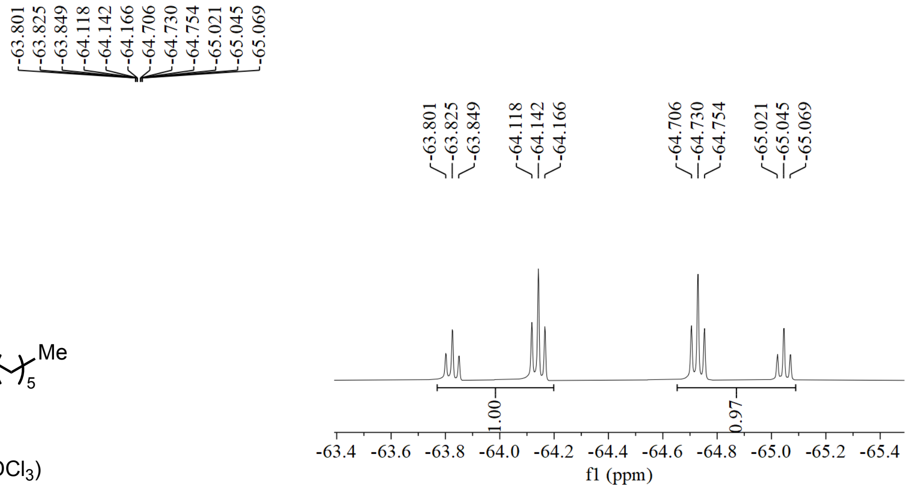

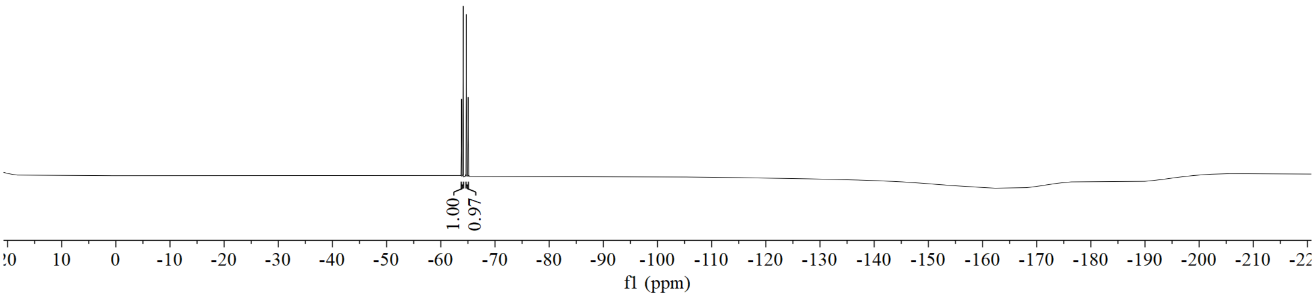

Supplement: Supplementary file 1 — ol4c00095_si_001.pdf [file ol4c00095_si_001.pdf]
